# Downward trends in the global burden of congenital complete hearing loss in children younger than five years from 1990 to 2030

**DOI:** 10.7189/jogh.13.04120

**Published:** 2023-10-13

**Authors:** Jian Xiao, Xiajing Liu, Wenwei Cheng, Jing Liu, Junyi Jiang, Heqing Li, Yexun Song

**Affiliations:** 1 Department of Otolaryngology-Head and Neck Surgery, The Third Xiangya Hospital of Central South University, Changsha, Hunan Province, China; 2 Xiangya School of Public Health, Central South University, Changsha, Hunan Province, China; 3 Department of Nephrology, Union Hospital, Tongji Medical College, Huazhong University of Science and Technology, Wuhan, China; 4 Institute of Clinical Pharmacology, Central South University, Hunan Key Laboratory of Pharmacogenetics, Changsha, Hunan Province, China

## Abstract

**Background:**

The global epidemiological data on congenital hearing loss in children is sparse. We aimed to analyse the trends in the burden of complete hearing loss caused by congenital birth defects in children younger than five years from 1990 to 2030.

**Methods:**

Using data from the Global Burden of Disease (GBD) Study 2019, we reported the counts and rates of prevalence and years lived with disability (YLD) by age, sex, and sociodemographic index (SDI). We also forecasted the prevalence rates until 2030 through the autoregressive integrated moving average (ARIMA) and Bayesian age-period-cohort (BAPC) models.

**Results:**

We observed a global prevalence rate of 15.4 (95% uncertainty interval (UI) = 5.8 to 33.8) and a YLD rate of 3.3 (95% UI = 1.1 to 7.1) per 100 000 population in 2019, with both showing downward trends from 1990 to 2019. Regionally, Oceania had the highest prevalence (47.2; 95% UI = 18.8 to 96.6) and YLD (10; 95% UI = 3.2 to 22.8) rates, while Central Europe had the lowest rates. Nationally, the prevalence (85.0; 95% UI = 36.8 to 166.8) and YLD (17.9; 95% UI = 6.6 to 36.9) rates were highest in Myanmar and lowest in Peru. Only the United States of America (2.6%; 95% UI = -4.6 to 14.4) and Norway (0.6%; 95% UI = -6.7 to 16.2) showed upward trends. Compared to girls, the prevalence and YLD rates were higher for boys at global, regional, and five SDI quintile levels, except for Eastern Sub-Saharan Africa. At the global level, downward trends were predicted in prevalence rates from 2019 to 2030 between boys and girls.

**Conclusions:**

Although the global burden of childhood congenital complete hearing loss showed inequalities across locations, sexes, and age groups, we found decreases in the global prevalence rates between 1990 and 2019 and predicted decreases from 2019 to 2030. Better prevention of infectious aetiologies, improving genetic diagnoses, and hearing restoration could alleviate this burden.

Congenital hearing loss can become a chronic condition and progressively worsen if untreated. Children with the condition have difficulties with language and speech acquisition [1], as well as multiple cognitive functions and social development [2]. Additionally, childhood hearing loss is related to anxiety and depression, as well as decreased subjective well-being and self-esteem [3]. Children with complete hearing loss cannot hear any speech or sound in a noisy environment [4], have significantly lower levels of language and literacy development than their normally hearing peers, and show especially poor performance in spoken word learning [5].

The importance of studying congenital hearing loss is increasingly being recognised. The Joint Committee on Infant Hearing published position statements in 2000 and 2007 to establish guidelines for early hearing detection and intervention [6,7]. Despite its detrimental effects on development, the ability to communicate, education and cognitive functions, most current studies only describe the prevalence and temporal trends of hearing loss [8,9], and few studies focus on congenital hearing loss specifically. Due to absence of recent data, particularly from low- and middle-income countries [10], the true prevalence of congenital hearing loss in children might be unknown, necessitating global estimates to provide insight into the burden and trends.

To the best of our knowledge, this is the first study to use estimates from the GBD Study 2019 to provide a retrospective and predictive assessment of the prevalence and years lived with disability (YLD) of complete hearing loss caused by congenital birth defects in children younger than five years from 1990 to 2030 and to stratify the analysis by age group, sex, and sociodemographic index (SDI), as well as to estimate global downward trends from 2019 to 2030 by autoregressive integrated moving average (ARIMA) and Bayesian age-period-cohort (BAPC) models.

## METHODS

### Study data

We extracted data on the burden of complete hearing loss caused by congenital birth defects through an online query tool from the Institute for Health Metrics and Evaluation (IHME) website [11]. During data retrieval and downloading, we limited the age of individuals to younger than five years, which were then subdivided into four groups (early neonatal (0-6 days), late neonatal (7-27 days), post-neonatal (28-364 days), and young children (1-4 years). The definition and audiometric thresholds of complete hearing loss are available elsewhere [8]. We coded congenital hearing loss as H90.501, and congenital ear deformities as Q16.000, Q16.100, Q16.101, Q16.102, Q16.103, Q16.200, Q16.300, Q16.301, Q16.400, Q16.400x001, Q16.401, Q16.500, Q16.500x002, Q16.501, Q16.900, Q16.900x002, and Q16.901, according to the 10th revision of the International Classification of Diseases and Injuries (ICD-10).

### Statistical analysis

We used counts and rates per 100 000 population with 95% uncertainty intervals (UIs) to estimate the burden of hearing loss. We measured the burden of hearing loss via prevalence and YLD and analysed it by age, sex, year, location, and SDI. We also calculated the percentage change in counts and rates between 1990 and 2019.

We applied the ARIMA and BAPC models (details available elsewhere [12]) to predict the prevalence rate from 2019 to 2030 for both sexes. The BAPC model has been proven to have higher accuracy in predicting the disease burden, so we used it within an integrated nested Laplacian approximation (R packages BAPC and INLA) to project the prevalence rates from 2019 to 2030 [13,14]. The general methodology of the GBD Study 2019 and supplementary methods for hearing loss burden have been explained elsewhere [8,15-17]. We followed the Guidelines for Accurate and Transparent Health Estimates Reporting (GATHER) for cross-sectional studies [18] in conducting this research.

We conducted all analyses in R, version 4.2.2 (R Core Team, Auckland, New Zealand) and SPSS, version 25.0 (IBM Corporation, New York, USA). *P*-values <0.05 were considered statistically significant.

## RESULTS

### Global level

There were 101 907 (95% UI = 38199, 223 910) complete hearing loss prevalent cases caused by congenital birth defects among children younger than five years and 21 615 (95% UI = 7387, 47 303) YLD globally in 2019, resulting in a prevalence rate of 15.4 (95% UI = 5.8, 33.8) and a YLD rate of 3.3 (95% UI = 1.1, 7.1) per 100 000 population (**Table 1**). Between 1990 and 2019, the global prevalence rate decreased by -45.6% (95% UI = -51.4, -41.2) and the YLD rate by -45.3% (95% UI = -52.1, -39.8), while the total number of prevalent cases decreased by -43.0% (95% UI = -49.0, -38.8) and the number of YLD by -42.6% (95% UI = -49.8, -36.9) (Table S1 in the **Online Supplementary Document**).

**Table 1:** The prevalence counts and YLDs for complete hearing loss caused by congenital birth defects in children younger than five years in 2019 for both sexes, and percentage change of rates by Global Burden of Disease regions from 1990 to 2019

	Prevalence (95% UI)			YLD (95% UI)		
	**Counts**	**Rates per 100 000 population (95% UI)**	**Percentage change in rates per 100 000 population (95%UI)**	**Counts**	**Rates per 100 000 population (95% UI)**	**Percentage change in rates per 100 000 population (95% UI)**
**Global – both sexes**	101 907 (381 99 to 223 910)	15.4 (5.8 to 33.8)	-45.6 (-51.4 to -41.2)	21 615 (7387 to 47 303)	3.3 (1.1 to 7.1)	-45.3 (-52.1 to -39.8)
Global – male	55752 (209 78 to 119871)	16.3 (6.1 to 35.0)	-46.9 (-52.3 to -42.6)	11 820 (4072 to 25 625)	3.5 (1.2 to 7.5)	-46.4 (-53.7 to -40.0)
Global – female	46154 (171 30 to 102989)	14.4 (5.4 to 32.1)	-44.4 (-50.1 to -39.3)	9794 (3340 to 22 077)	3.1 (1.0 to 6.9)	-43.8 (-51.6 to -37.4)
**High SDI**	2991 (842 to 8076)	5.7 (1.6 to 15.4)	-19.6 (-25.1 to -15.8)	641 (172 to 1791)	1.2 (0.3 to 3.4)	-19.7 (-26.4 to -15.1)
**High-middle SDI**	9078 (3302 to 20952)	11 (4 to 25.4)	-45.2 (-52.5 to -39.9)	1936 (653 to 4613)	2.3 (0.8 to 5.6)	-44.9 (-57.3 to -33.8)
**Middle SDI**	28 089 (10 631 to 62 094)	15.2 (5.8 to 33.7)	-50 (-56.3 to -45.3)	5993 (2059 to 13164)	3.3 (1.1 to 7.1)	-49.6 (-59.1 to -41.4)
**Low-middle SDI**	242 65 (8860 to 53 858)	14.1 (5.1 to 31.3)	-57 (-63.2 to -52.1)	5167 (1795 to 11 718)	3 (1 to 6.8)	-56.3 (-64.4 to -49.2)
**Low SDI**	37 395 (14 169 to 77 821)	21.9 (8.3 to 45.5)	-41.3 (-46.1 to -37.4)	7859 (2819 to 17 043)	4.6 (1.6 to 10)	-41 (-48.8 to -34.1)
**Andean Latin America**	190 (42 to 573)	3 (0.7 to 9)	-29.7 (-49 to -20.9)	41 (9 to 123)	0.6 (0.1 to 1.9)	-29.7 (-49 to -20.9)
**Australasia**	71 (16 to 211)	3.9 (0.9 to 11.6)	-22.9 (-41.4 to -9.8)	15 (3 to 44)	0.8 (0.2 to 2.4)	-23 (-41.4 to -9.8)
**Caribbean**	173 (42 to 448)	4.4 (1.1 to 11.3)	-30.1 (-48.3 to -20.2)	37 (9 to 106)	0.9 (0.2 to 2.7)	-30.1 (-48.3 to -20.2)
**Central Asia**	429 (99 to 1279)	4.5 (1 to 13.4)	-36.6 (-50 to -27.6)	92 (21 to 265)	1 (0.2 to 2.8)	-36.6 (-50 to -27.6)
**Central Europe**	134 (26 to 437)	2.4 (0.5 to 7.7)	-44.8 (-67.7 to -34.6)	29 (5 to 94)	0.5 (0.1 to 1.7)	-44.8 (-67.7 to -34.6)
**Central Latin America**	773 (189 to 2084)	3.6 (0.9 to 9.6)	-41.8 (-56.3 to -33.5)	166 (40 to 478)	0.8 (0.2 to 2.2)	-41.8 (-56.3 to -33.5)
**Central Sub-Saharan Africa**	2404 (855 to 5389)	11.6 (4.1 to 26)	-44 (-55.1 to -33.7)	515 (166 to 1181)	2.5 (0.8 to 5.7)	-43.4 (-62.5 to -17.5)
**East Asia**	19 975 (7695 to 42 933)	23.7 (9.1 to 51)	-45.2 (-53.4 to -40.3)	4266 (1424 to 9383)	5.1 (1.7 to 11.2)	-44.7 (-57.4 to -33.8)
**Eastern Europe**	324 (63 to 1061)	2.6 (0.5 to 8.6)	-37 (-59 to -28)	69 (12 to 233)	0.6 (0.1 to 1.9)	-37 (-59 to -28)
**Eastern Sub-Saharan Africa**	17 448 (6888 to 35 222)	27.2 (10.7 to 54.9)	-45.9 (-51.8 to -41.8)	3685 (1315 to 7891)	5.7 (2.1 to 12.3)	-45.6 (-54.6 to -36)
**High-income Asia Pacific**	364 (85 to 1025)	5 (1.2 to 14.1)	-33.1 (-48.2 to -24.8)	78 (18 to 225)	1.1 (0.2 to 3.1)	-33.1 (-48.2 to -24.8)
**High-income North America**	1074 (213 to 3294)	5.1 (1 to 15.7)	0.2 (-6.1 to 10)	230 (45 to 707)	1.1 (0.2 to 3.4)	0.2 (-6.1 to 10)
**North Africa and Middle East**	2063 (421 to 6717)	3.5 (0.7 to 11.2)	-57 (-72.3 to -45.6)	442 (81 to 1448)	0.7 (0.1 to 2.4)	-57 (-72.3 to -45.6)
**Oceania**	874 (348 to 1788)	47.2 (18.8 to 96.6)	-20.3 (-29.4 to -11.5)	185 (59 to 421)	10 (3.2 to 22.8)	-19.8 (-51.6 to 24.6)
**South Asia**	12129 (3521 to 31 354)	7.4 (2.1 to 19.1)	-65 (-76.9 to -56.1)	2598 (745 to 6887)	1.6 (0.5 to 4.2)	-64.2 (-77 to -53.3)
**Southeast Asia**	18 971 (7447 to 38632)	34.8 (13.7 to 70.9)	-48.4 (-55.3 to -43.7)	4026 (1500 to 8571)	7.4 (2.8 to 15.7)	-48 (-57.8 to -39.9)
**Southern Latin America**	424 (114 to 1136)	8.7 (2.3 to 23.4)	-27.2 (-38.5 to -18.5)	91 (25 to 245)	1.9 (0.5 to 5)	-27.2 (-38.5 to -18.5)
**Southern sub-Saharan Africa**	2224 (850 to 4598)	27.5 (10.5 to 56.8)	-44.8 (-50.8 to -39.3)	473 (168 to 1049)	5.8 (2.1 to 13)	-44.7 (-56.7 to -32.7)
**Tropical Latin America**	564 (149 to 1399)	3.5 (0.9 to 8.7)	-41.9 (-51.6 to -35.9)	120 (32 to 313)	0.7 (0.2 to 1.9)	-41.9 (-51.6 to -35.9)
**Western Europe**	1463 (453 to 3654)	6.6 (2.1 to 16.6)	-10.5 (-17.1 to 3.2)	313 (90 to 842)	1.4 (0.4 to 3.8)	-10.5 (-17.5 to 2.8)
**Western sub-Saharan Africa**	19 836 (7750 to 40 170)	27.3 (10.7 to 55.2)	-34 (-39.2 to -29.4)	4143 (1494 to 8952)	5.7 (2.1 to 12.3)	-33.8 (-44.2 to -24.8)

SDI – sociodemographic index, UI – uncertainty interval, YLD – years lived with disability

### Regional level

East Asia (n = 19 975; 95% UI = 7695, 42 933) and Australasia (n = 71; 95% UI = 16, 211) had the highest and lowest numbers of prevalent cases in 2019, respectively, as well as the highest and lowest number of YLD (East Asia: n = 4266; 95% UI = 1424, 9383 vs Australasia: n = 15; 95% UI = 3, 44) (**Table 1**). The prevalence and YLD was higher among males than among females in all regions in 2019, except for Eastern Sub-Saharan Africa (Figures S1-2 in the **Online Supplementary Document**).

Between 1990 and 2019, the percentage change in prevalence and YLD counts decreased in most of the 21 GBD regions among males and females except for central sub-Saharan Africa, Oceania, and western sub-Saharan Africa (Figures S5-6 in the **Online Supplementary Document**). Among the 21 regions included in the GBD Study 2019, Oceania (47.2; 95% UI = 18.8, 96.6) and Central Europe (95% UI = 2.4; 0.5, 7.7) had the highest and lowest prevalence rates, as well as the highest and lowest YLD rates (Oceania: 10; 95% UI = 3.2 to 22.8 vs Central Europe: 0.5; 95% UI = 0.1, 1.7), respectively (**Table 1**, Figure S1 in the **Online Supplementary Document**). The prevalence and YLD rates in 2019 were higher for males compared to females in all GBD regions except for eastern sub-Saharan Africa (Figures S3 and S4 in the **Online Supplementary Document**).

Between 1990 and 2019, the percentage changes in prevalence and YLD rates decreased in most of the 21 GBD regions among males and females, except for females in high-income North America (Figures S7-8 in the **Online Supplementary Document**).

### SDI regional level

**Figure 1:**
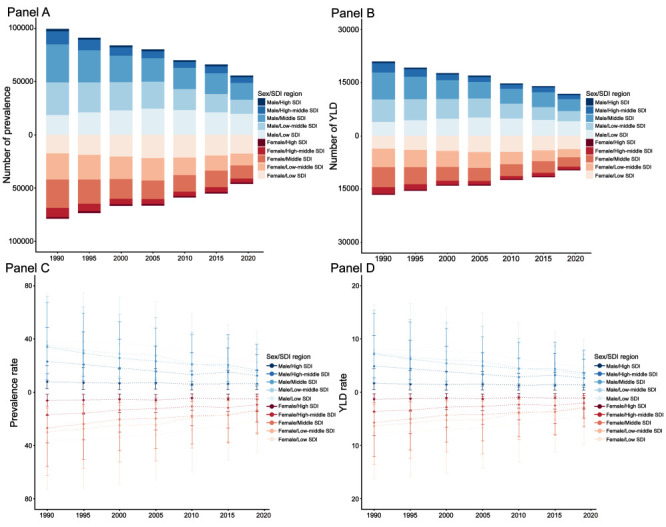
Temporal trends in counts and rates of prevalence and YLD in 5 SDI regions from 1990 to 2019, stratified by sex. **Panel A.** Prevalence counts. **Panel B.** YLD counts. **Panel C.** Prevalence rates. **Panel D.** YLD rates. YLD – years lived with disability.

In 2019, the highest prevalence and YLD counts and rates were all in the low SDI region, while the lowest counts and rates were in the high SDI region. We observed downward trends in prevalence and YLD counts from 1990 to 2019 in most of the five SDI quintiles except for the low SDI region, which had upward trends from 1990 to 2005, followed by downward trends from 2005 to 2019. The prevalence and YLD rates of all five SDI regions decreased from 1990 to 2019 (**Figure 1** and Figures S9-12 in the **Online Supplementary Document**).

### National level

**Figure 2:**
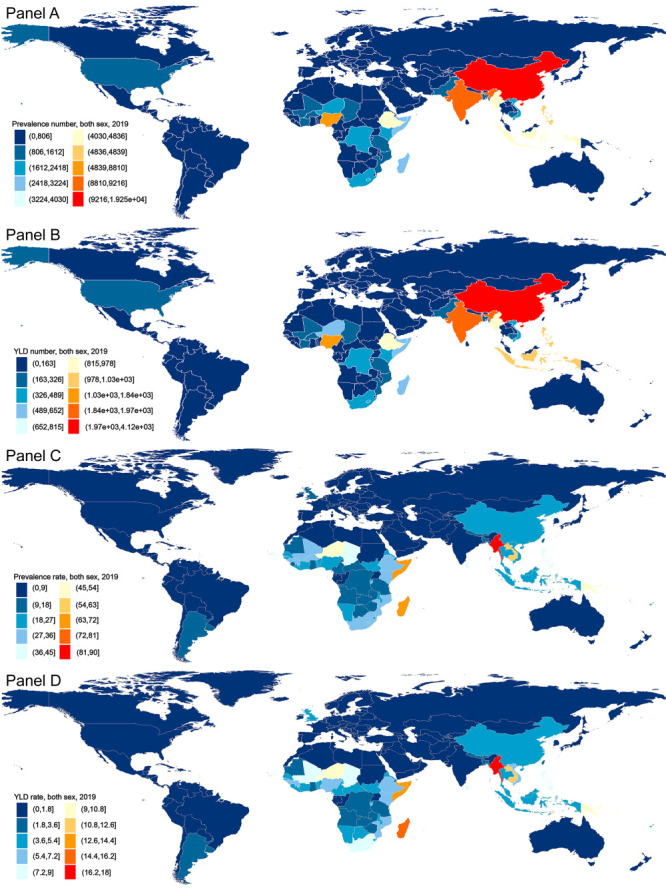
The counts and rates of prevalence and YLD for both sexes in 2019 in 204 countries and territories. **Panel A.** Prevalence counts. **Panel B.** YLD counts. **Panel C.** Prevalence rates. **Panel D.** YLD rates. YLD – years lived with disability.

At the national level in 2019, China had the highest number of prevalent cases (n = 19 254; 95% UI = 7406, 41 555), while Andorra had the lowest (n = 0; 95% UI = 0, 0) (**Figure 2**, panel A and Table S2 in the **Online Supplementary Document**). China had the highest number of YLD (n = 4115; 95% UI = 1394, 9038) and American Samoa the lowest (n = 0; 95% UI = 0, 0) (**Figure 2**, panel B and Table S2 in the **Online Supplementary Document**).

The percentage change in prevalence and YLD counts from 1990 to 2019 differed substantially among the 204 countries and territories. Somalia had the largest increases for prevalence and YLD counts from 1990 to 2019, while we found an opposite trend in Saudi Arabia, with largest decreases in prevalence and YLD counts during this period (Table S3 in the **Online Supplementary Document**).

Myanmar and Peru had the highest and lowest estimated prevalence rate in 2019 (Myanmar: 8; 95% UI = 36.8, 166.8 vs Peru: 1, 95% UI = 0.1, 3.5) as well as the highest and lowest YLD rates (Myanmar: 17.9; 95% UI = 6.6, 36.9 vs Peru: 0.2; 95% UI = 0, 0.8) (**Figure 2**, panels C-D and Table S2 in the **Online Supplementary Document**).

The percentage change in prevalence and YLD rates from 1990 to 2019 differed substantially among the 204 countries and territories, and only two countries, the United States of America (2.6%; 95% UI = -4.6, 14.4) and Norway (0.6%; 95% UI = -6.7, 16.2), showed upward trends. In contrast, Equatorial Guinea (-83.7%; 95% UI = -89.7, -78.8) showed the largest decreases in prevalence and YLD rates during this period (Table S2 in the **Online Supplementary Document**).

### Age and sex patterns

In 1990 and 2019, both the prevalence and YLD counts and the corresponding rates were higher among males than among females worldwide and in the fives SDI quintiles (**Table 1**, Figures S9-S12 Table S1 in the **Online Supplementary Document**).

**Figure 3:**
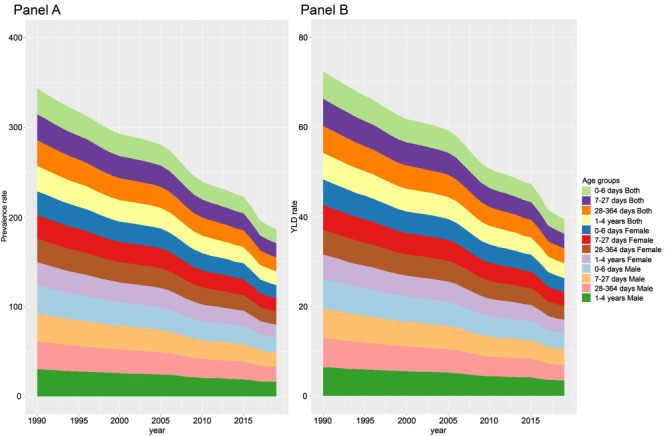
Global trends for the age-specific prevalence and YLD rates by both sexes, females, males, from 1990 to 2019. **Panel A.** Prevalence rates. **Panel B.** YLD rates. YLD – years lived with disability.

We divided children under five years into four age subgroups: 0-6 days, 7-27 days, 28-364 days, and 1-4 years according to the GBD age subgroup arrangement. In 2019, both the global count and prevalence rate and the global number and rate of YLD were higher among males than among females in all for age subgroups (**Figure 3**, Tables S5-6 and Figures S13-14 in the **Online Supplementary Document**). The global counts of prevalence and YLD followed upward trends from the 0-6-day age group to the 1-4-year age group, with similar patterns between males and females. However, the global prevalence and YLD rates followed stable trends from the 0-6-day age group to the 1-4-year age group, and was likewise similar between males and females (**Figure 3**, Table S5-6 and Figures S13-14 in the **Online Supplementary Document**). Both the global prevalence and YLD counts and the global prevalence and YLD rates followed downward trends among males and females in all four age subgroups from 1990 to 2019 (**Figure 3**, Table S5-6 and Figures S13-14 in the **Online Supplementary Document**).

### Burden of complete hearing loss by SDI

**Figure 4:**
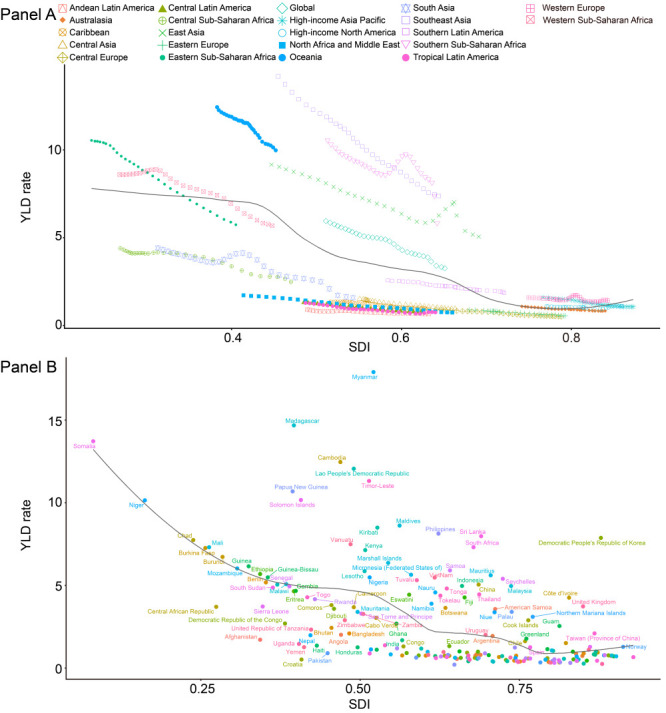
YLD rates for 21 GBD regions from 1990 to 2019 and for 204 countries and territories in 2019, for both sexes by SDI. **Panel A.** 21 GBD regions. **Panel B.** 204 countries and territories. The black line represents the expected YLD rates based solely on SDI. Locations higher than the solid black line had a higher-than-expected burden, while those below the line had a lower-than-expected burden. SDI – sociodemographic index, YLD – years lived with disability.

Both the regional burden and the national burden of complete hearing loss presented trends in the YLD rate in terms of the SDI from 1990 to 2019 (**Figure 4**, panels A-B). There were generally negative associations between the corresponding YLD rates and SDI values (ranging from 0 to 0.8), followed by slowly increasing trends of YLD rates by SDI value (ranging from 0.8 to 1.0). 

### Predictions of prevalence rates at global, regional, and national levels by sex patterns

#### Global level

The predictions of global prevalence rates for males, females and both sexes shared similar downward trends from 2019 to 2030 by the BAPC model. In 2030, the rate was projected to decrease by -58.6% (from 14.0 in 2019 to 5.8 in 2030) for males and by -59.7% (from 12.4 in 2019 to 5.0 in 2030) for females. For both sexes, the rate decreased by -59.1% (from 13.2 in 2019 to 5.4 in 2030) (Figure S28 in the **Online Supplementary Document**).

#### Regional level

**Figure 5:**
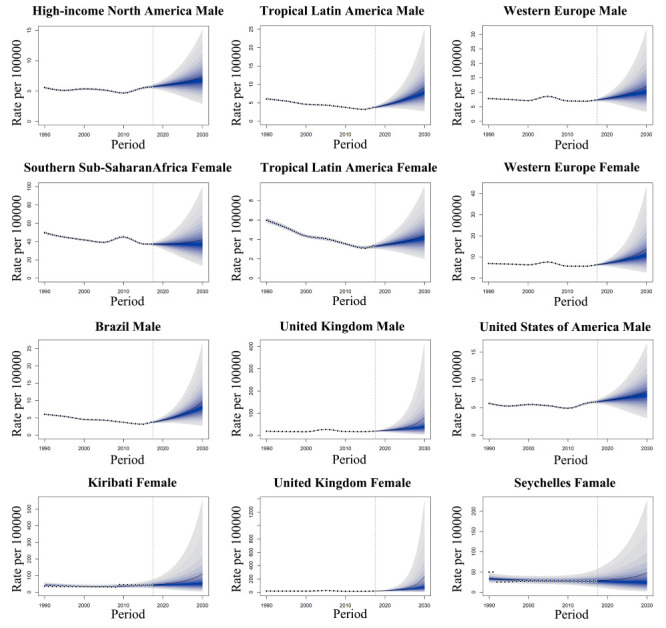
The regions and nations with top three increasing trends in prevalence rates from 2019 to 2030, by males and females.

Predicted by the BAPC model, the prevalence rates for males are predicted to increase from 2019 to 2030 in four regions, including Andean Latin America, high-income North America, Western Europe, and tropical Latin America (**Figure 5**), while 17 regions showed downward trends (Figure S29 in the **Online Supplementary Document**).

For females, the prevalence rates are predicted to decrease from 2019 to 2030 in 14 regions, while five regions, including the Caribbean, Central Sub-Saharan Africa, Southern Sub-Saharan Africa, tropical Latin America and Western Europe, showed upward trends (**Figure 5**). The prevalence rates of two regions (high-income North America and Oceania) were projected to first decrease and then increase from 2019 to 2030 (Figure S30 in the **Online Supplementary Document**). For both sexes, the prevalence rates are predicted to decrease from 2019 to 2030 in 16 regions, but increase in five regions (Caribbean, Central sub-Saharan Africa, high-income North America, Tropical Latin America and Western Europe) (Figure S31 in the **Online Supplementary Document**.

#### National level

Predicted by the BAPC model, the prevalence rates for males in 25 countries failed to be predicted due to the predicted absolute prevalence counts being lower than 1. The prevalence rates from 2019 to 2030 are predicted to increase in 29 countries (**Figure 5**) and decrease in 144 countries. The prevalence rates are predicted to decrease first and then increase in Brunei Darussalam, France, the Marshall Islands, Trinidad and Tobago, while we found a reverse trend in Peru. The Netherlands showed stable trends in the prevalence rate from 2019 to 2030 (Figure S32 in the **Online Supplementary Document**).

For females, the prevalence rates of 27 countries failed to be predicted due to the predicted absolute prevalence counts being lower than 1. The prevalence rates from 2019 to 2030 are predicted to increase in 23 (**Figure 5**) and decreased in 148 countries. The prevalence rates are predicted to decrease first and then increase in Finland, Guyana, Haiti, and the United States of America. The Netherlands and Sweden showed stable trends in the prevalence rate from 2019 to 2030 (Figure S33 in the **Online Supplementary Document**). For both sexes, we were unable to predict the prevalence rates in 21 countries due to the predicted absolute prevalence counts being lower than 1. However, our model predicted that the prevalence rates will increase from 2019 to 2030 in 20 countries and decrease in 151. The prevalence rates are predicted to first decrease then increase in Venezuala, Suriname, Islands, Haiti, Guyana, Democratic Republic of Congo, and Bolivia, while Greece, Israel, Netherlands, Peru, and Sweden are predicted to have stable rates (Figure S34 in the **Online Supplementary Document**.

Notably, the prevalence rates showed contrasting trends between males and females in 12 countries, including Bolivia, Cyprus, Ecuador, Greece, Ireland, Israel, Italy, Malta, Pakistan, the Republic of Korea, Tonga and Venezuela, which presented upward trends for males and downward trends for females from 2019 to 2030. The prevalence rates in five countries, including the Democratic Republic of the Congo, Estonia, North Macedonia, Slovenia and South Africa, presented downward trends for males and upward trends for females from 2019 to 2030.

We used the ARIMA model in our study to predict the prevalence rate of complete hearing loss caused by congenital birth defects in children younger than five years from 2019 to 2030 for males, females, and both sexes. Overall, the prevalence rates predicted by the ARIMA model were generally consistent with those predicted by the BAPC model at the global, regional, and national levels (Figures S35-S41 in the **Online Supplementary Document**).

## DISCUSSION

To the best of our knowledge, this is the first study to describe the prevalence and YLD of congenital complete hearing loss among children younger than five years from 1990 to 2019. We found an an overall decrease in the global prevalence and YLD between 1990 and 2019 and predicted ongoing downward trends from 2019 to 2030. This change is in contrast with the trends in the number of people with moderate-to-complete hearing loss, which increased between 1990 and 2019 [8], probably related to worldwide efforts in treatment for congenital infections and reduced usage of ototoxic medications during pregnancy, significantly reducing the risk of congenital hearing loss [19,20].

We found that the global prevalence and YLD counts of congenital complete hearing loss showed upward trends from the 0-6-day age group to the 1-4-year age group. In countries with universal hearing screening, the estimated prevalence of perpetual bilateral hearing loss were 13.3 per 10 000 neonates [21], with a further increase to 28.3 per 10 000 children aged 0-5 years [22]. This increase over time may reflect a cumulative increase in patients with hearing loss due to progressive or late-onset genetic causes [19]. We found that the congenital complete hearing loss burden increased over time, suggesting that neonatal hearing screening may fail to identify children with ongoing hearing loss; therefore, repeated screenings at frequent intervals are recommended, especially for high-risk infants.

Consistent with previous findings on sex-related variations in hearing loss [23,24], we found a higher congenital complete hearing loss burden among males than among females. The sex disparity in hearing may be attributed to the variation in sex hormones, with estrogen potentially protecting the auditory system [25]. Compared to females, males are projected to have a higher prevalence rate of congenital complete hearing loss from 2019 to 2030, which indicates that additional research is urgently needed to help facilitate the development of sex-specific interventions, guidelines, or therapies. Compared to the 1-4-year age group, there was a significant decrease in the prevalence of congenital hearing loss in the 0-6-day age group during the study period. This discrepancy may be attributed to the progressive nature of autosomal dominant nonsyndromic hearing loss, which accounts for 20% of genetic hearing loss [1]. The progressive nature of this condition implies that certain genetic changes may manifest at different ages, leading to variations in the prevalence between different age groups. Moreover, first-line genetic tests usually only screen for mutations in GJB2 and GJB6 [2], which highlights the urgent need for comprehensive genetic testing.

Prevalence and YLD rates decreased in most regions among males and females from 1990 to 2019, except for high-income North America, which might be due to four North American epidemics caused by Toxoplasma gondii infection [26] and periodic resurgences of syphilis every 10-15 years [27]. Although the prevalence rates are forecasted to decrease in most regions from 2019 to 2030, upward trends are projected in the Caribbean, Central Sub-Saharan Africa, Western Europe, tropical Latin America and high-income North America. These results indicate that low SDI regions should improve primary care by screening and raising awareness of hearing care, which might be cost-effective, while improving genetic diagnoses is necessary for high SDI regions to moderate the effects of genetic hearing loss.

Nationally, Myanmar, Madagascar, and Somalia had the highest burden of congenital hearing loss among developing countries due in part to inadequate immunisation, increased exposure to ototoxic agents, and consanguinity [28]. In most developing countries, prevention measures and access to health care services are not considered high-priority needs, as limited resources often require difficult choices. Although the prevalence of congenital hearing loss decreased globally between 1990 and 2019, we found a significant increase in the burden of complete congenital hearing loss in a few developed countries. Previous studies revealed that genetic causes account for up to 80% of congenital hearing loss in developed countries, while environmental or acquired causes make up the remaining 20% [29]. The reason for the increased burden in a few developed countries is in part related to inherited hearing loss, such as mutations in the GJB2 gene [30]. Although the prevalence rates in most countries are predicted to decrease from 2019 to 2030, they are predicted to increase in more than 20 countries. Given that most countries with increased predicted prevalence rates have a low socioeconomic status, better prevention of infectious etiologies, such as vaccination programs, is urgently needed.

Our study has several limitations, such as the sparsity of data from low-income countries and insufficient data on the causes of congenital hearing loss, such as mutations in GJB2, congenital cytomegalovirus infection, and congenital rubella infection. Additionally, while we used the SDI to describe socioeconomic differences among countries, further classification could provide further information for understanding congenital hearing loss, such as ethnicity.

## CONCLUSIONS

We found that the burden of congenital complete hearing loss in children under five years varied widely across locations, sexes, and age groups from 1900 to 2030. Cost-effective, preventive treatment strategies, including newborn hearing screening, enhanced prenatal care, improved sanitation, immunisation, and reduction of exposure to ototoxic drugs, are needed to alleviate the adverse effects of congenital hearing loss.

## Additional material


Online Supplementary Document
