# Supplementary material for: Downward trends in the global burden of congenital complete hearing loss in children younger than five years from 1990 to 2030
Source: J Glob Health. 2023 Oct 13;13:04120. doi: 10.7189/jogh.13.04120 (PMC10569368; doi:10.7189/jogh.13.04120)
Supplement: Online Supplementary Document [file jogh-13-04120-s001.pdf]

Supplementary materials

Table of contents

Supplementary figures: 3
Figure S1 3
Figure S2 4
Figure S3 5
Figure S4 6
Figure S5 7
Figure S6 8
Figure S7 9
Figure S8 10
Figure S9 11
Figure S10 12
Figure S11 13
Figure S12 14
Figure S13 15
Figure S14 16
Figure S15 17
Figure S16 18
Figure S17 19
Figure S18 20
Figure S19 21
Figure S20 22
Figure S21 23
Figure S22 24
Figure S23 25
Figure S24 26
Figure S25 27
Figure S26 28
Figure S27 29
Figure S28 30
Figure S29 31
Figure S30 33
Figure S31 35
Figure S32 37
Figure S33 49
Figure S34 61
Figure S35 72
Figure S36 73
Figure S37 75
Figure S38 77
Figure S39 79
Figure S40 93

Figure S41 107
Supplementary tables: 121
Table 1 121
Table 2 124
Table 3 135
Table 4 144
Table 5 145
Table 6 147
Supplementary method: 149
SDI analysis 149

Supplementary figures:

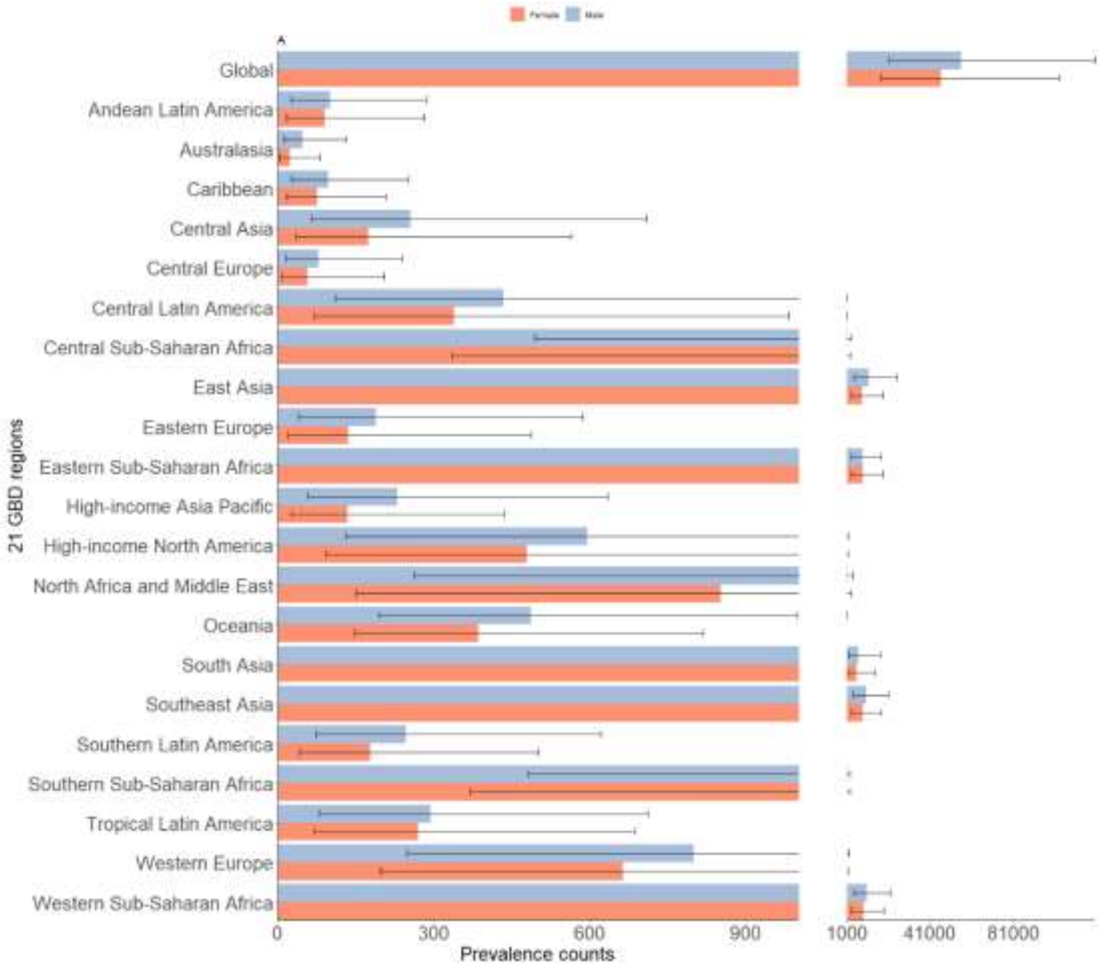

Figure S1. Prevalence number of complete hearing loss caused by congenital birth defects in children younger than 5 years for 21 GBD regions by sex, 2019.

Error bars indicate the 95% uncertainty intervals (95% UI).

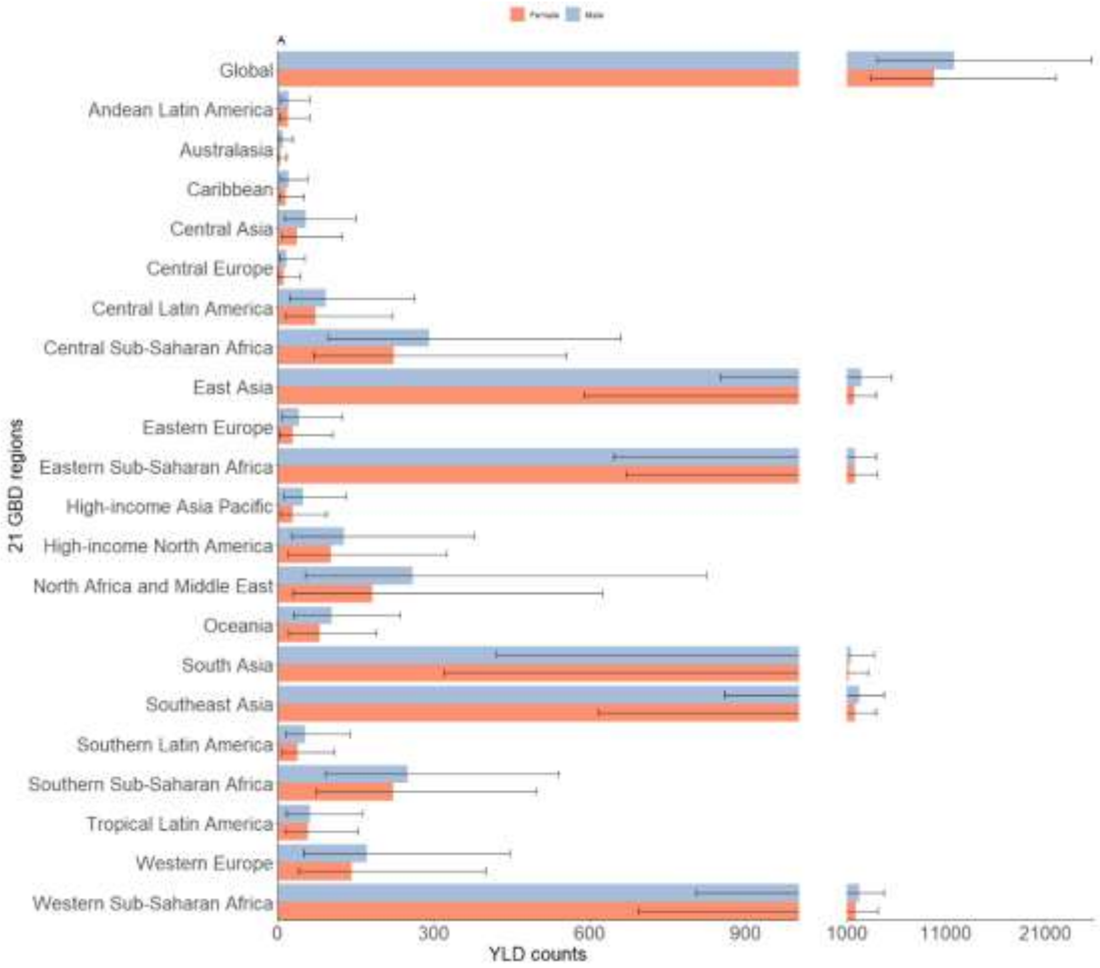

Figure S2. YLD number of complete hearing loss caused by congenital birth defects in children younger than 5 years for 21 GBD regions by sex, 2019.

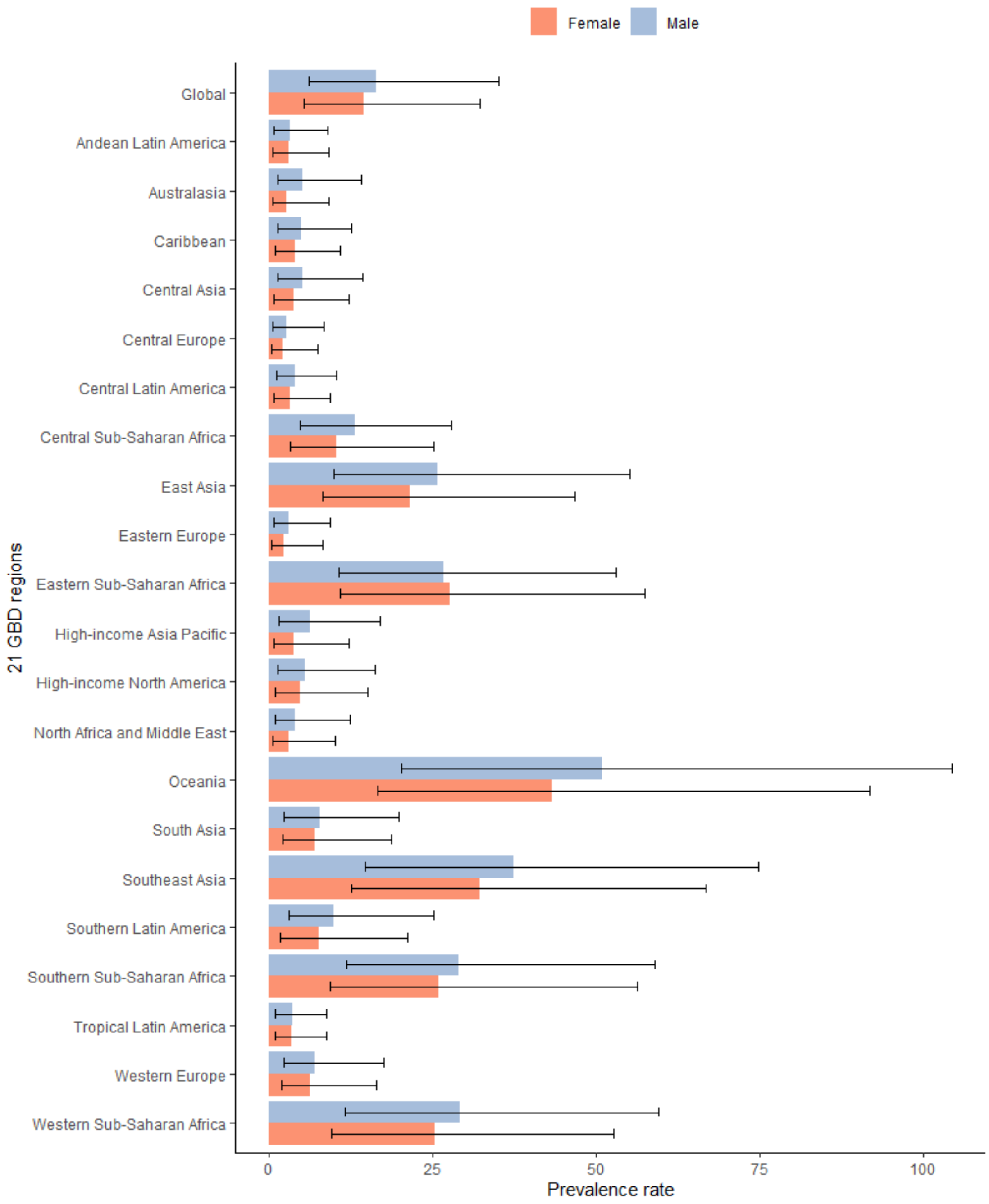

Figure S3. Prevalence rate of complete hearing loss caused by congenital birth defects in children younger than 5 years for 21 GBD regions by sex, 2019.

Error bars indicate the 95% uncertainty intervals (95% UI).

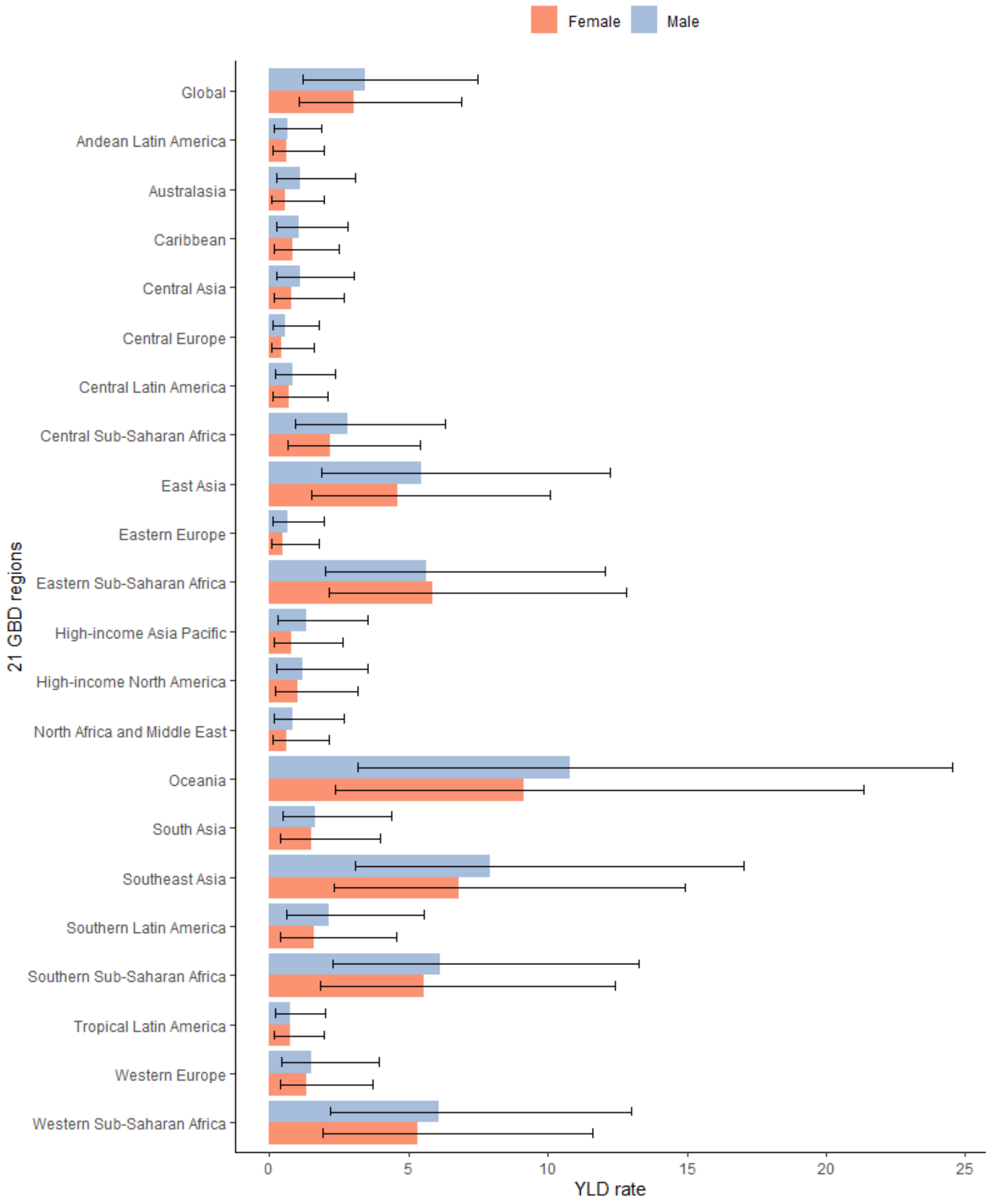

Figure S4. YLD rate of complete hearing loss caused by congenital birth defects in children younger than 5 years for 21 GBD regions by sex, 2019.

Error bars indicate the 95% uncertainty intervals (95% UI).

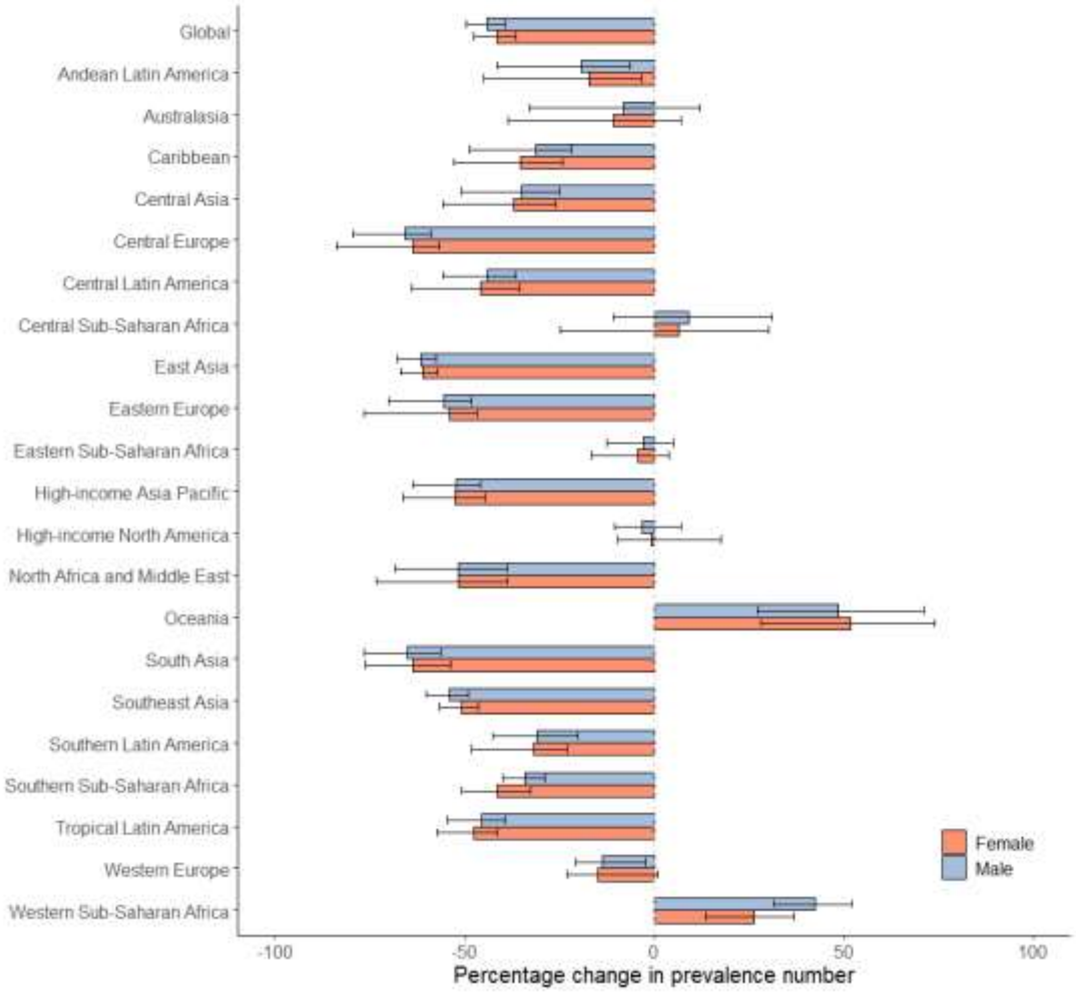

Figure S5. The percentage change in prevalence numbers of complete hearing loss caused by congenital birth defects in children younger than 5 years per 100,000 by sex for 21 Global Burden of Disease regions, 1990-2019.

Error bars indicate the 95% uncertainty intervals (95% UI).

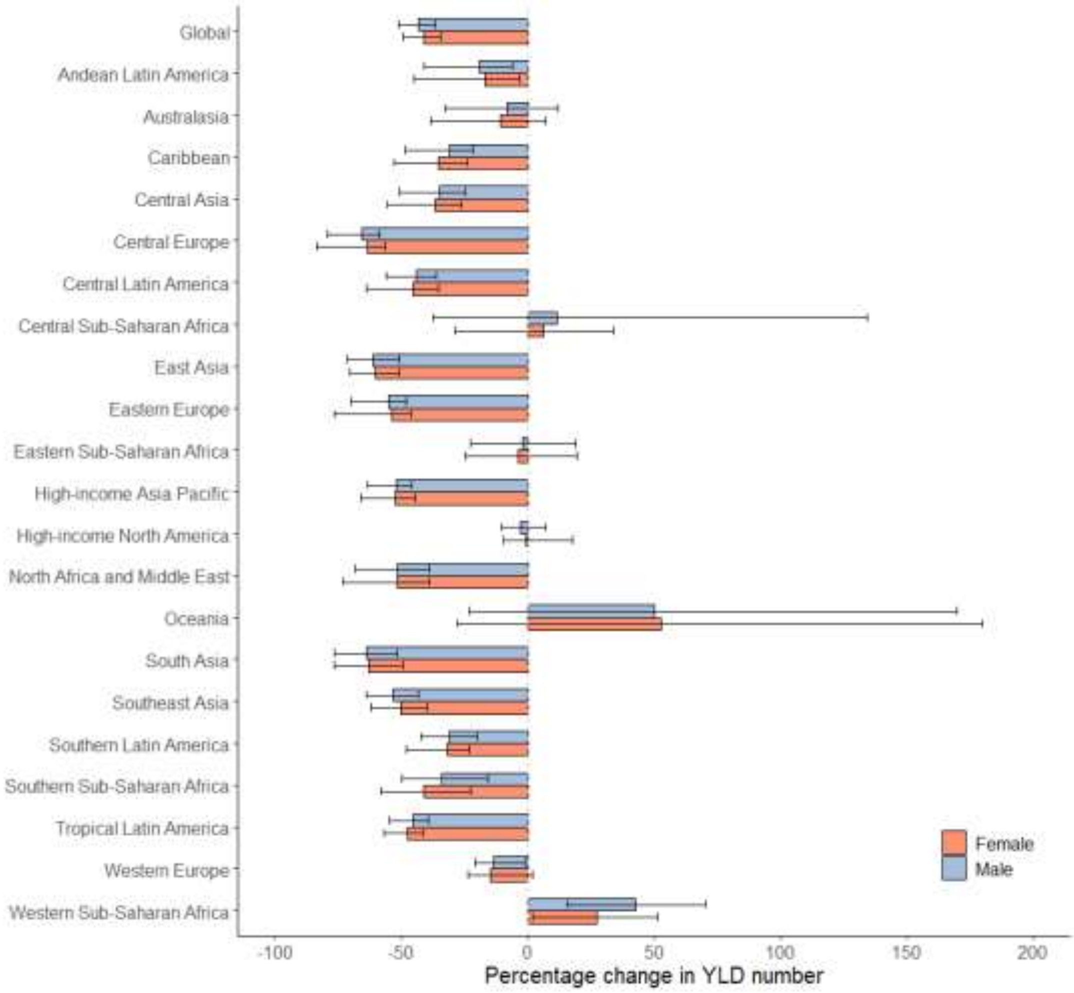

Figure S6. The percentage change in YLD numbers of complete hearing loss caused by congenital birth defects in children younger than 5 years per 100,000 by sex for 21 Global Burden of Disease regions, 1990-2019.

Error bars indicate the 95% uncertainty intervals (95% UI). YLDs: years lived with disability.

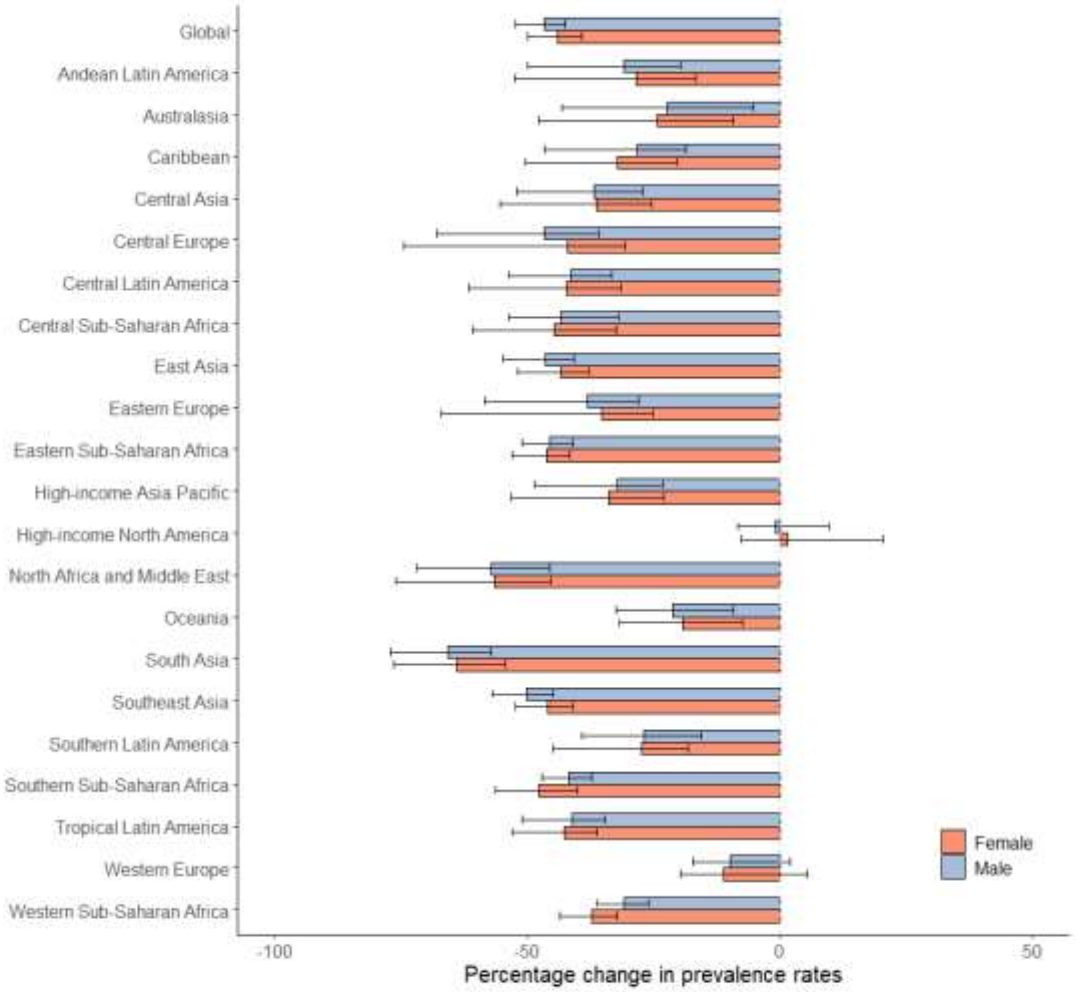

Figure S7. The percentage change in prevalence rates of complete hearing loss caused by congenital birth defects in children younger than 5 years per 100,000 by sex for 21 Global Burden of Disease regions, 1990-2019.

Error bars indicate the 95% uncertainty intervals (95% UI).

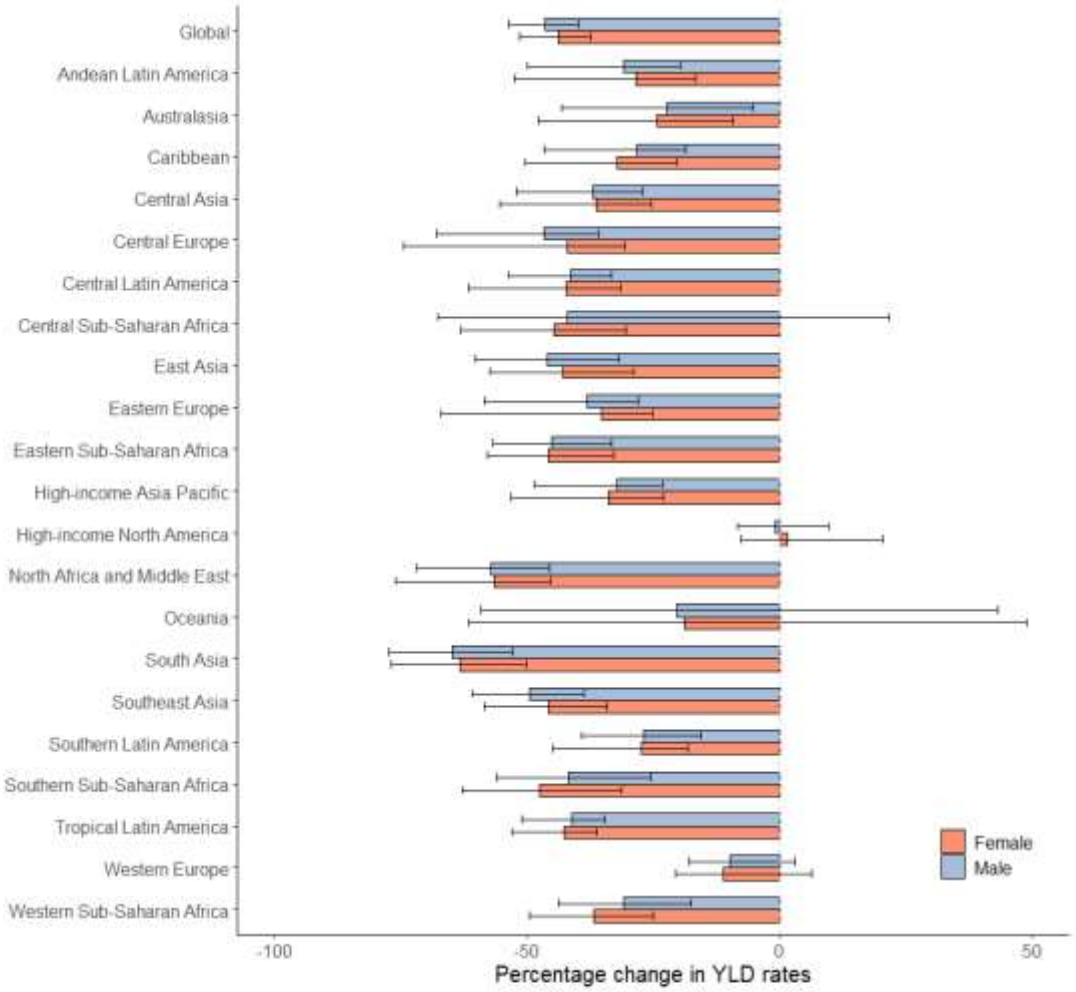

Figure S8. The percentage change in YLD rates of complete hearing loss caused by congenital birth defects in children younger than 5 years per 100,000 by sex for 21 Global Burden of Disease regions, 1990-2019.

Error bars indicate the 95% uncertainty intervals (95% UI).

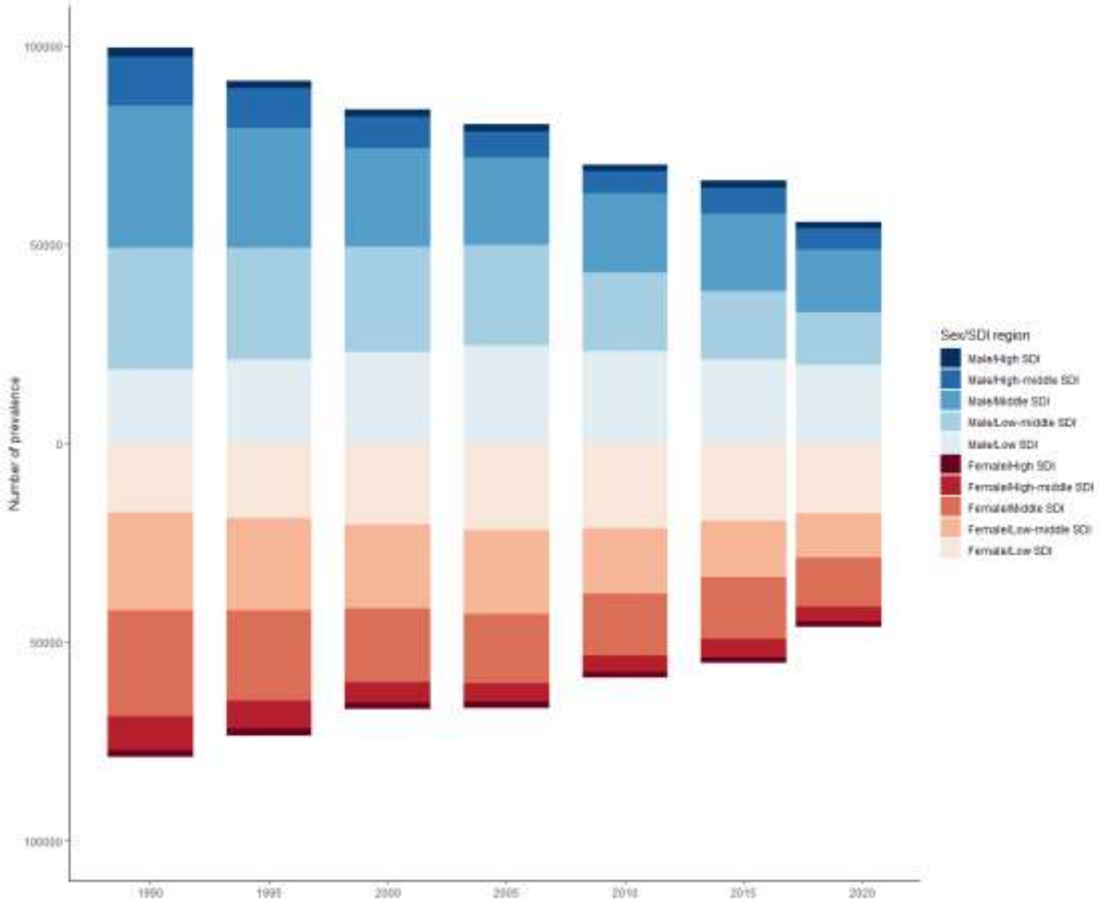

Figure S9. Temporal trends in counts of prevalence 5 SDI regions from 1990 to 2019, by sex.

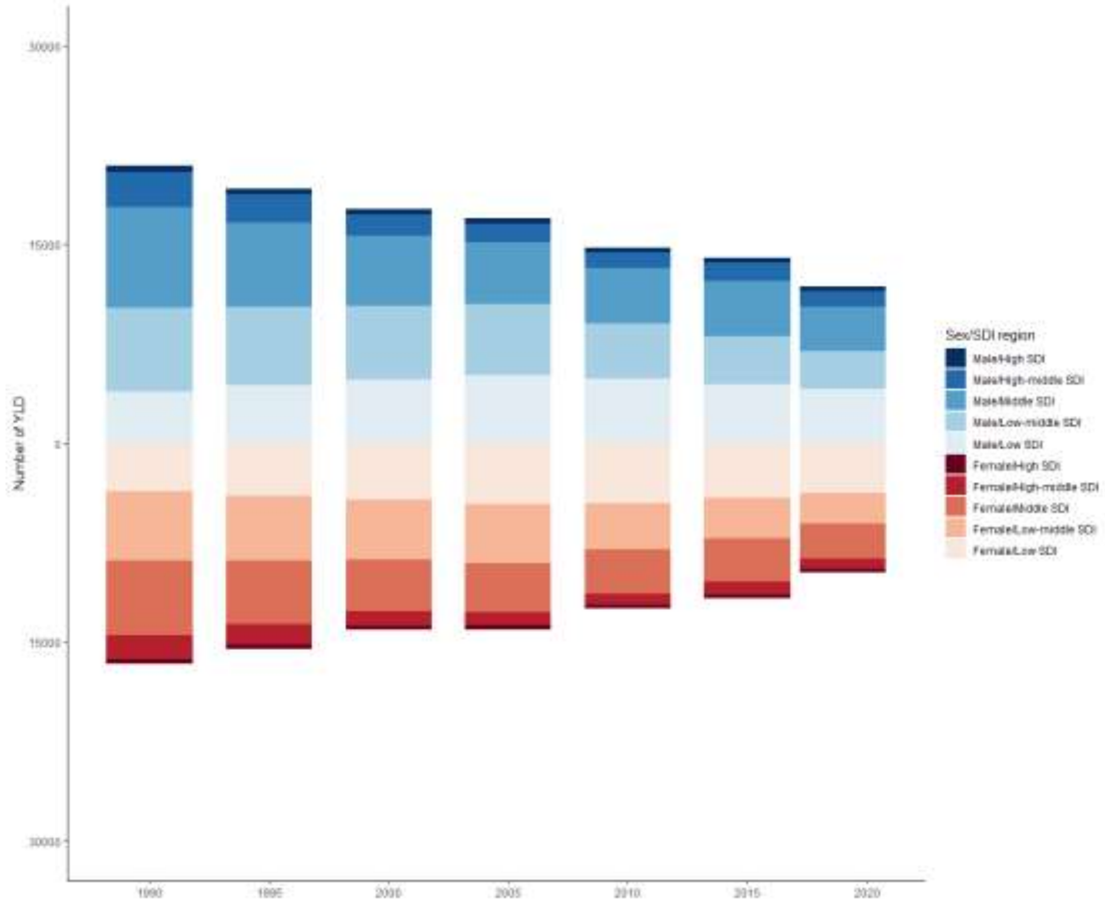

Figure S10. Temporal trends in counts of YLD in 5 SDI regions from 1990 to 2019, by sex.

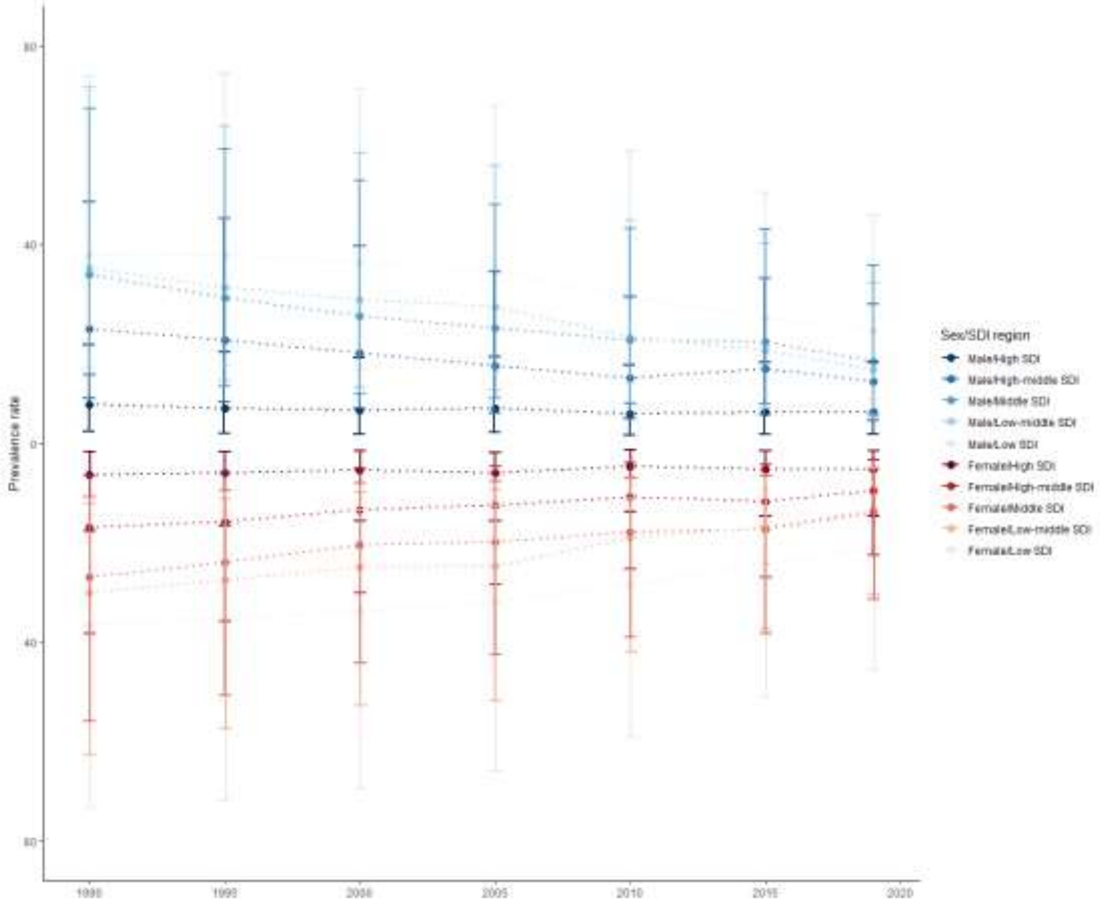

Figure S11. Temporal trends in rates of prevalence in 5 SDI regions from 1990 to 2019, by sex.

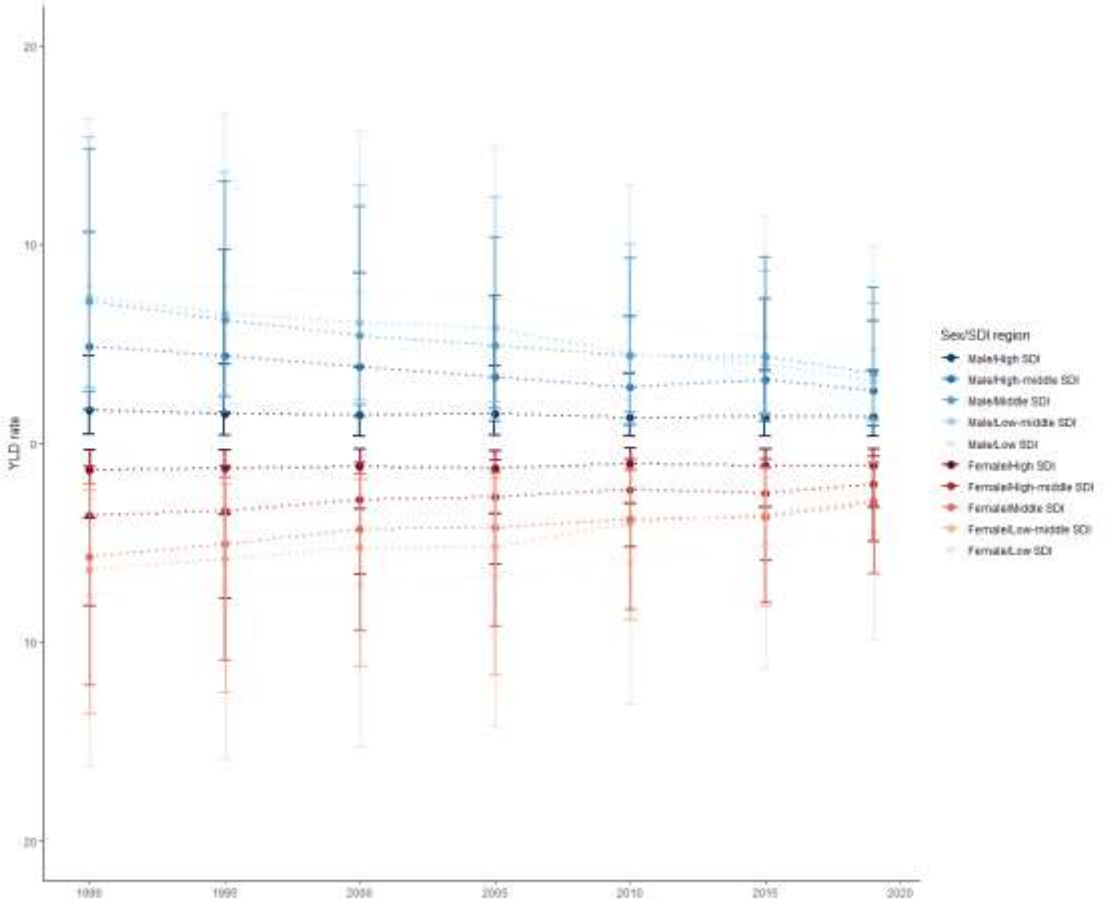

Figure S12. Temporal trends in rates of YLD in 5 SDI regions from 1990 to 2019, by sex.

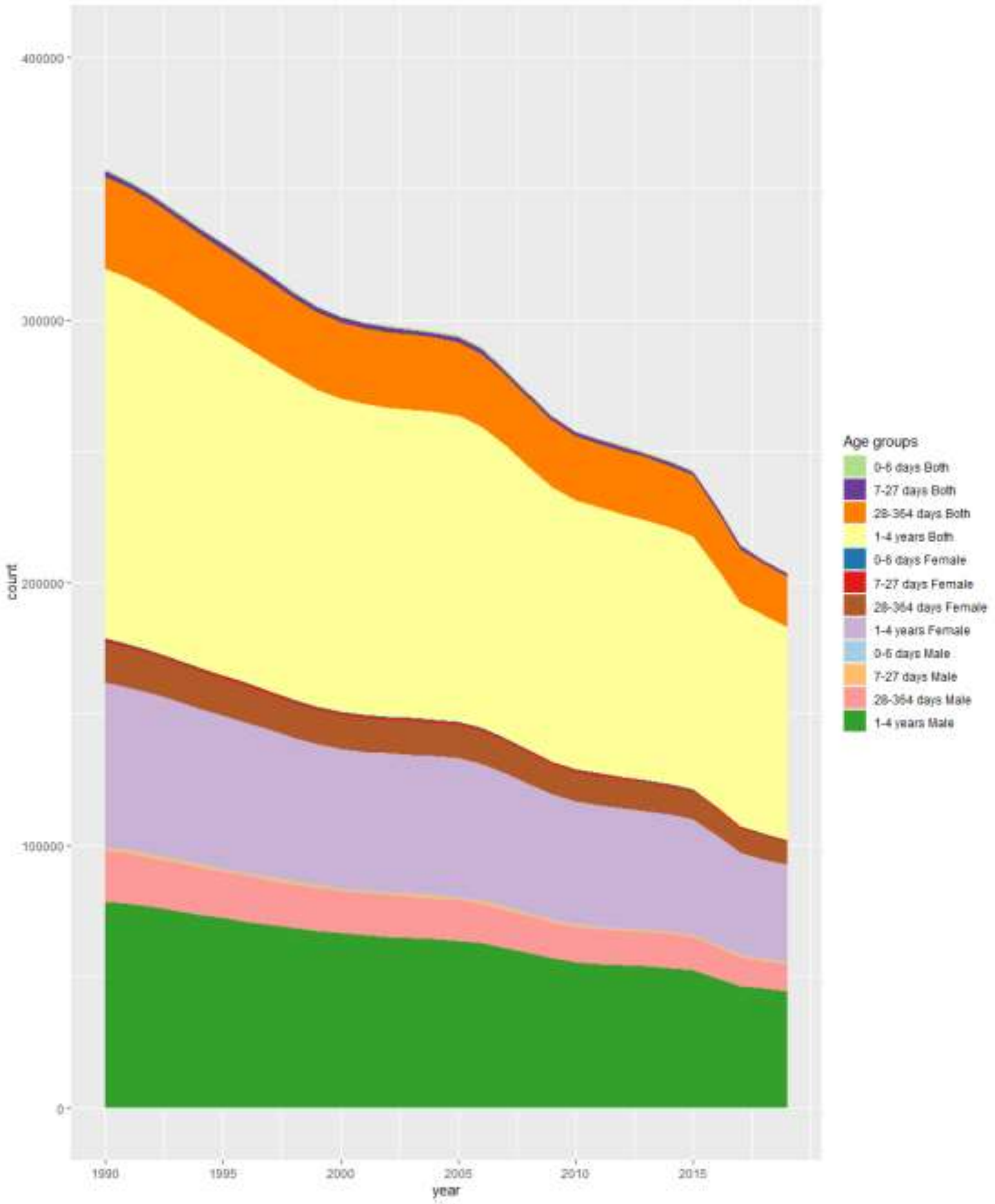

Figure S13. Global trends for the age-specific prevalence number of complete hearing loss by both sexes, female, male, from 1990 to 2019.

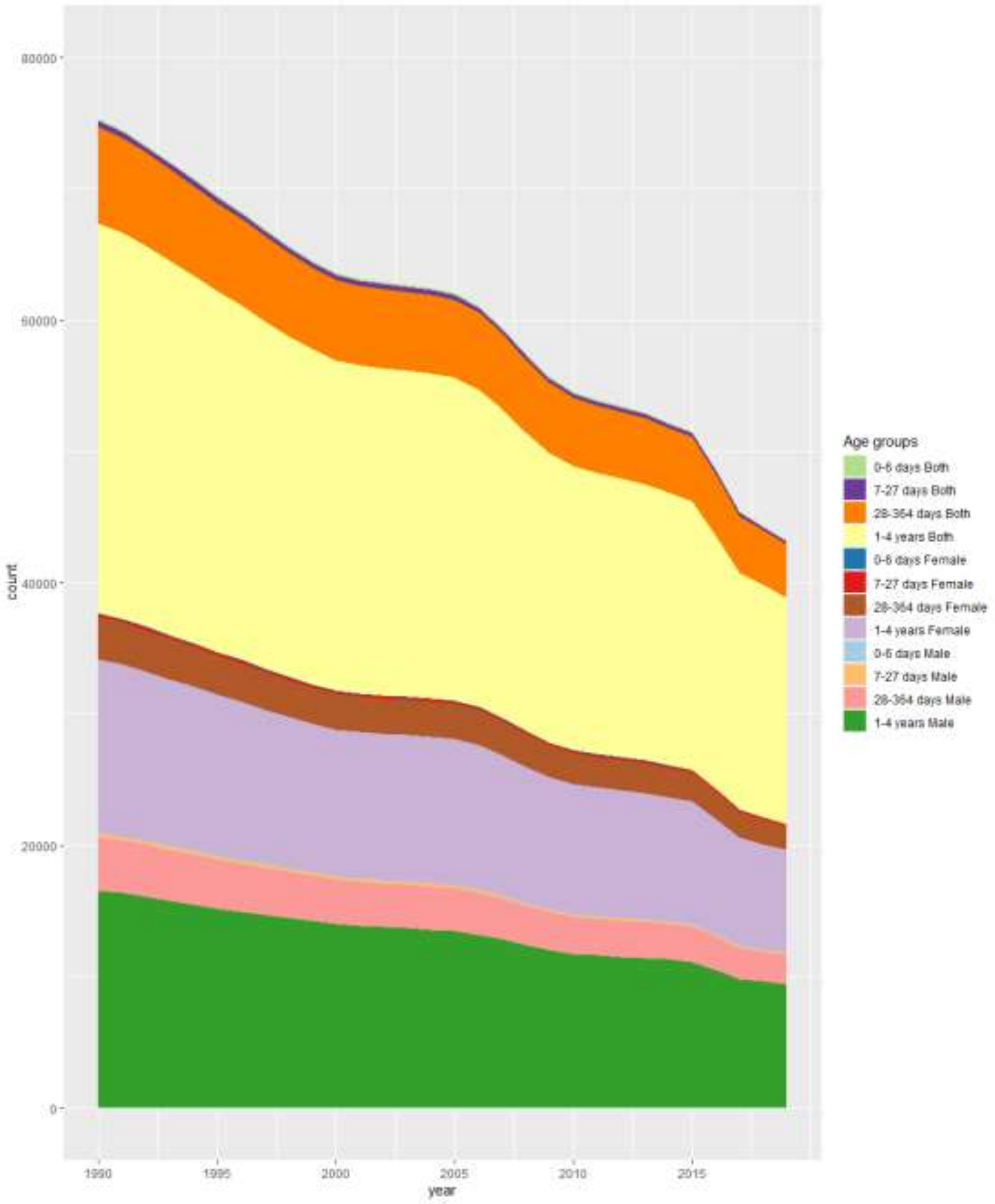

Figure S14. Global trends for the age-specific YLD number of complete hearing loss by both sexes, female, male, from 1990 to 2019.

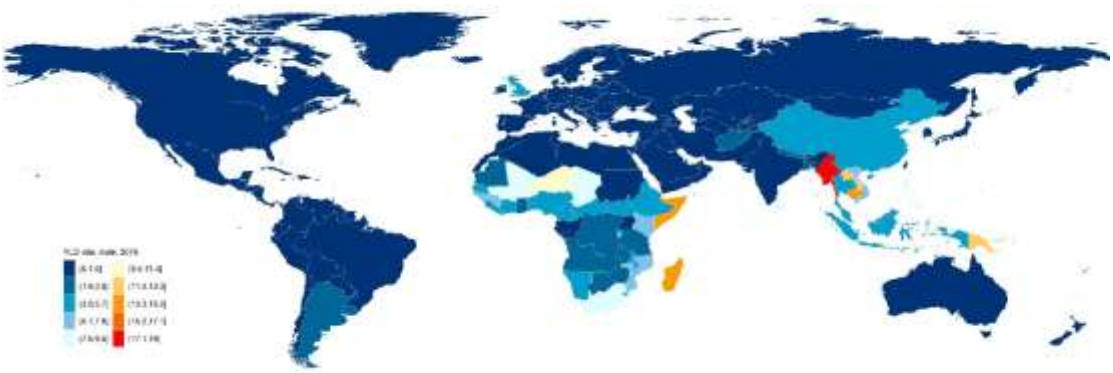

Figure S15. YLD rate per 100,000 of complete hearing loss caused by congenital birth defects younger than 5 years for male in 2019 in 204 countries.

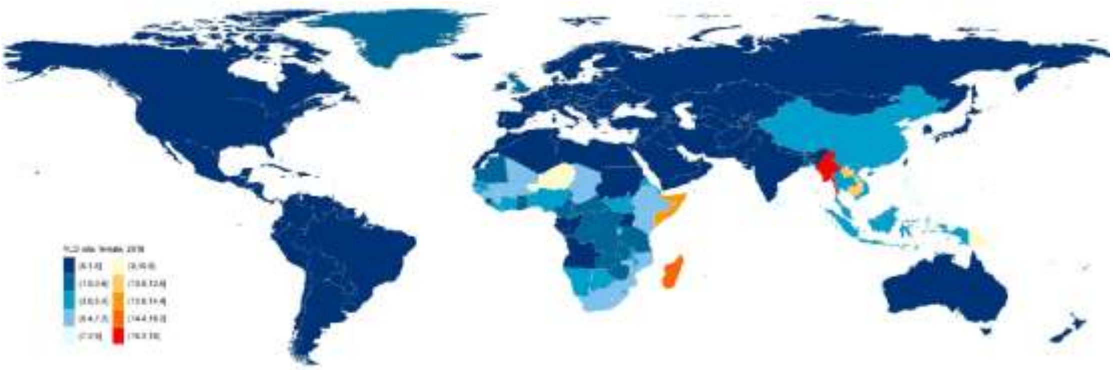

Figure S16. YLD rate per 100,000 of complete hearing loss caused by congenital birth defects younger than 5 years for female in 2019 in 204 countries.

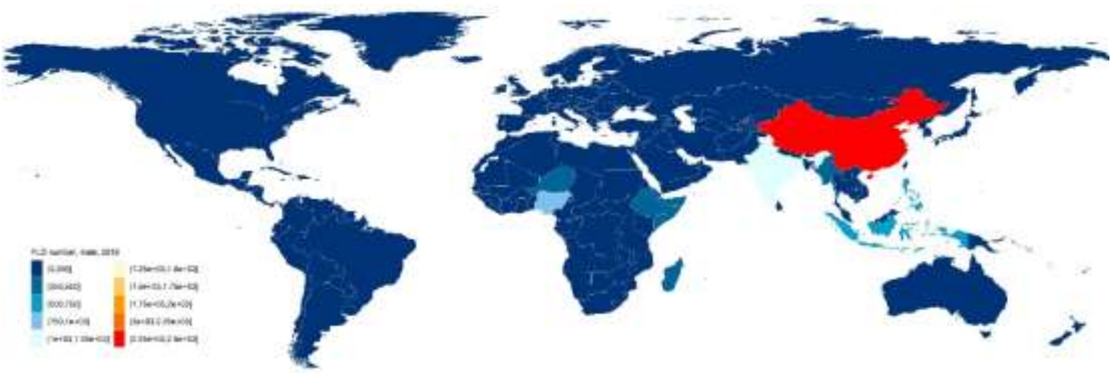

Figure S17. YLD number of complete hearing loss caused by congenital birth defects younger than 5 years for male in 2019 in 204 countries.

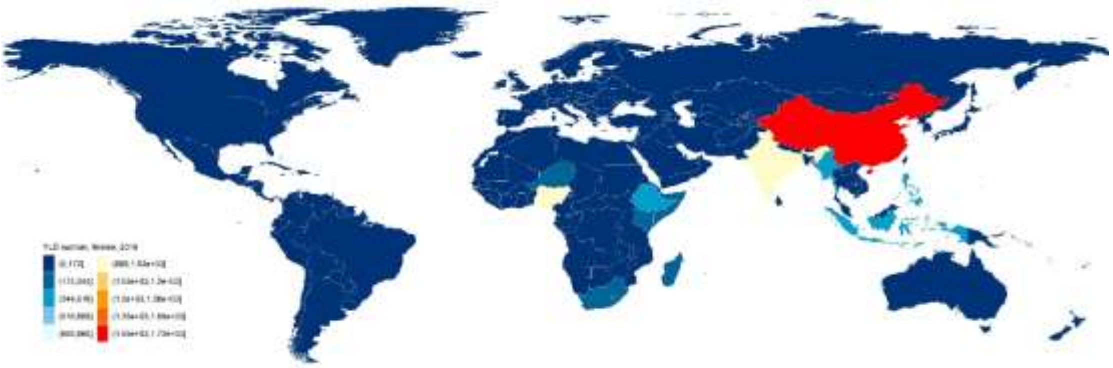

Figure S18. YLD number of complete hearing loss caused by congenital birth defects younger than 5 years for female in 2019 in 204 countries.

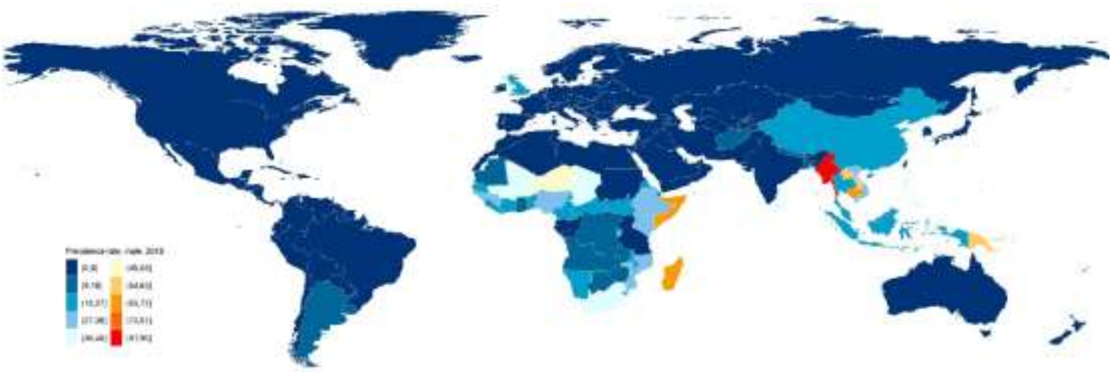

Figure S19. Prevalence rate per 100,000 of complete hearing loss caused by congenital birth defects younger than 5 years for male in 2019 in 204 countries.

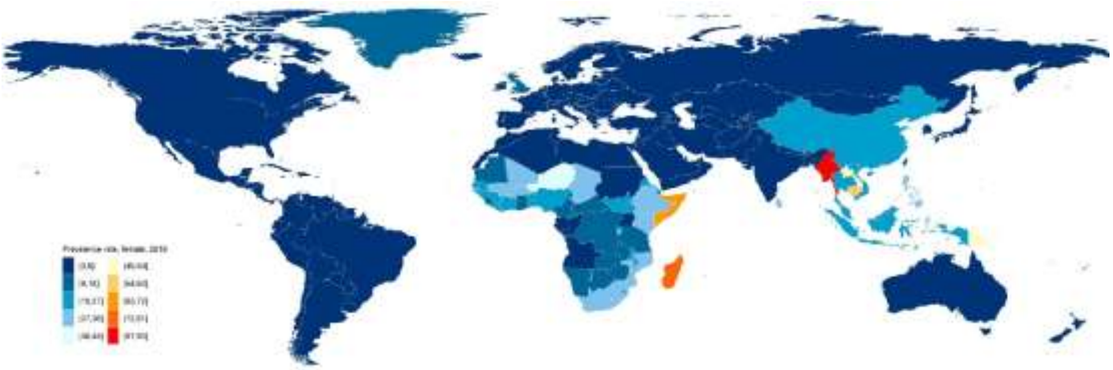

Figure S20. Prevalence rate per 100,000 of complete hearing loss caused by congenital birth defects younger than 5 years for female in 2019 in 204 countries.

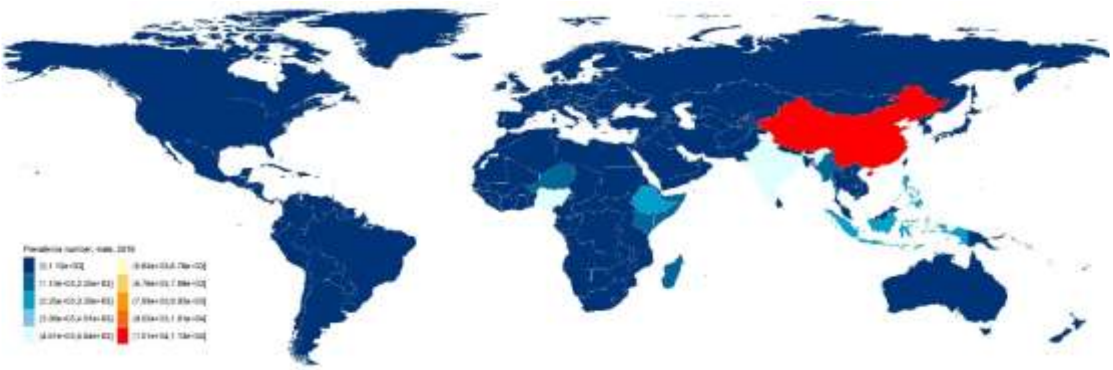

Figure S21. Prevalence number of complete hearing loss caused by congenital birth defects younger than 5 years for male in 2019 in 204 countries.

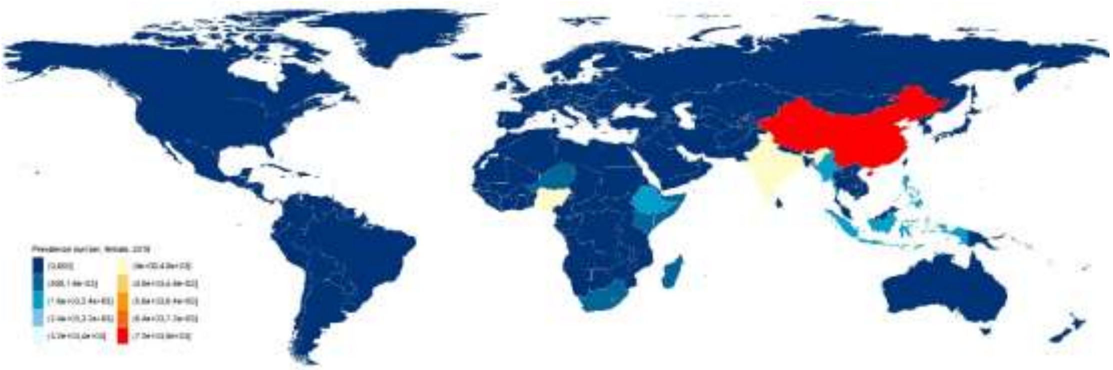

Figure S22. Prevalence number of complete hearing loss caused by congenital birth defects younger than 5 years for female in 2019 in 204 countries.

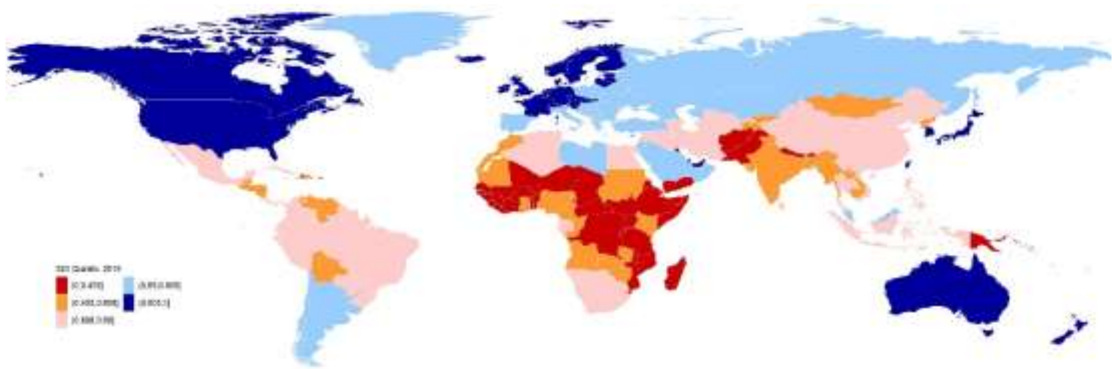

Figure S23. The 204 countries and territories distribution by SDI quintile, 2019.

Abbreviations: SDI, Socio-Demographic Index.

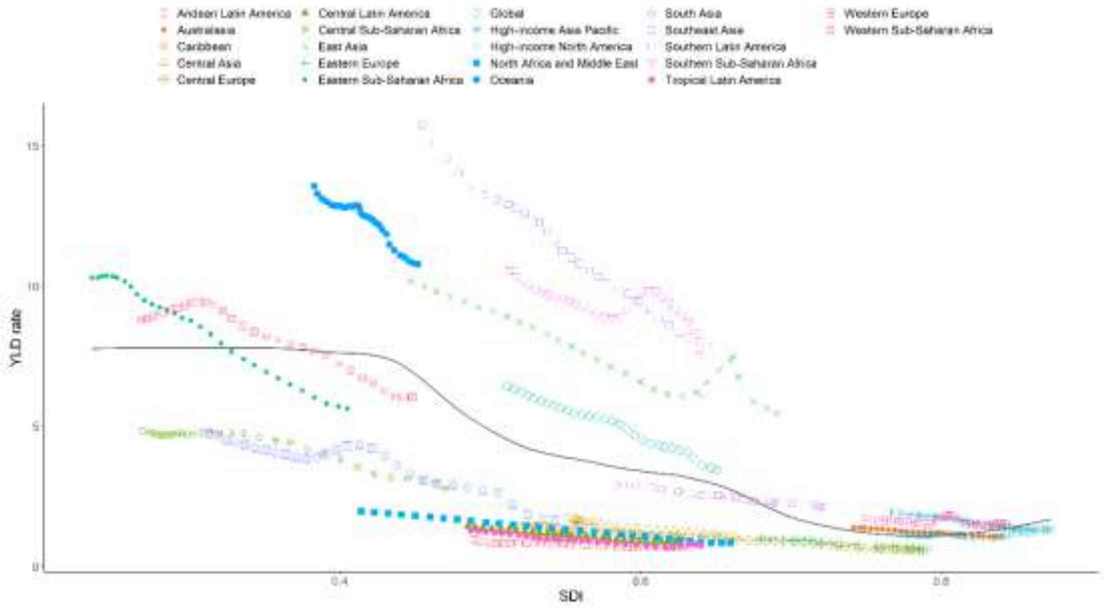

Figure S24. YLD rate per 100,000 for complete hearing loss caused by congenital birth defects in children younger than 5 years for 21 GBD regions from 1990 to 2019, for male by SDI.

The black line represents the expected YLD rates based solely on SDI. SDI: sociodemographic index; YLD: years lived with disability.

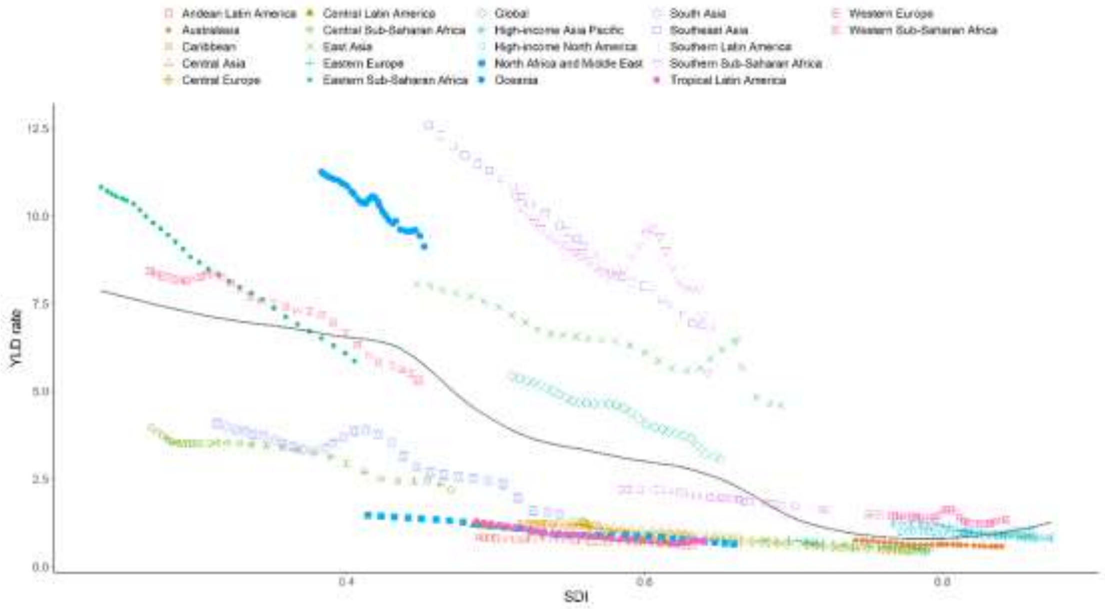

Figure S25. YLD rate per 100,000 for complete hearing loss caused by congenital birth defects in children younger than 5 years for 21 GBD regions from 1990 to 2019, for female by SDI.

The black line represents the expected YLD rates based solely on SDI. SDI: sociodemographic index; YLD: years lived with disability.

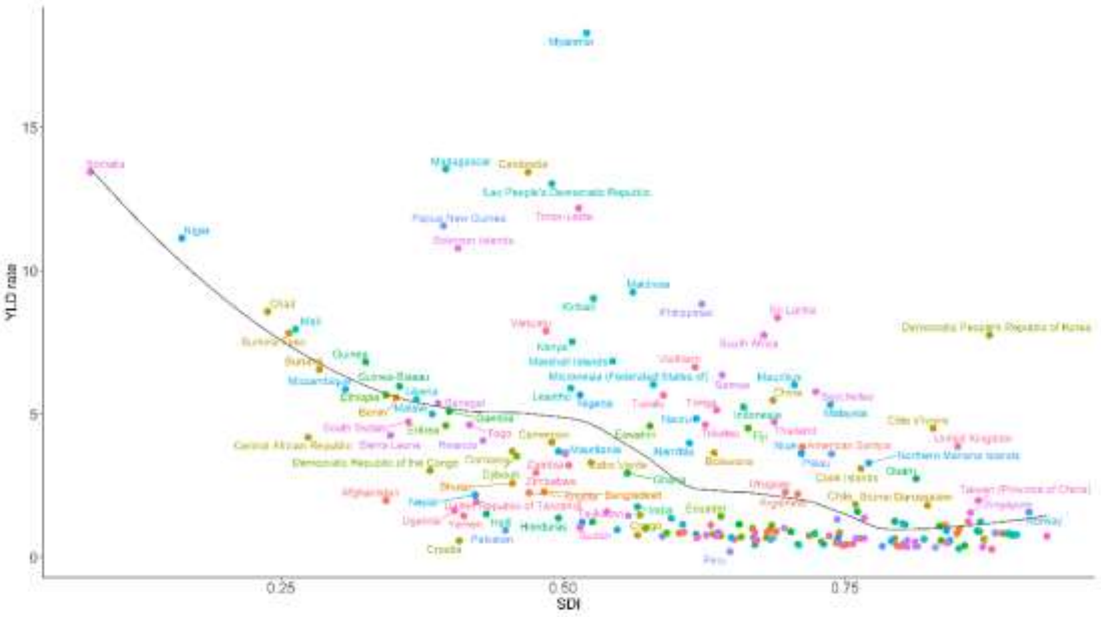

Figure S26. YLD rates for complete hearing loss caused by congenital birth defects in children younger than 5 years for 204 countries and territories in 2019, for male by SDI.

The black line represents the expected YLD rates based solely on SDI. SDI: sociodemographic index; YLD: years lived with disability.

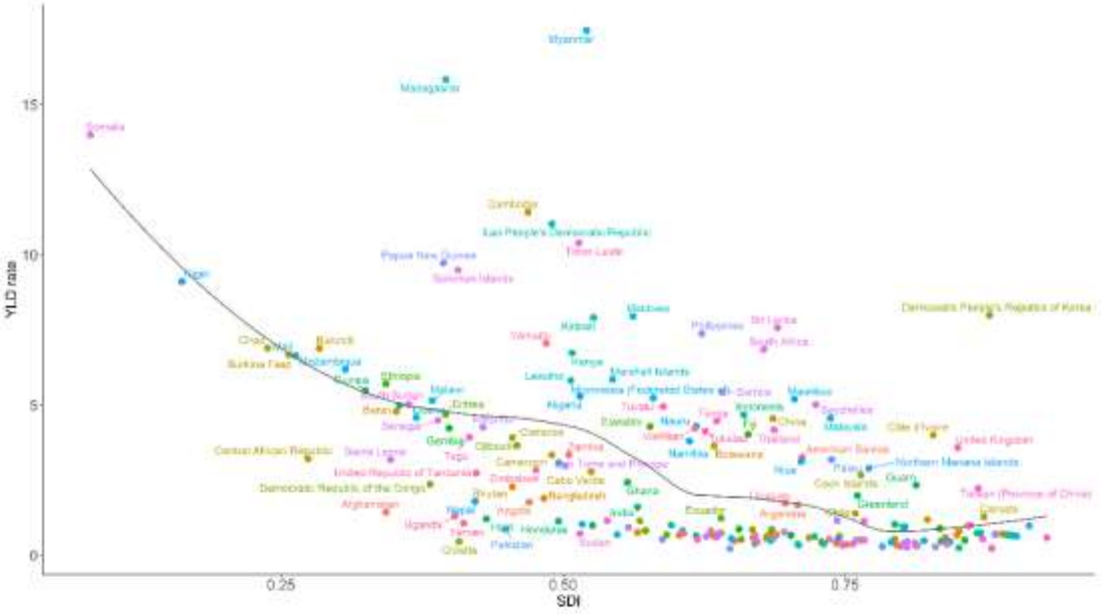

Figure S27. YLD rates for complete hearing loss caused by congenital birth defects in children younger than 5 years for 204 countries and territories in 2019, for female by SDI.

The black line represents the expected YLD rates based solely on SDI. SDI: sociodemographic index; YLD: years lived with disability. Error bars indicate the 95% uncertainty intervals (95% UI)**.**

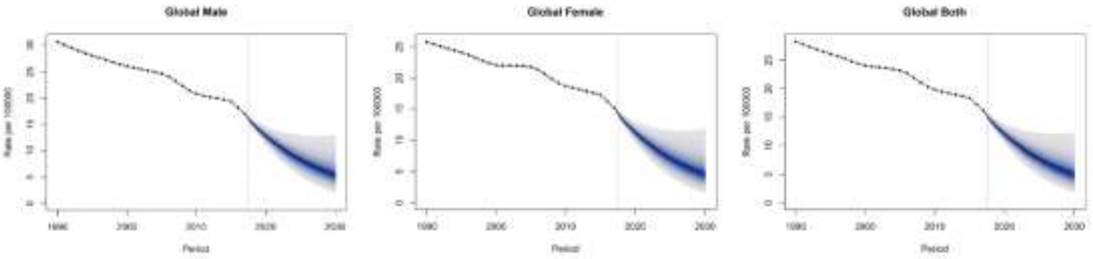

Figure S28. Global trends in prevalence rates from 1990 to 2030 for males, females and both sexes by BAPC model.

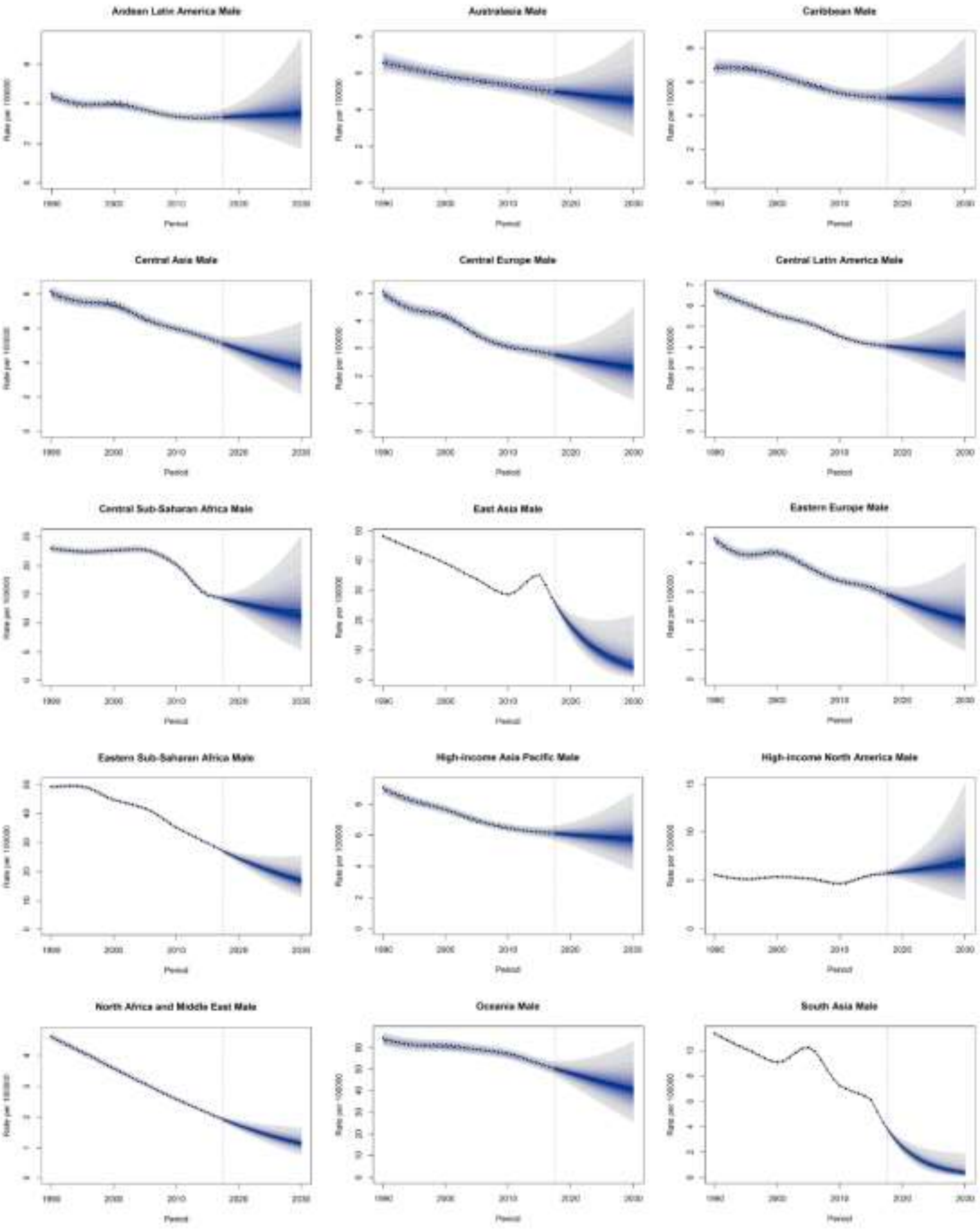
Andean Latin America Male
Australasia Male
Caribbean Male
Central Asia Male
Central Europe Male
Central Latin America Male
Central Sub-Saharan Africa Male
East Asia Male
Eastern Europe Male
Eastern Sub-Saharan Africa Male
High-income Asia Pacific Male
High-income North America Male
North Africa and Middle East Male
Oceania Male
South Asia Male
Rate per 100000
Period

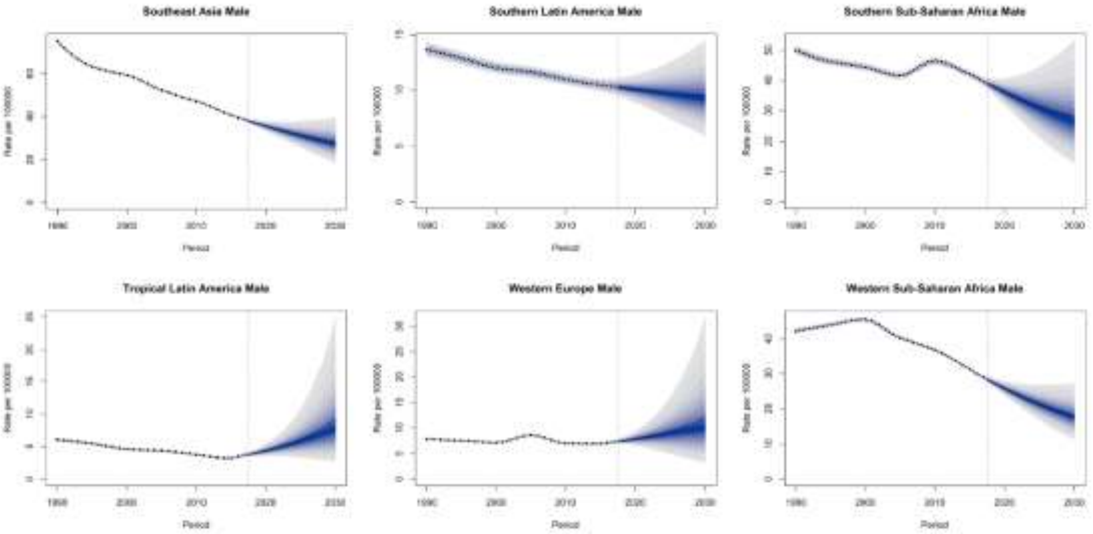

Figure S29. Trends in prevalence rates of 21 GBD regions from 1990 to 2030 for males by BAPC model.

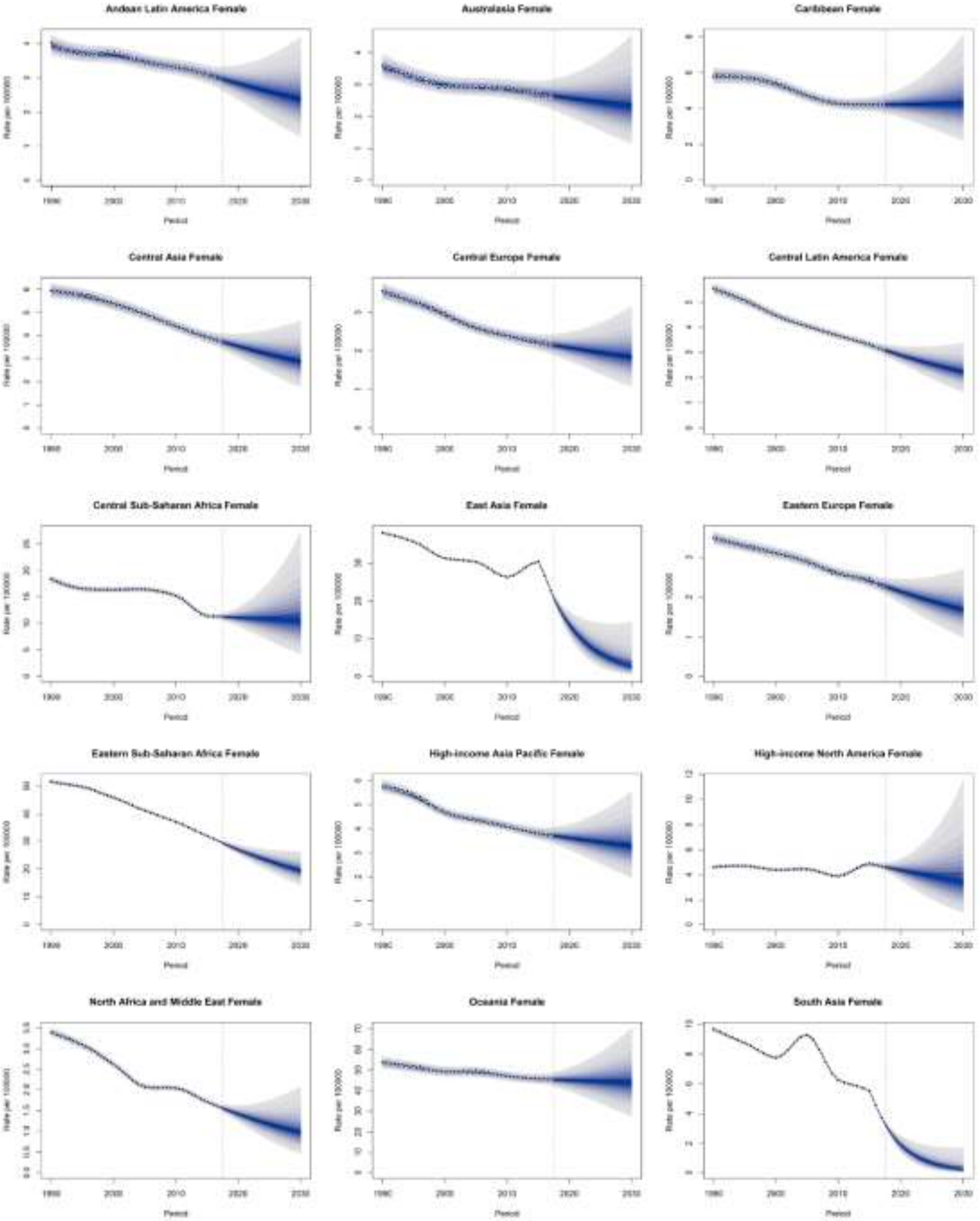
Andean Latin America Female
Australasia Female
Caribbean Female
Central Asia Female
Central Europe Female
Central Latin America Female
Central Sub-Saharan Africa Female
East Asia Female
Eastern Europe Female
Eastern Sub-Saharan Africa Female
High-income Asia Pacific Female
High-income North America Female
North Africa and Middle East Female
Oceania Female
South Asia Female

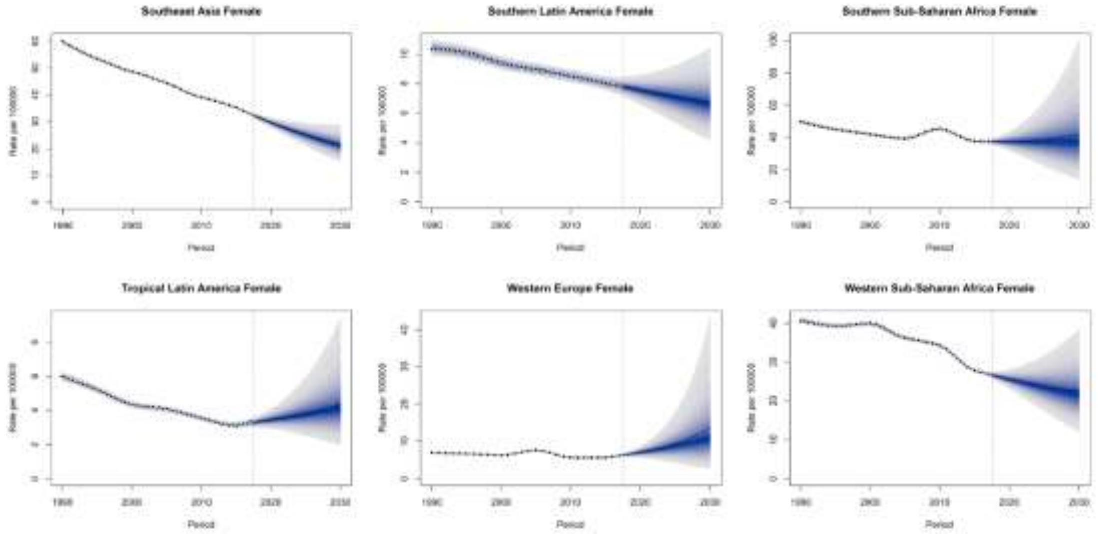

Figure S30. Trends in prevalence rates of 21 GBD regions from 1990 to 2030 for females by BAPC model.

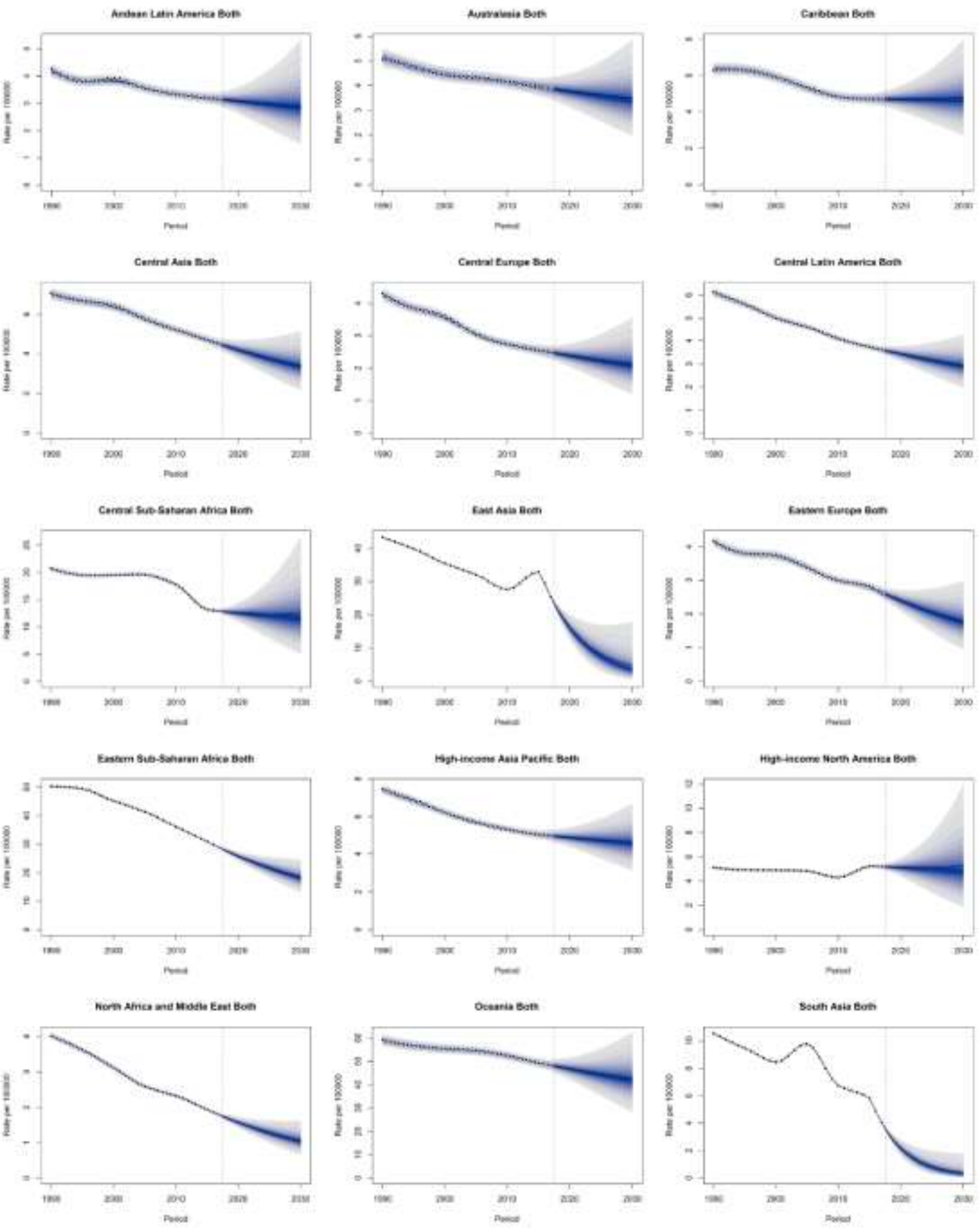
Andean Latin America Both
Australasia Both
Caribbean Both
Central Asia Both
Central Europe Both
Central Latin America Both
Central Sub-Saharan Africa Both
East Asia Both
Eastern Europe Both
Eastern Sub-Saharan Africa Both
High-income Asia Pacific Both
High-income North America Both
North Africa and Middle East Both
Oceania Both
South Asia Both
Rate per 100000
Period

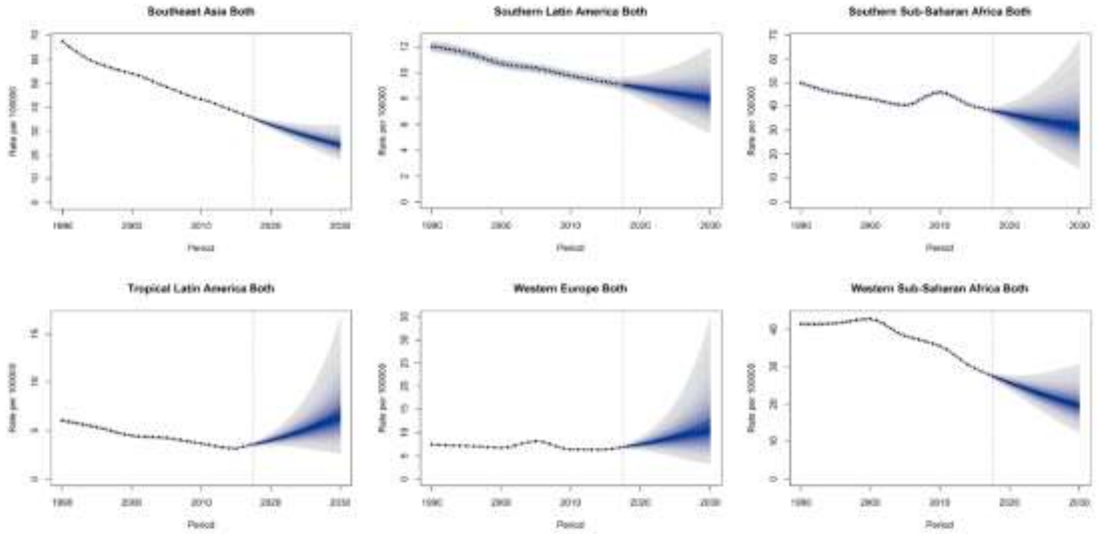

Figure S31. Trends in prevalence rates of 21 GBD regions from 1990 to 2030 for both sexes by BAPC model.

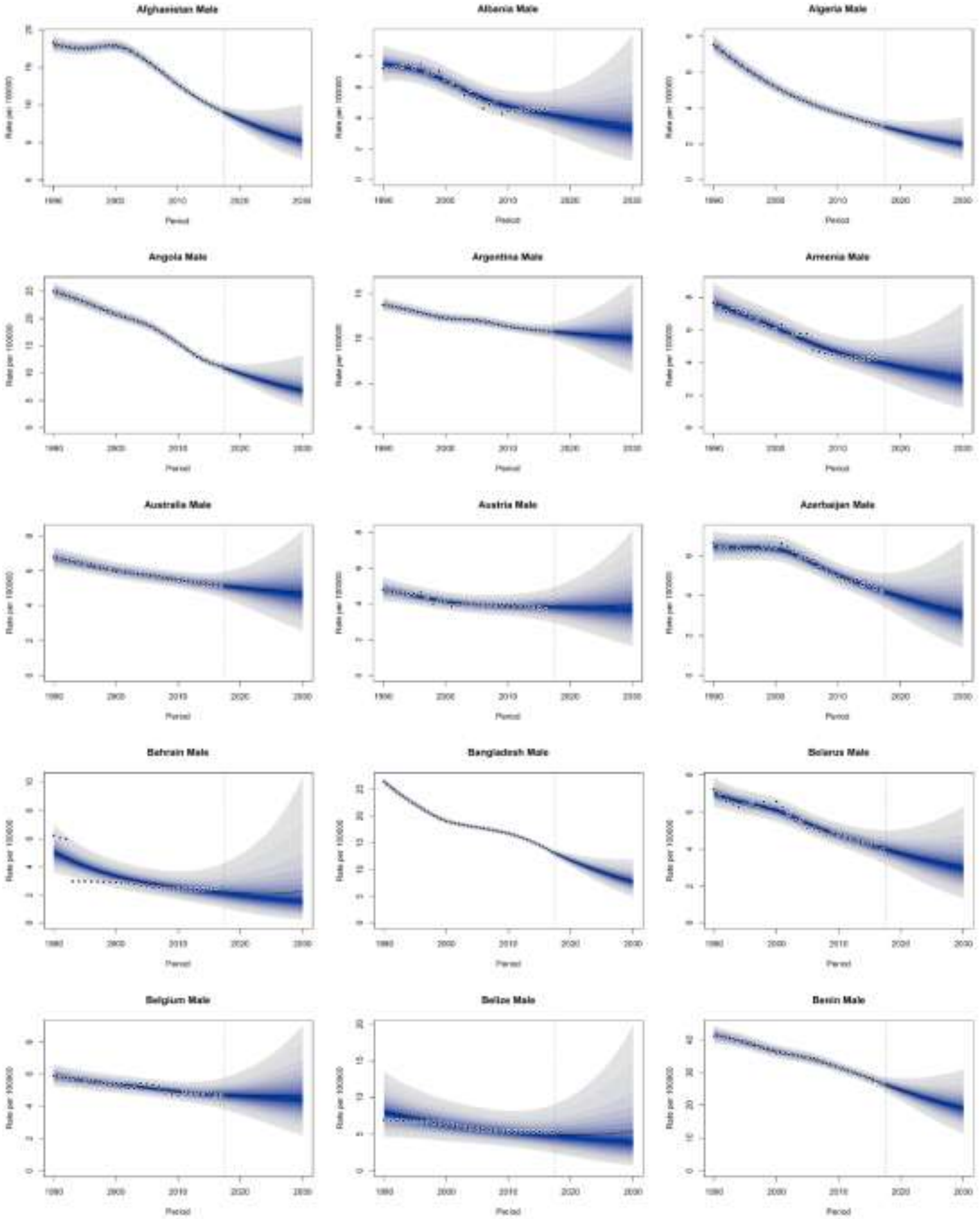
Afghanistan Male
Albania Male
Algeria Male
Angola Male
Argentina Male
Armenia Male
Australia Male
Austria Male
Azerbaijan Male
Bahrain Male
Bangladesh Male
Belarus Male
Belgium Male
Belize Male
Benin Male
Rate per 100000
Period

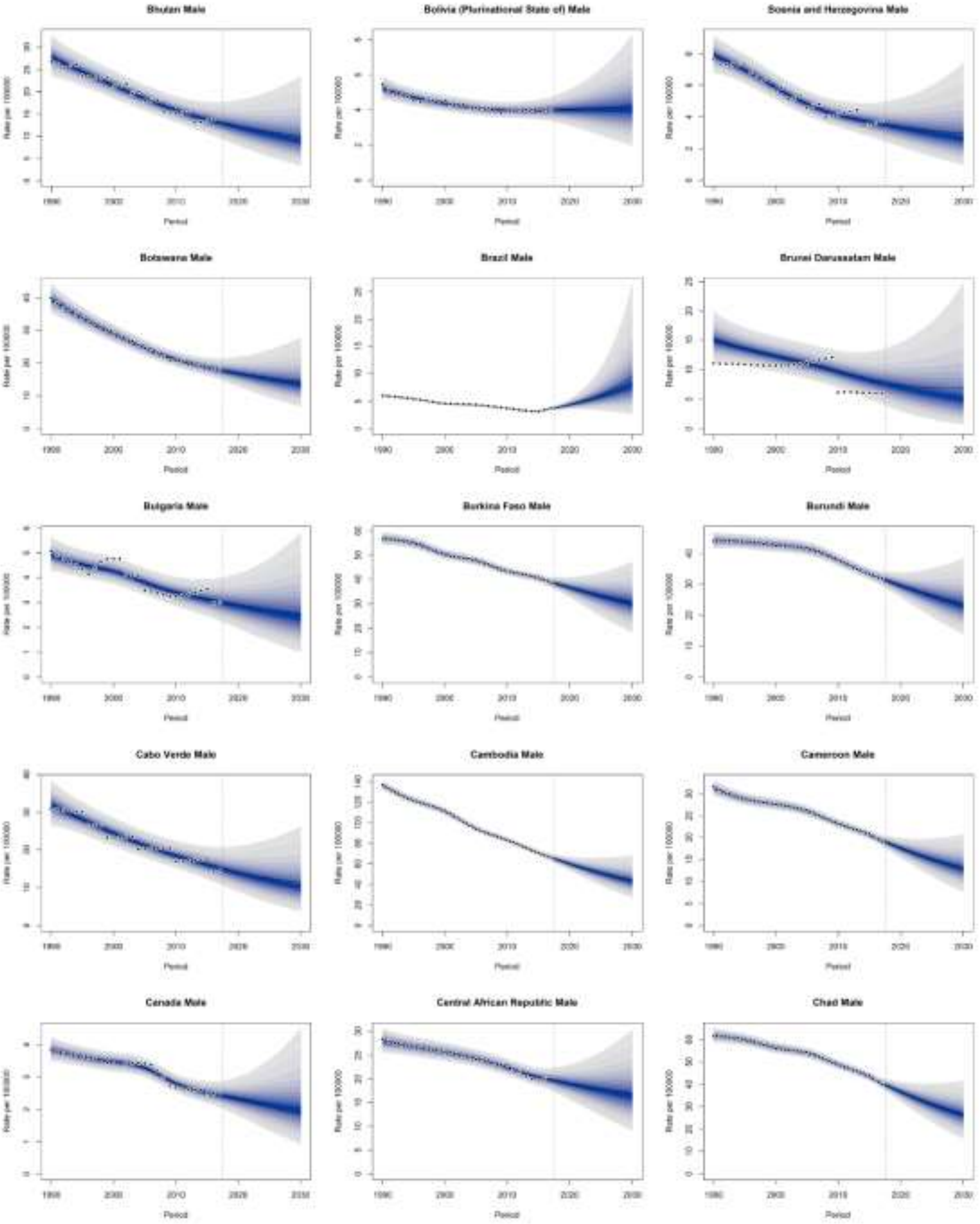
Bhutan Male
Bolivia (Plurinational State of) Male
Bosnia and Herzegovina Male
Botswana Male
Brazil Male
Brunei Darussalam Male
Bulgaria Male
Burkina Faso Male
Burundi Male
Cabo Verde Male
Cambodia Male
Cameroon Male
Canada Male
Central African Republic Male
Chad Male

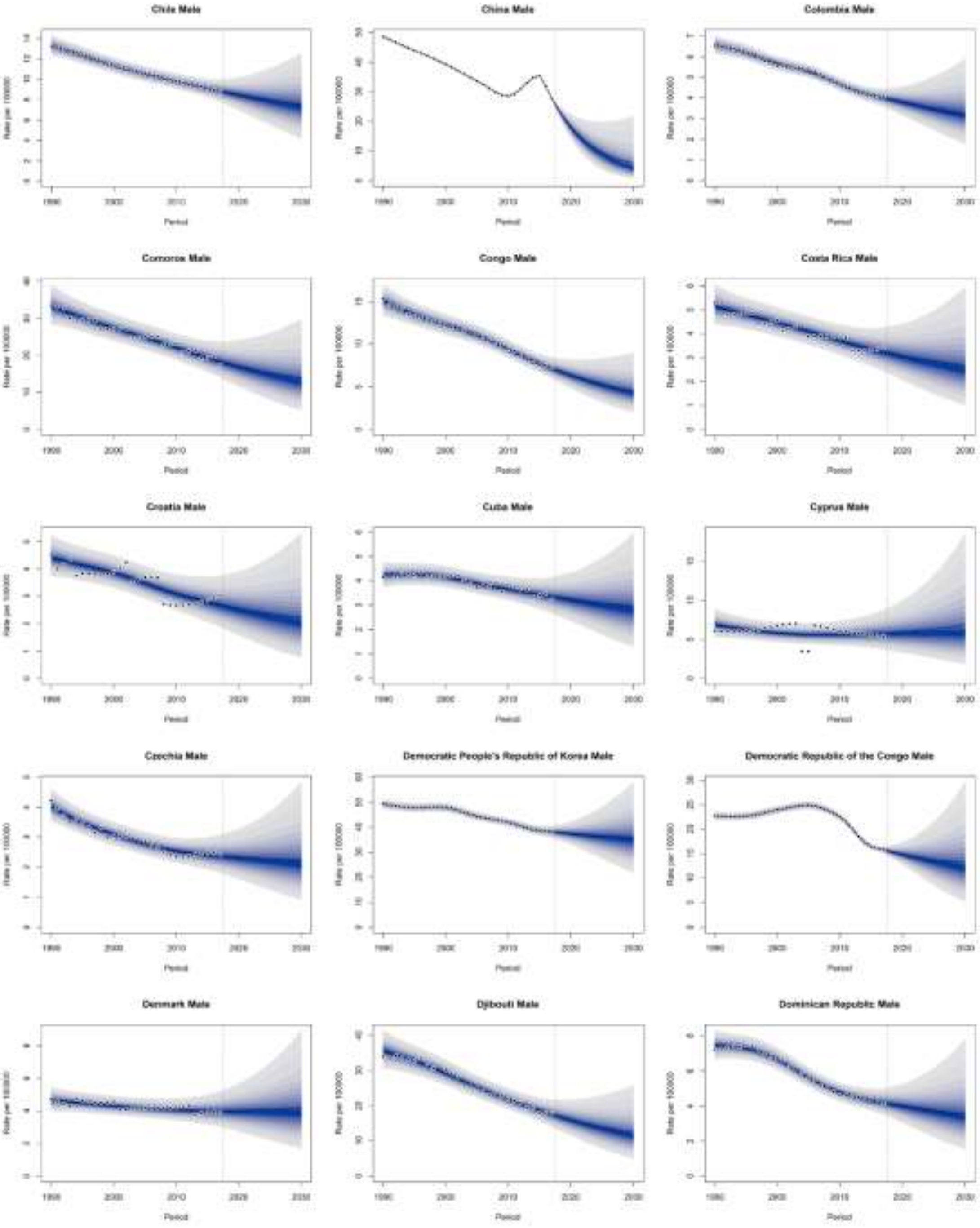
Chile Male
China Male
Colombia Male
Comoros Male
Congo Male
Costa Rica Male
Croatia Male
Cuba Male
Cyprus Male
Czechia Male
Democratic People's Republic of Korea Male
Democratic Republic of the Congo Male
Denmark Male
Djibouti Male
Dominican Republic Male
Period
Rate per 100000

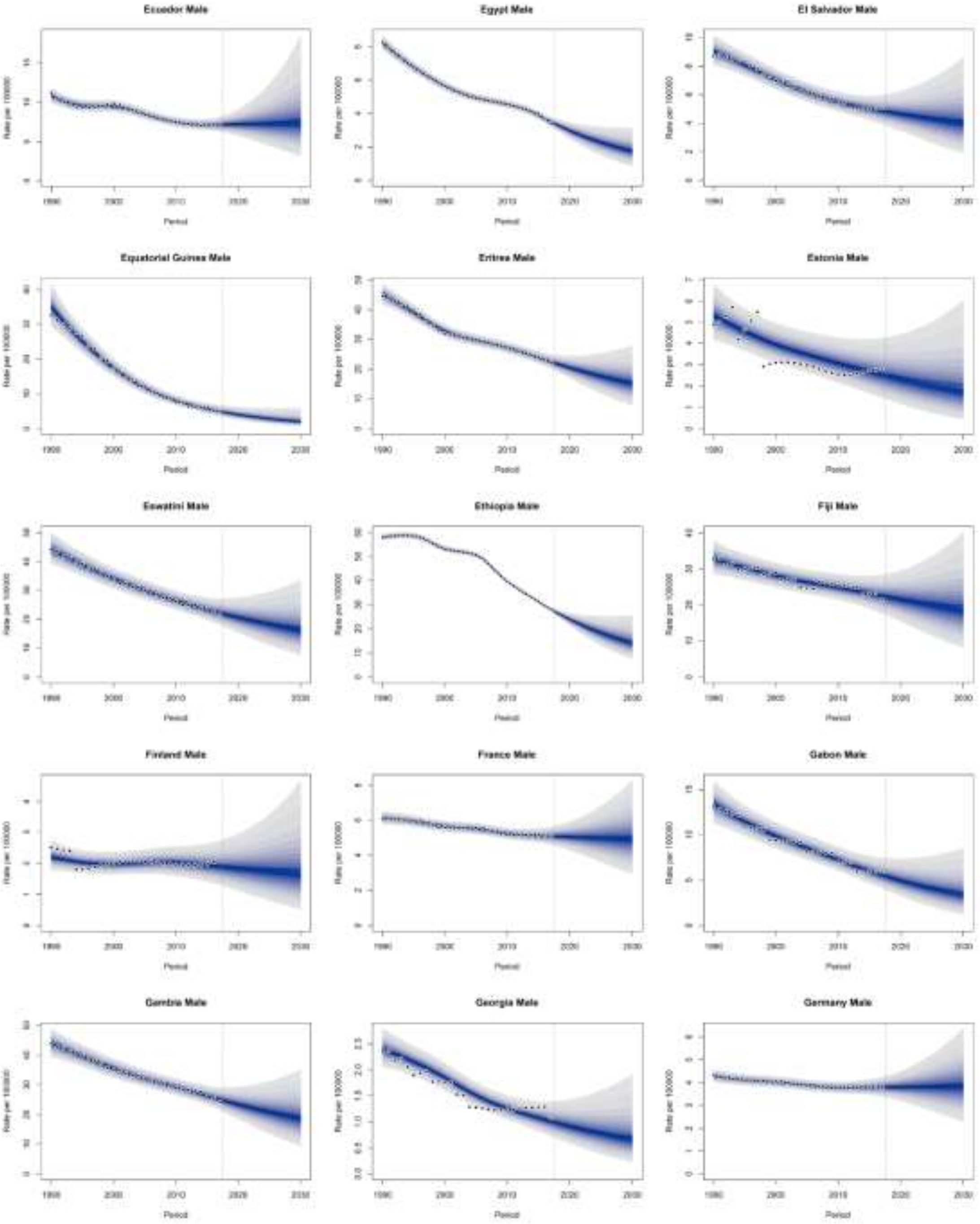
Ecuador Male
Egypt Male
El Salvador Male
Equatorial Guinea Male
Eritrea Male
Estonia Male
Eswatini Male
Ethiopia Male
Fiji Male
Finland Male
France Male
Gabon Male
Gambia Male
Georgia Male
Germany Male
Rate per 100000
Period

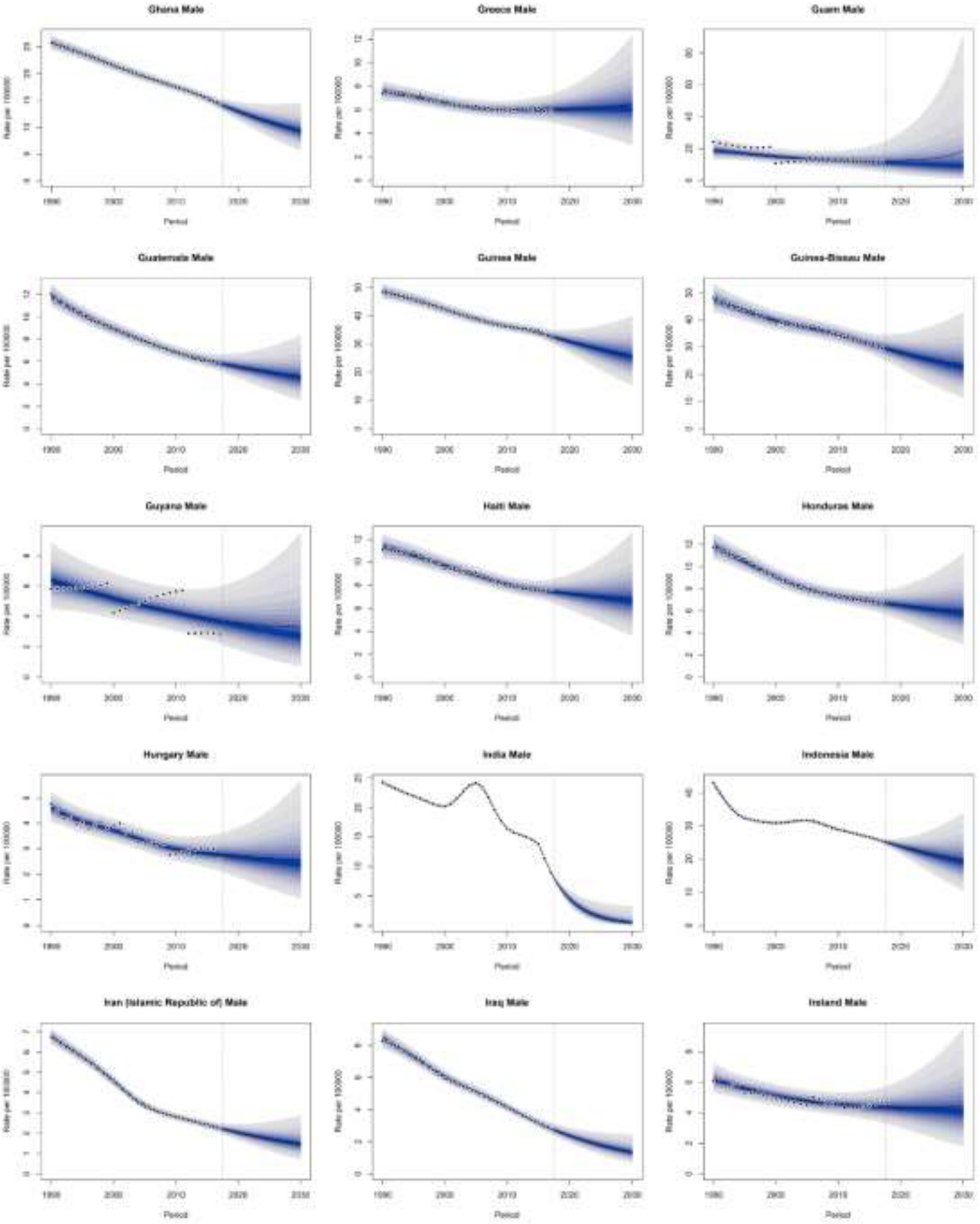
Ghana Male
Greece Male
Guam Male
Guatemala Male
Guinea Male
Guinea-Bissau Male
Guyana Male
Haiti Male
Honduras Male
Hungary Male
India Male
Indonesia Male
Iran (Islamic Republic of) Male
Iraq Male
Ireland Male
Rate per 100000
Period

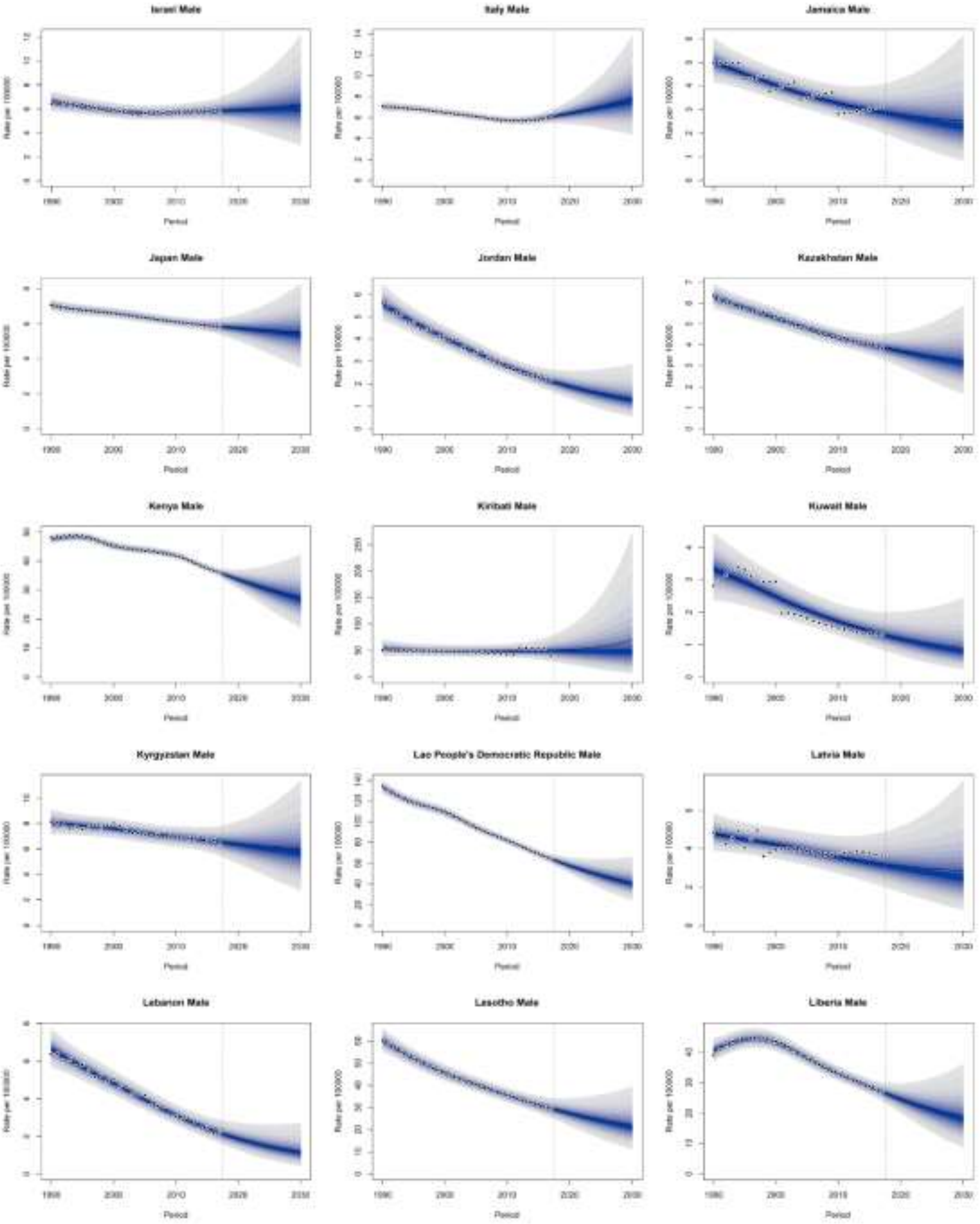
Israel Male
Italy Male
Jamaica Male
Japan Male
Jordan Male
Kazakhstan Male
Kenya Male
Kiribati Male
Kuwait Male
Kyrgyzstan Male
Lao People's Democratic Republic Male
Latvia Male
Lebanon Male
Lesotho Male
Liberia Male
Period
Rate per 100000

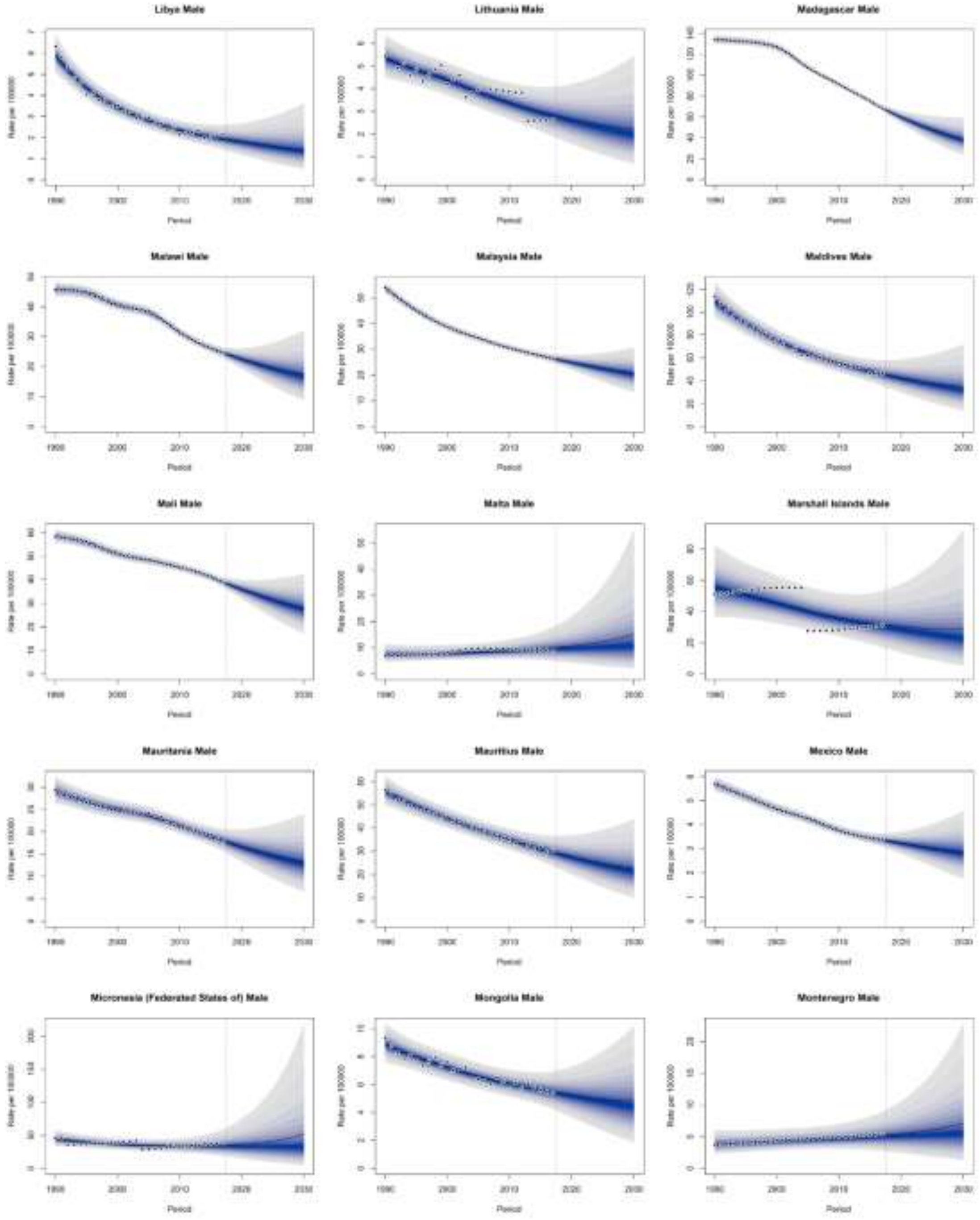
Libya Male
Lithuania Male
Madagascar Male
Malawi Male
Malaysia Male
Maldives Male
Mali Male
Malta Male
Marshall Islands Male
Mauritania Male
Mauritius Male
Mexico Male
Micronesia (Federated States of) Male
Mongolia Male
Montenegro Male

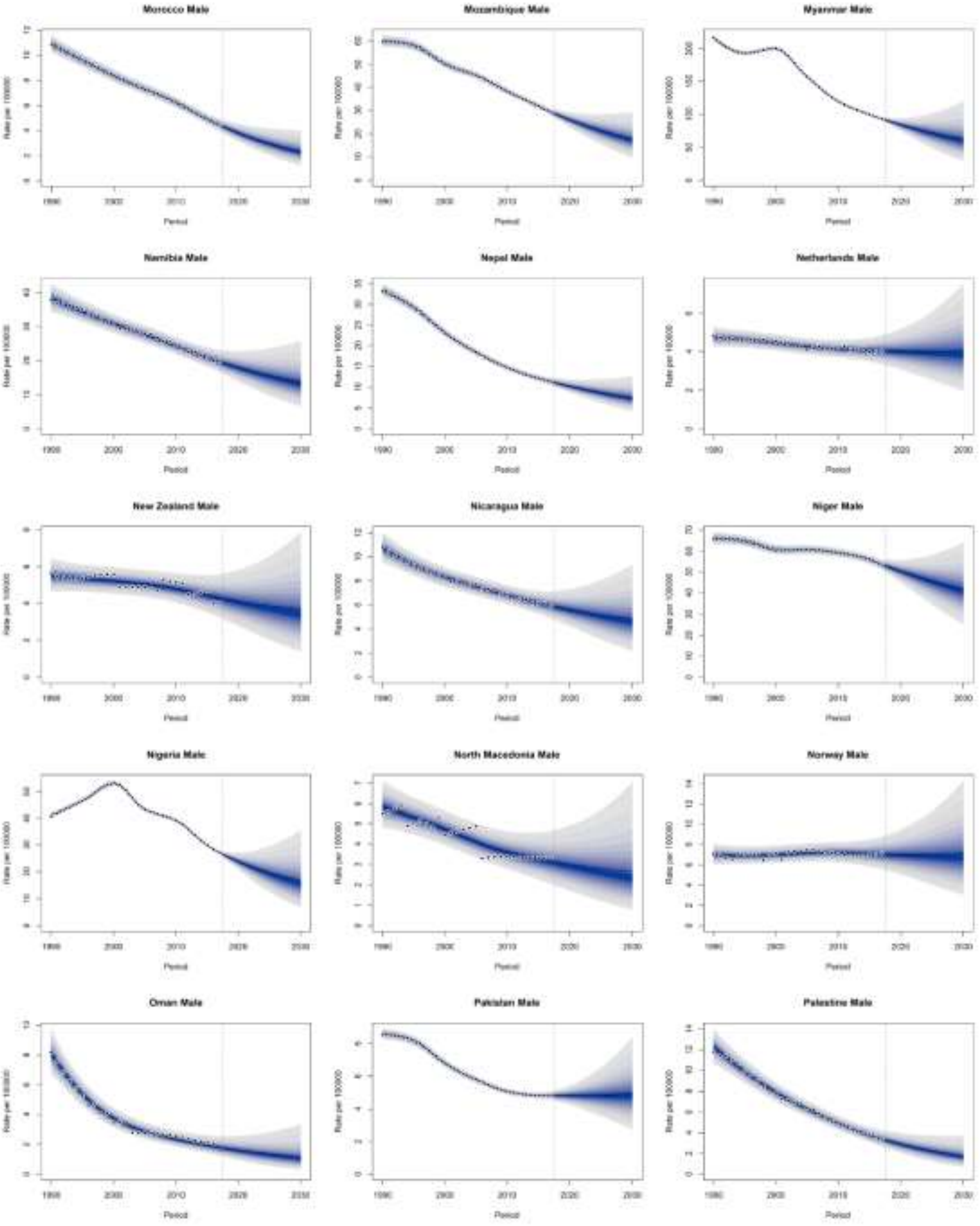
Morocco Male
Mozambique Male
Myanmar Male
Namibia Male
Nepal Male
Netherlands Male
New Zealand Male
Nicaragua Male
Niger Male
Nigeria Male
North Macedonia Male
Norway Male
Oman Male
Pakistan Male
Palestine Male

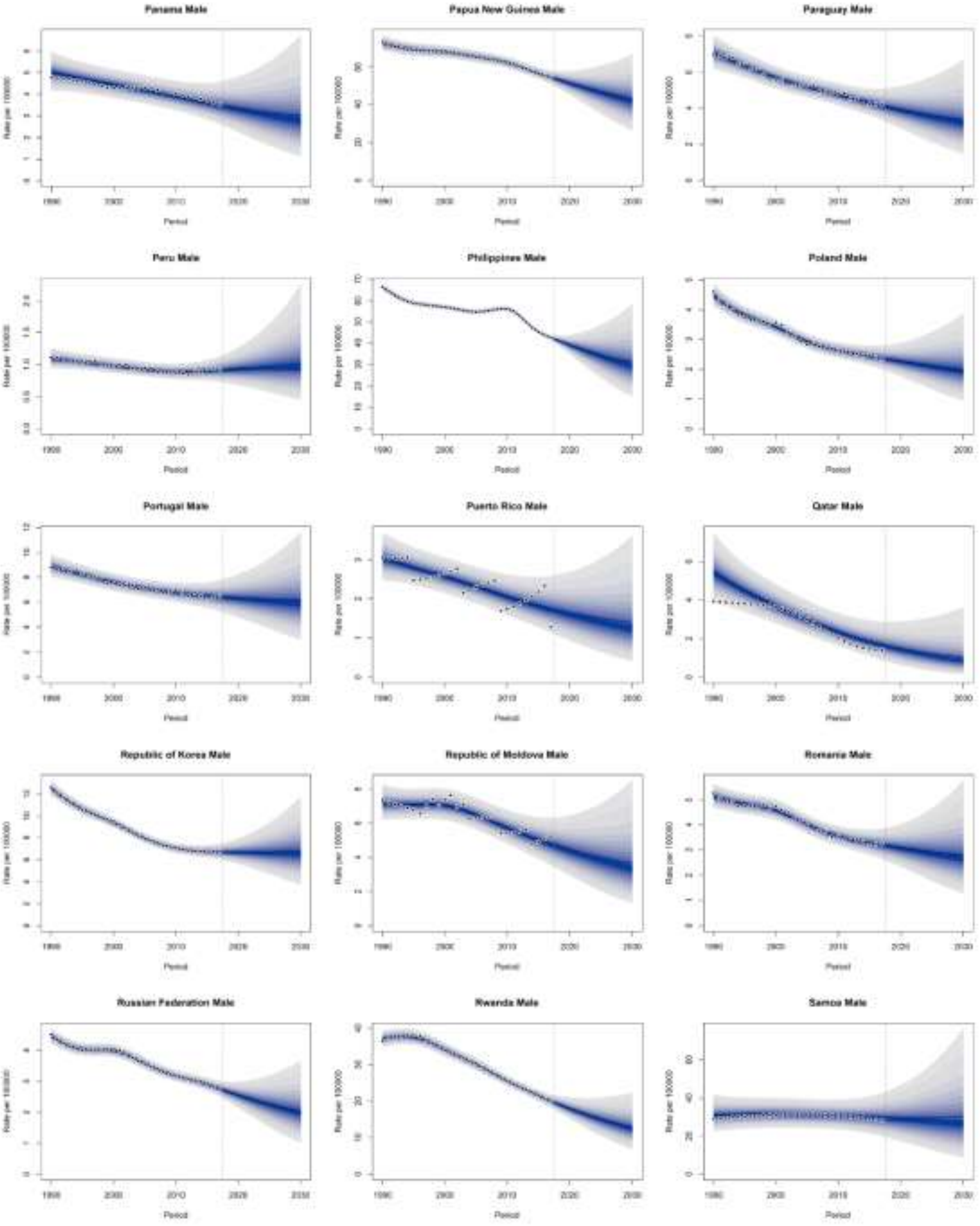
Panama Male
Papua New Guinea Male
Paraguay Male
Peru Male
Philippines Male
Poland Male
Portugal Male
Puerto Rico Male
Qatar Male
Republic of Korea Male
Republic of Moldova Male
Romania Male
Russian Federation Male
Rwanda Male
Samoa Male

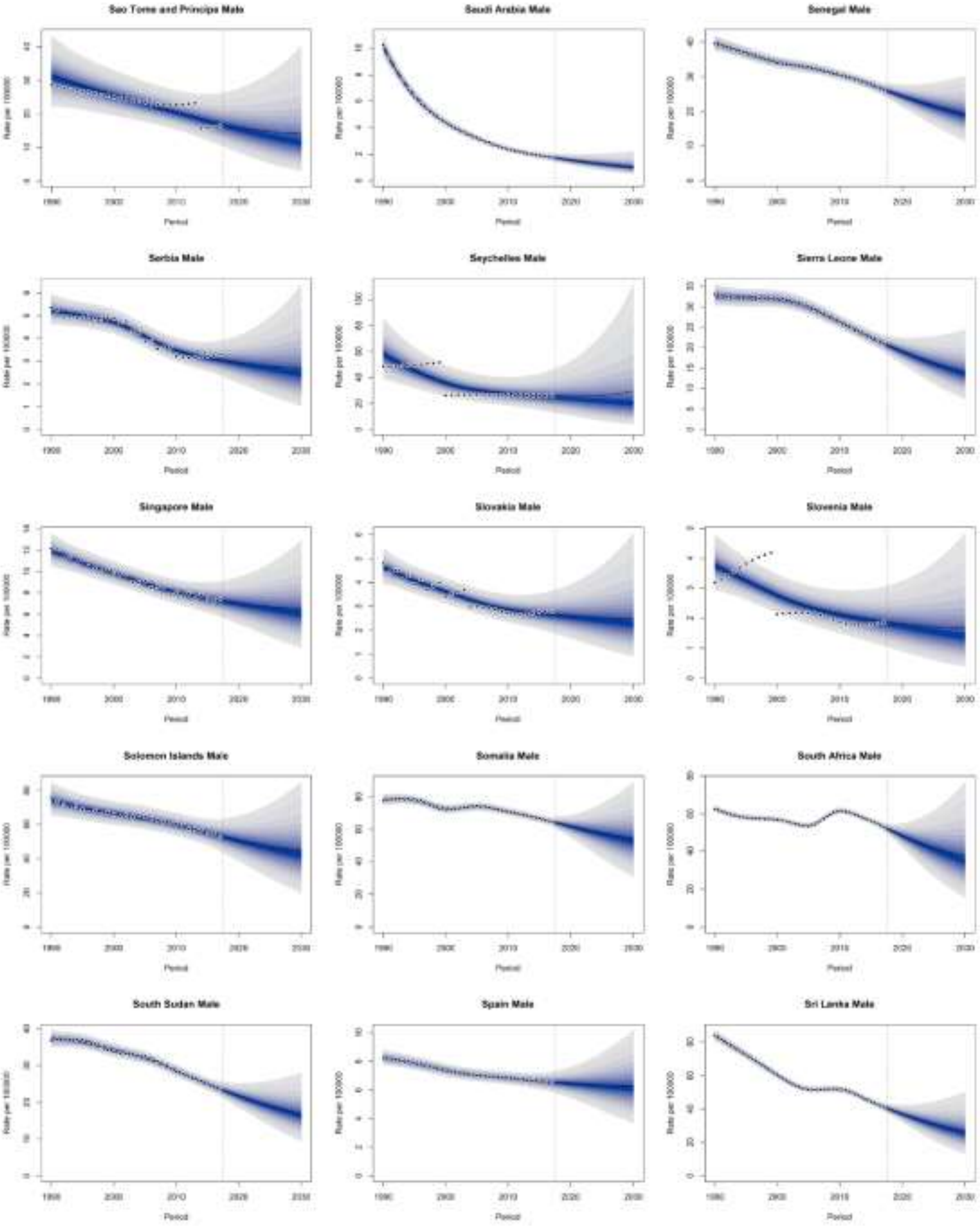
Sao Tome and Principe Male
Saudi Arabia Male
Senegal Male
Serbia Male
Seychelles Male
Sierra Leone Male
Singapore Male
Slovakia Male
Slovenia Male
Solomon Islands Male
Somalia Male
South Africa Male
South Sudan Male
Spain Male
Sri Lanka Male
Rate per 100000
Period

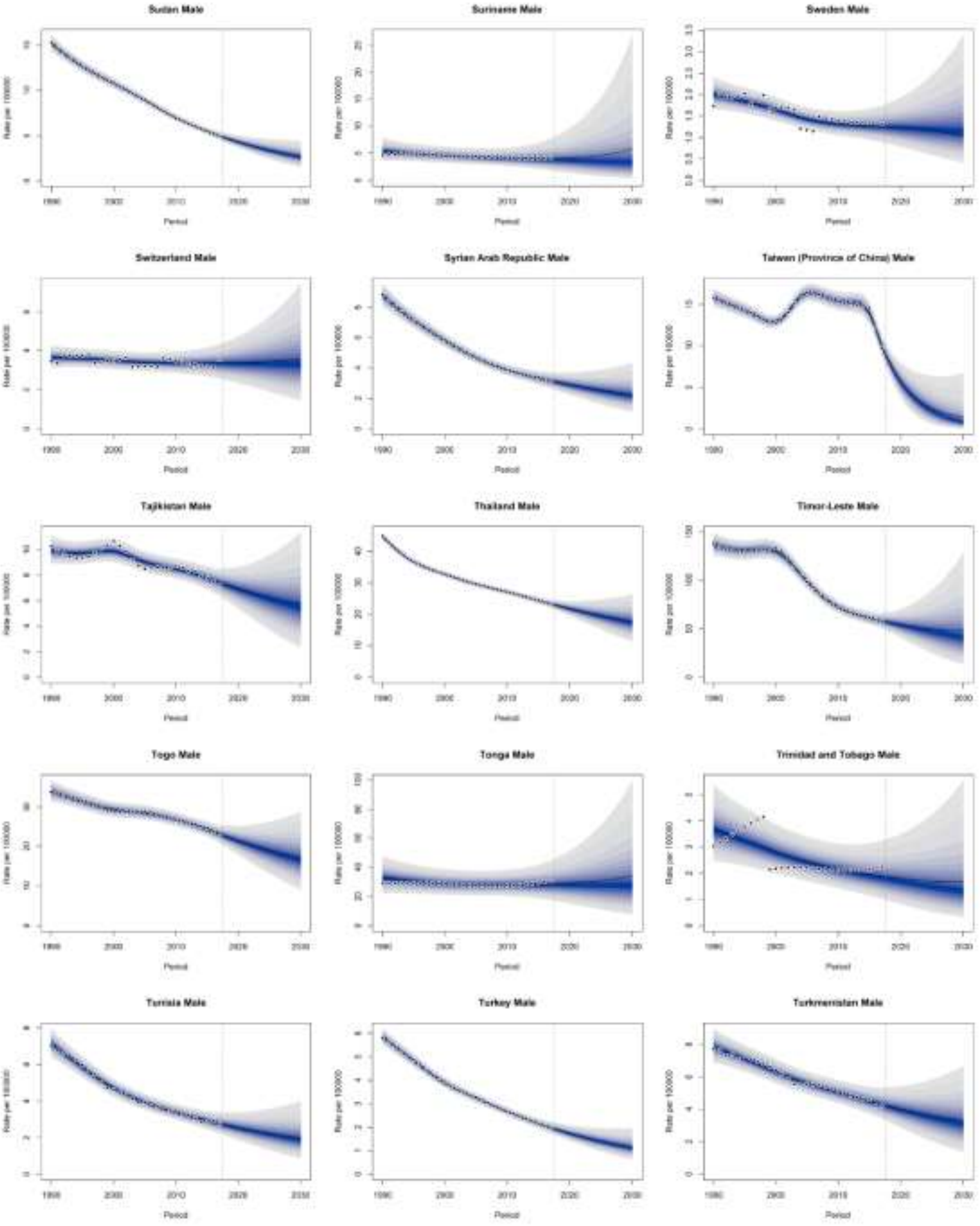
Sudan Male
Suriname Male
Sweden Male
Switzerland Male
Syrian Arab Republic Male
Taiwan (Province of China) Male
Tajikistan Male
Thailand Male
Timor-Leste Male
Togo Male
Tonga Male
Trinidad and Tobago Male
Tunisia Male
Turkey Male
Turkmenistan Male

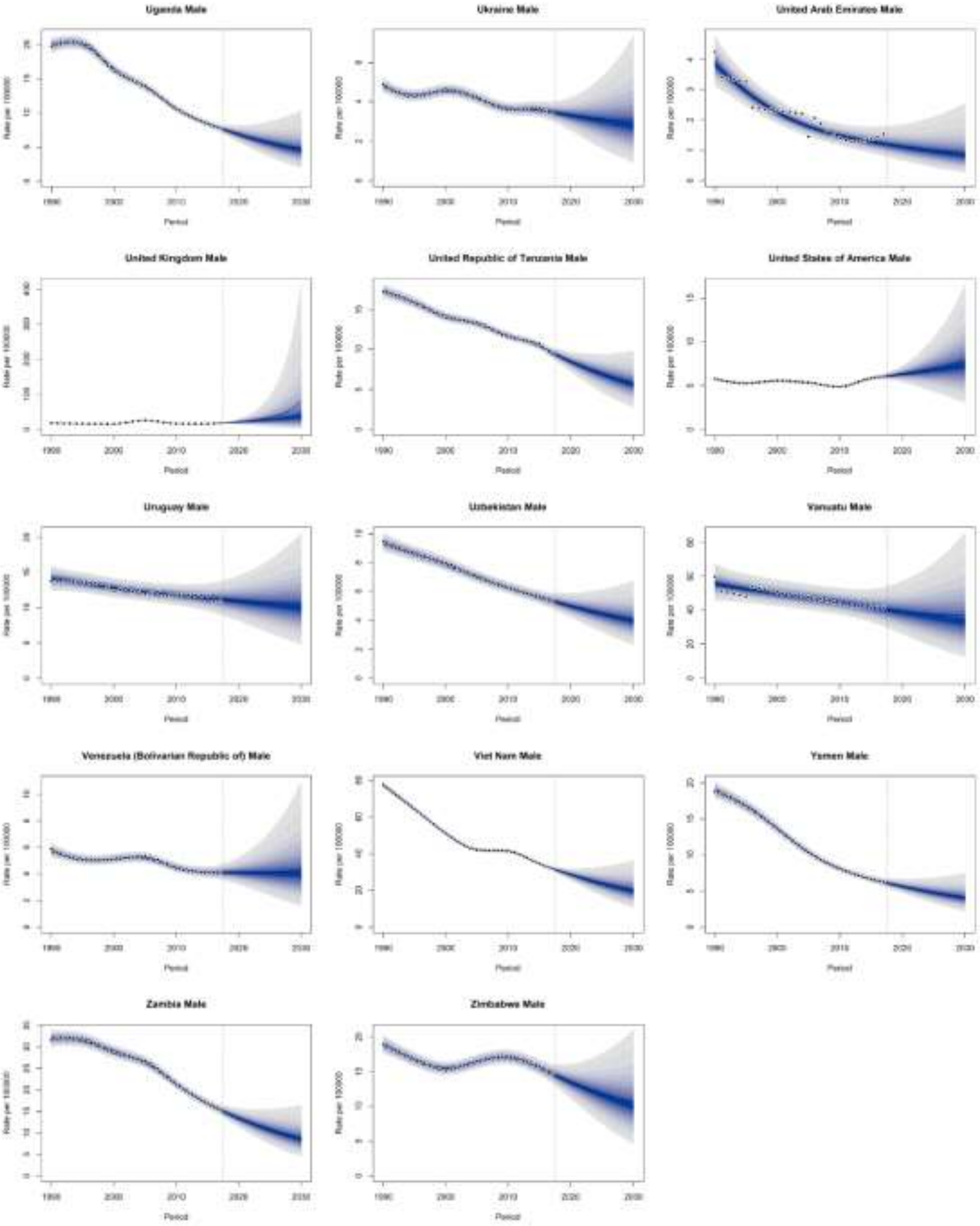

Figure S32. Trends in prevalence rates of 179 countries and territories from 1990 to 2030 for males by BAPC model.

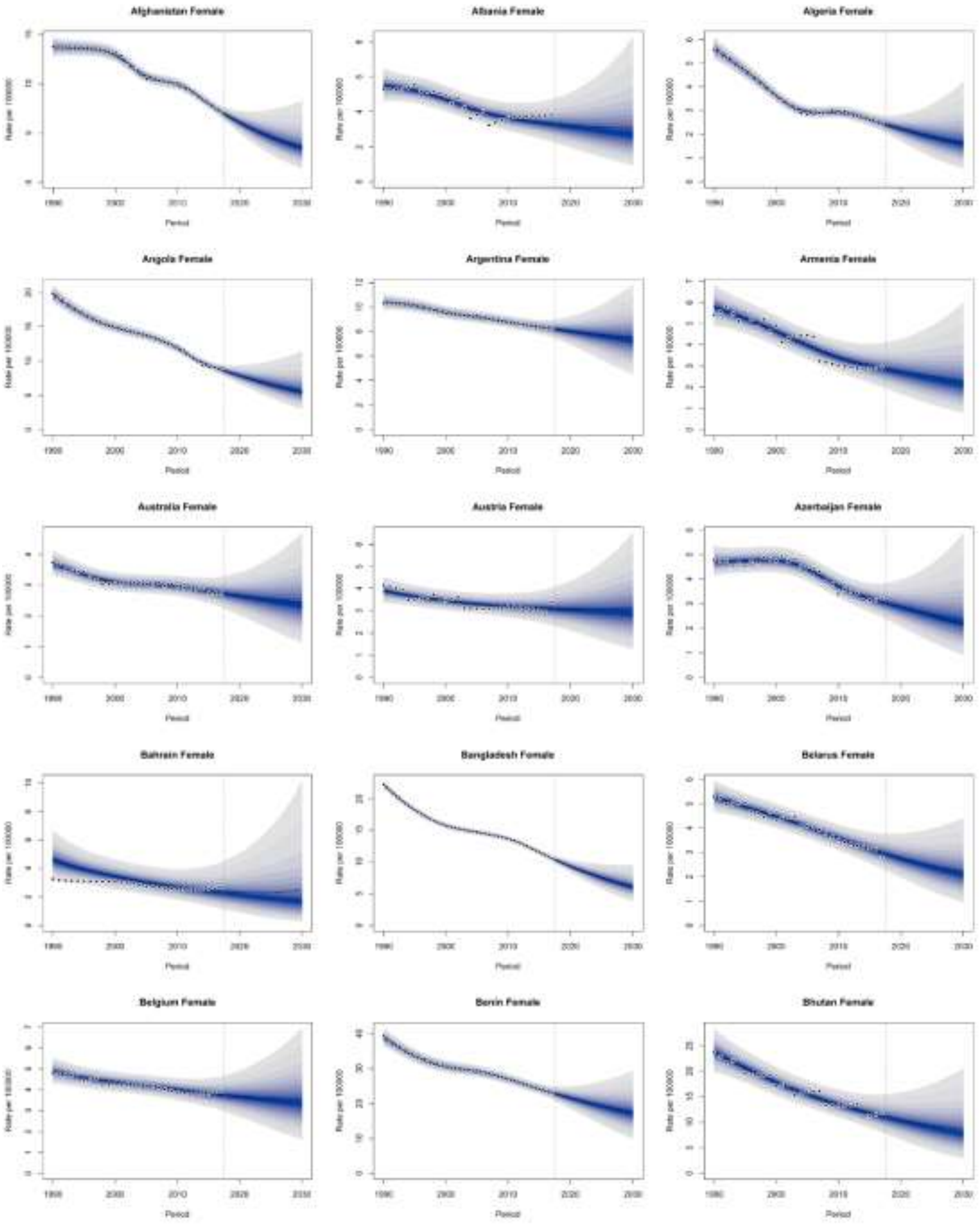
Afghanistan Female
Albania Female
Algeria Female
Angola Female
Argentina Female
Armenia Female
Australia Female
Austria Female
Azerbaijan Female
Bahrain Female
Bangladesh Female
Belarus Female
Belgium Female
Benin Female
Bhutan Female

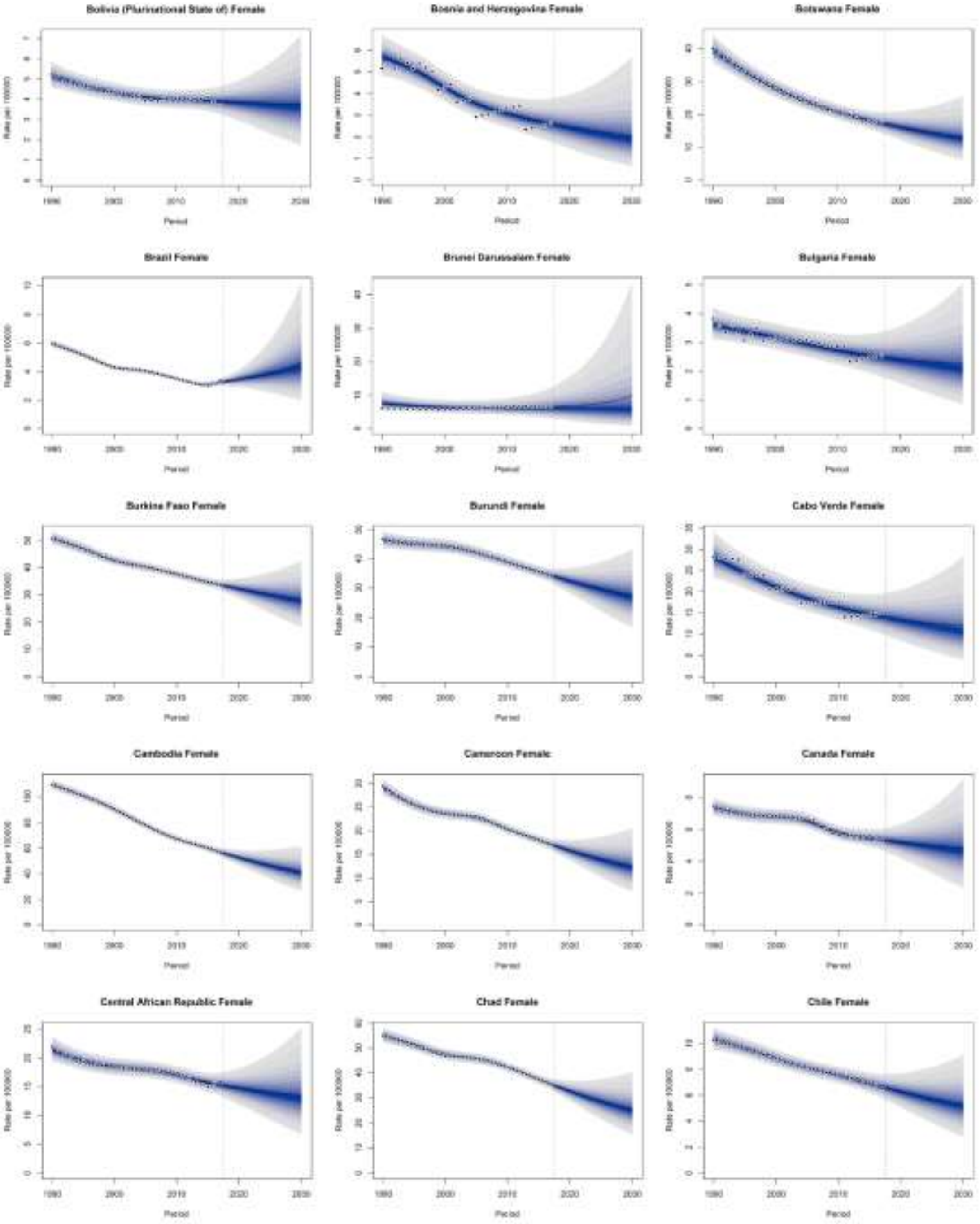
Bolivia (Plurinational State of) Female
Bosnia and Herzegovina Female
Botswana Female
Brazil Female
Brunei Darussalam Female
Bulgaria Female
Burkina Faso Female
Burundi Female
Cabo Verde Female
Cambodia Female
Cameroon Female
Canada Female
Central African Republic Female
Chad Female
Chile Female

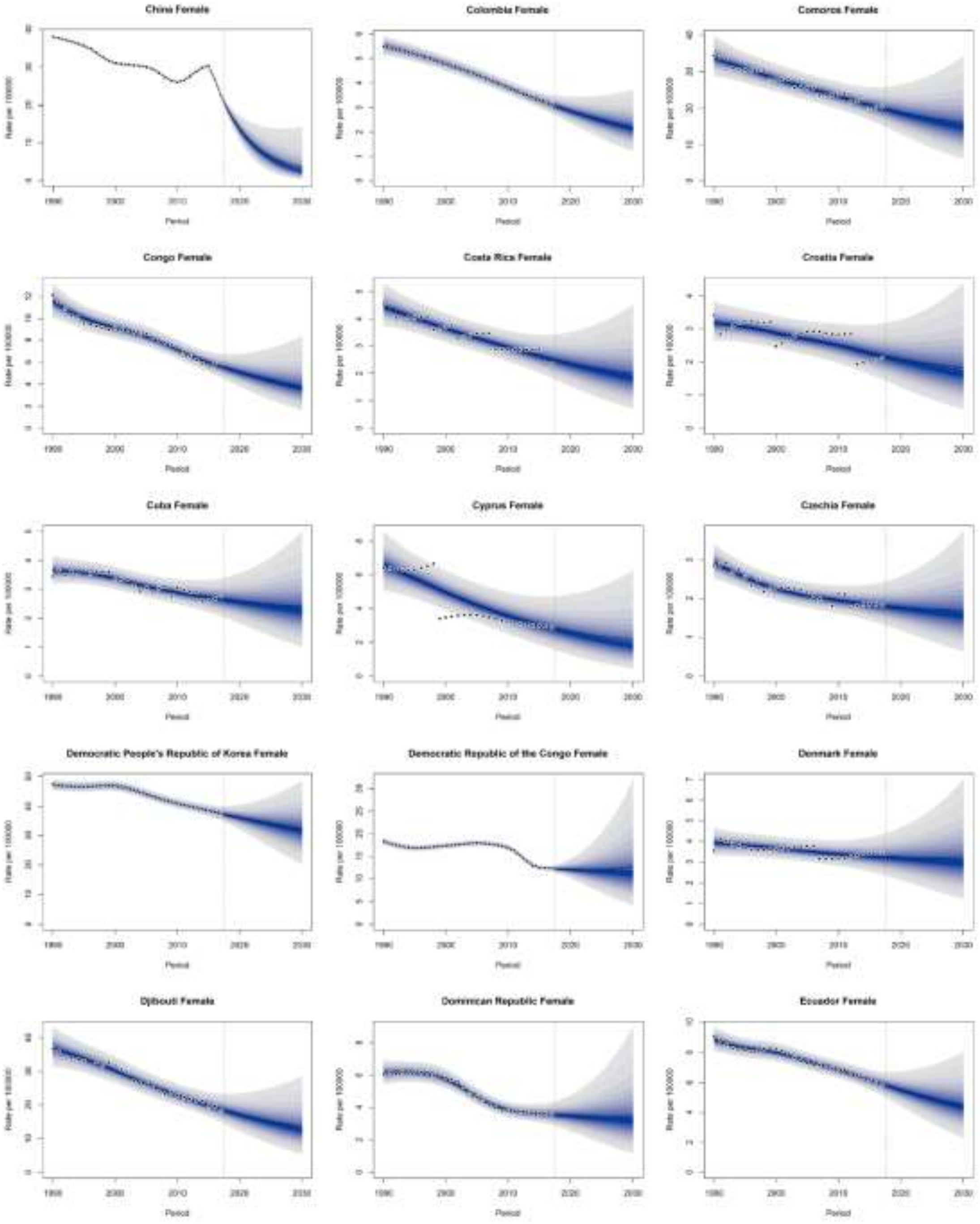
China Female
Colombia Female
Comoros Female
Congo Female
Costa Rica Female
Croatia Female
Cuba Female
Cyprus Female
Czechia Female
Democratic People's Republic of Korea Female
Democratic Republic of the Congo Female
Denmark Female
Djibouti Female
Dominican Republic Female
Ecuador Female

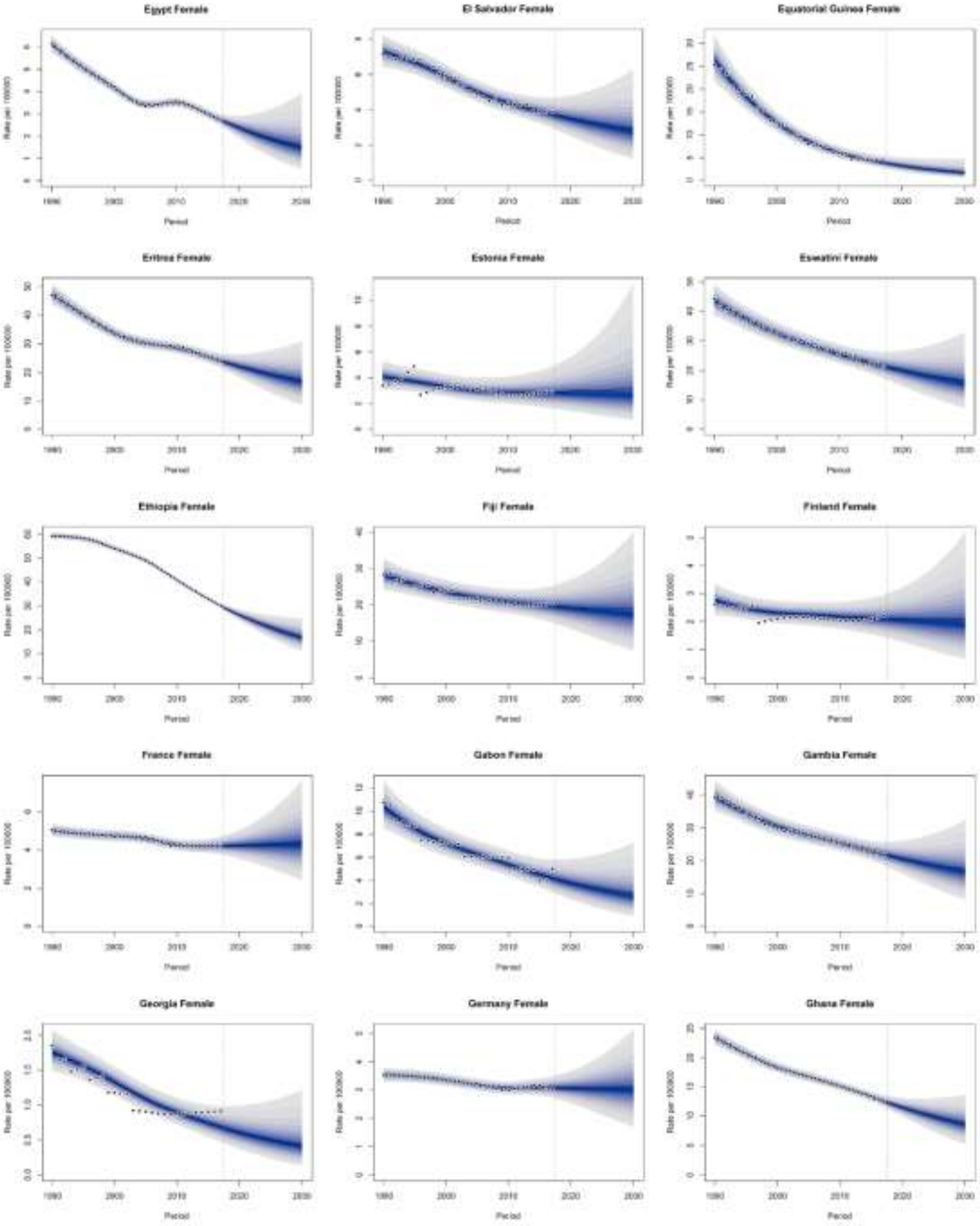
Egypt Female
El Salvador Female
Equatorial Guinea Female
Eritrea Female
Estonia Female
Eswatini Female
Ethiopia Female
Fiji Female
Finland Female
France Female
Gabon Female
Gambia Female
Georgia Female
Germany Female
Ghana Female
Period
Rate per 100000

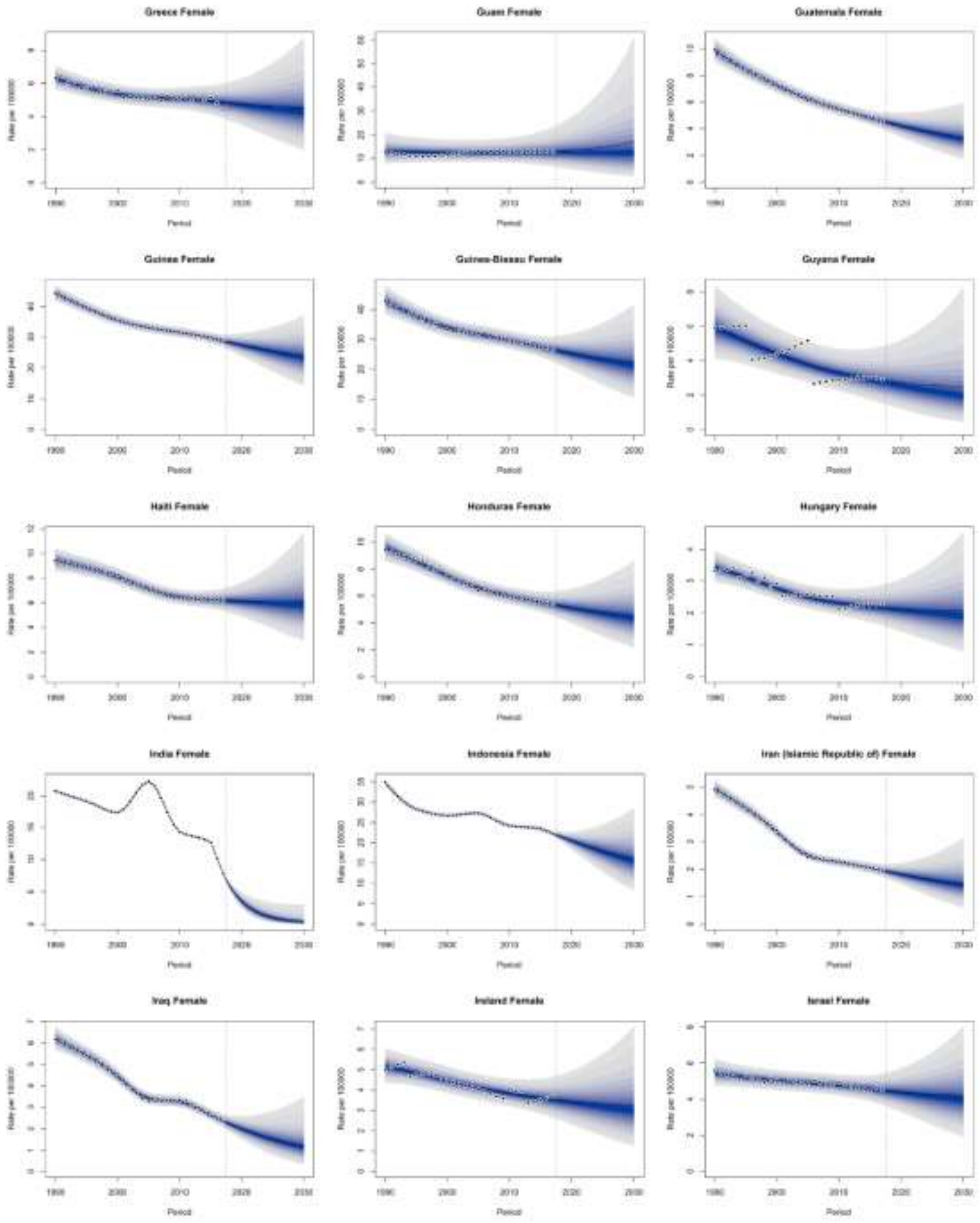

Greece Female
Guam Female
Guatemala Female
Guinea Female
Guinea-Bissau Female
Guyana Female
Haiti Female
Honduras Female
Hungary Female
India Female
Indonesia Female
Iran (Islamic Republic of) Female
Iraq Female
Ireland Female
Israel Female
Period

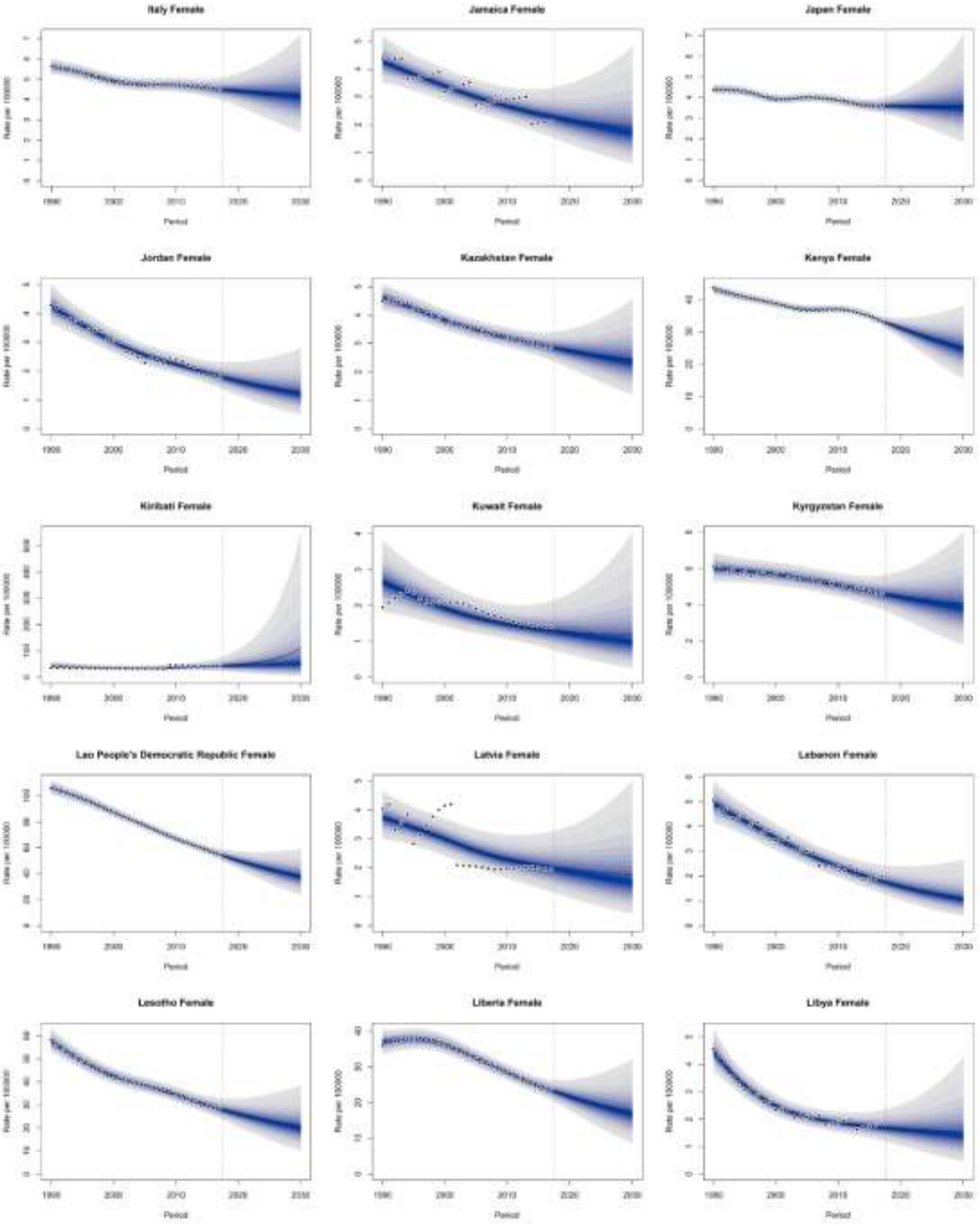
Italy Female
Jamaica Female
Japan Female
Jordan Female
Kazakhstan Female
Kenya Female
Kiribati Female
Kuwait Female
Kyrgyzstan Female
Lao People's Democratic Republic Female
Latvia Female
Lebanon Female
Lesotho Female
Liberia Female
Libya Female
Rate per 100000
Period

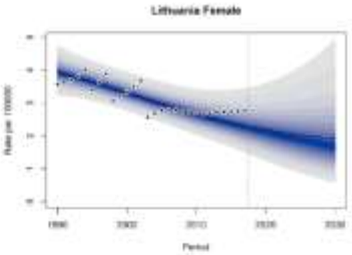
Lithuania Female
Rate per 100000
Period
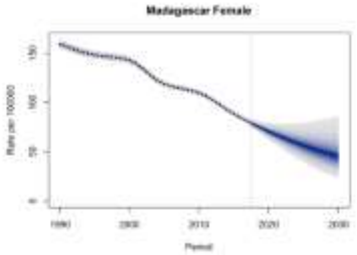
Madagascar Female
Rate per 100000
Period
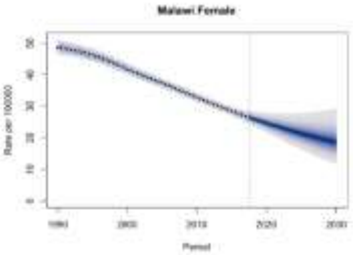
Malawi Female
Rate per 100000
Period
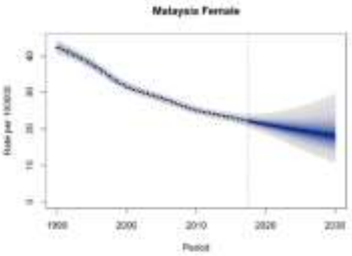
Malaysia Female
Rate per 100000
Period
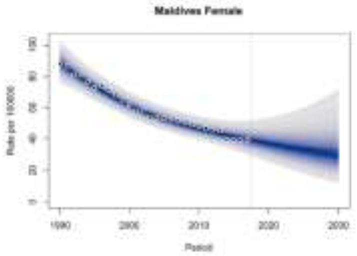
Maldives Female
Rate per 100000
Period
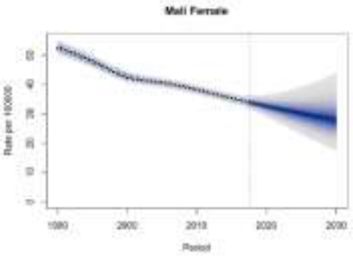
Mali Female
Rate per 100000
Period
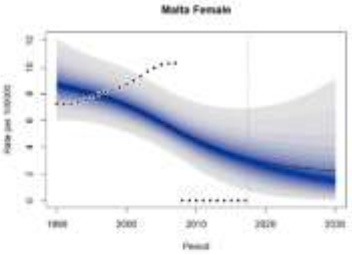
Malta Female
Rate per 100000
Period
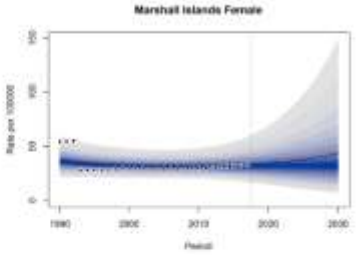
Marshall Islands Female
Rate per 100000
Period
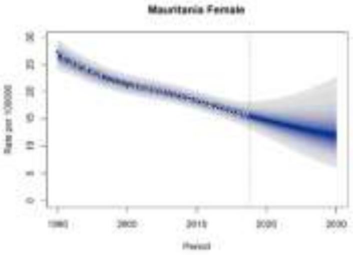
Mauritania Female
Rate per 100000
Period
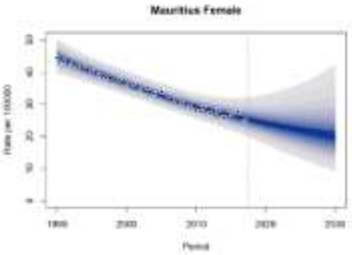
Mauritius Female
Rate per 100000
Period
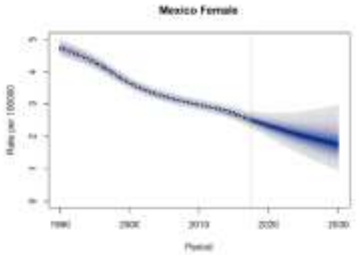
Mexico Female
Rate per 100000
Period
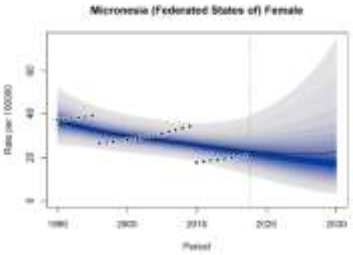
Micronesia (Federated States of) Female
Rate per 100000
Period
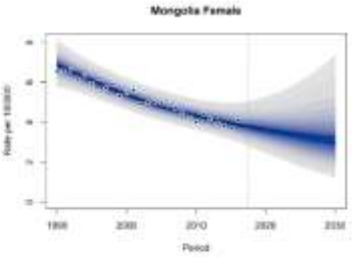
Mongolia Female
Rate per 100000
Period
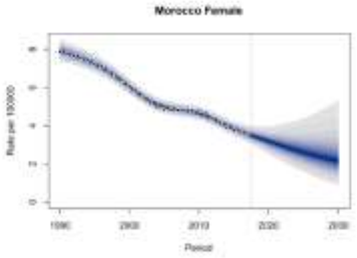
Morocco Female
Rate per 100000
Period
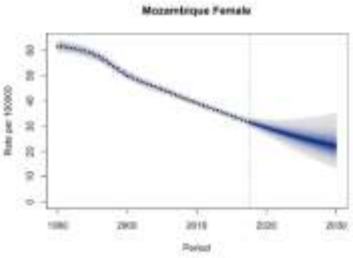
Mozambique Female
Rate per 100000
Period

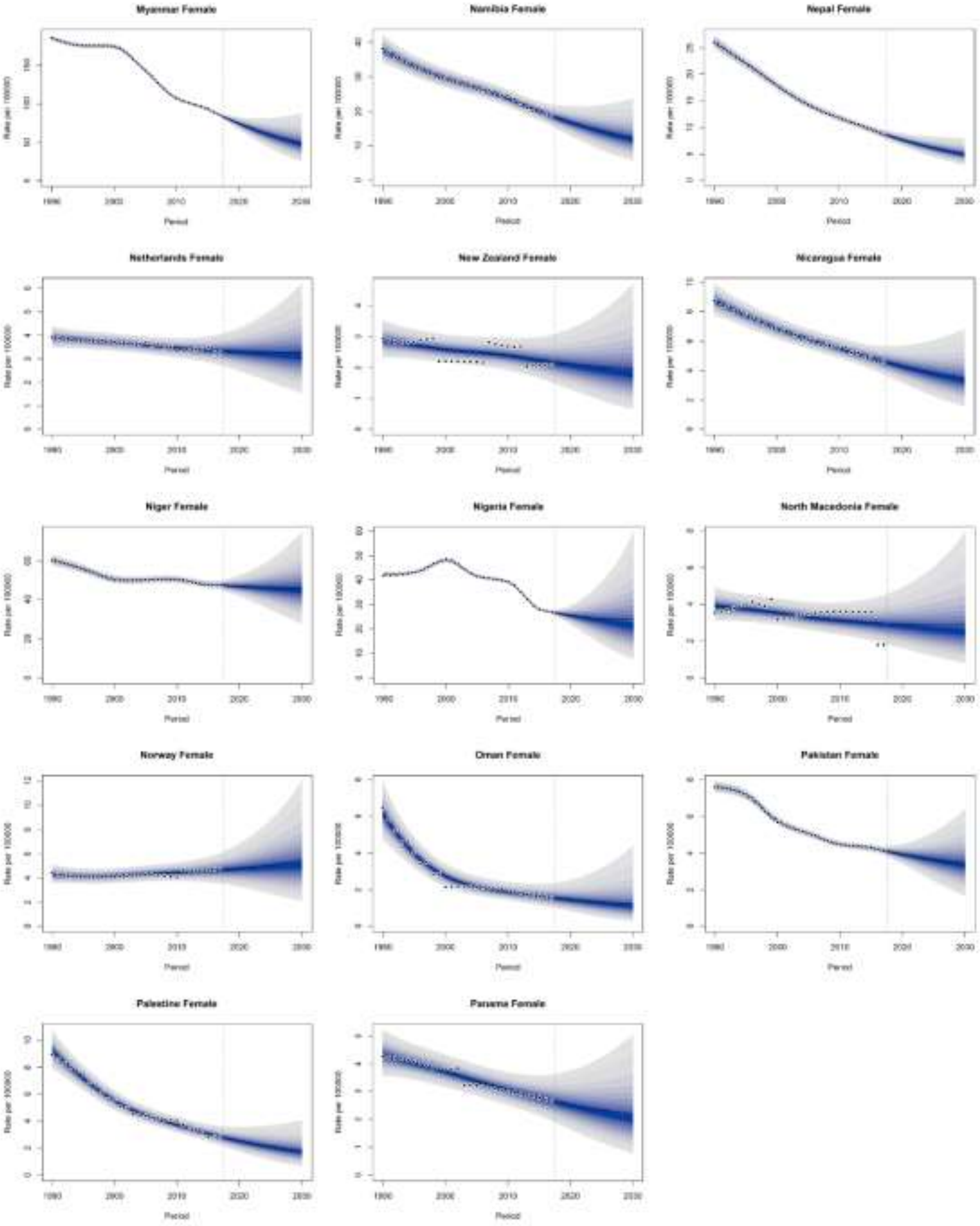
Myanmar Female
Namibia Female
Nepal Female
Netherlands Female
New Zealand Female
Nicaragua Female
Niger Female
Nigeria Female
North Macedonia Female
Norway Female
Oman Female
Pakistan Female
Palestine Female
Panama Female

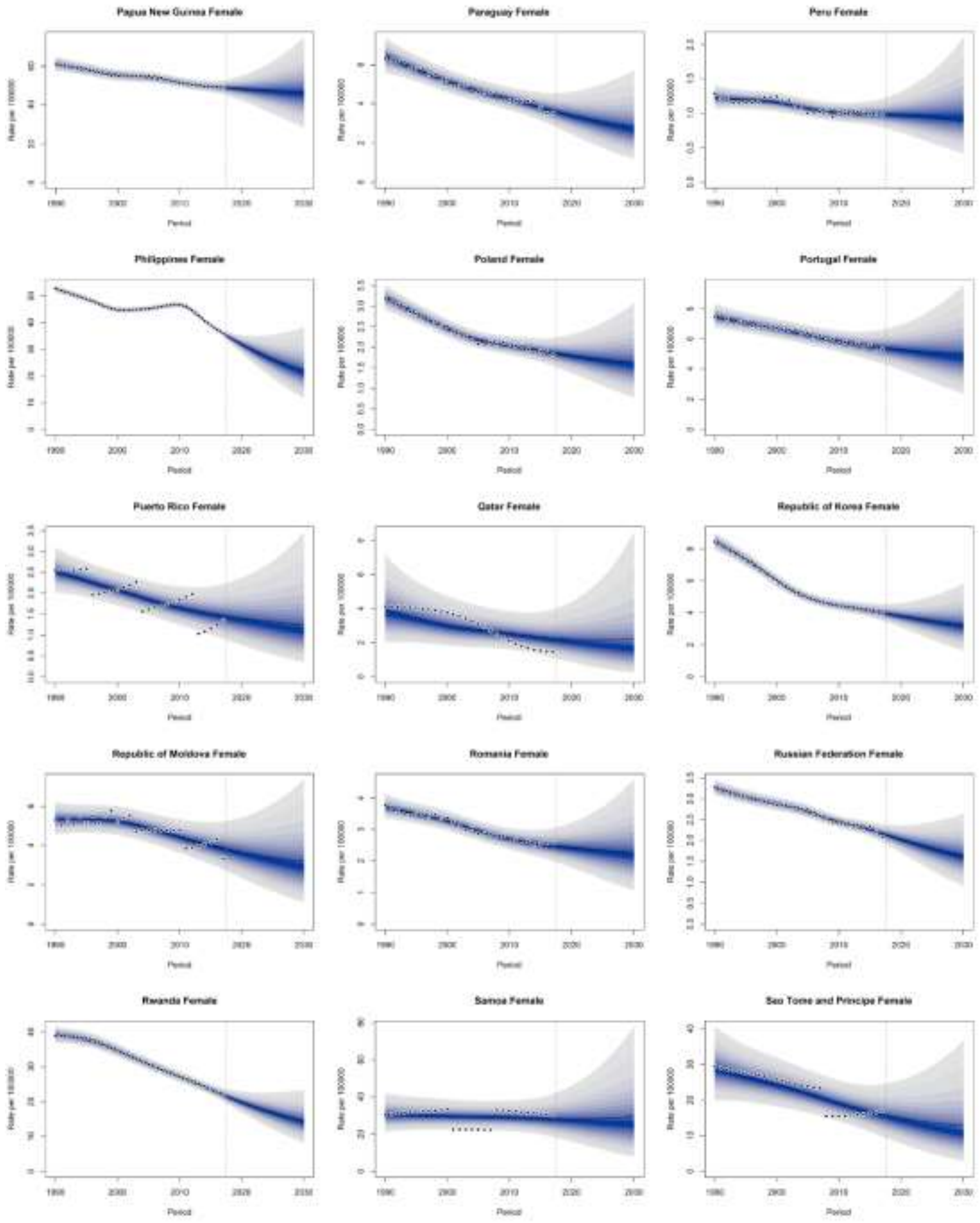
Papua New Guinea Female
Paraguay Female
Peru Female
Philippines Female
Poland Female
Portugal Female
Puerto Rico Female
Qatar Female
Republic of Korea Female
Republic of Moldova Female
Romania Female
Russian Federation Female
Rwanda Female
Samoa Female
Sao Tome and Principe Female

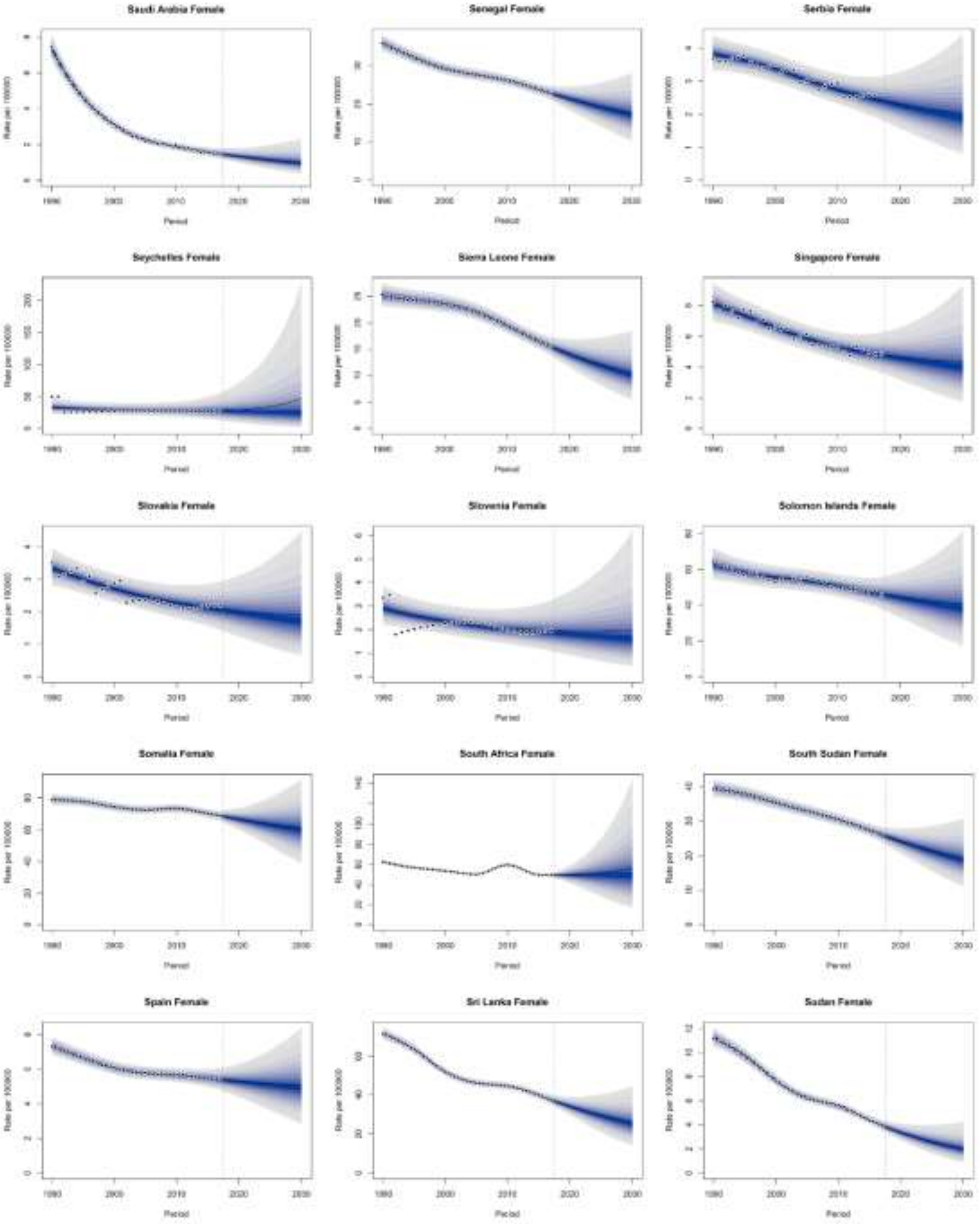
Saudi Arabia Female
Senegal Female
Serbia Female
Seychelles Female
Sierra Leone Female
Singapore Female
Slovakia Female
Slovenia Female
Solomon Islands Female
Somalia Female
South Africa Female
South Sudan Female
Spain Female
Sri Lanka Female
Sudan Female

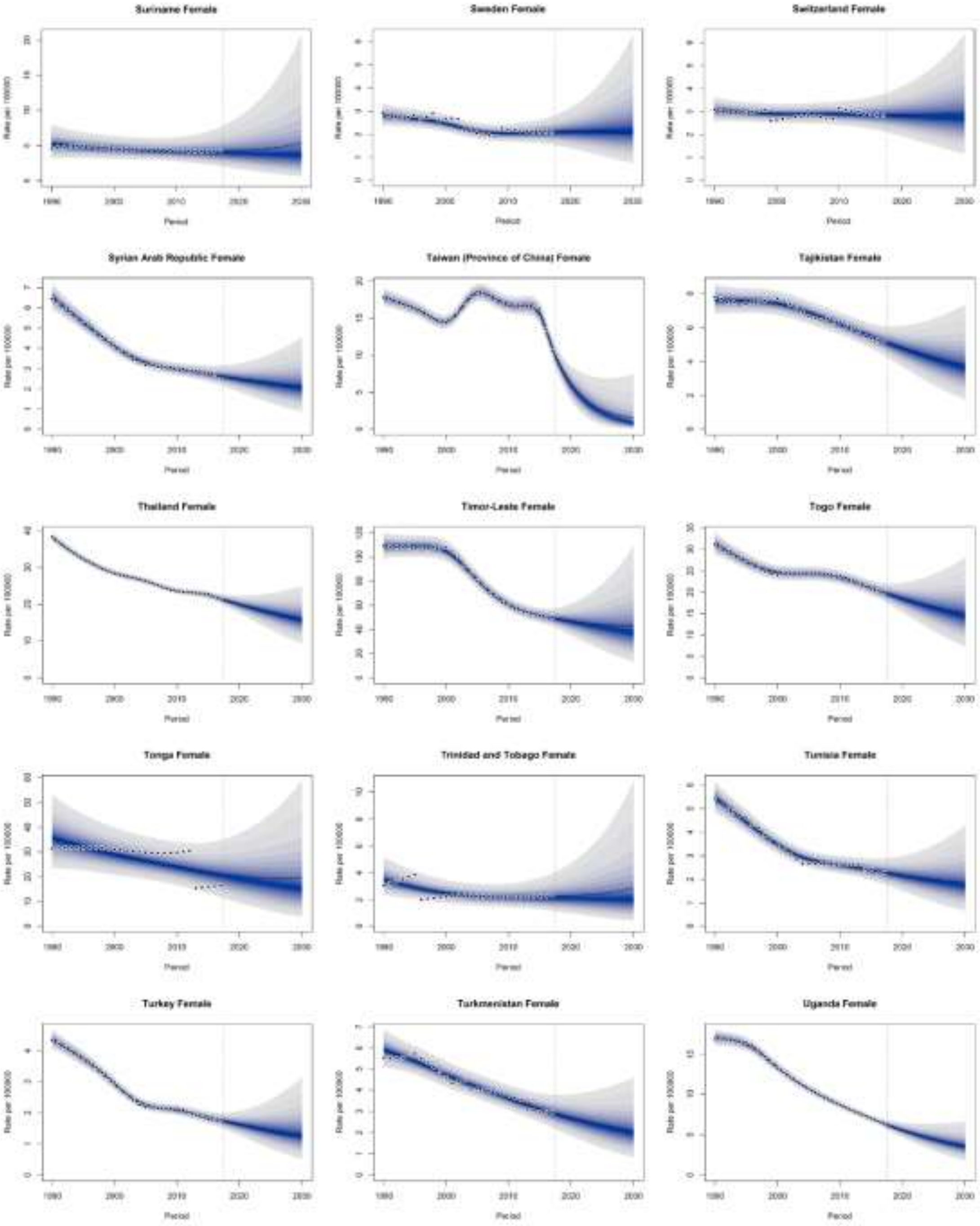

Suriname Female
Sweden Female
Switzerland Female
Syrian Arab Republic Female
Taiwan (Province of China) Female
Tajikistan Female
Thailand Female
Timor-Leste Female
Togo Female
Tonga Female
Trinidad and Tobago Female
Tunisia Female
Turkey Female
Turkmenistan Female
Uganda Female

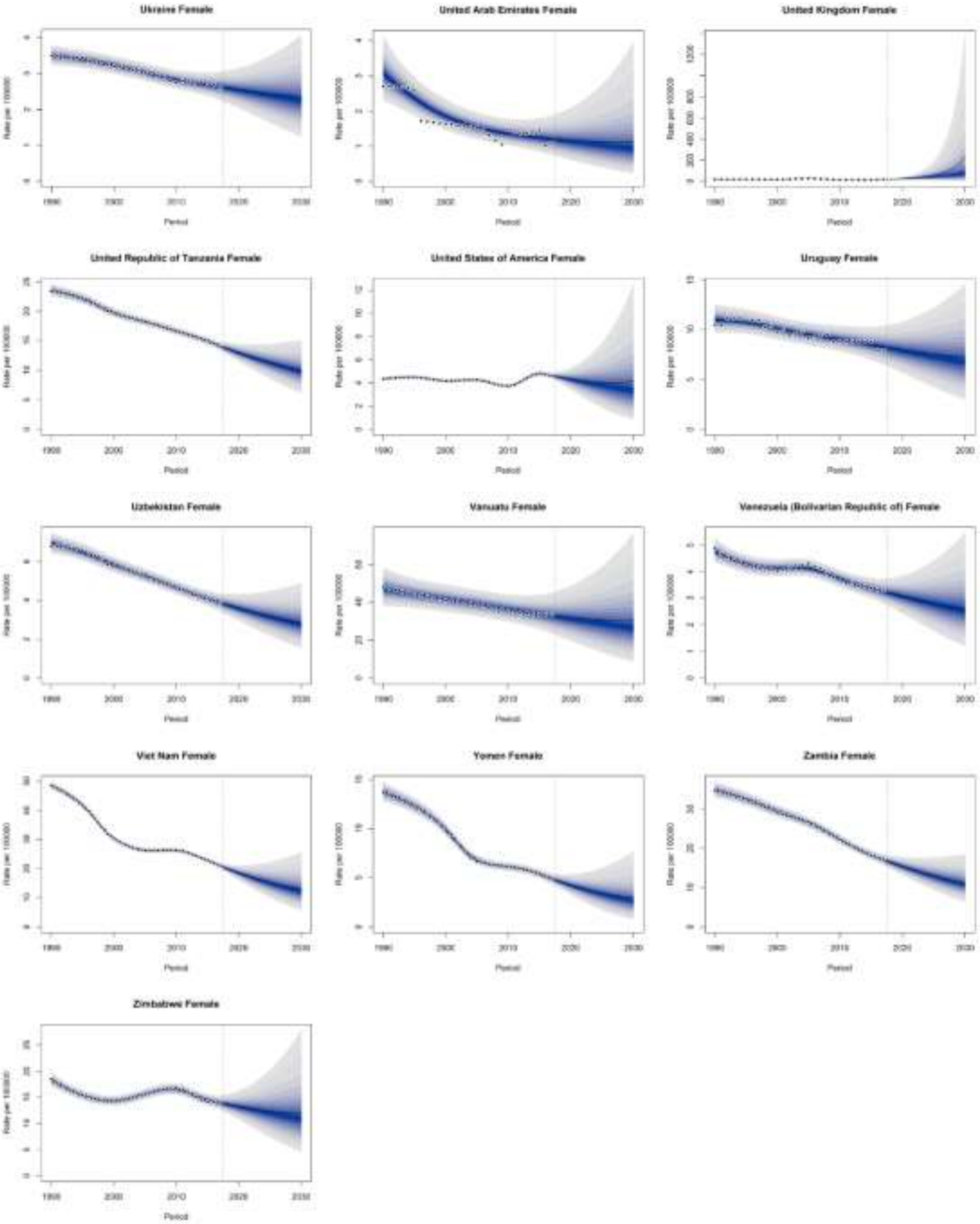

Figure S33. Trends in prevalence rates of 178 countries and territories from 1990 to 2030 for females by BAPC model.

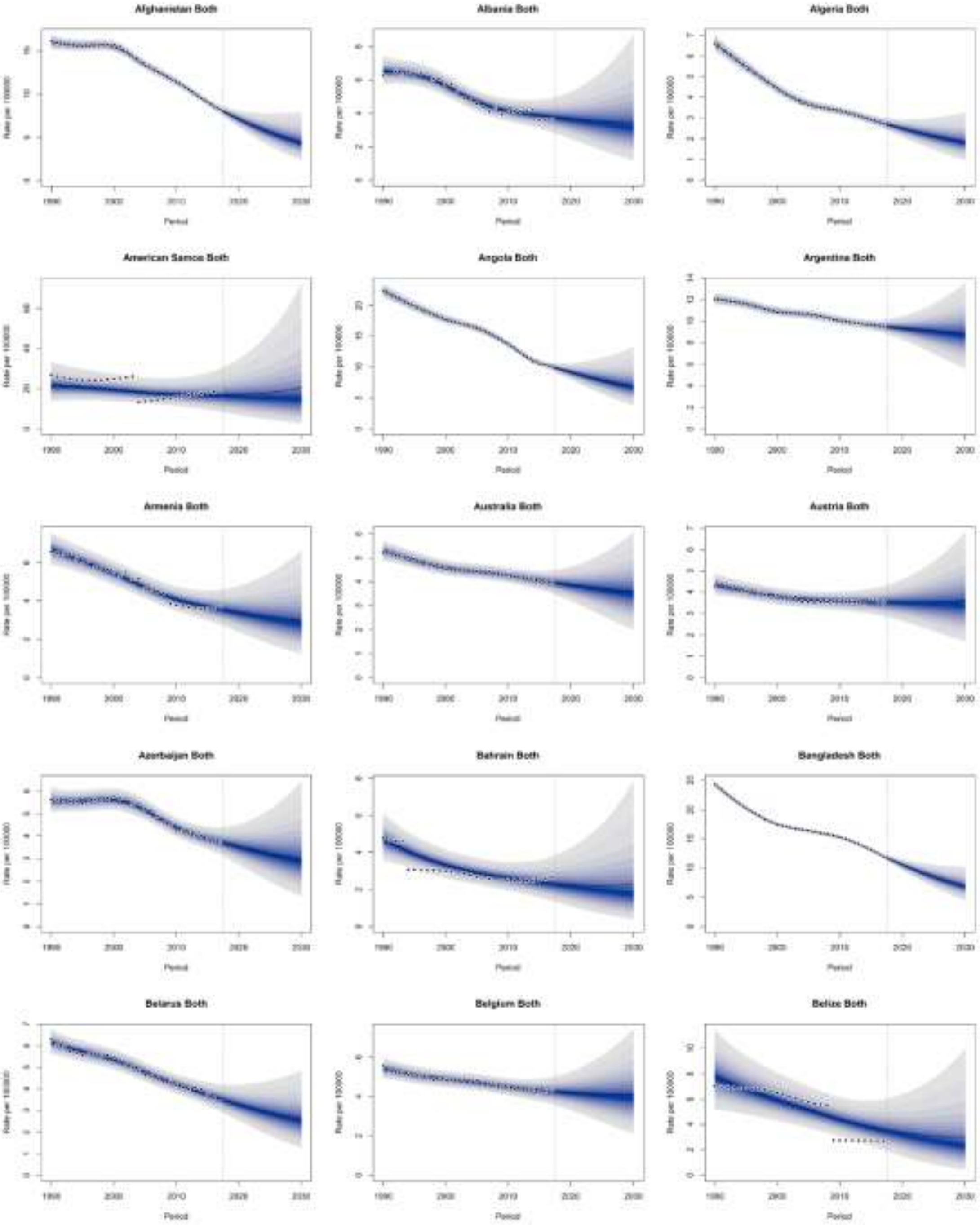
Afghanistan Both
Albania Both
Algeria Both
American Samoa Both
Angola Both
Argentina Both
Armenia Both
Australia Both
Austria Both
Azerbaijan Both
Bahrain Both
Bangladesh Both
Belarus Both
Belgium Both
Belize Both
Period
Rate per 100000

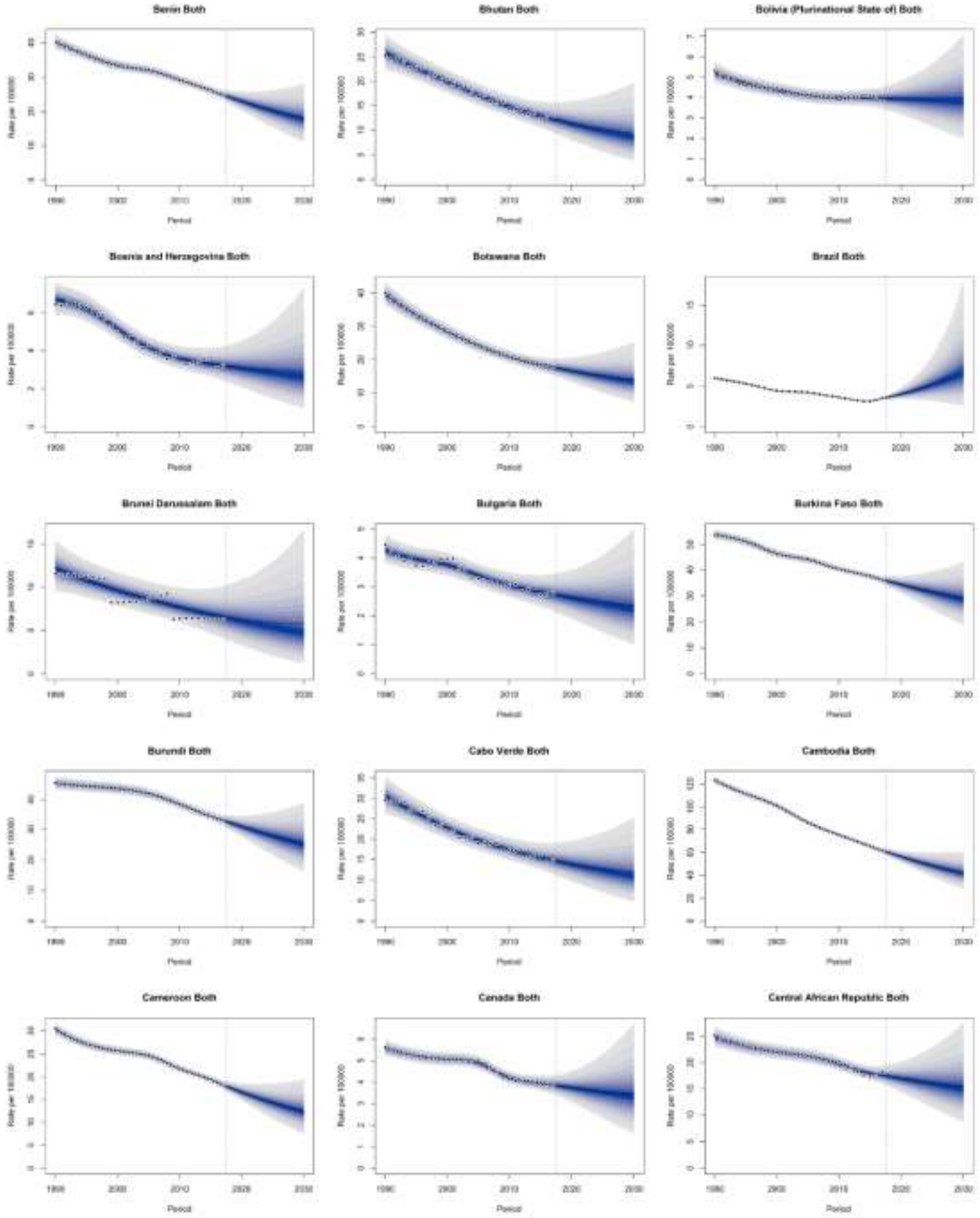
Benin Both
Bhutan Both
Bolivia (Plurinational State of) Both
Bosnia and Herzegovina Both
Botswana Both
Brazil Both
Brunei Darussalam Both
Bulgaria Both
Burkina Faso Both
Burundi Both
Cabo Verde Both
Cambodia Both
Cameroon Both
Canada Both
Central African Republic Both

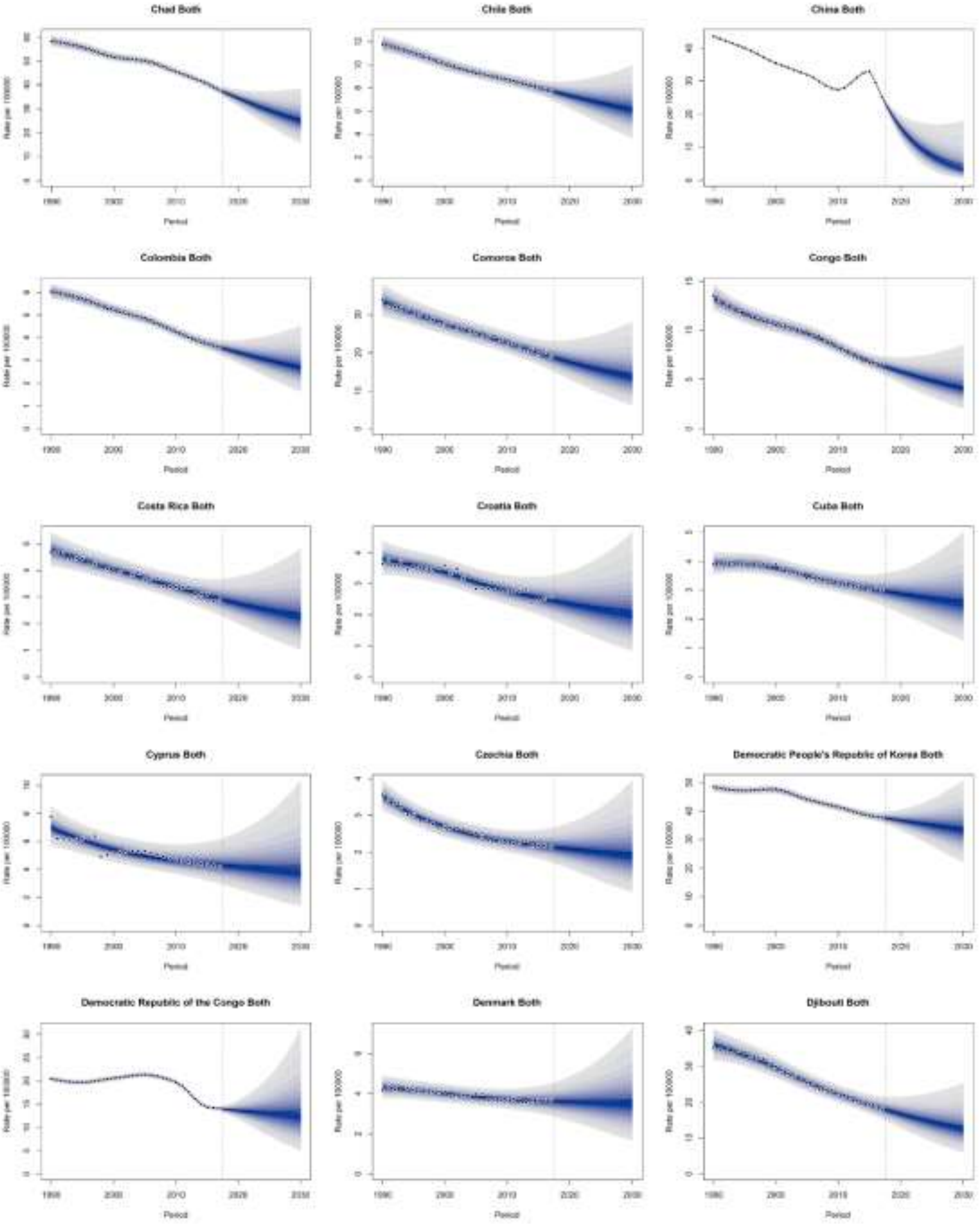
Chad Both
Chile Both
China Both
Colombia Both
Comoros Both
Congo Both
Costa Rica Both
Croatia Both
Cuba Both
Cyprus Both
Czechia Both
Democratic People's Republic of Korea Both
Democratic Republic of the Congo Both
Denmark Both
Djibouti Both

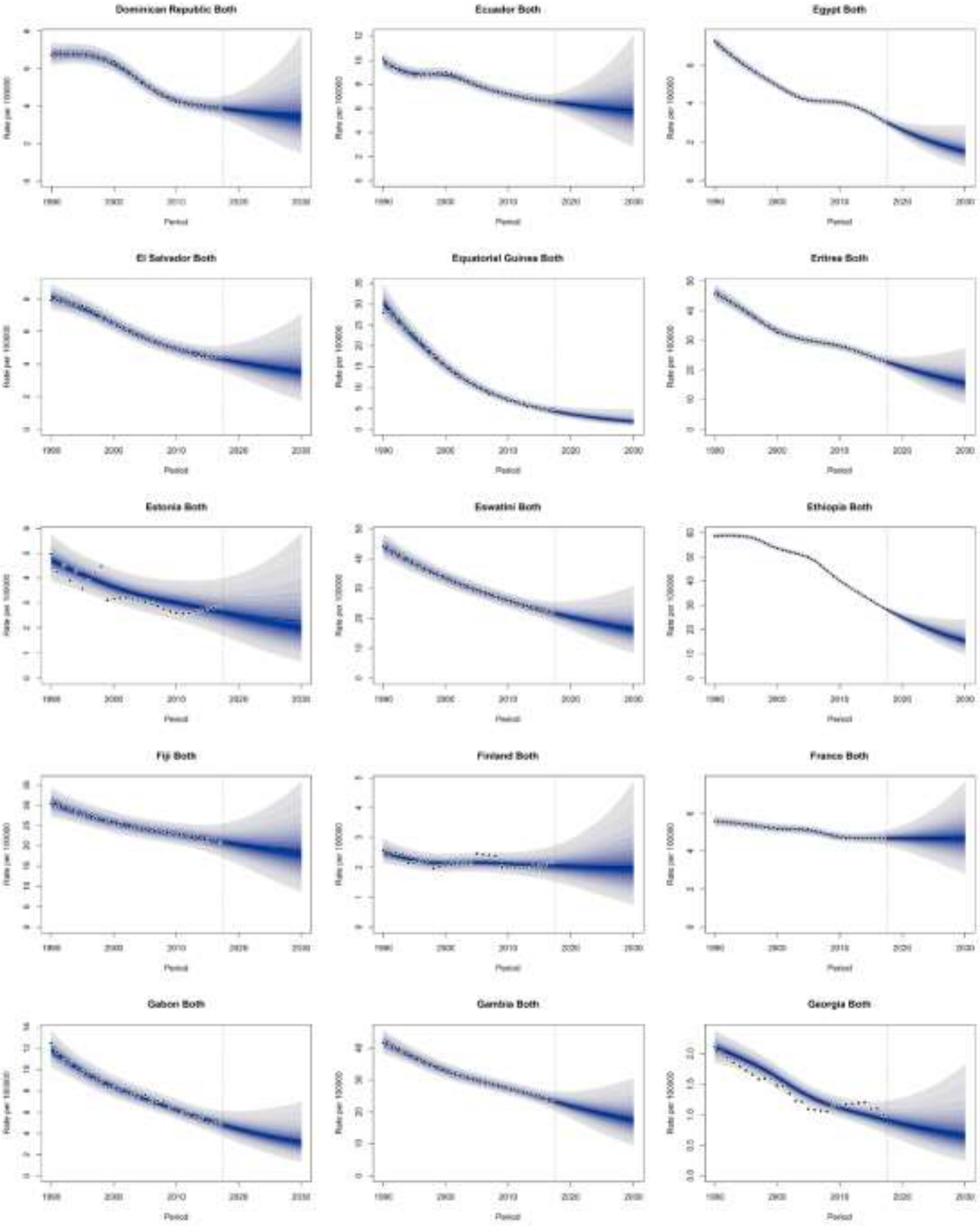
Dominican Republic Both
Ecuador Both
Egypt Both
El Salvador Both
Equatorial Guinea Both
Eritrea Both
Estonia Both
Eswatini Both
Ethiopia Both
Fiji Both
Finland Both
France Both
Gabon Both
Gambia Both
Georgia Both

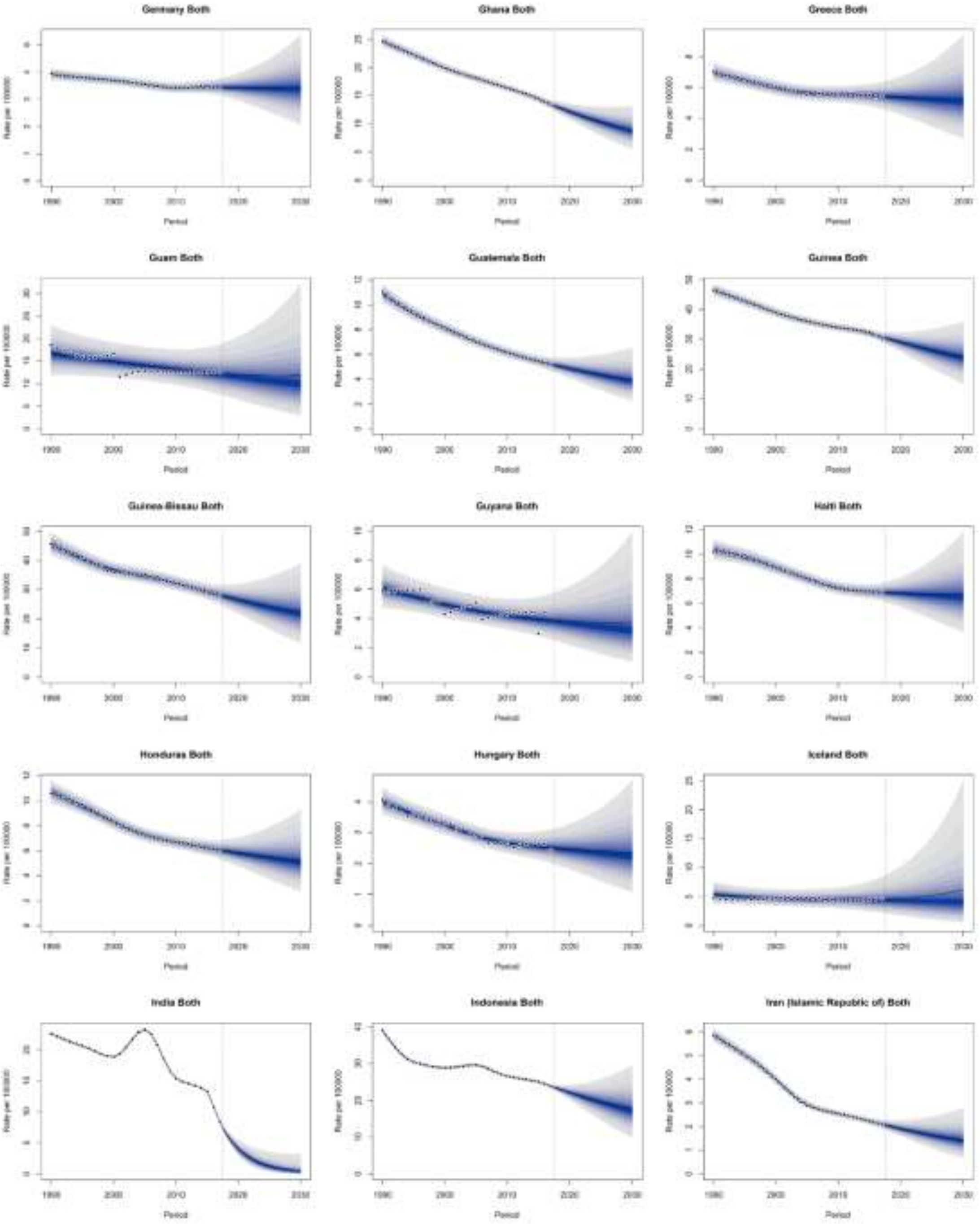
Germany Both
Ghana Both
Greece Both
Guam Both
Guatemala Both
Guinea Both
Guinea-Bissau Both
Guyana Both
Haiti Both
Honduras Both
Hungary Both
Iceland Both
India Both
Indonesia Both
Iran (Islamic Republic of) Both
Period

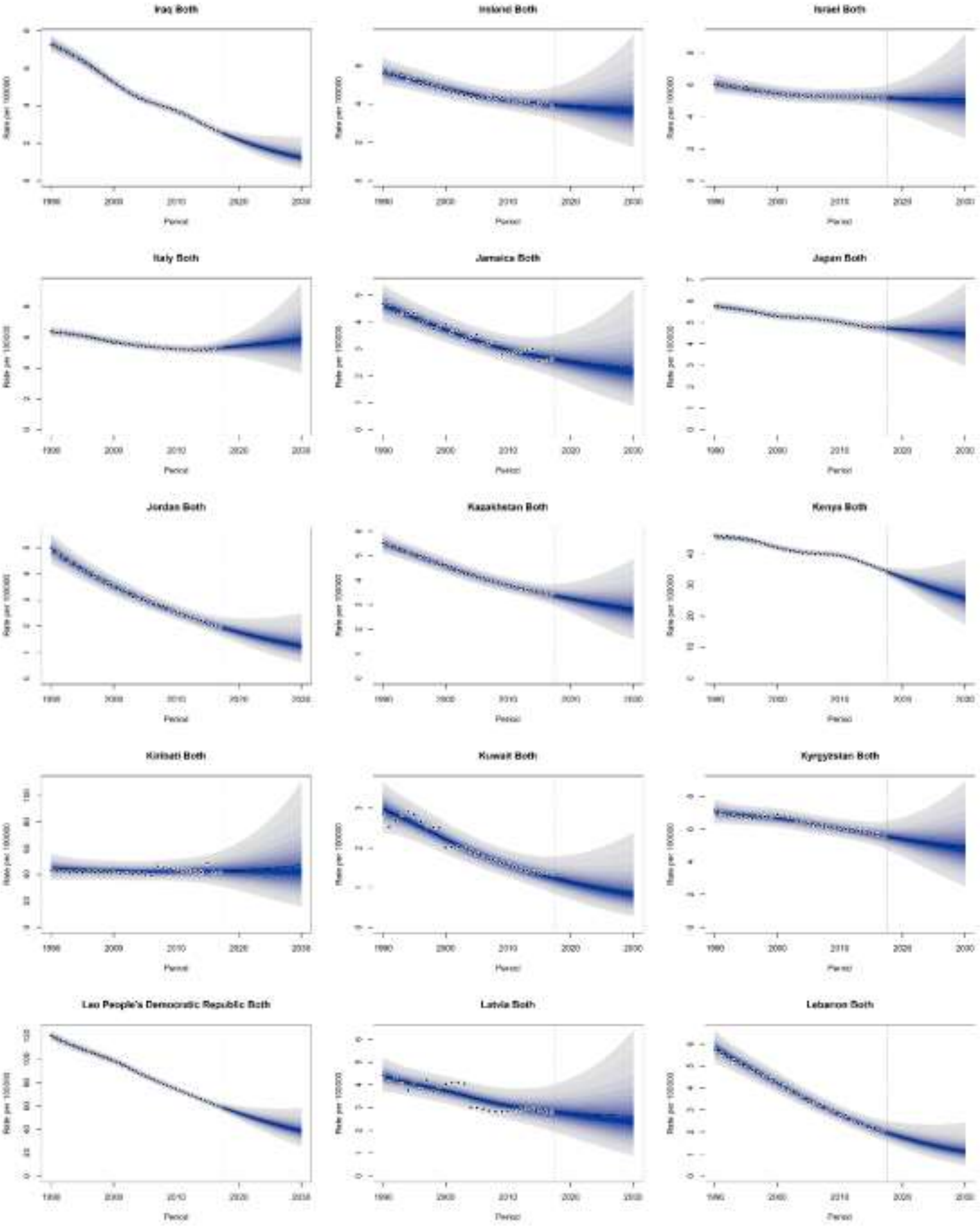
Iraq Both
Ireland Both
Israel Both
Italy Both
Jamaica Both
Japan Both
Jordan Both
Kazakhstan Both
Kenya Both
Kiribati Both
Kuwait Both
Kyrgyzstan Both
Lao People's Democratic Republic Both
Latvia Both
Lebanon Both

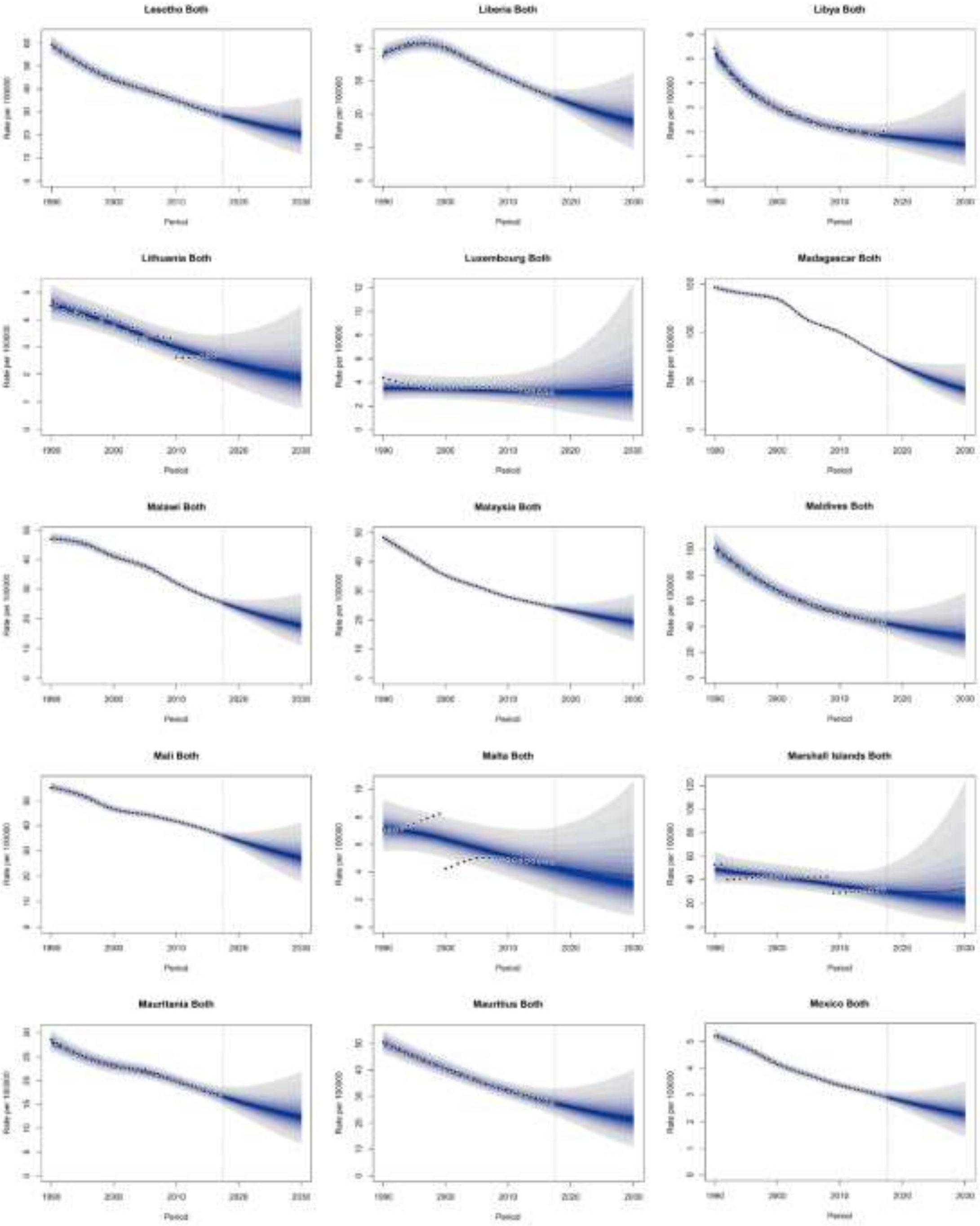
Lesotho Both
Liberia Both
Libya Both
Lithuania Both
Luxembourg Both
Madagascar Both
Malawi Both
Malaysia Both
Maldives Both
Mali Both
Malta Both
Marshall Islands Both
Mauritania Both
Mauritius Both
Mexico Both

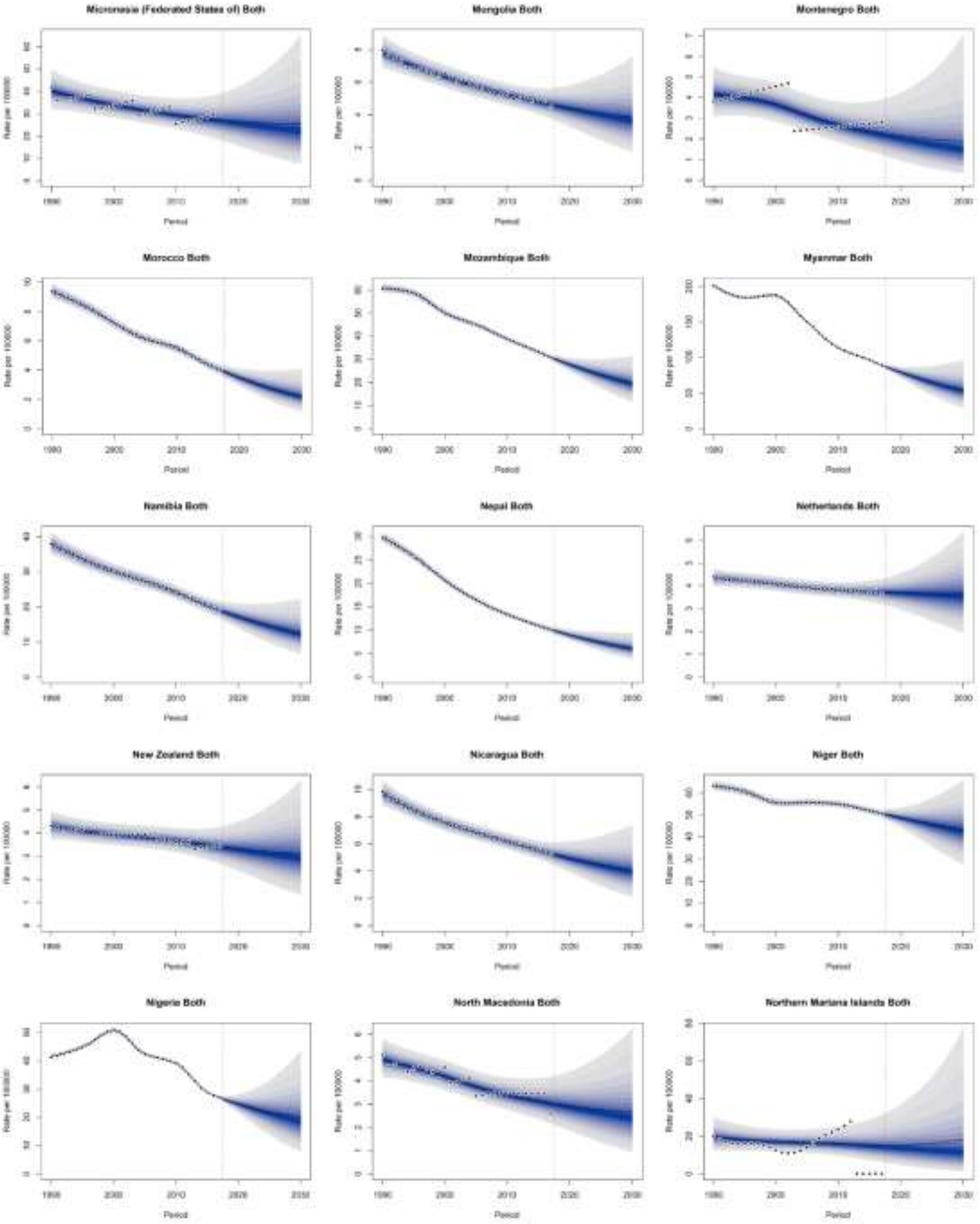
Micronesia (Federated States of) Both
Mongolia Both
Montenegro Both
Morocco Both
Mozambique Both
Myanmar Both
Namibia Both
Nepal Both
Netherlands Both
New Zealand Both
Nicaragua Both
Niger Both
Nigeria Both
North Macedonia Both
Northern Mariana Islands Both

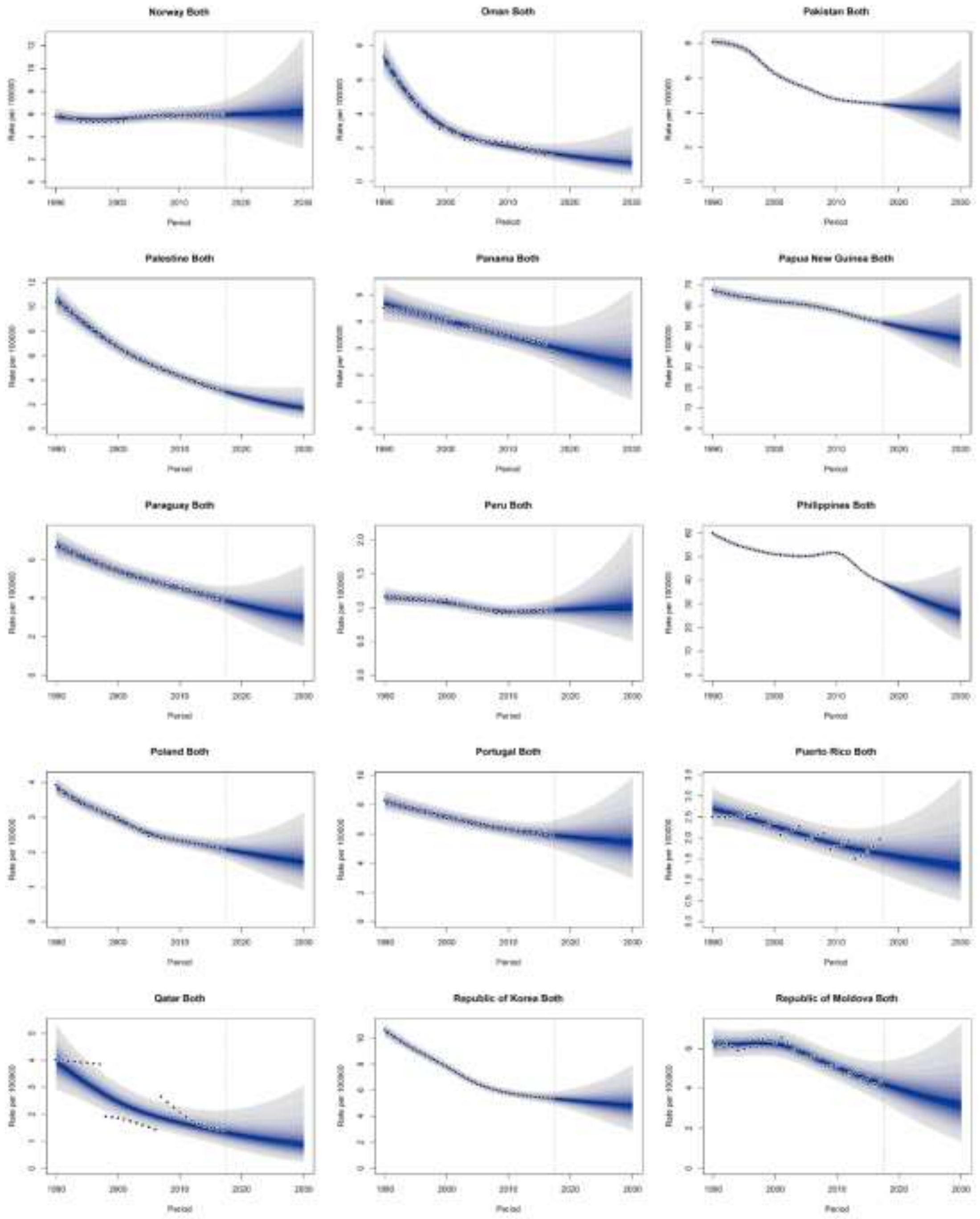
Norway Both
Oman Both
Pakistan Both
Palestine Both
Panama Both
Papua New Guinea Both
Paraguay Both
Peru Both
Philippines Both
Poland Both
Portugal Both
Puerto Rico Both
Qatar Both
Republic of Korea Both
Republic of Moldova Both
Rate per 100000
Period

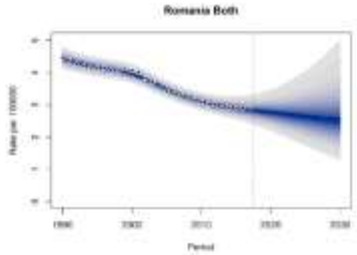
Romania Both
Rate per 100000
Period

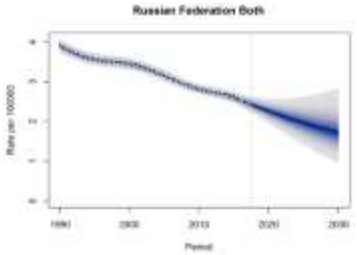
Russian Federation Both
Rate per 100000
Period

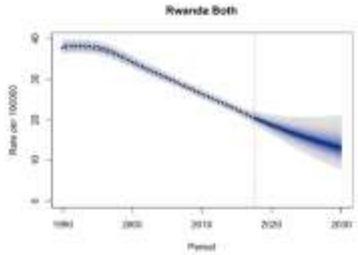
Rwanda Both
Rate per 100000
Period

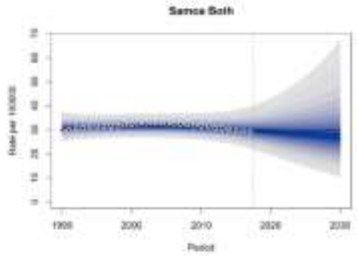
Samoa Both
Rate per 100000
Period

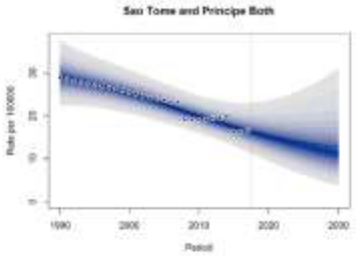
Sao Tome and Principe Both
Rate per 100000
Period

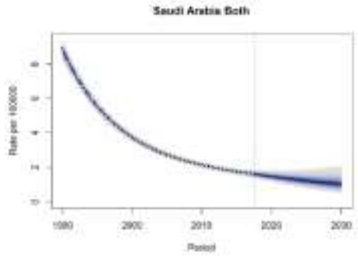
Saudi Arabia Both
Rate per 100000
Period

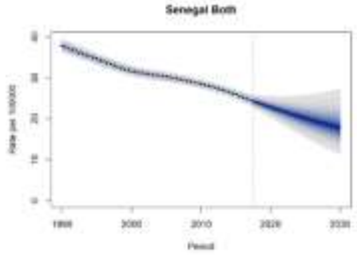
Senegal Both
Rate per 100000
Period

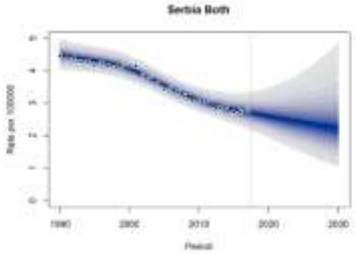
Serbia Both
Rate per 100000
Period

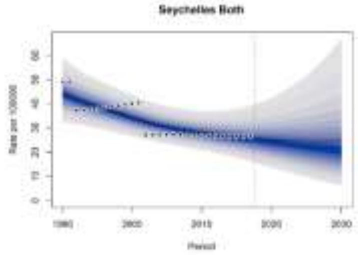
Seychelles Both
Rate per 100000
Period

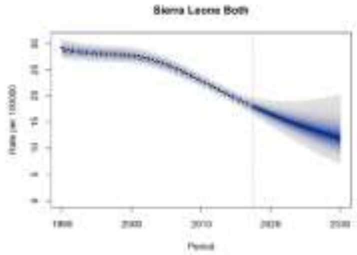
Sierra Leone Both
Rate per 100000
Period

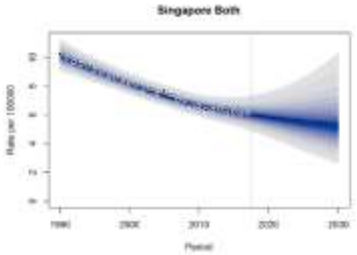
Singapore Both
Rate per 100000
Period

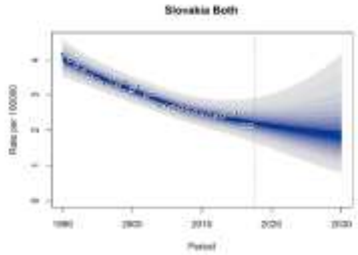
Slovakia Both
Rate per 100000
Period

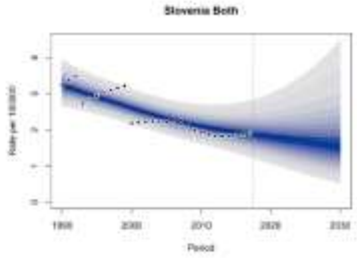
Slovenia Both
Rate per 100000
Period

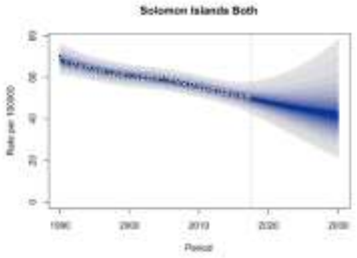
Solomon Islands Both
Rate per 100000
Period

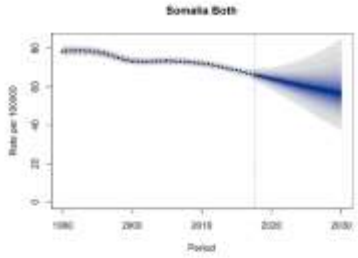
Somalia Both
Rate per 100000
Period

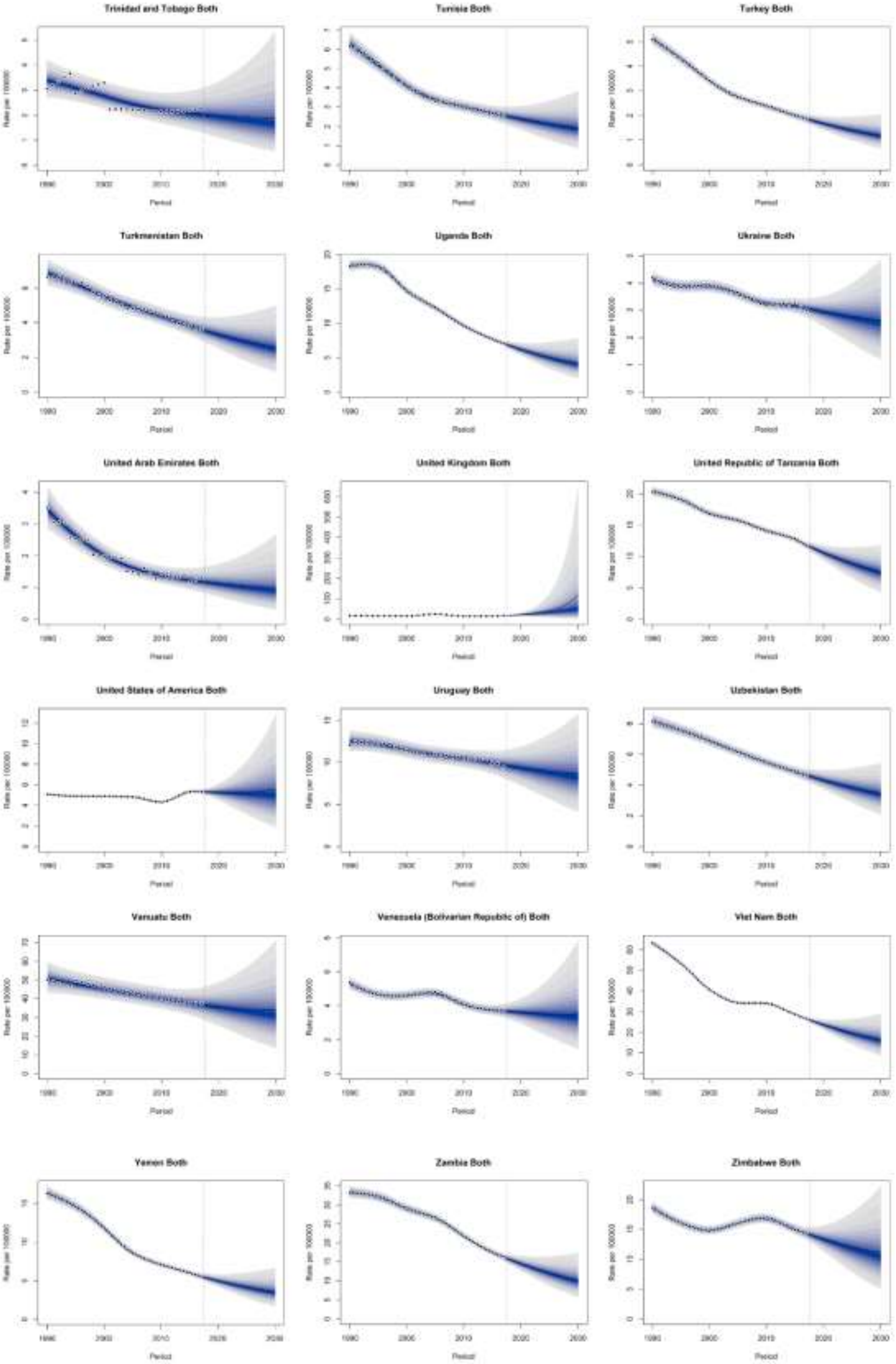

Figure S34. Trends in prevalence rates of 183 countries and territories from 1990 to 2030 for both sexes by BAPC model.

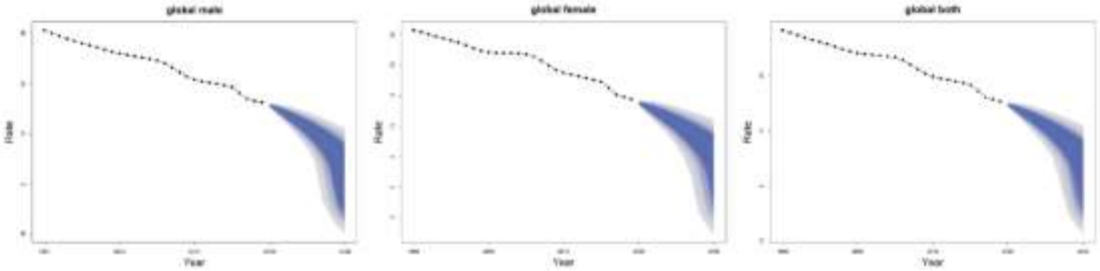

Figure S35. Global trends in prevalence rates from 1990 to 2030 for males, females and both sexes by ARIMA model.

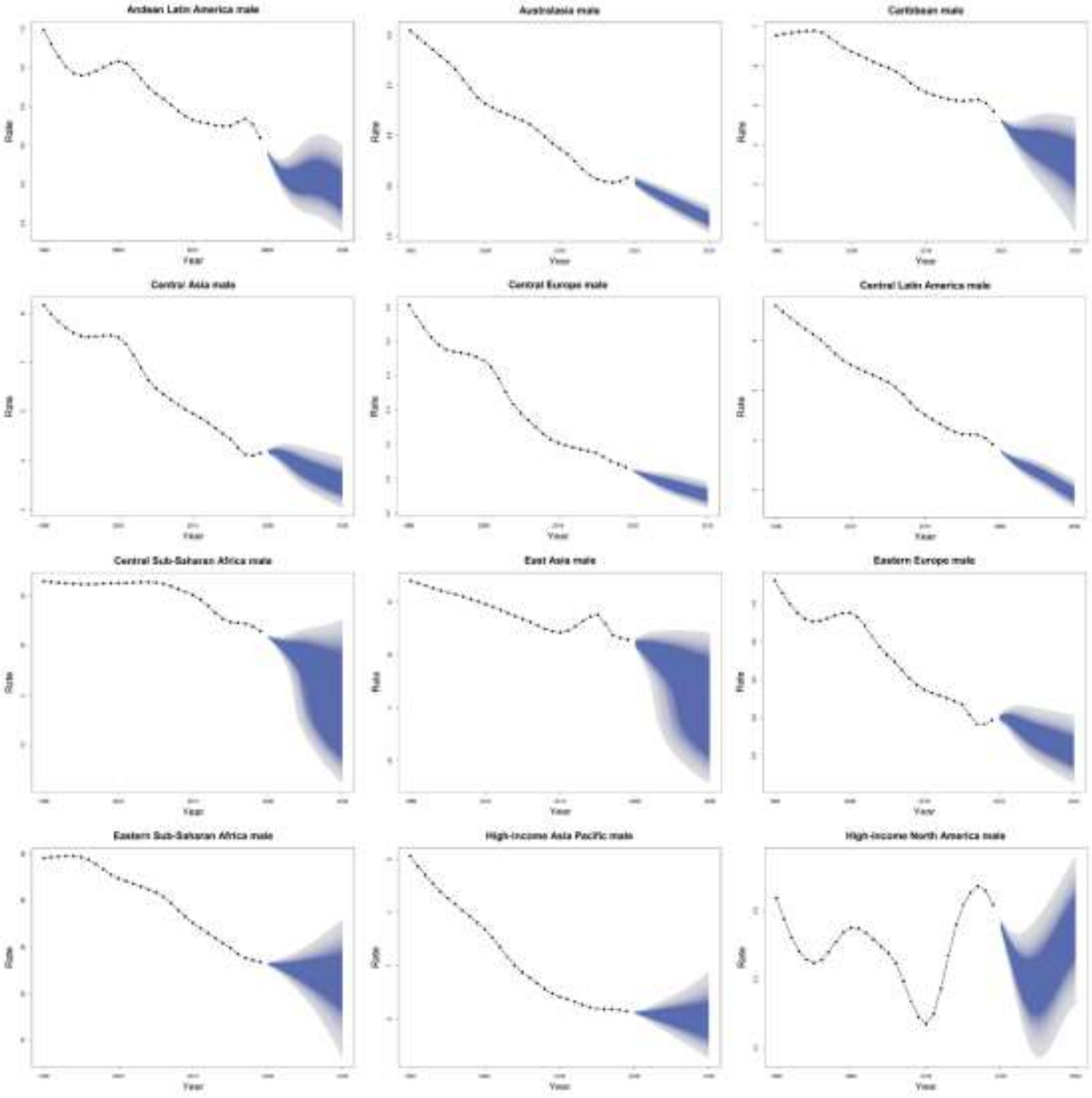
Andean Latin America male
Australasia male
Caribbean male
Central Asia male
Central Europe male
Central Latin America male
Central Sub-Saharan Africa male
East Asia male
Eastern Europe male
Eastern Sub-Saharan Africa male
High-income Asia Pacific male
High-income North America male
Rate
Year

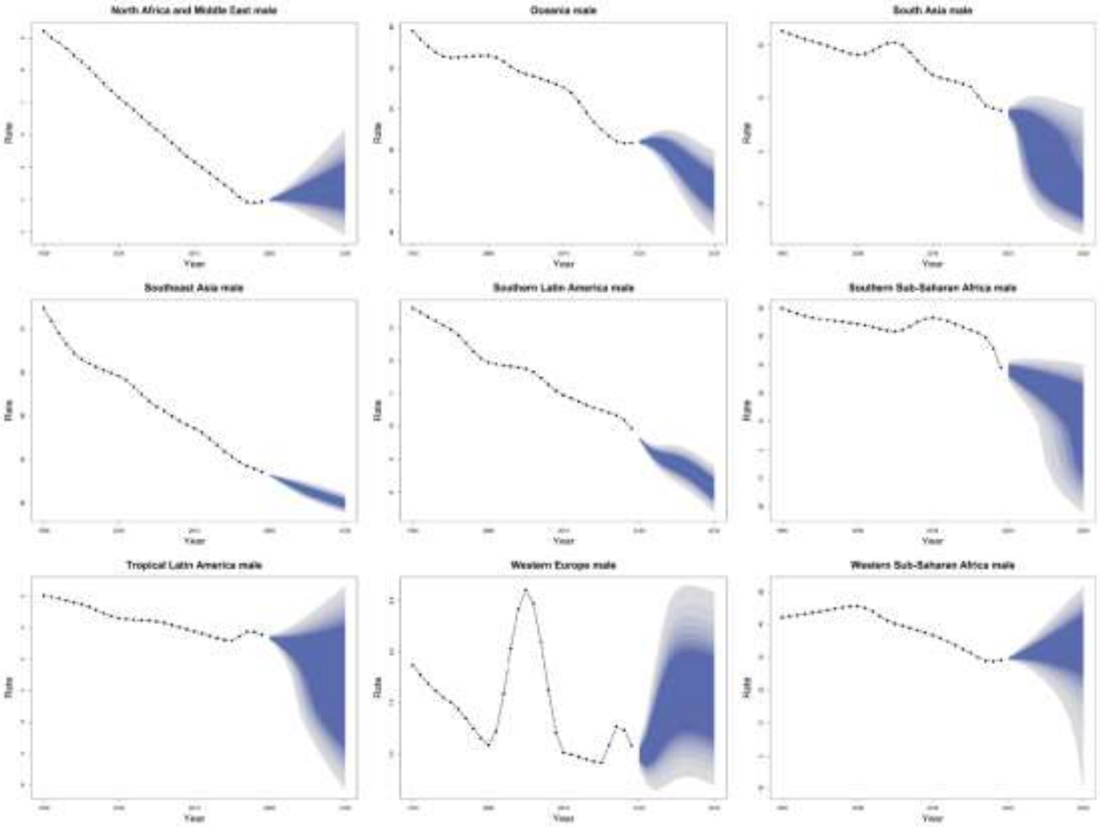

Figure S36. Trends in prevalence rates of 21 GBD regions from 1990 to 2030 for males by ARIMA model.

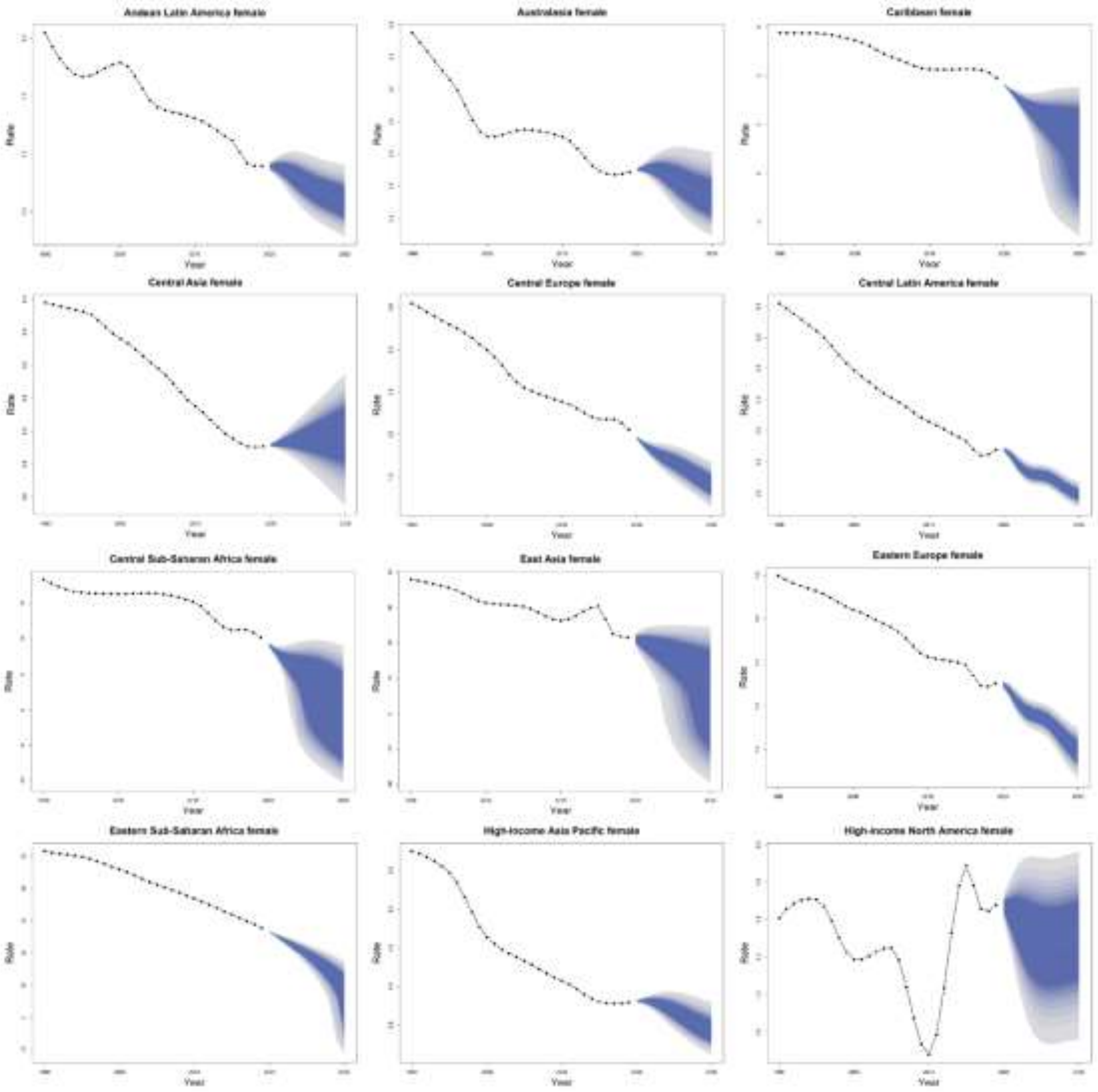
Andean Latin America female
Australasia female
Caribbean female
Central Asia female
Central Europe female
Central Latin America female
Central Sub-Saharan Africa female
East Asia female
Eastern Europe female
Eastern Sub-Saharan Africa female
High-income Asia Pacific female
High-income North America female
Rate
Year

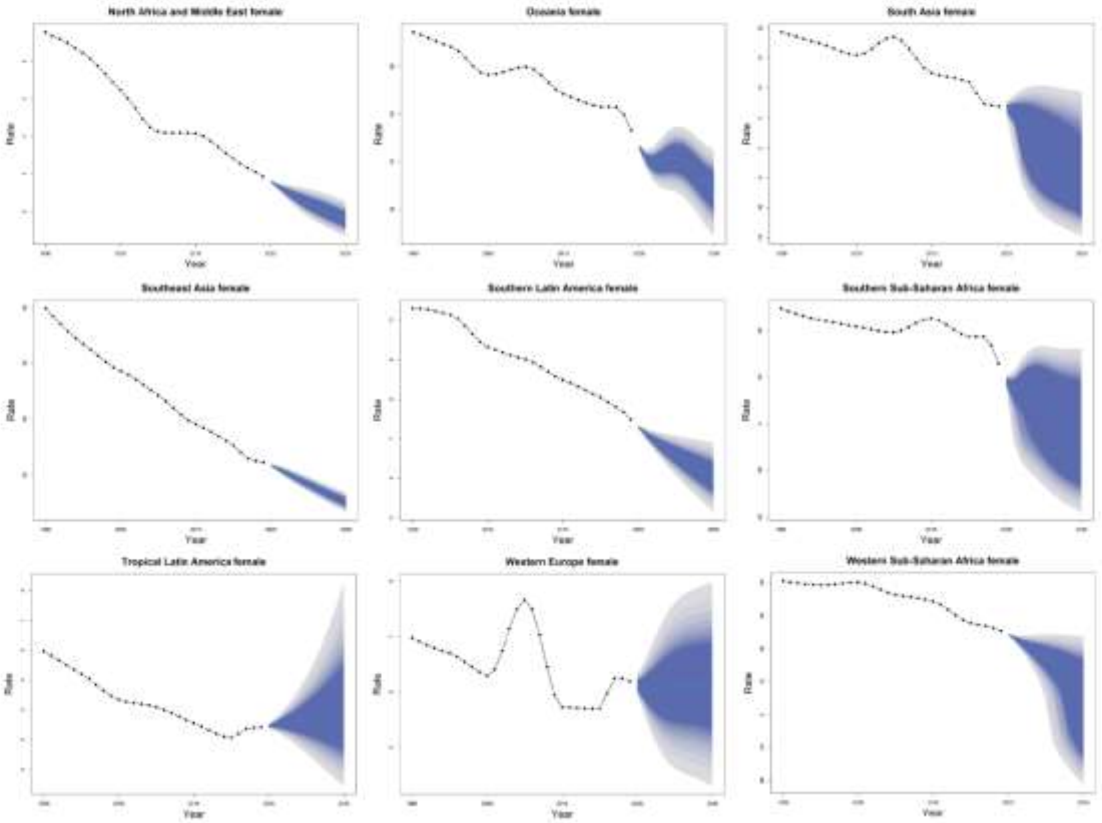

Figure S37. Trends in prevalence rates of 21 GBD regions from 1990 to 2030 for females by ARIMA model.

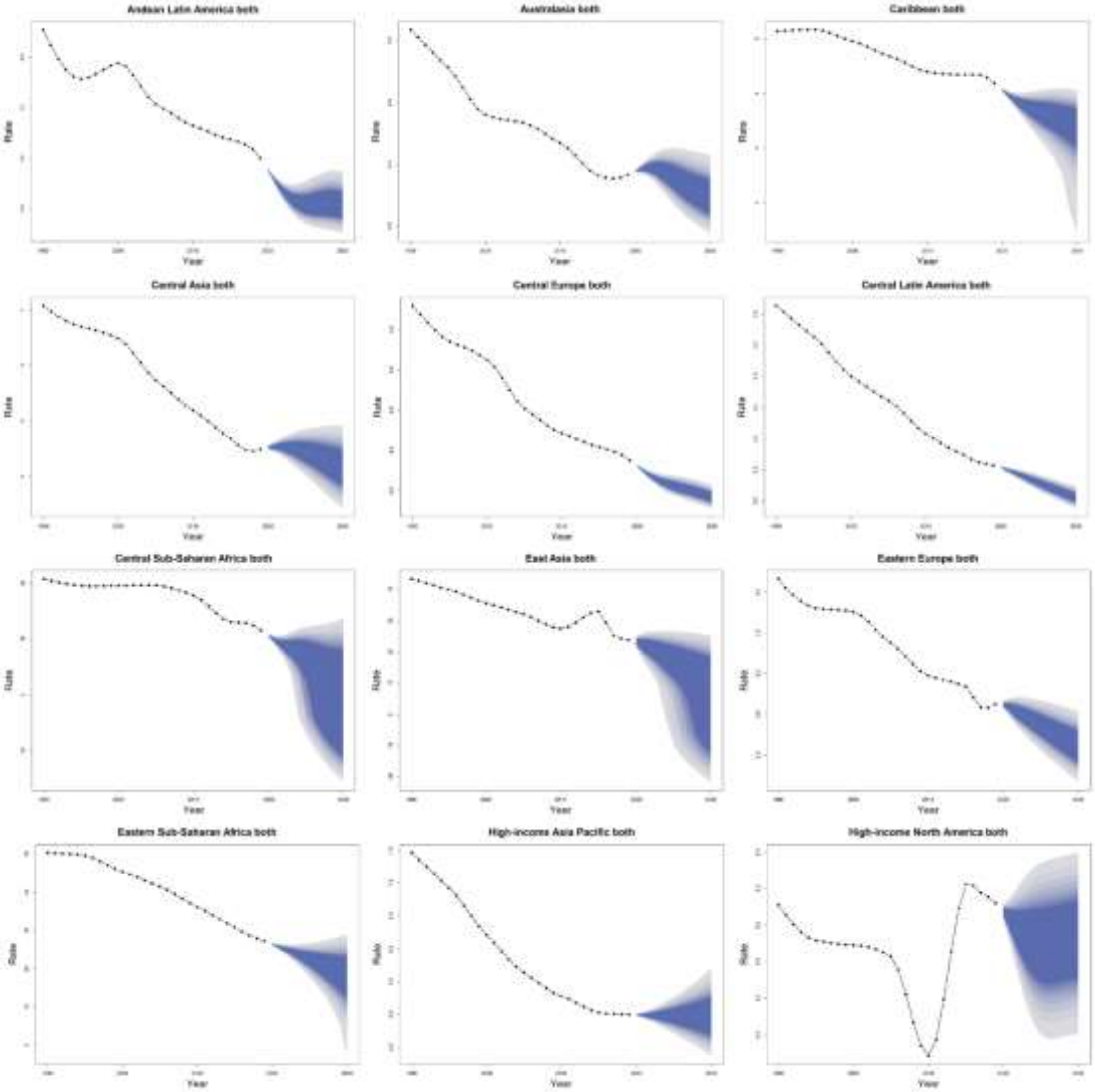

Andean Latin America both
Australasia both
Caribbean both
Central Asia both
Central Europe both
Central Latin America both
Central Sub-Saharan Africa both
East Asia both
Eastern Europe both
Eastern Sub-Saharan Africa both
High-income Asia Pacific both
High-income North America both
Rate
Year

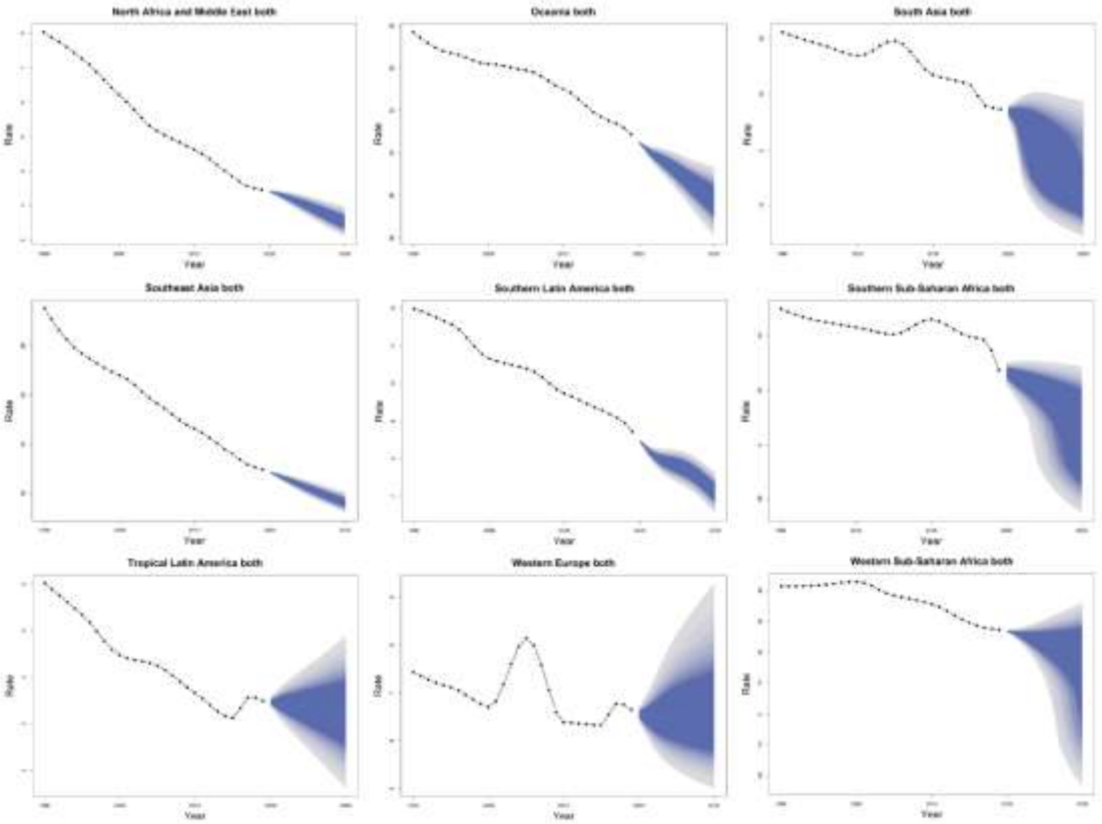

Figure S38. Trends in prevalence rates of 21 GBD regions from 1990 to 2030 for both sexes by ARIMA model.

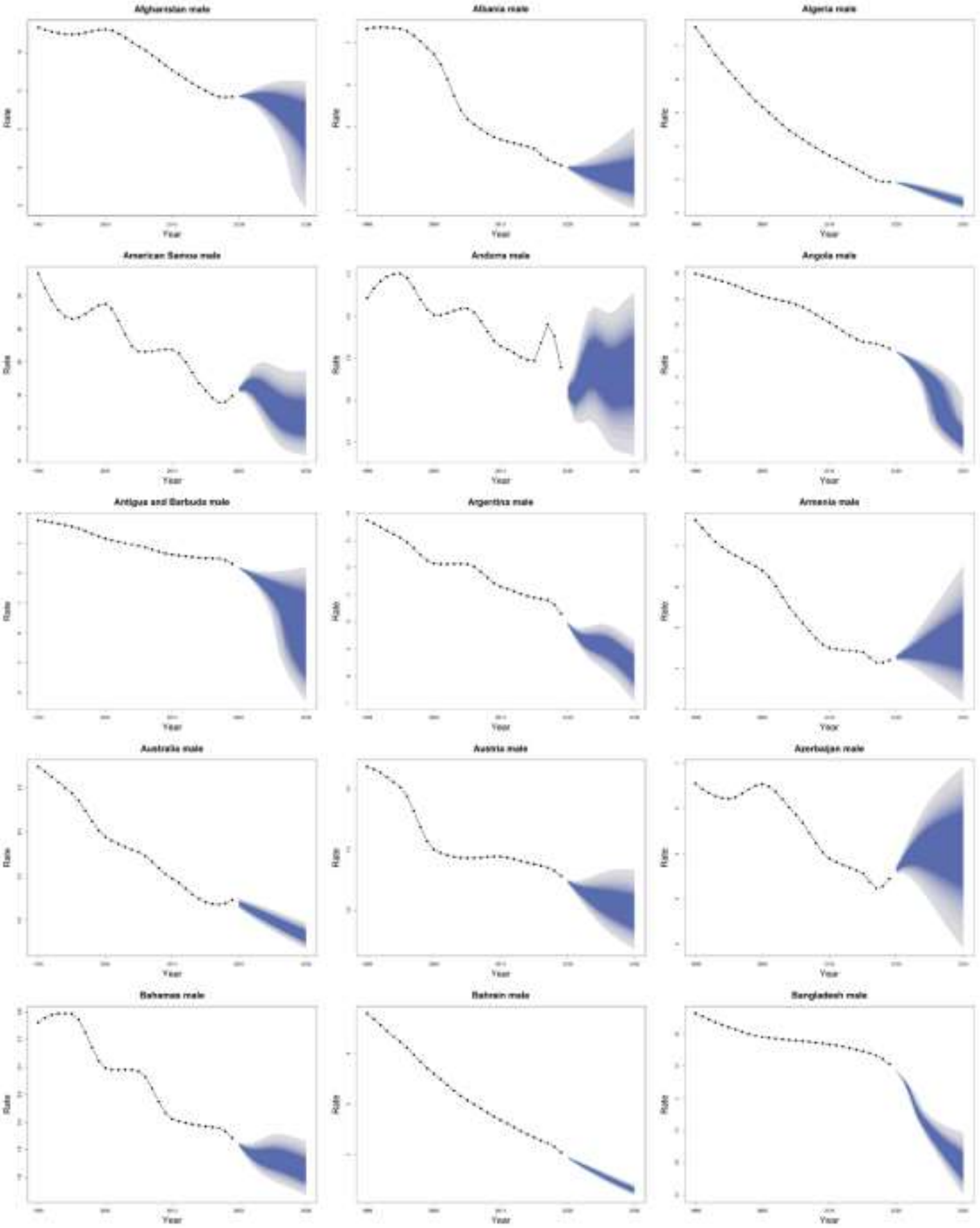
Afghanistan male
Albania male
Algeria male
American Samoa male
Andorra male
Angola male
Antigua and Barbuda male
Argentina male
Armenia male
Australia male
Austria male
Azerbaijan male
Bahamas male
Bahrain male
Bangladesh male
Rate
Year

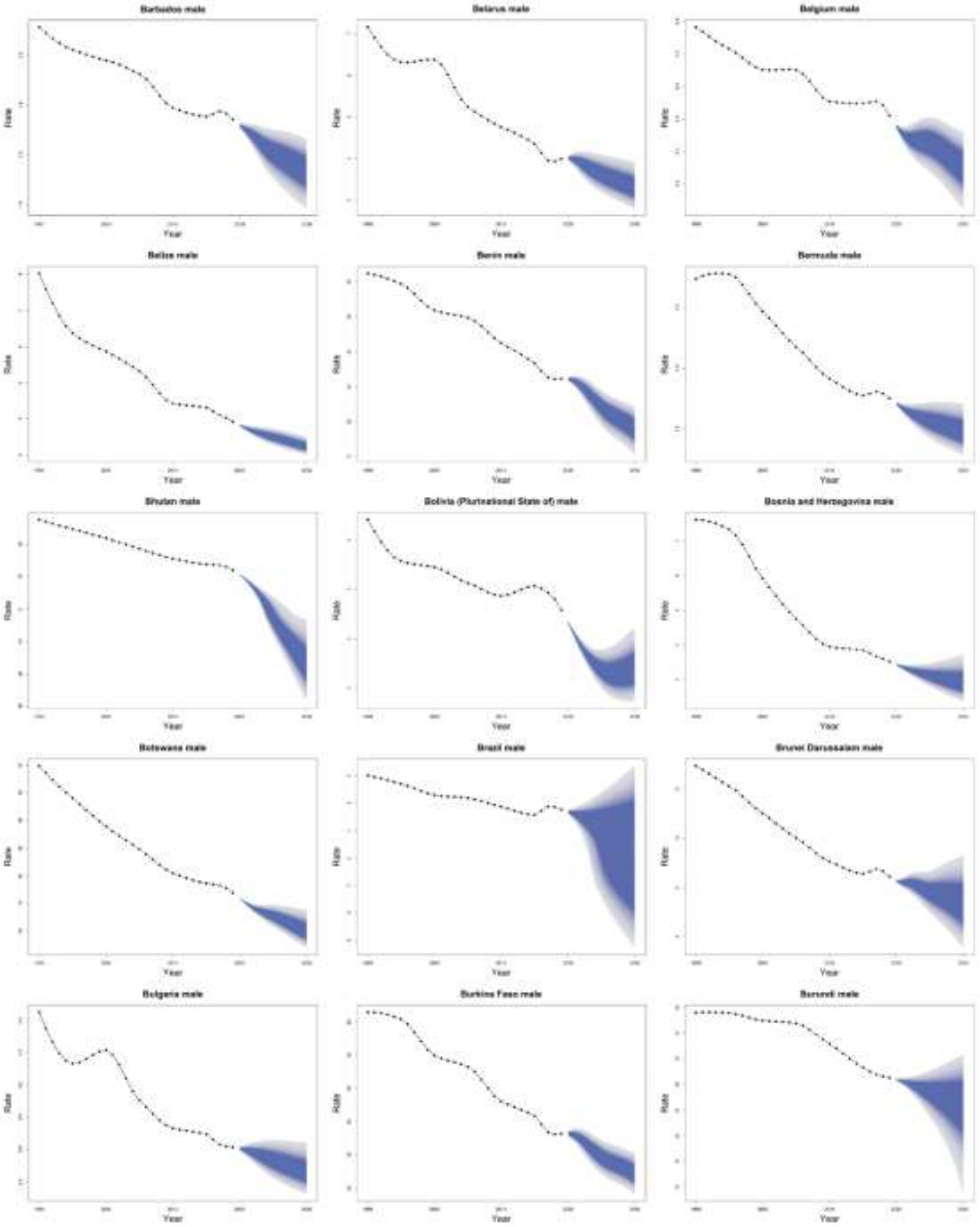

Barbados male
Belarus male
Belgium male
Belize male
Benin male
Bermuda male
Bhutan male
Bolivia (Plurinational State of) male
Bosnia and Herzegovina male
Botswana male
Brazil male
Brunei Darussalam male
Bulgaria male
Burkina Faso male
Burundi male
Rate
Year

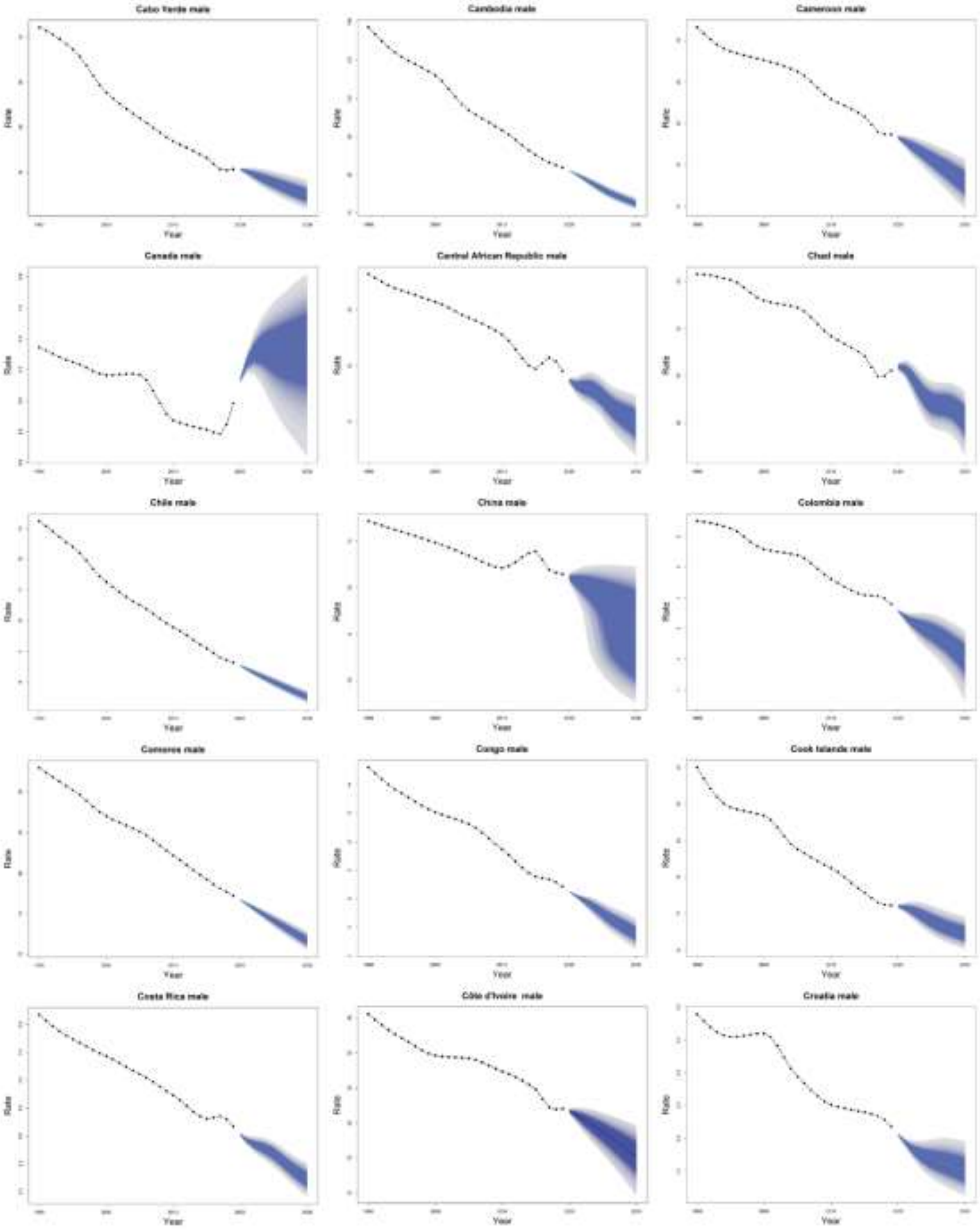
Cabo Verde male
Cambodia male
Cameroon male
Canada male
Central African Republic male
Chad male
Chile male
China male
Colombia male
Comoros male
Congo male
Cook Islands male
Costa Rica male
Côte d'Ivoire male
Croatia male
Rate
Year

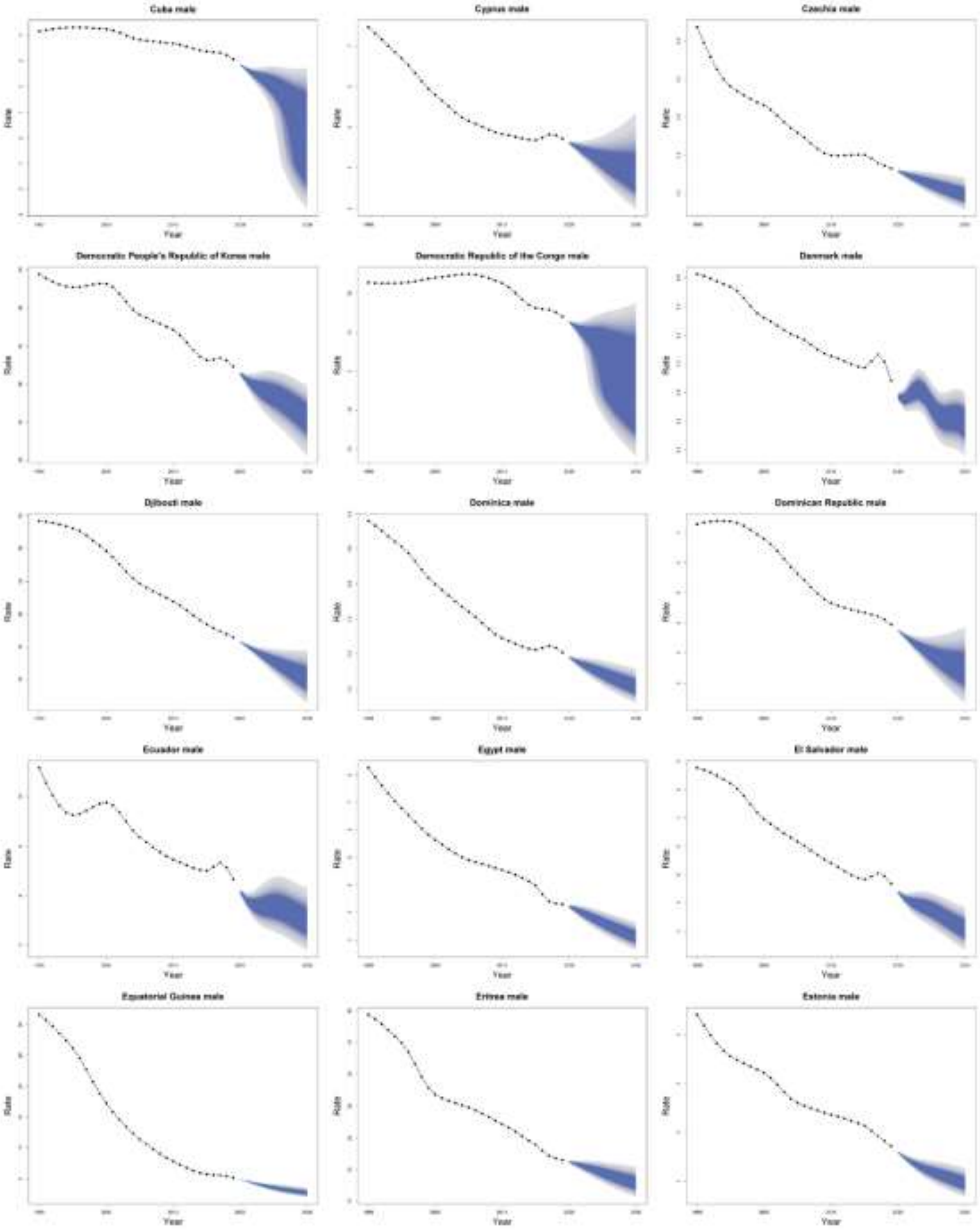
Cuba male
Cyprus male
Czechia male
Democratic People's Republic of Korea male
Democratic Republic of the Congo male
Denmark male
Djibouti male
Dominica male
Dominican Republic male
Ecuador male
Egypt male
El Salvador male
Equatorial Guinea male
Eritrea male
Estonia male
Rate
Year

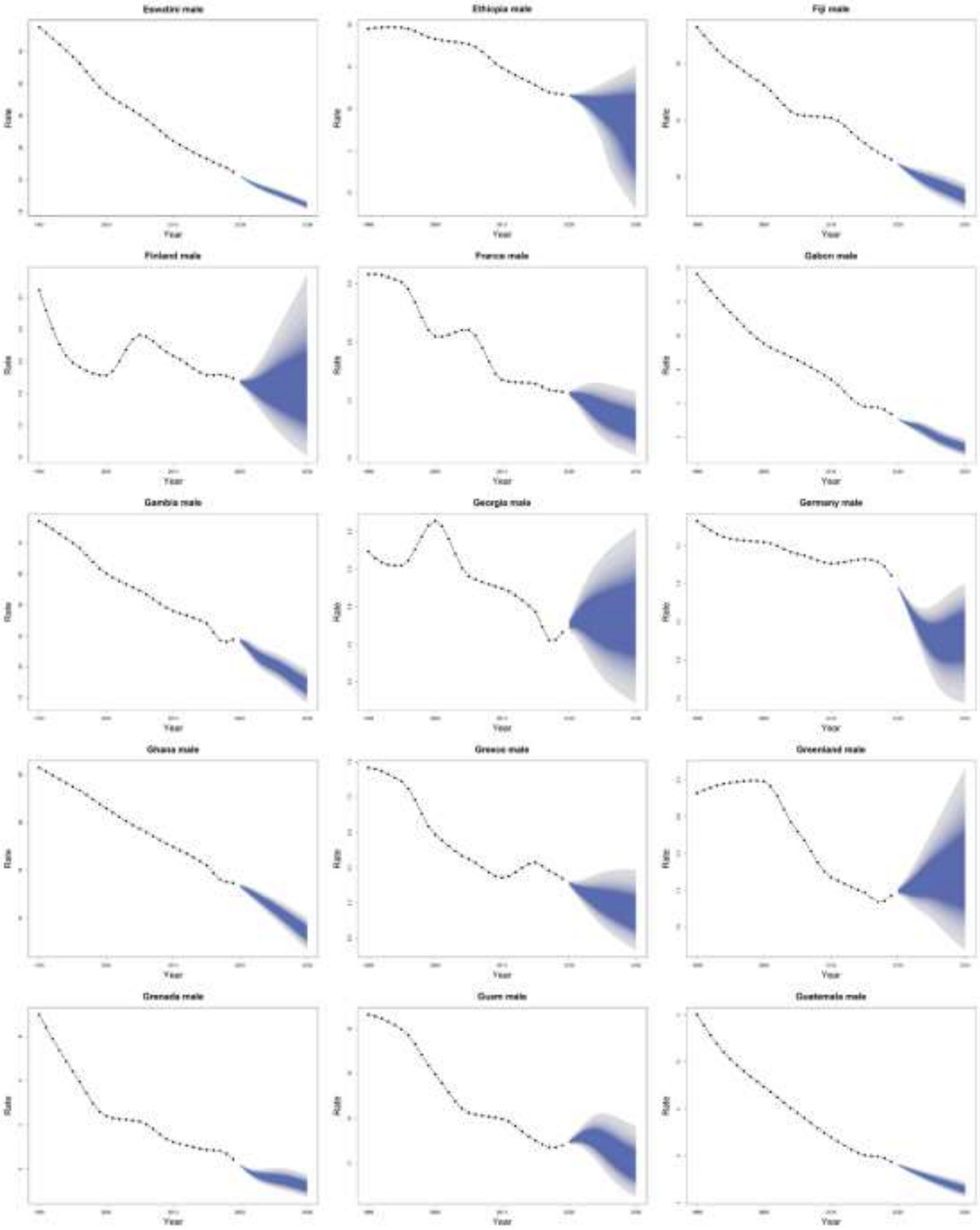
Eswatini male
Ethiopia male
Fiji male
Finland male
France male
Gabon male
Gambia male
Georgia male
Germany male
Ghana male
Greece male
Greenland male
Grenada male
Guam male
Guatemala male
Rate
Year

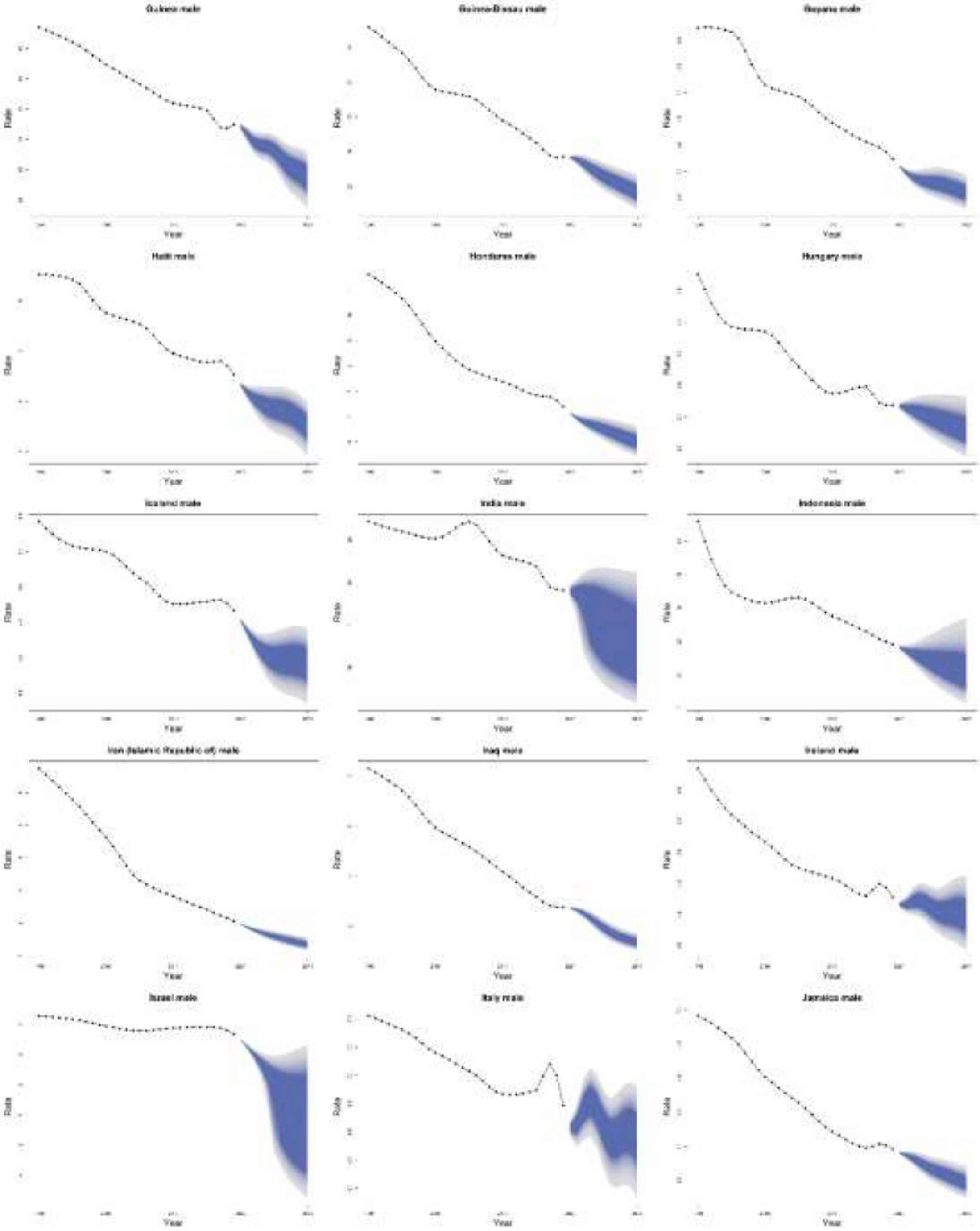
Guinea male
Guinea-Bissau male
Guyana male
Haiti male
Honduras male
Hungary male
Iceland male
India male
Indonesia male
Iran (Islamic Republic of) male
Iraq male
Ireland male
Israel male
Italy male
Jamaica male
Rate
Year

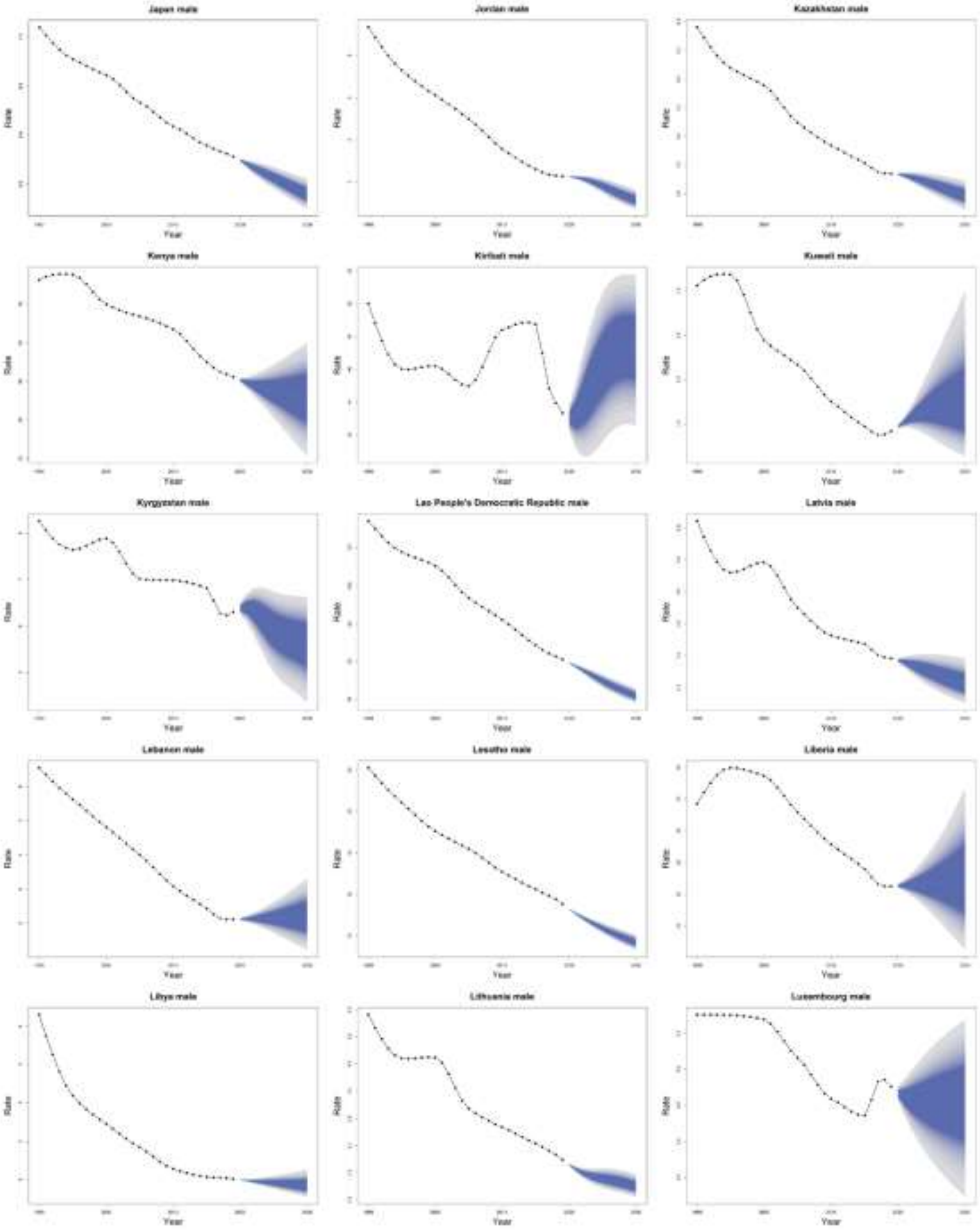

Japan male
Jordan male
Kazakhstan male
Kenya male
Kiribati male
Kuwait male
Kyrgyzstan male
Lao People's Democratic Republic male
Latvia male
Lebanon male
Lesotho male
Liberia male
Libya male
Lithuania male
Luxembourg male
Rate
Year

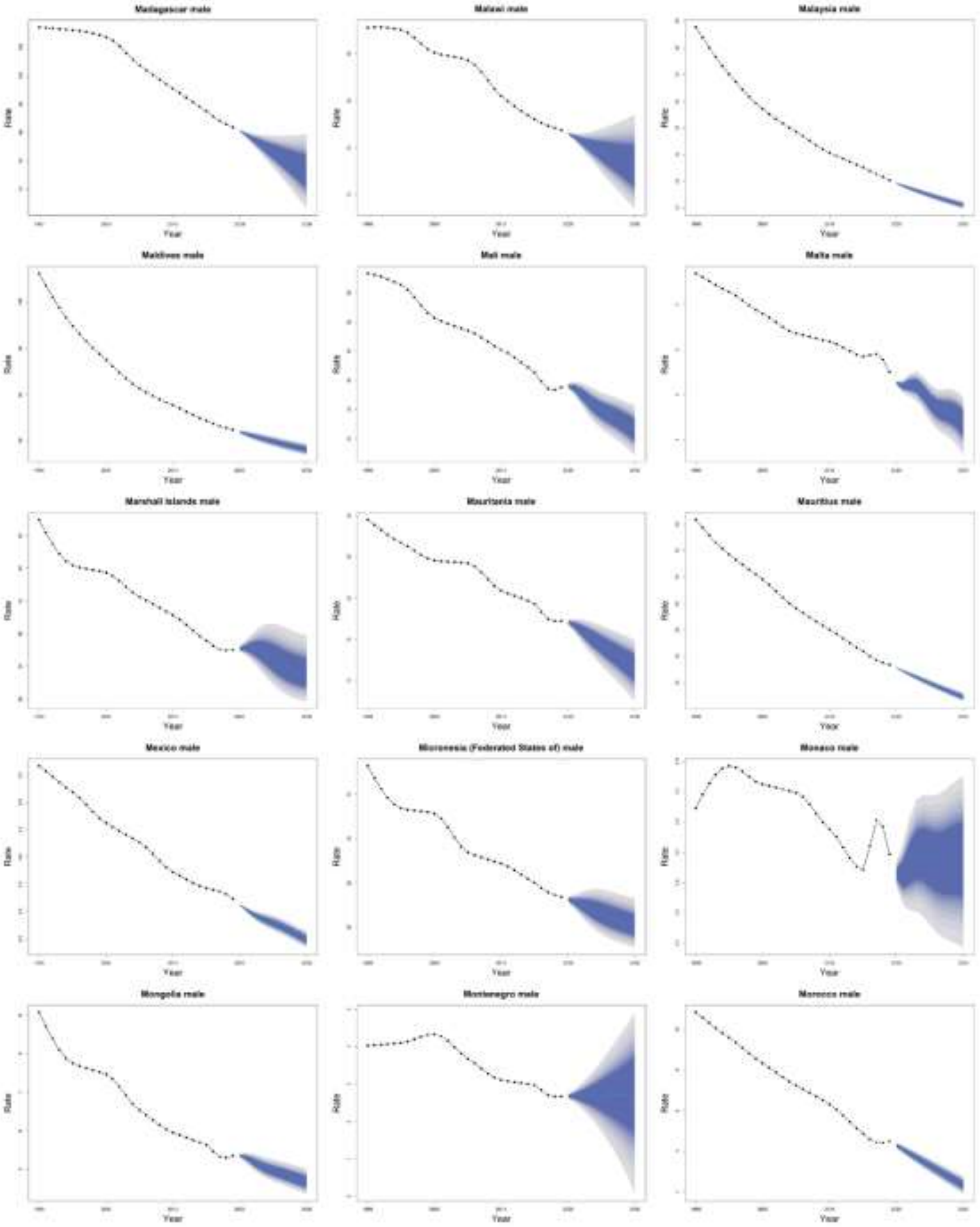
Madagascar male
Malawi male
Malaysia male
Maldives male
Mali male
Malta male
Marshall Islands male
Mauritania male
Mauritius male
Mexico male
Micronesia (Federated States of) male
Monaco male
Mongolia male
Montenegro male
Morocco male
Rate
Year

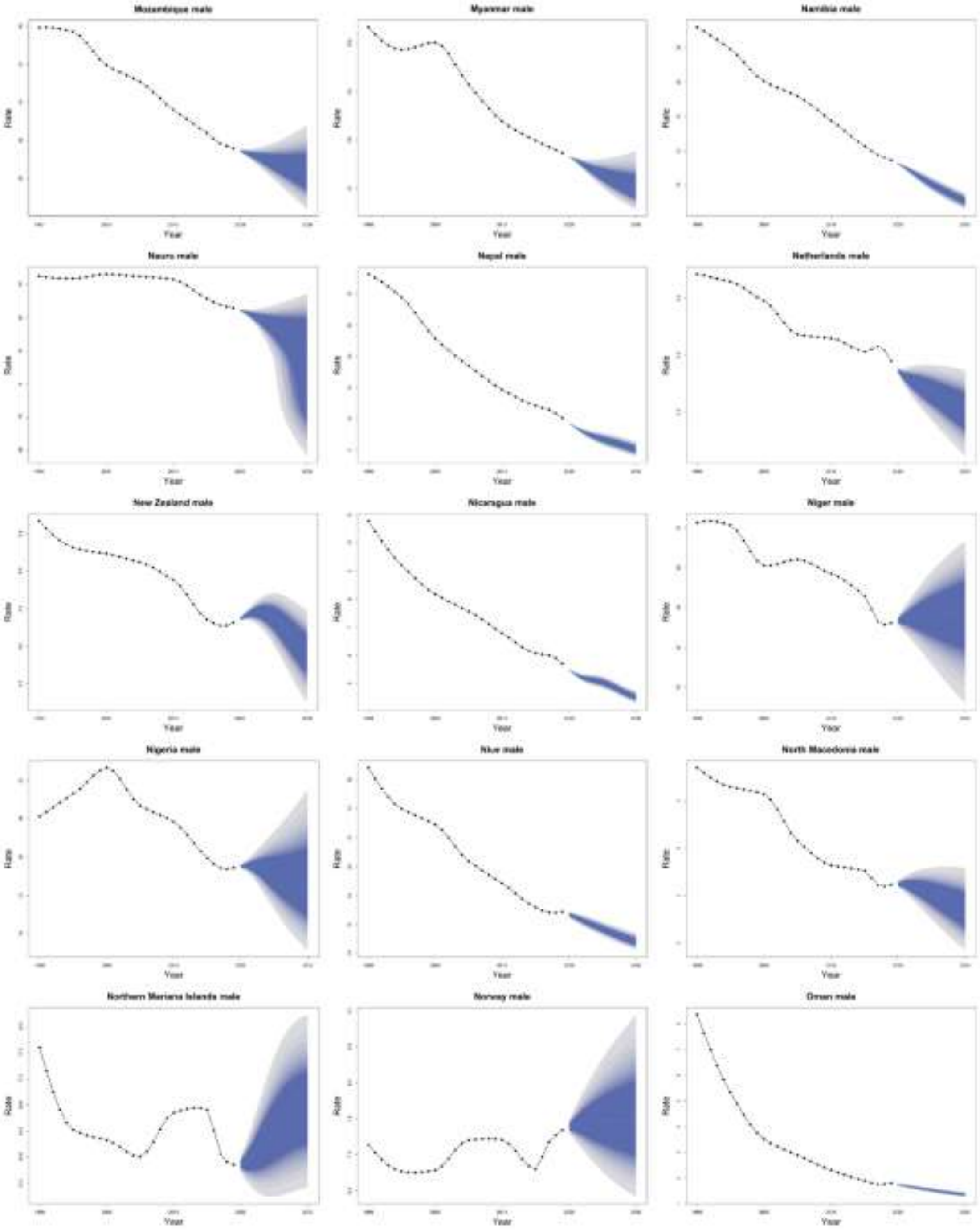
Mozambique male
Myanmar male
Namibia male
Nauru male
Nepal male
Netherlands male
New Zealand male
Nicaragua male
Niger male
Nigeria male
Niue male
North Macedonia male
Northern Mariana Islands male
Norway male
Oman male
Rate
Year

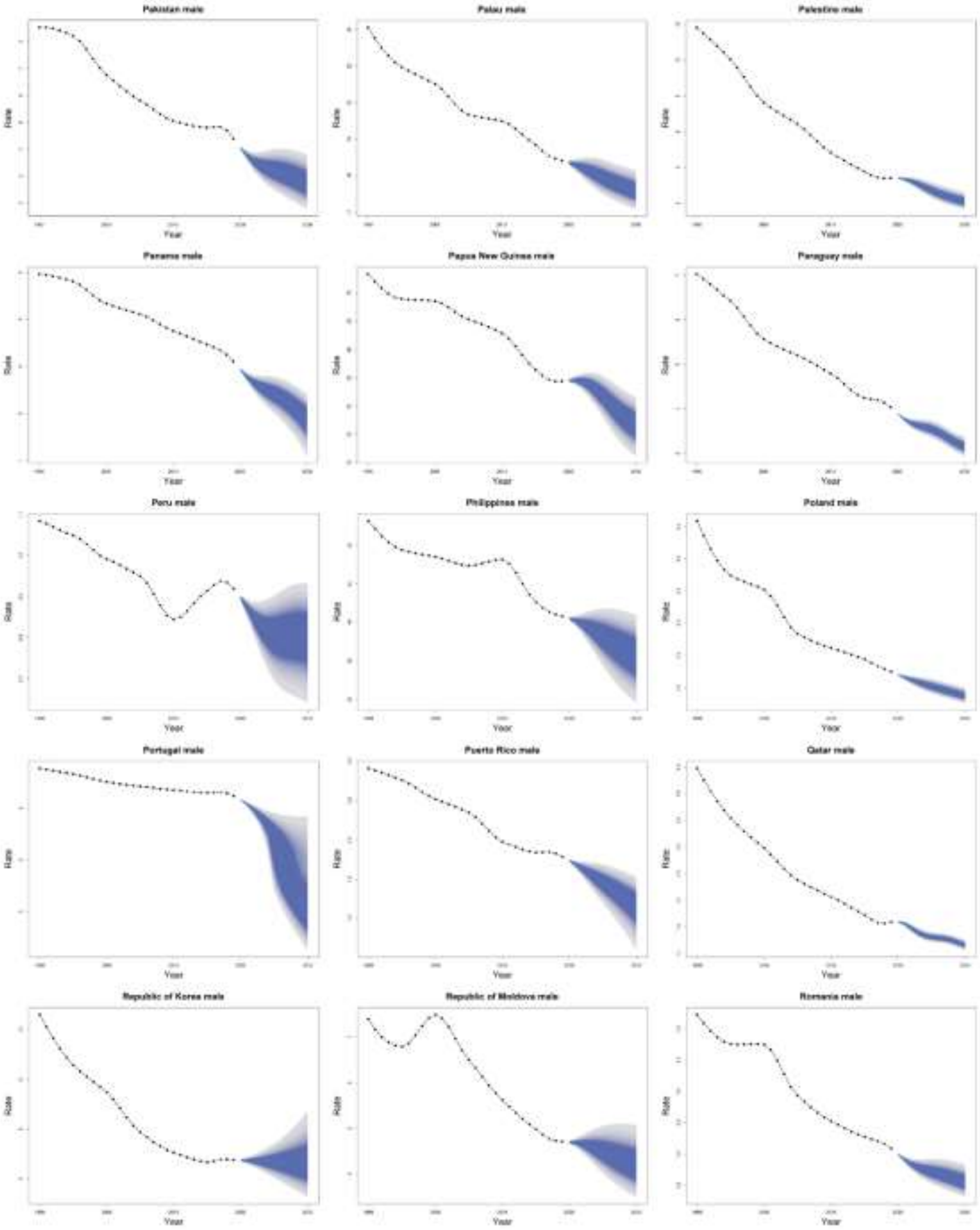
Pakistan male
Palau male
Palestine male
Panama male
Papua New Guinea male
Paraguay male
Peru male
Philippines male
Poland male
Portugal male
Puerto Rico male
Qatar male
Republic of Korea male
Republic of Moldova male
Romania male
Rate
Year

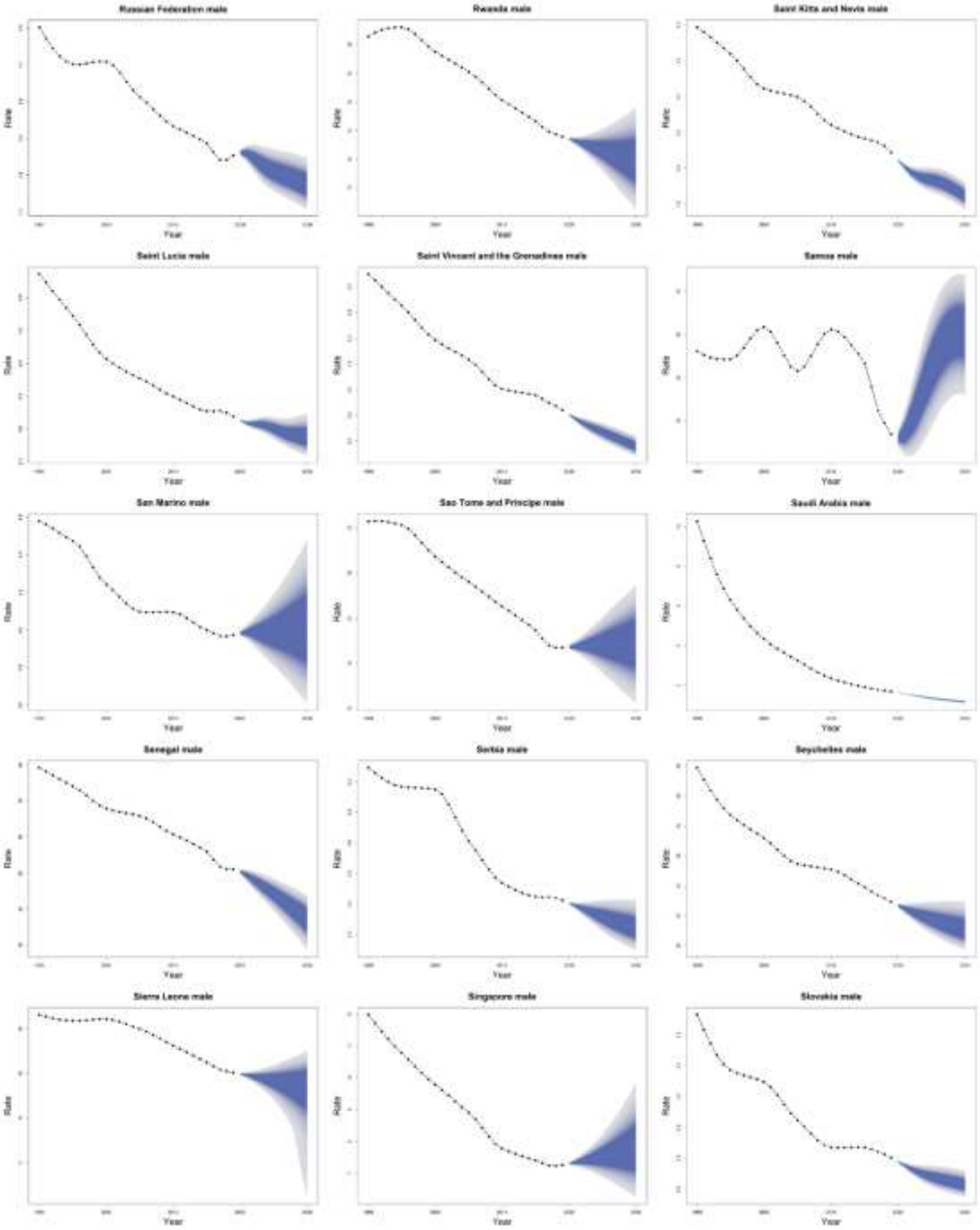
Russian Federation male
Rwanda male
Saint Kitts and Nevis male
Saint Lucia male
Saint Vincent and the Grenadines male
Samoa male
San Marino male
Sao Tome and Principe male
Saudi Arabia male
Senegal male
Serbia male
Seychelles male
Sierra Leone male
Singapore male
Slovakia male
Rate
Year

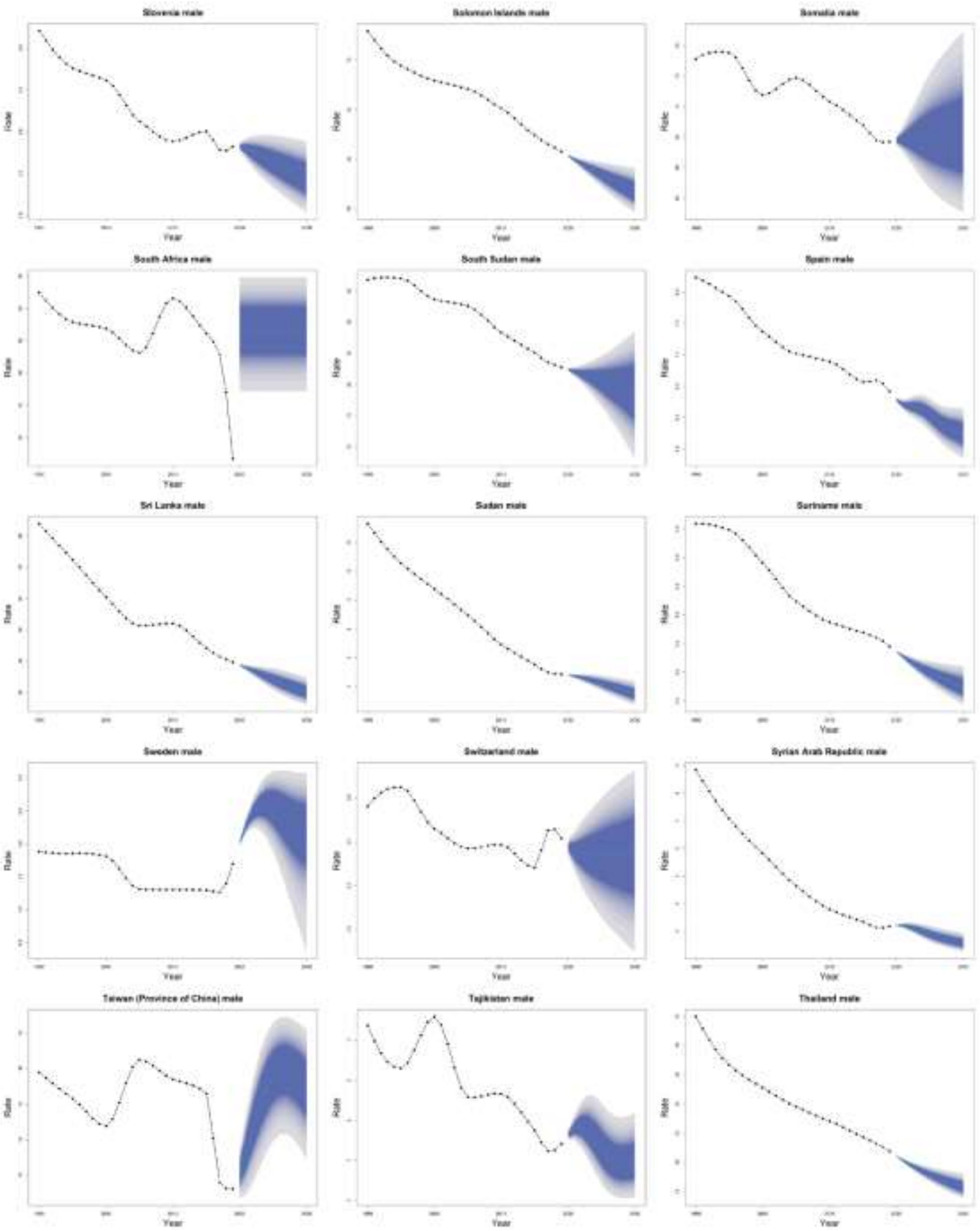
Slovenia male
Solomon Islands male
Somalia male
South Africa male
South Sudan male
Spain male
Sri Lanka male
Sudan male
Suriname male
Sweden male
Switzerland male
Syrian Arab Republic male
Taiwan (Province of China) male
Tajikistan male
Thailand male
Rate
Year

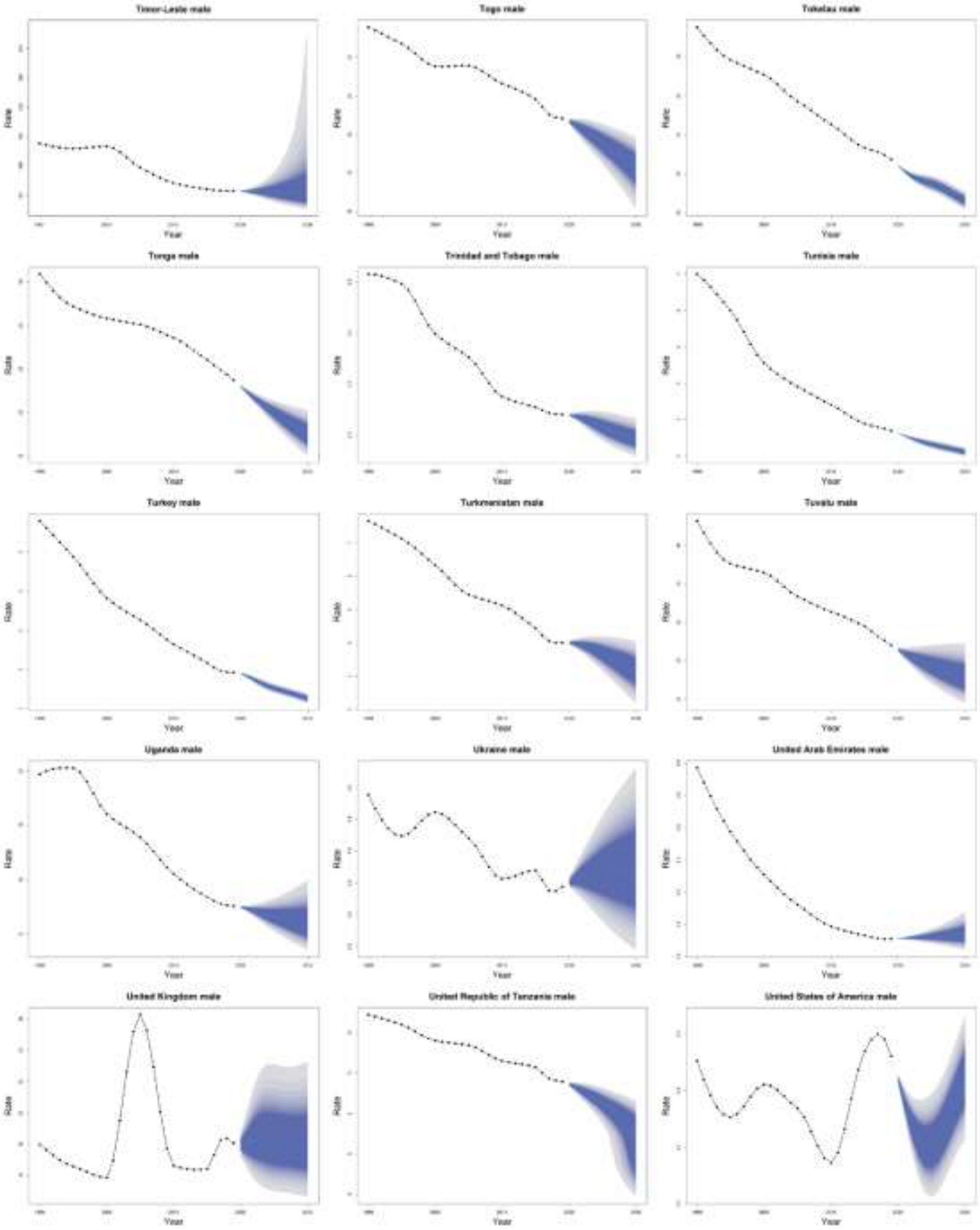
Timor-Leste male
Togo male
Tokelau male
Tonga male
Trinidad and Tobago male
Tunisia male
Turkey male
Turkmenistan male
Tuvalu male
Uganda male
Ukraine male
United Arab Emirates male
United Kingdom male
United Republic of Tanzania male
United States of America male
Rate
Year

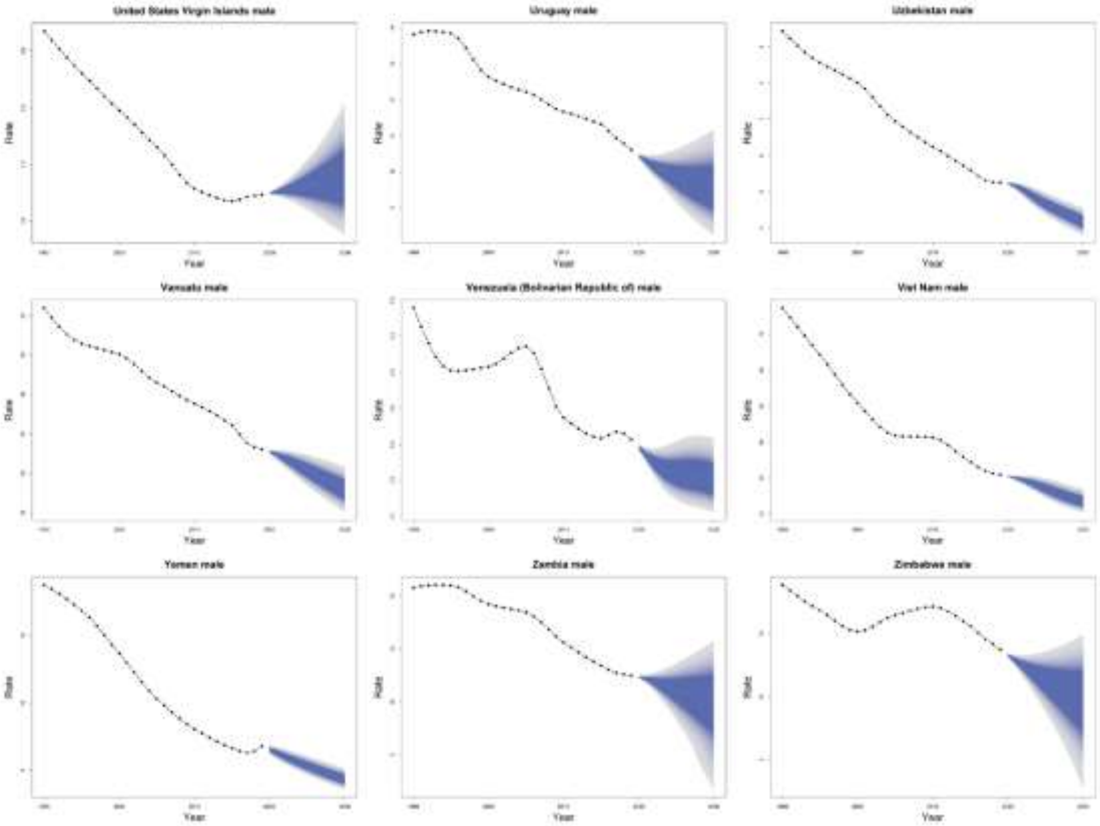

Figure S39. Trends in prevalence rates of 204 countries and territories from 1990 to 2030 for males by ARIMA model.

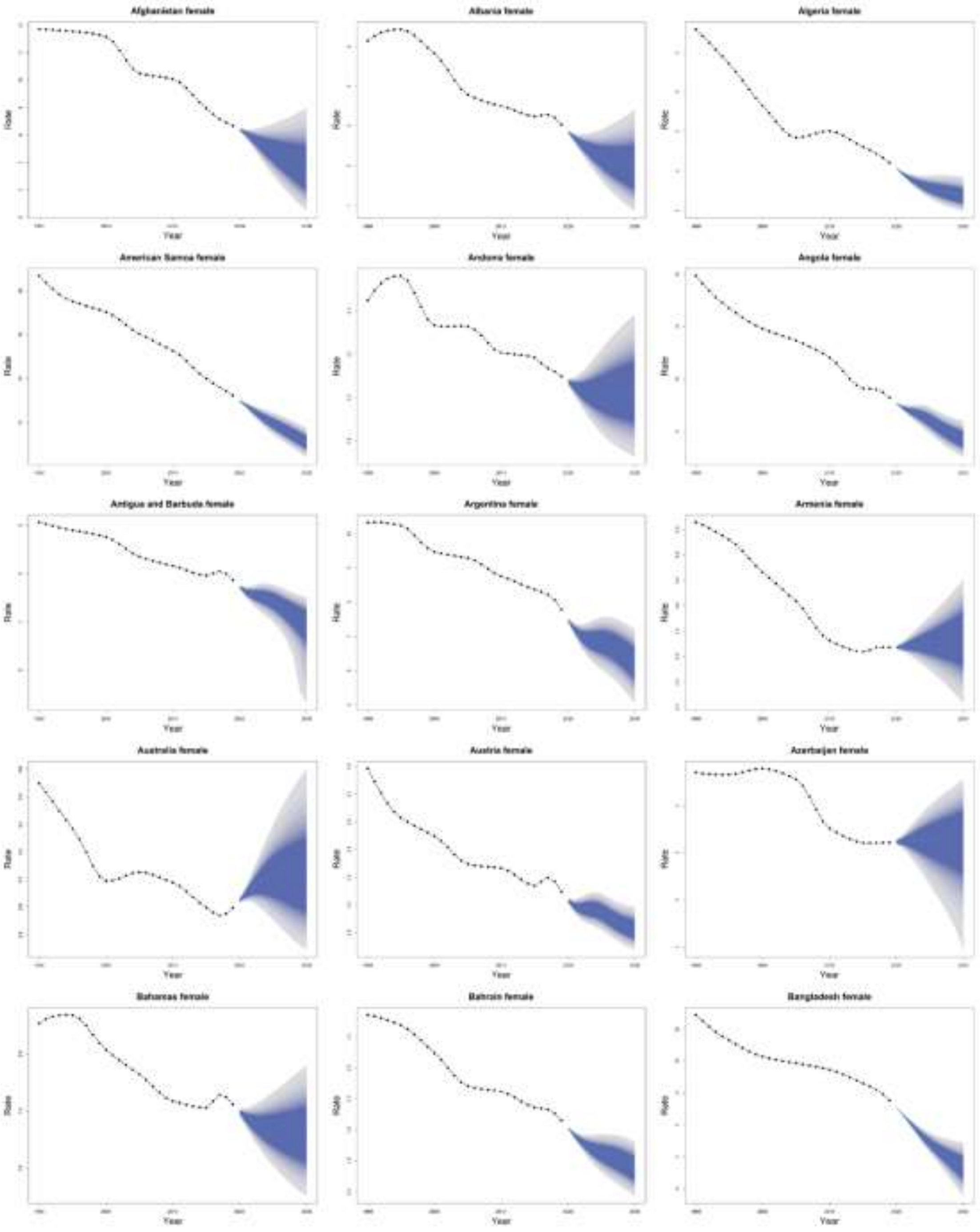

Afghanistan female
Albania female
Algeria female
American Samoa female
Andorra female
Angola female
Antigua and Barbuda female
Argentina female
Armenia female
Australia female
Austria female
Azerbaijan female
Bahamas female
Bahrain female
Bangladesh female
Rate
Year

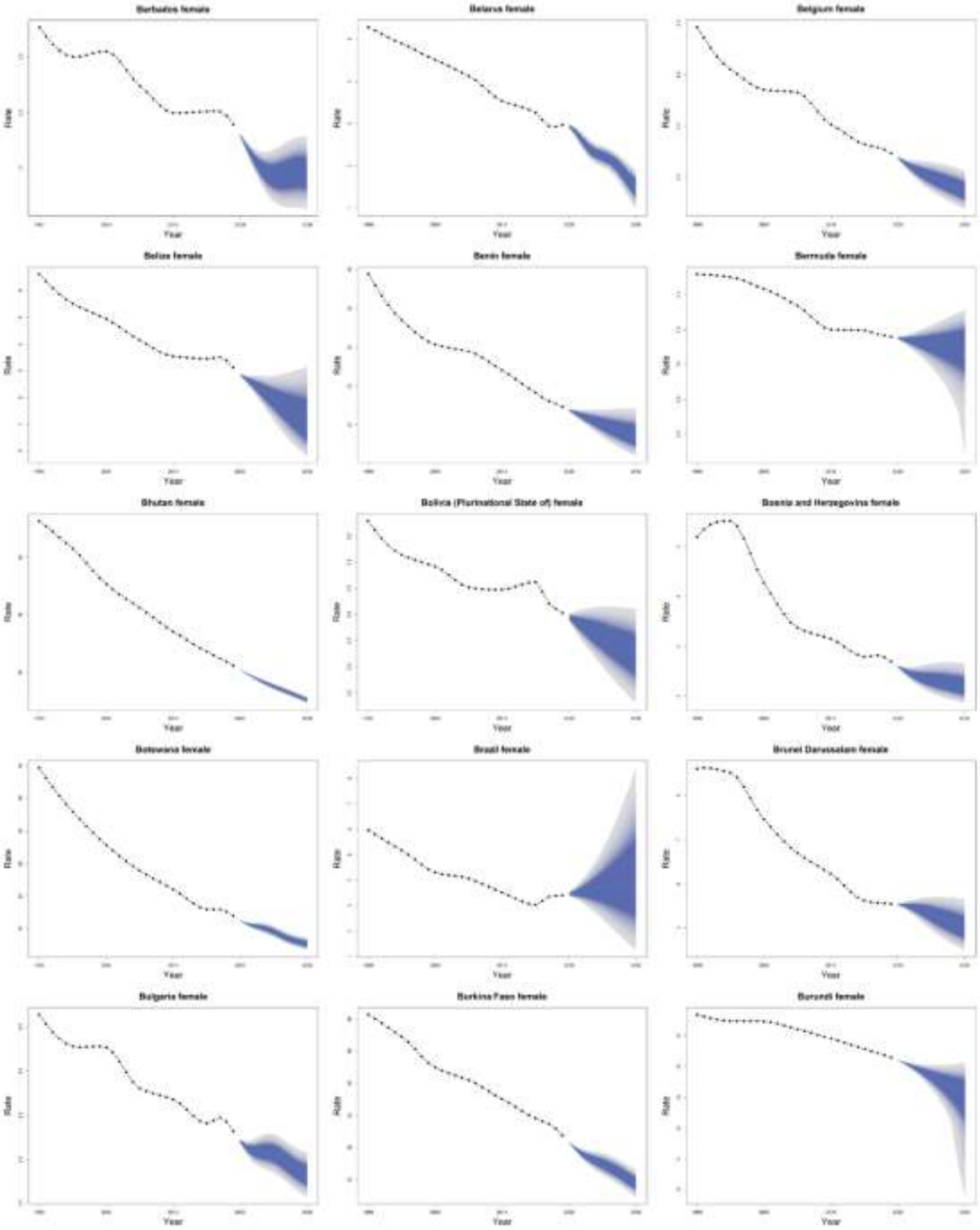
Barbados female
Belarus female
Belgium female
Belize female
Benin female
Bermuda female
Bhutan female
Bolivia (Plurinational State of) female
Bosnia and Herzegovina female
Botswana female
Brazil female
Brunei Darussalam female
Bulgaria female
Burkina Faso female
Burundi female
Rate
Year

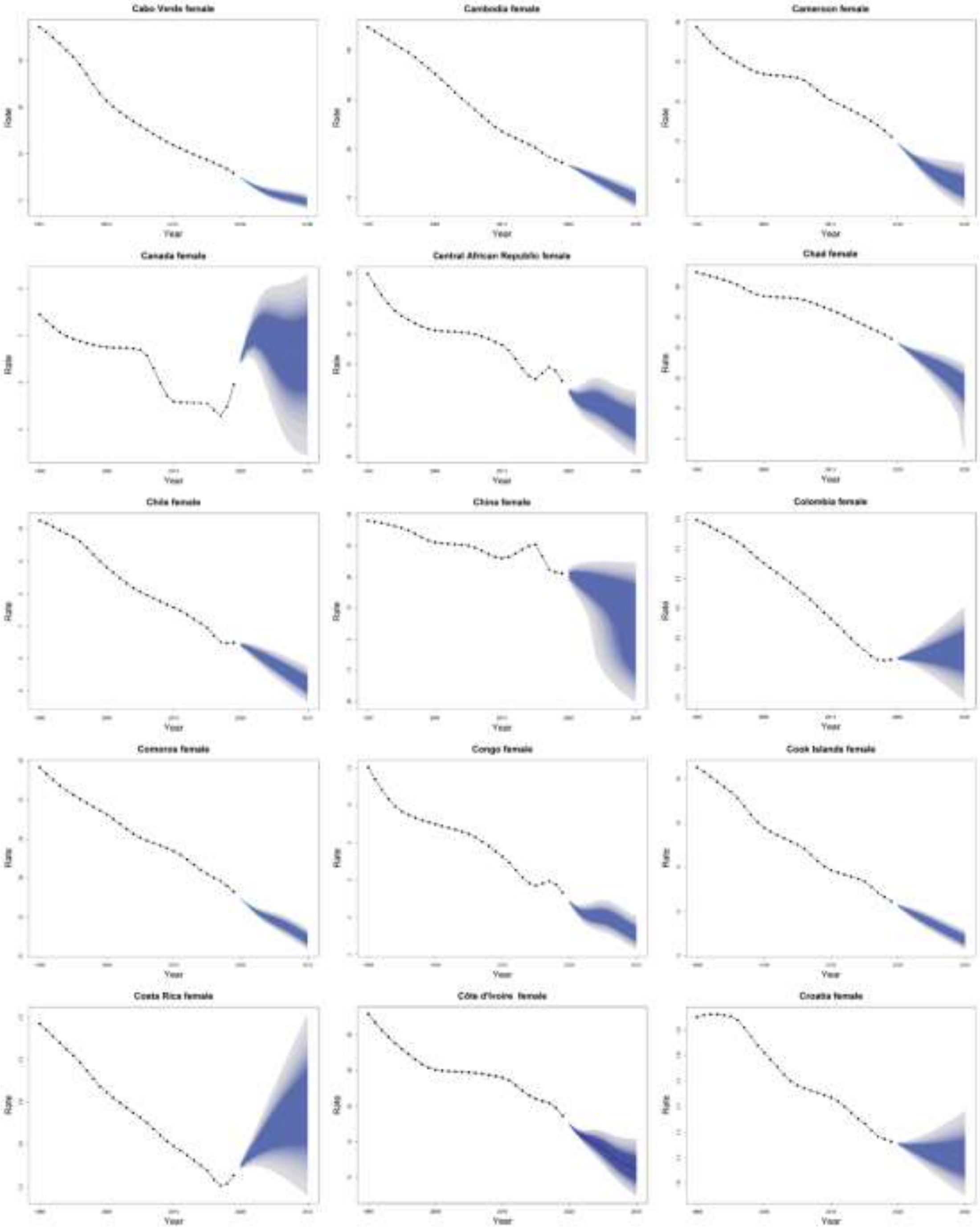
Cabo Verde female
Cambodia female
Cameroon female
Canada female
Central African Republic female
Chad female
Chile female
China female
Colombia female
Comoros female
Congo female
Cook Islands female
Costa Rica female
Côte d'Ivoire female
Croatia female
Rate
Year

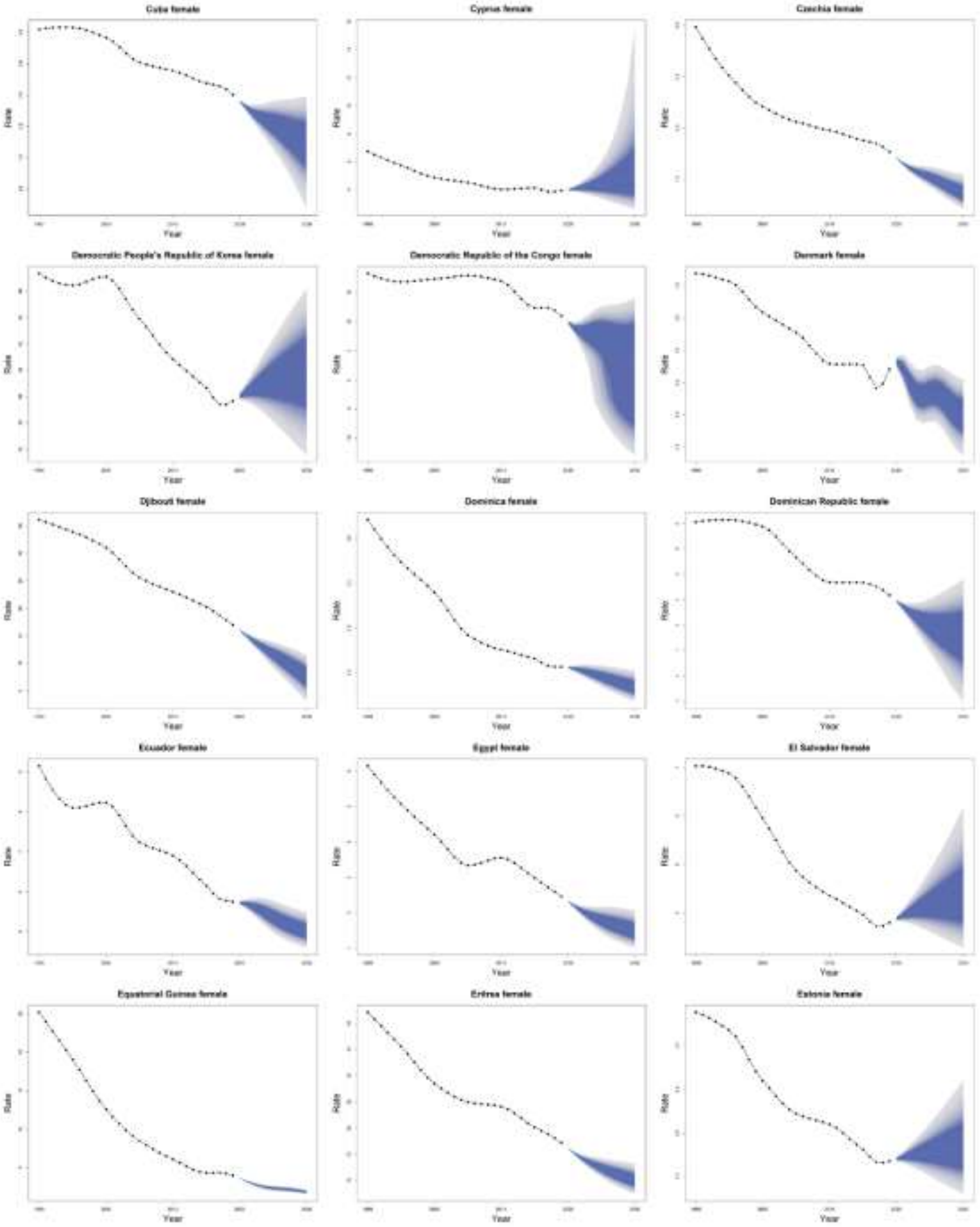
Cuba female
Cyprus female
Czechia female
Democratic People's Republic of Korea female
Democratic Republic of the Congo female
Denmark female
Djibouti female
Dominica female
Dominican Republic female
Ecuador female
Egypt female
El Salvador female
Equatorial Guinea female
Eritrea female
Estonia female
Rate
Year

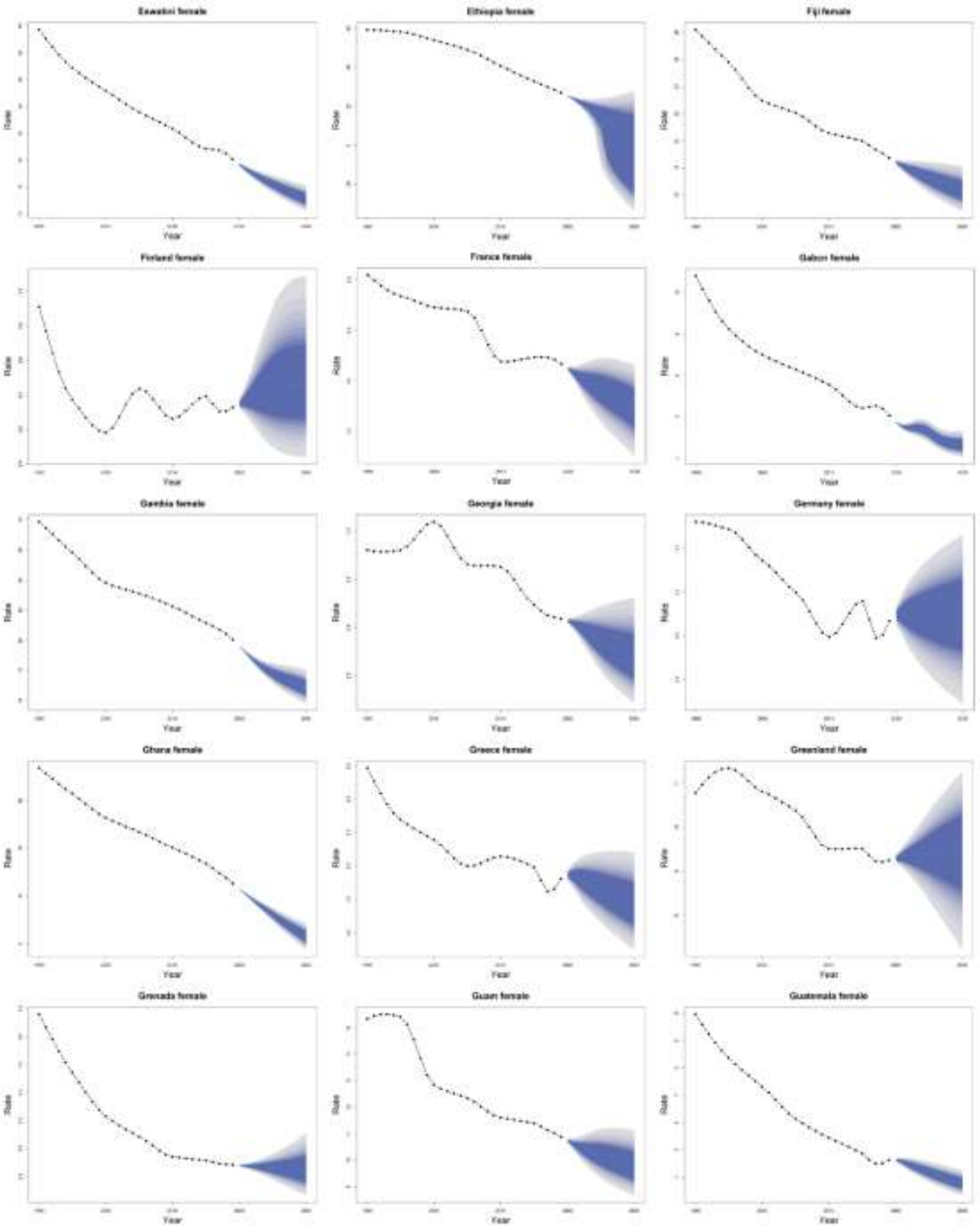
Eswatini female
Ethiopia female
Fiji female
Finland female
France female
Gabon female
Gambia female
Georgia female
Germany female
Ghana female
Greece female
Greenland female
Grenada female
Guam female
Guatemala female
Year
Rate

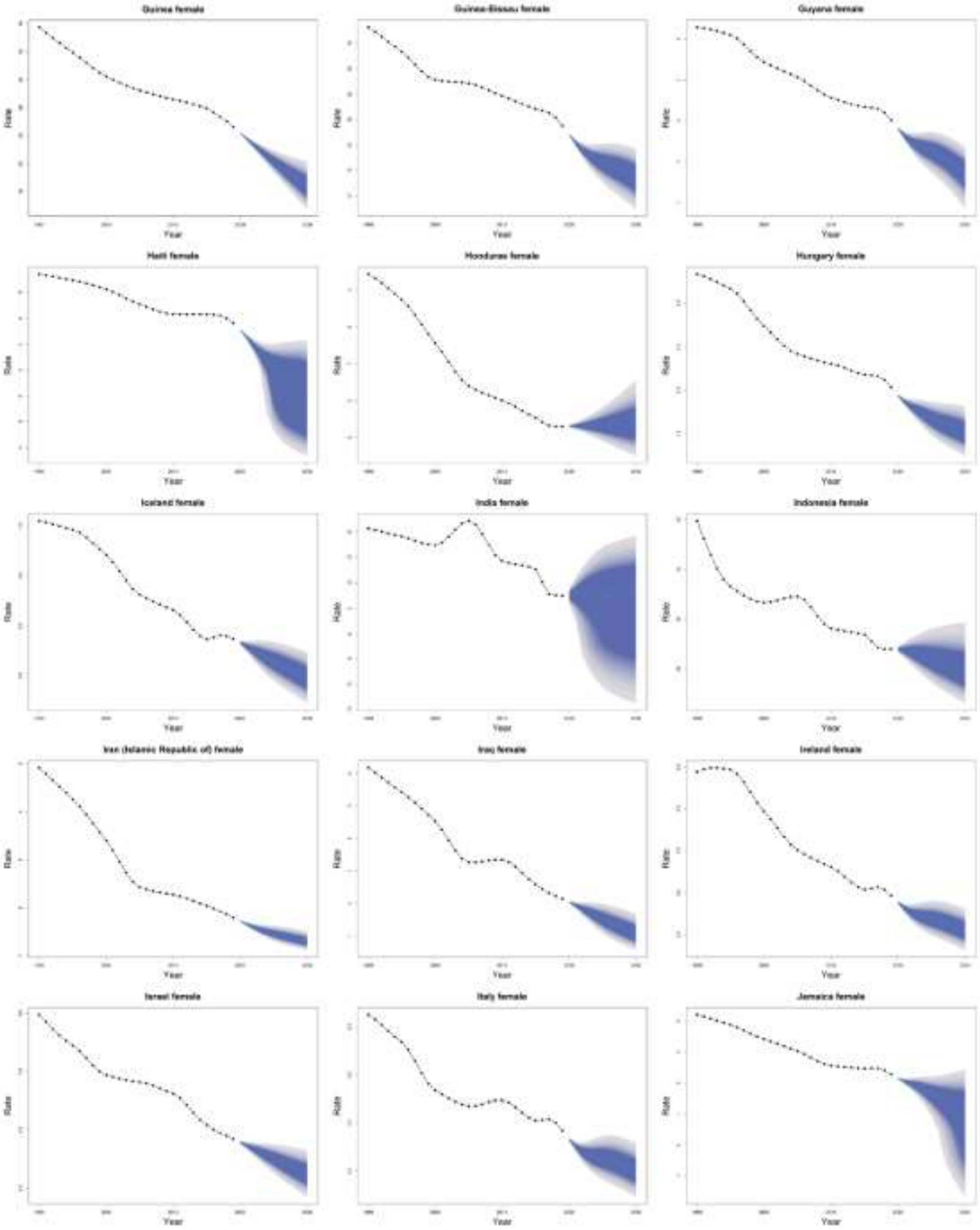
Guinea female
Guinea-Bissau female
Guyana female
Haiti female
Honduras female
Hungary female
Iceland female
India female
Indonesia female
Iran (Islamic Republic of) female
Iraq female
Ireland female
Israel female
Italy female
Jamaica female
Rate
Year

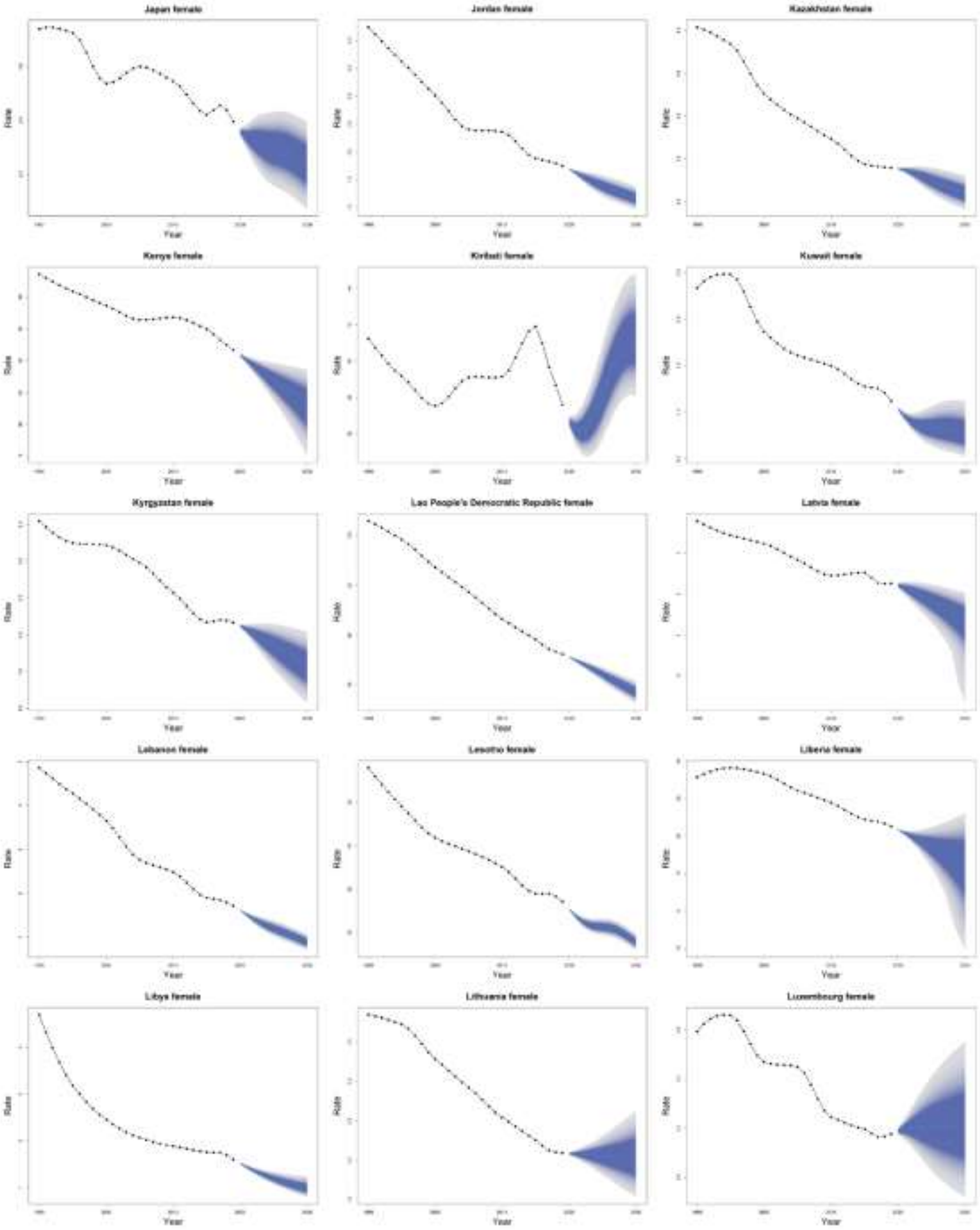
Japan female
Jordan female
Kazakhstan female
Kenya female
Kiribati female
Kuwait female
Kyrgyzstan female
Lao People's Democratic Republic female
Latvia female
Lebanon female
Lesotho female
Liberia female
Libya female
Lithuania female
Luxembourg female
Rate
Year

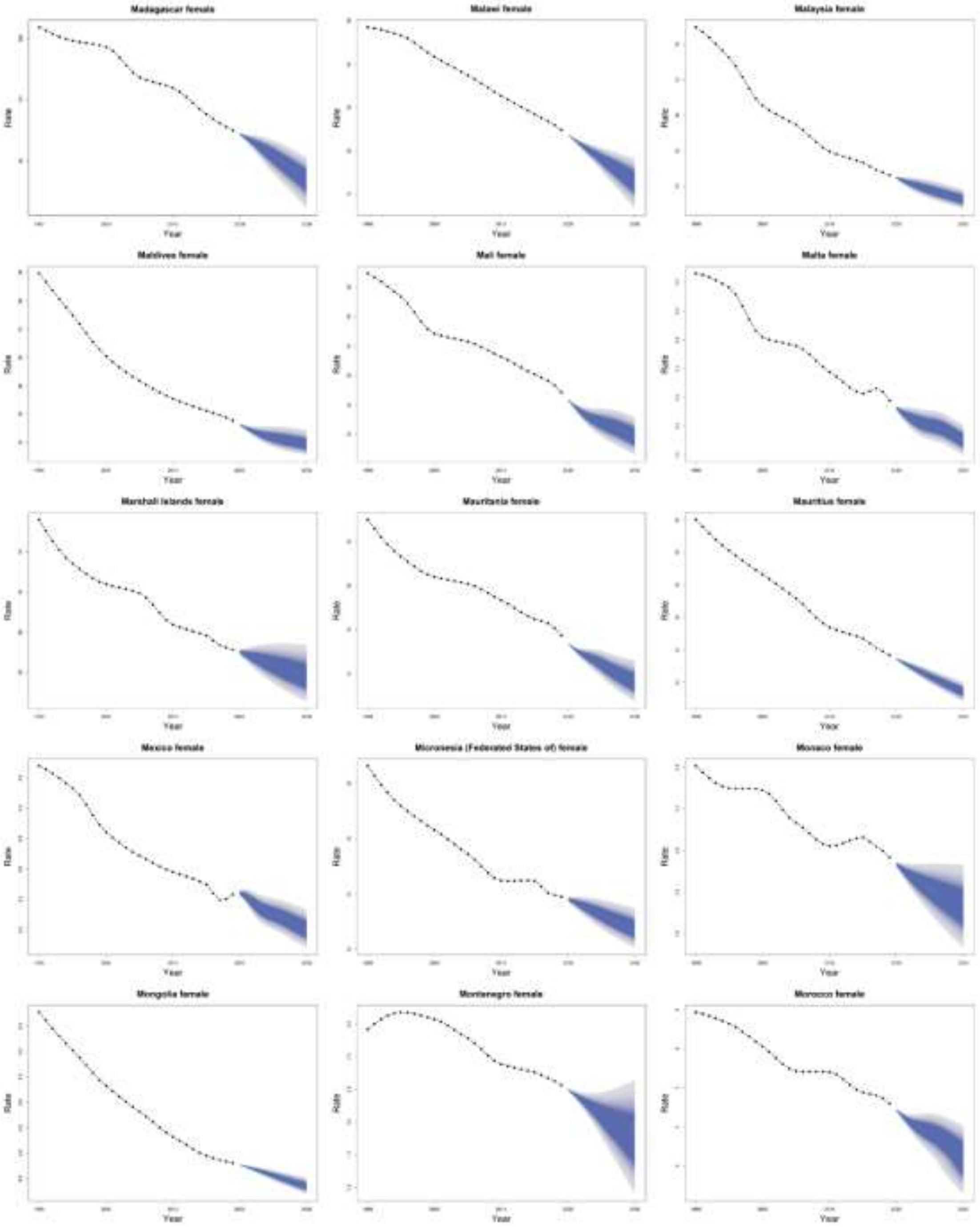
Madagascar female
Malawi female
Malaysia female
Maldives female
Mali female
Malta female
Marshall Islands female
Mauritania female
Mauritius female
Mexico female
Micronesia (Federated States of) female
Monaco female
Mongolia female
Montenegro female
Morocco female
Rate
Year

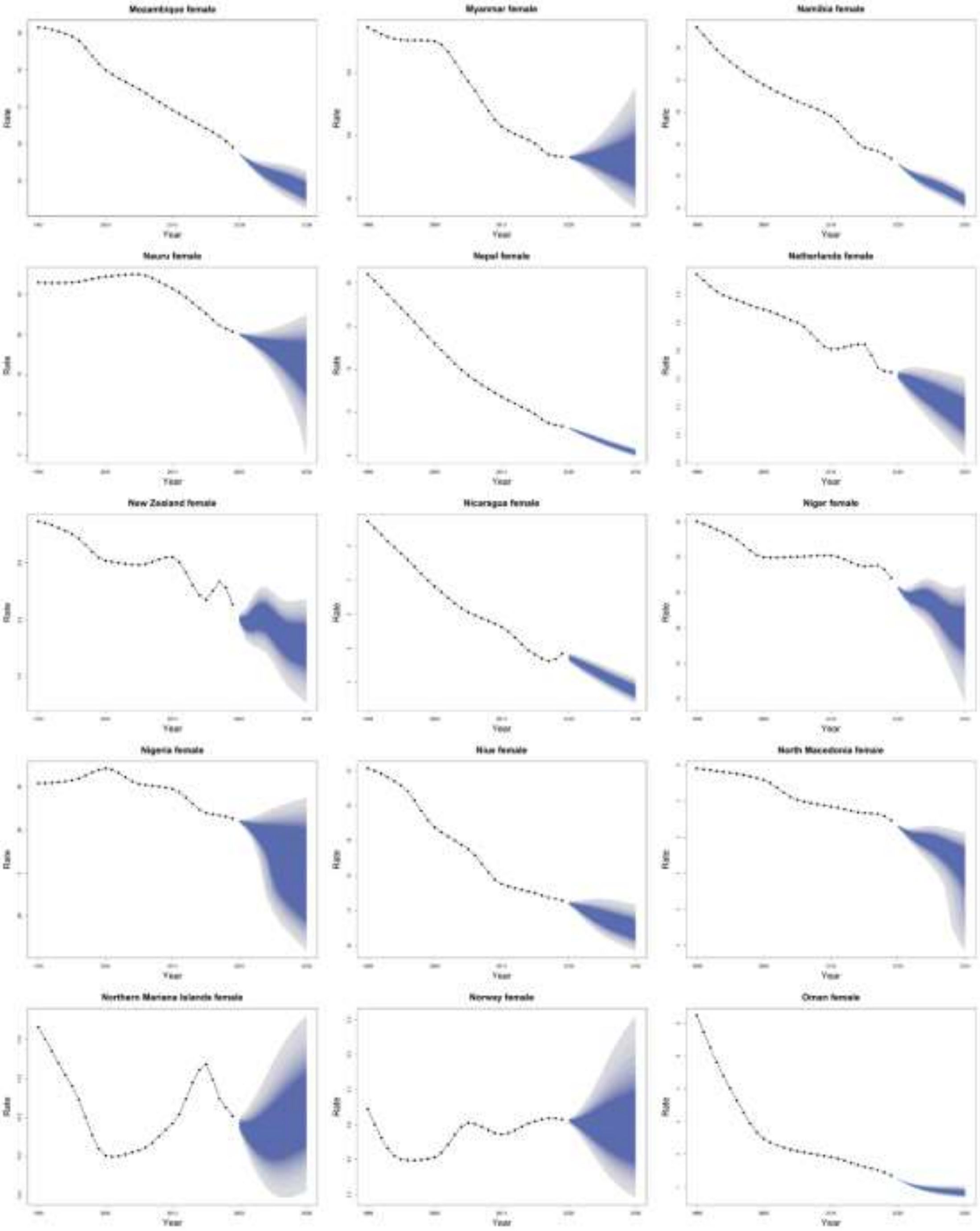
Mozambique female
Myanmar female
Namibia female
Nauru female
Nepal female
Netherlands female
New Zealand female
Nicaragua female
Niger female
Nigeria female
Niue female
North Macedonia female
Northern Mariana Islands female
Norway female
Oman female
Rate
Year

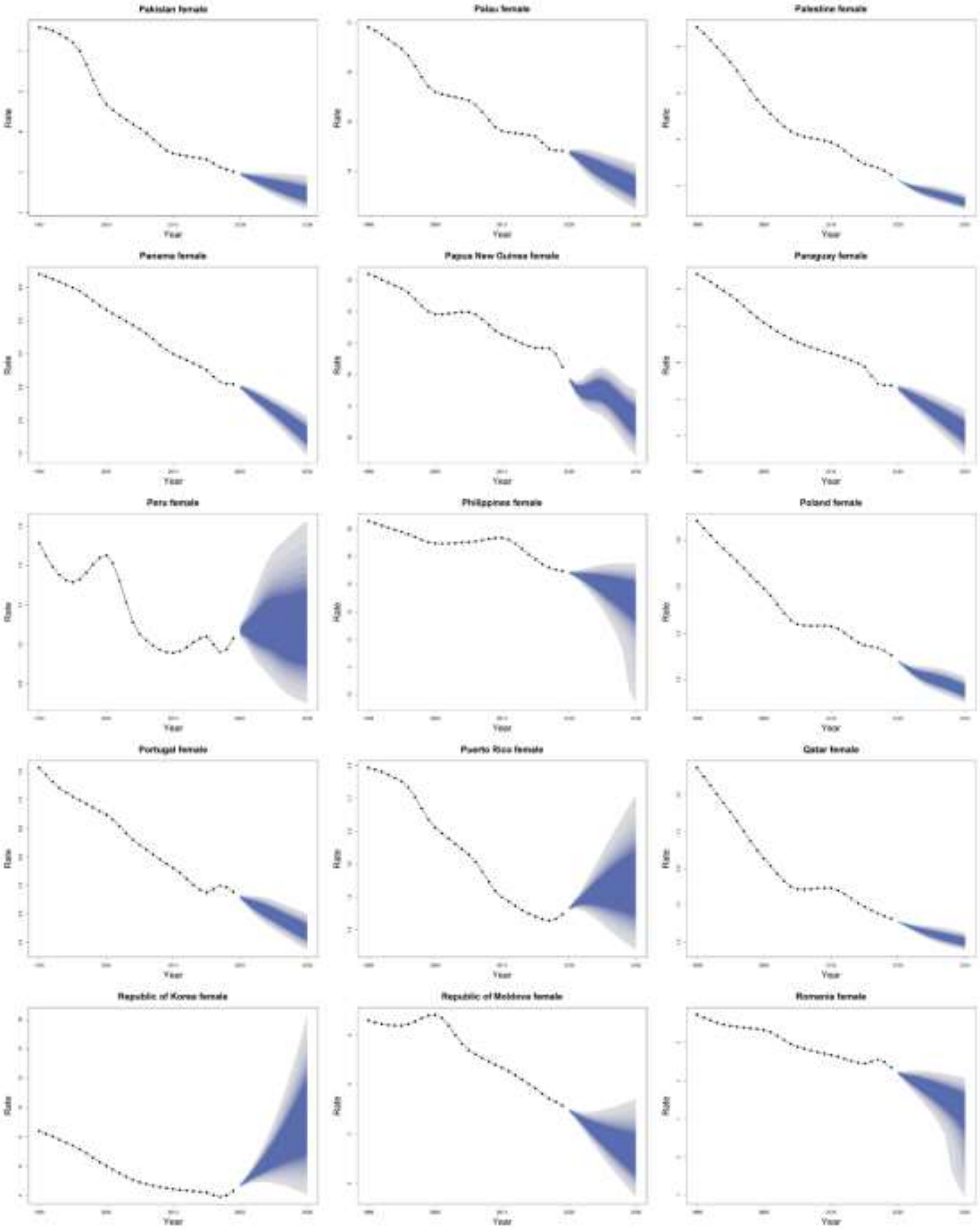

Pakistan female
Palau female
Palestine female
Panama female
Papua New Guinea female
Paraguay female
Peru female
Philippines female
Poland female
Portugal female
Puerto Rico female
Qatar female
Republic of Korea female
Republic of Moldova female
Romania female
Rate
Year

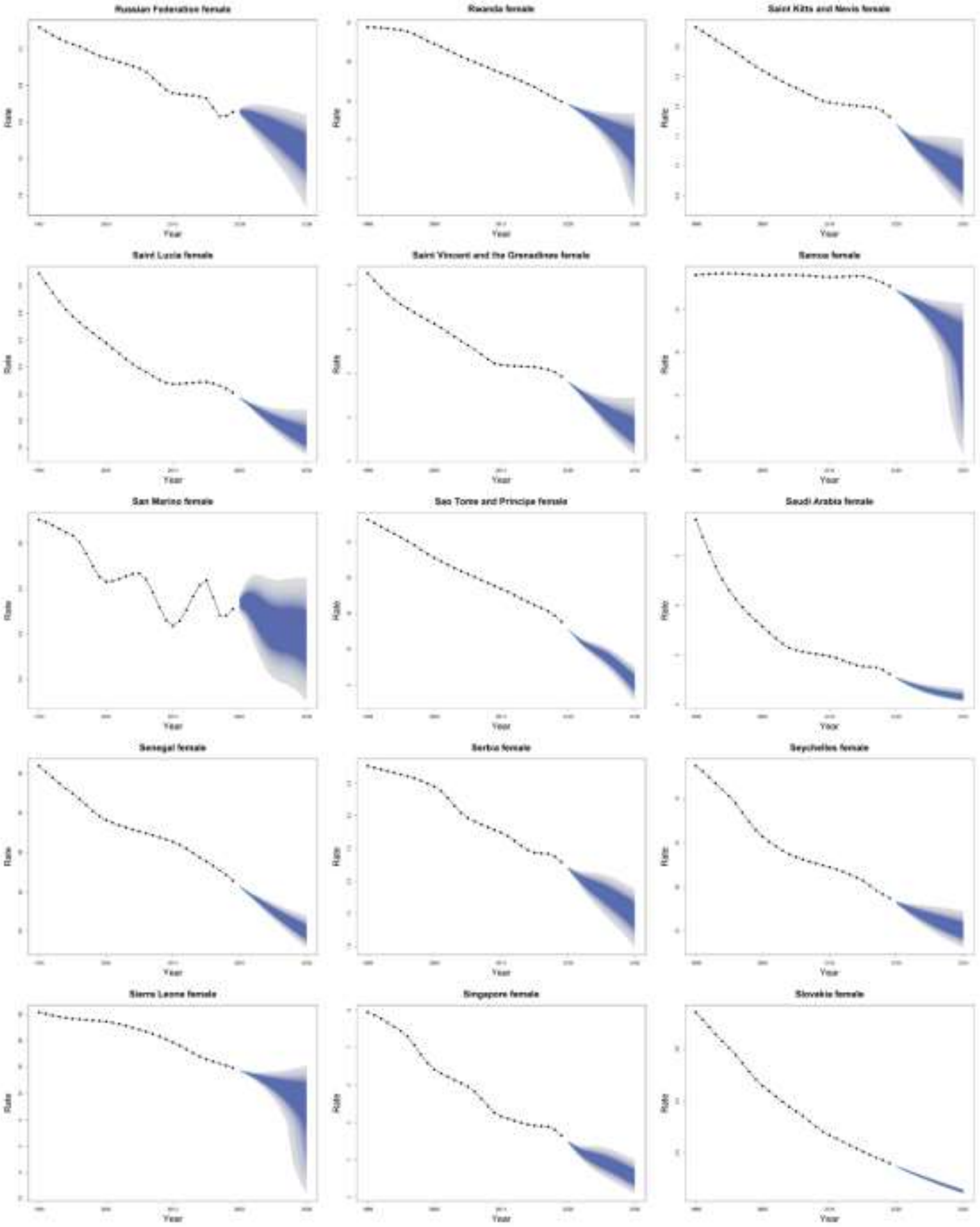
Russian Federation female
Rwanda female
Saint Kitts and Nevis female
Saint Lucia female
Saint Vincent and the Grenadines female
Samoa female
San Marino female
Sao Tome and Principe female
Saudi Arabia female
Senegal female
Serbia female
Seychelles female
Sierra Leone female
Singapore female
Slovakia female
Rate
Year

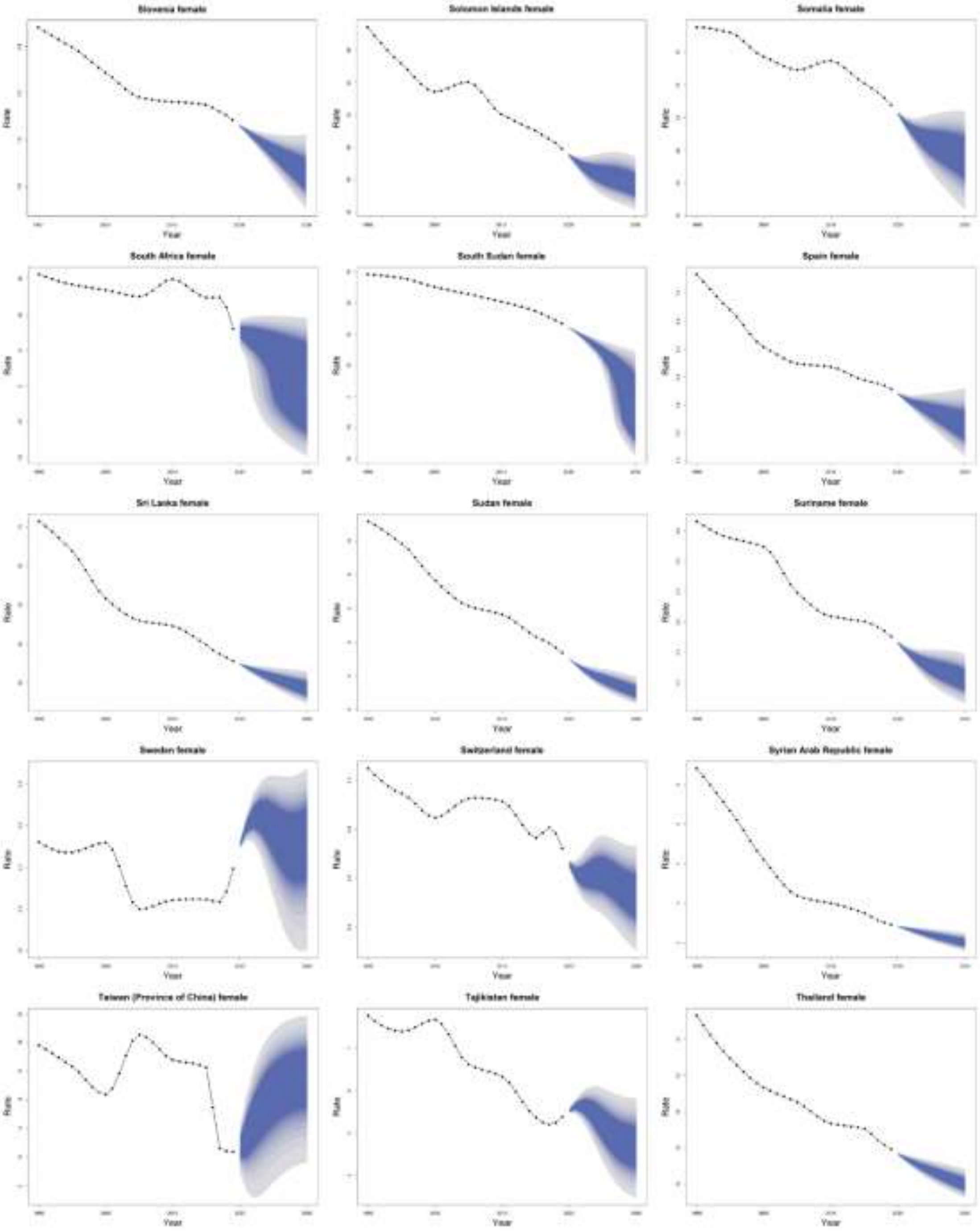
Slovenia female
Solomon Islands female
Somalia female
South Africa female
South Sudan female
Spain female
Sri Lanka female
Sudan female
Suriname female
Sweden female
Switzerland female
Syrian Arab Republic female
Taiwan (Province of China) female
Tajikistan female
Thailand female
Rate
Year

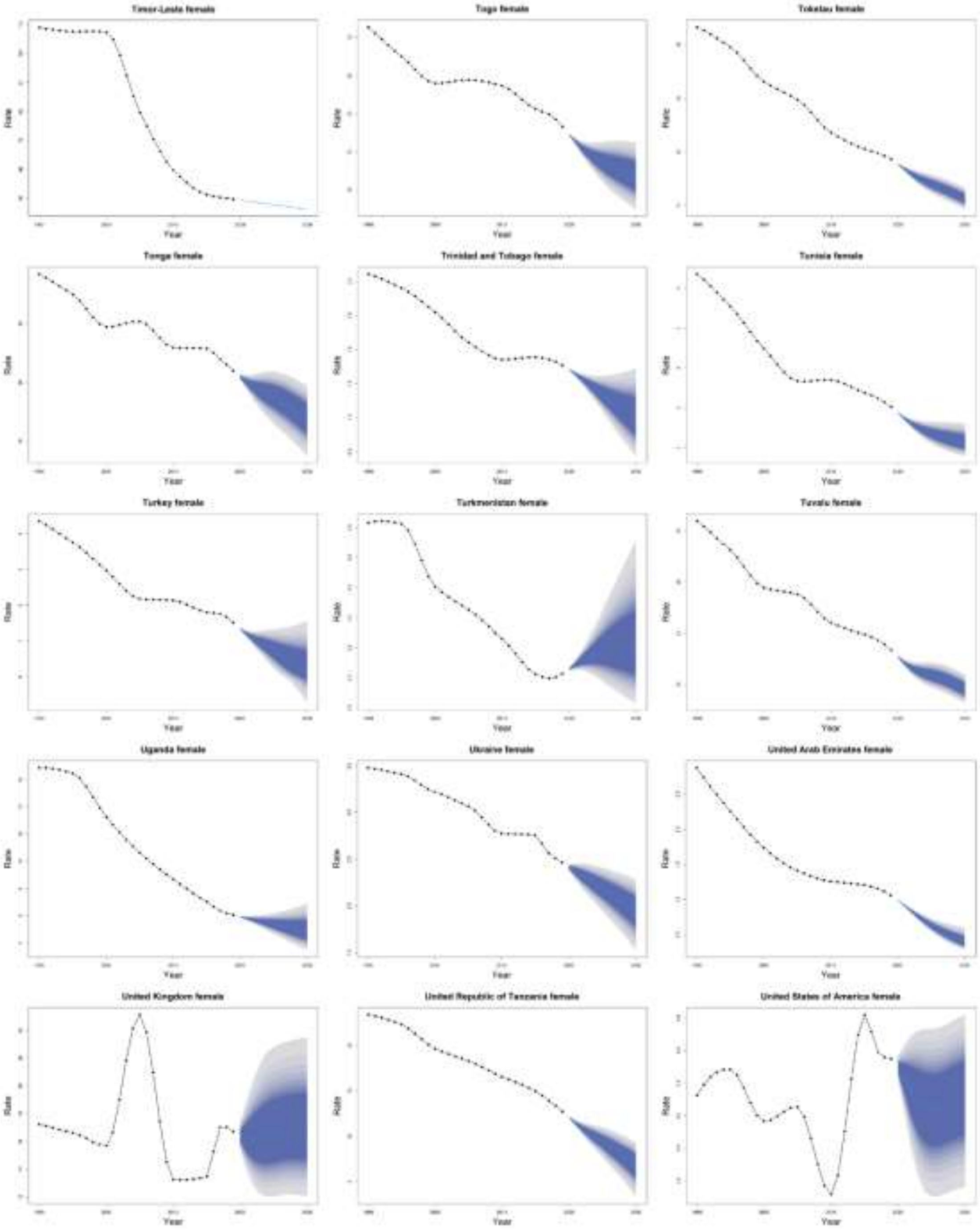
Timor-Leste female
Togo female
Tokelau female
Tonga female
Trinidad and Tobago female
Tunisia female
Turkey female
Turkmenistan female
Tuvalu female
Uganda female
Ukraine female
United Arab Emirates female
United Kingdom female
United Republic of Tanzania female
United States of America female
Rate
Year

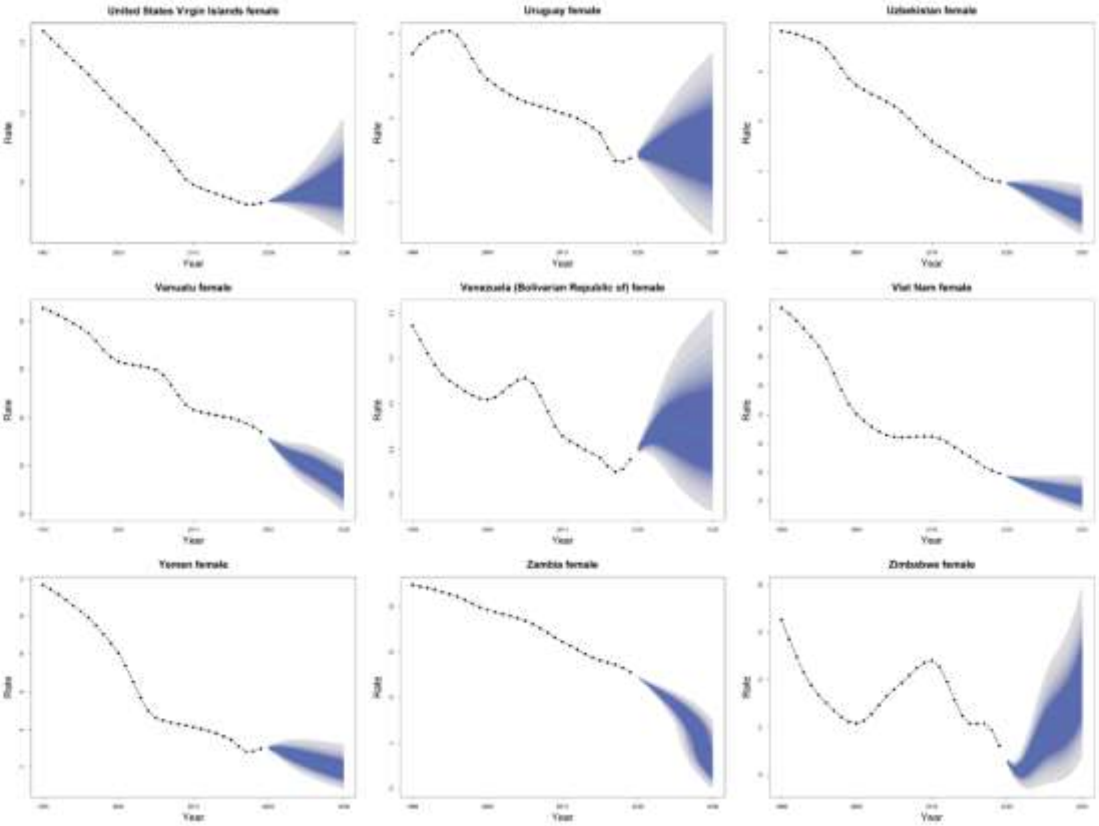

Figure S40. Trends in prevalence rates of 204 countries and territories from 1990 to 2030 for females by ARIMA model.

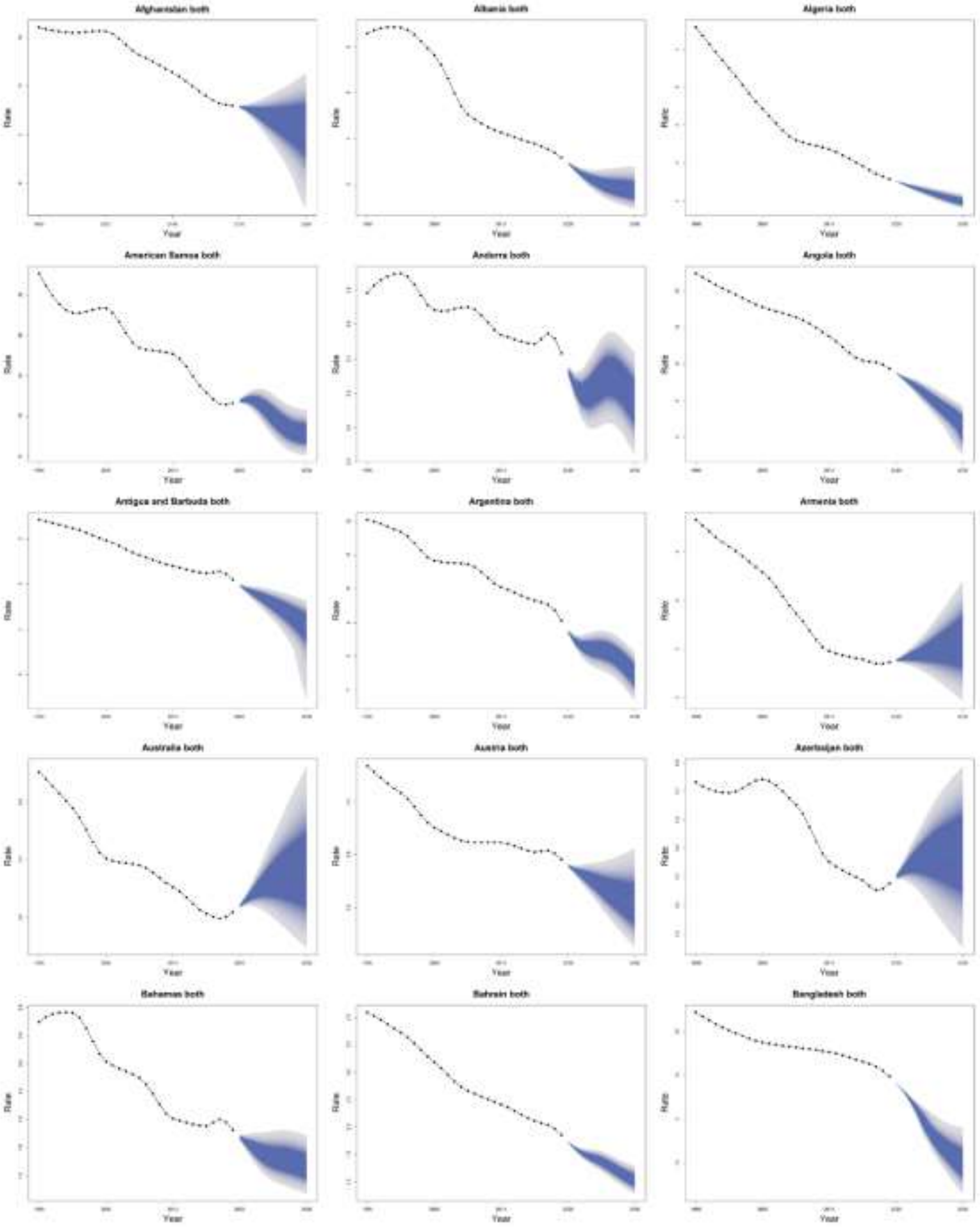
Afghanistan both
Albania both
Algeria both
American Samoa both
Andorra both
Angola both
Antigua and Barbuda both
Argentina both
Armenia both
Australia both
Austria both
Azerbaijan both
Bahamas both
Bahrain both
Bangladesh both
Rate
Year

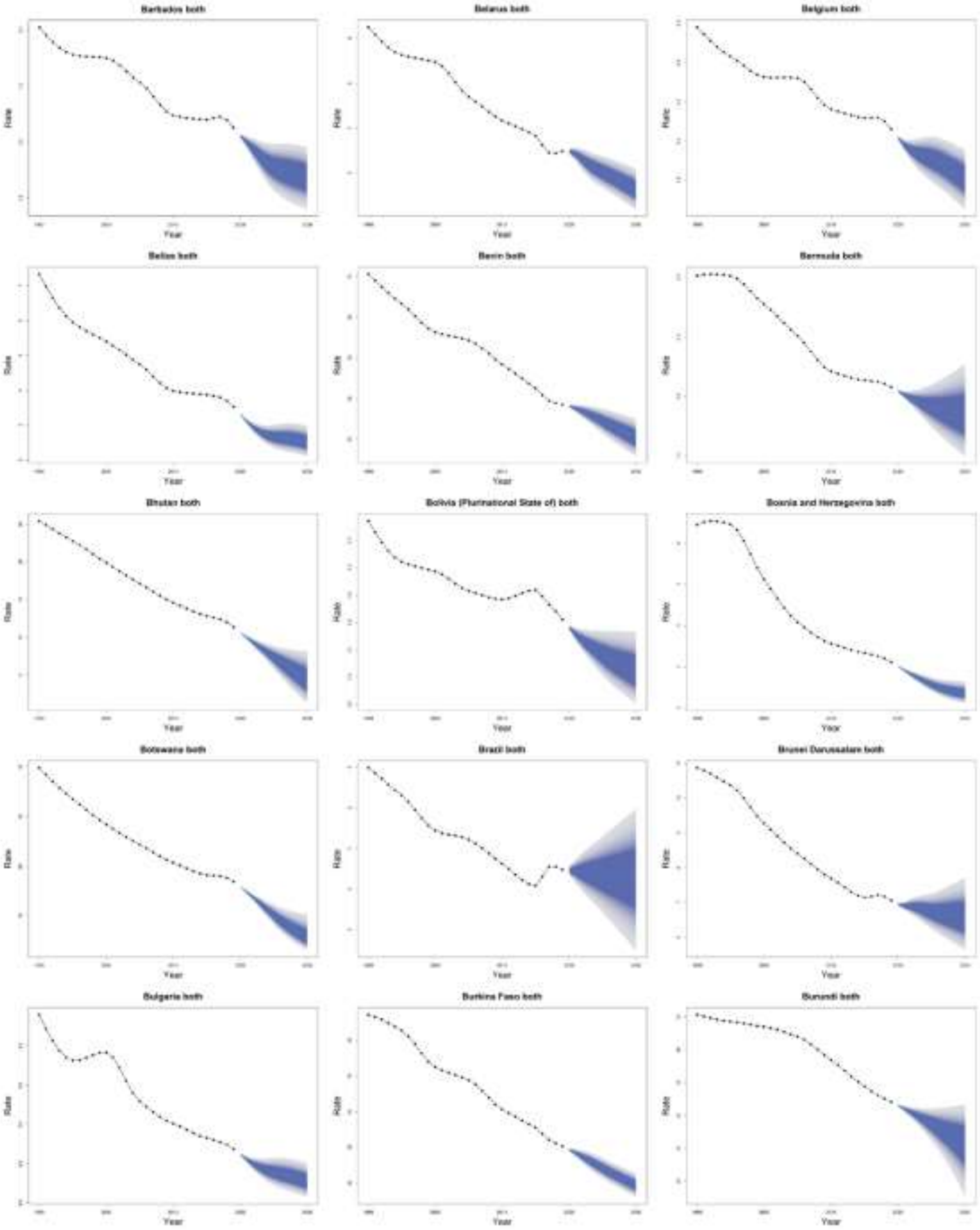

Barbados both
Belarus both
Belgium both
Belize both
Benin both
Bermuda both
Bhutan both
Bolivia (Plurinational State of) both
Bosnia and Herzegovina both
Botswana both
Brazil both
Brunei Darussalam both
Bulgaria both
Burkina Faso both
Burundi both
Rate
Year

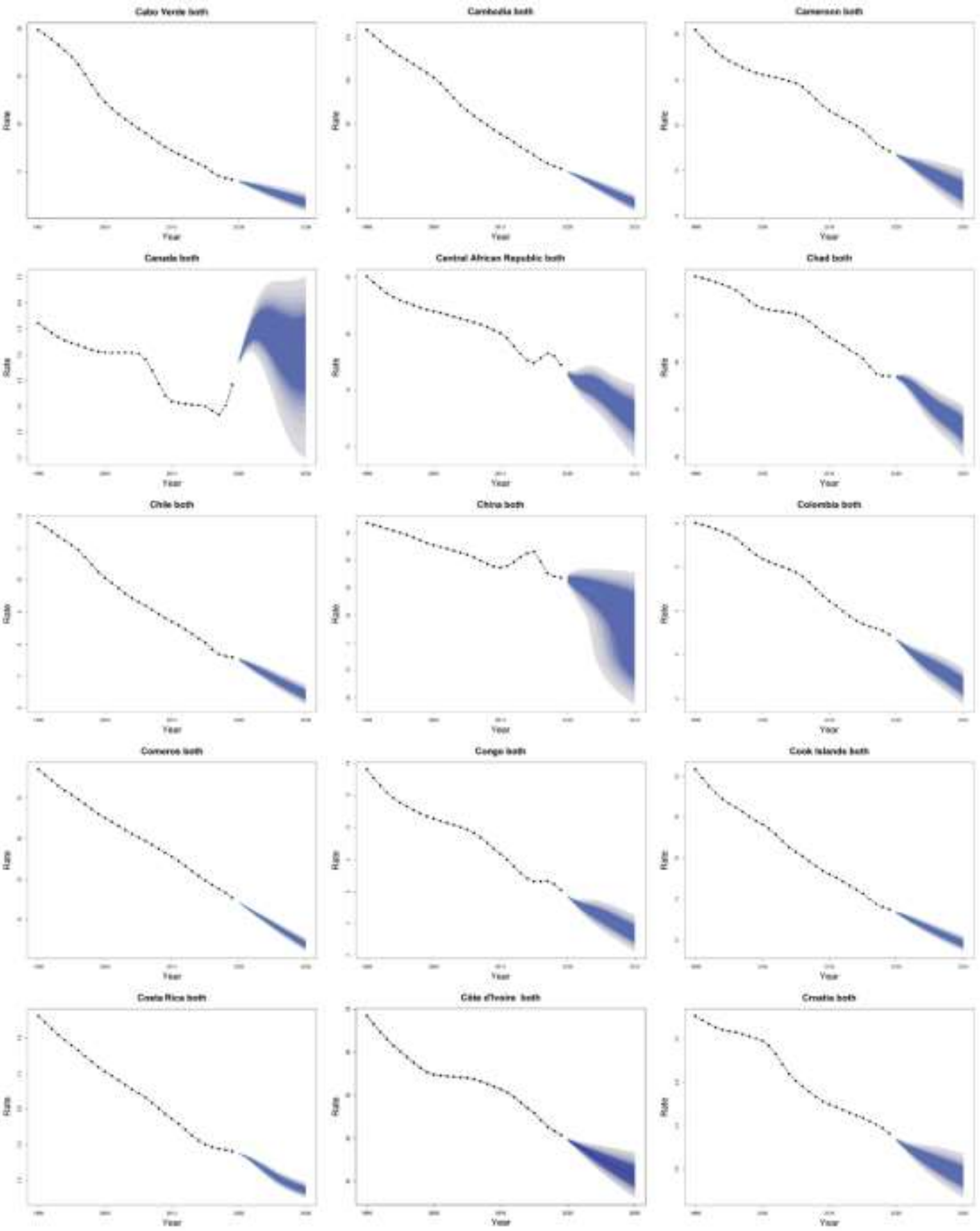
Cabo Verde both
Cambodia both
Cameroon both
Canada both
Central African Republic both
Chad both
Chile both
China both
Colombia both
Comoros both
Congo both
Cook Islands both
Costa Rica both
Côte d'Ivoire both
Croatia both
Rate
Year

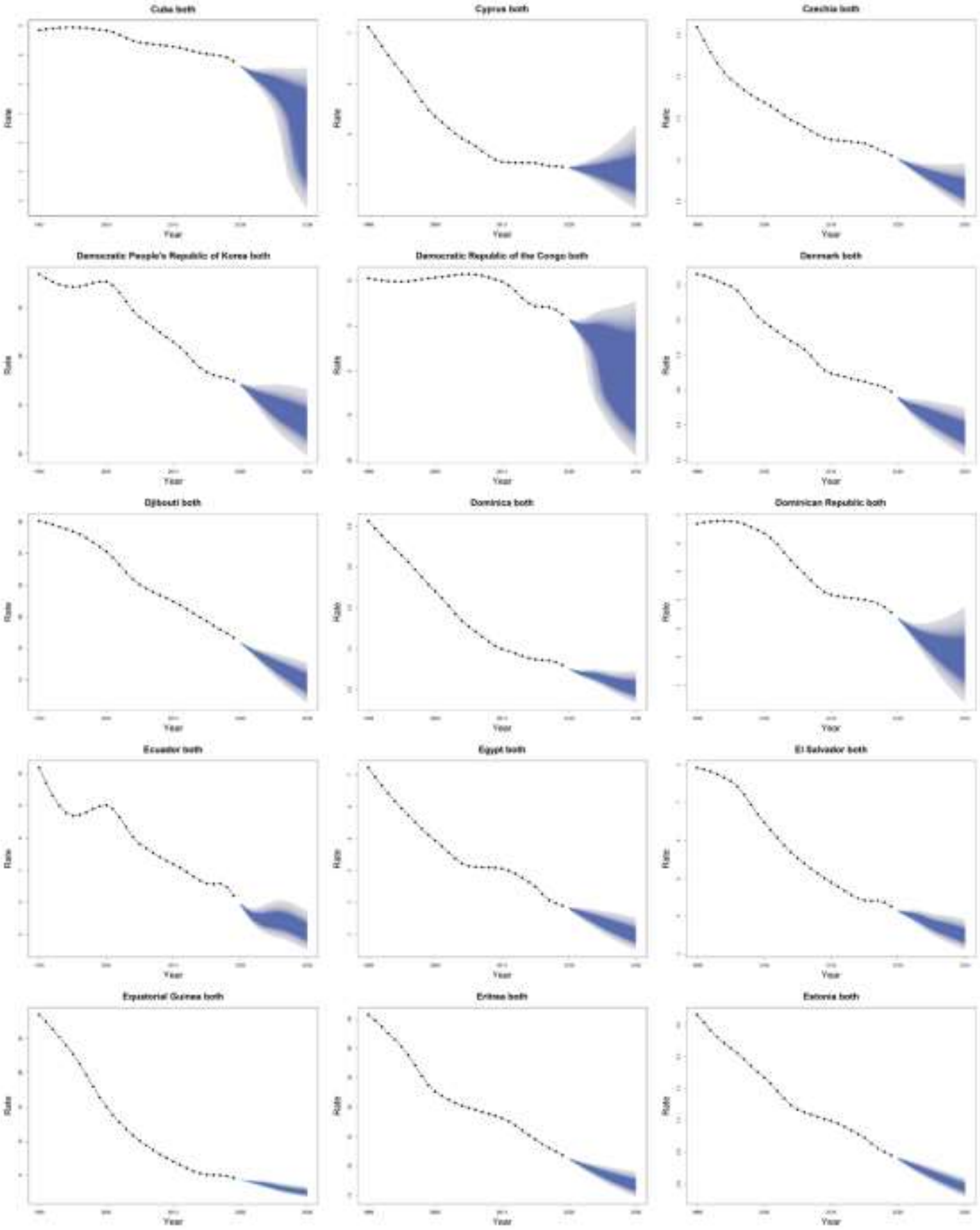
Cuba both
Cyprus both
Czechia both
Democratic People's Republic of Korea both
Democratic Republic of the Congo both
Denmark both
Djibouti both
Dominica both
Dominican Republic both
Ecuador both
Egypt both
El Salvador both
Equatorial Guinea both
Eritrea both
Estonia both
Rate
Year

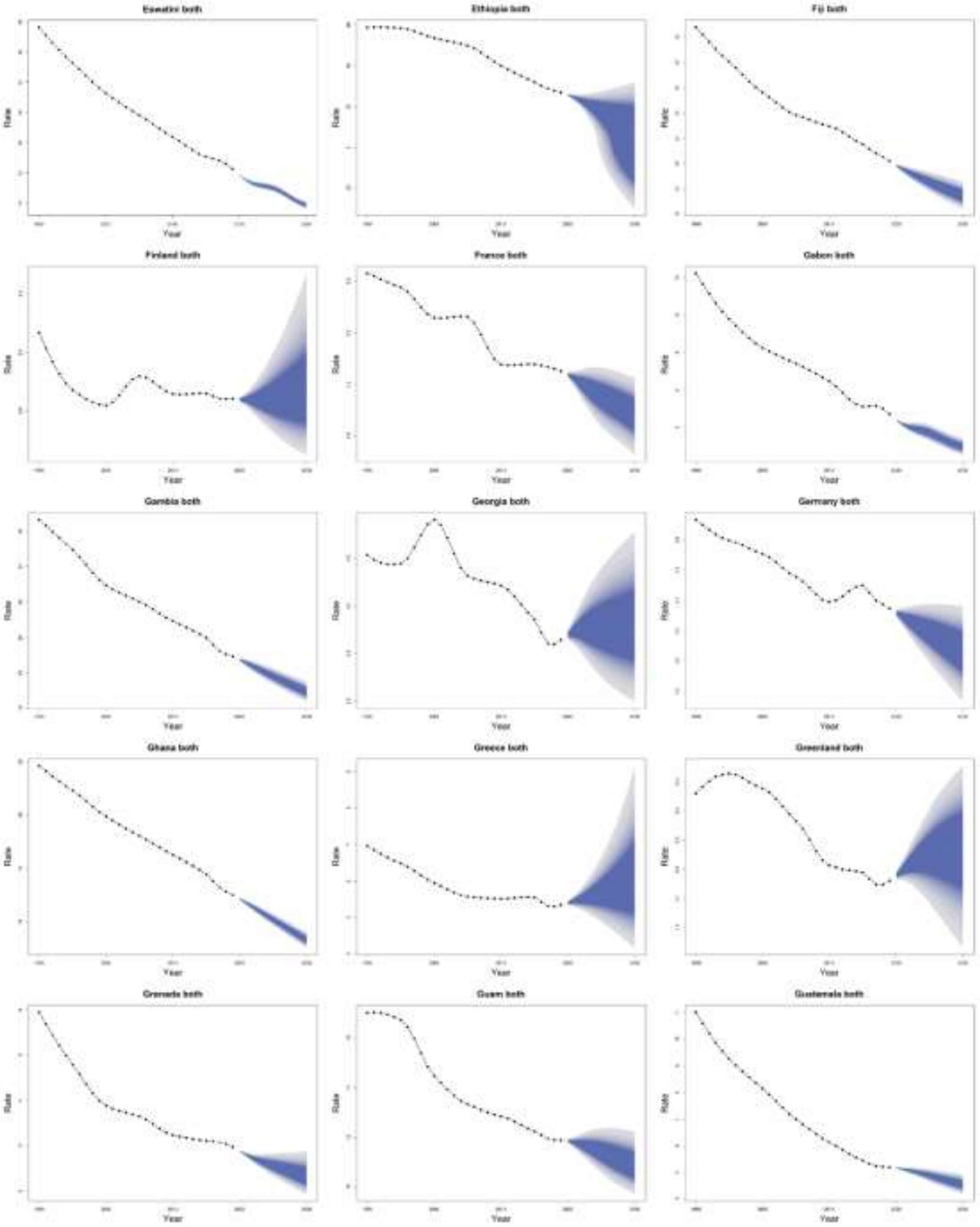
Eswatini both
Ethiopia both
Fiji both
Finland both
France both
Gabon both
Gambia both
Georgia both
Germany both
Ghana both
Greece both
Greenland both
Grenada both
Guam both
Guatemala both
Rate
Year

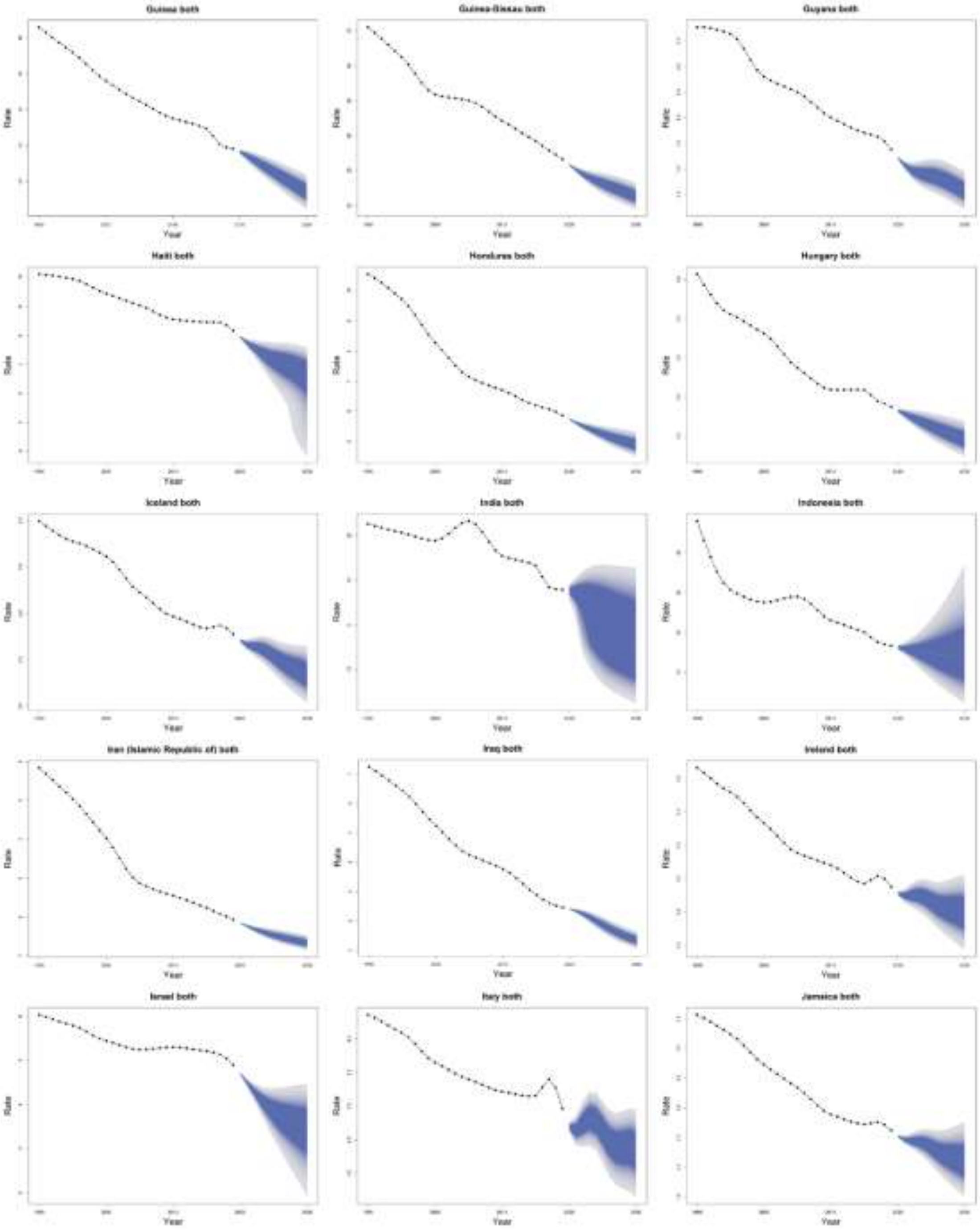

Guinea both
Guinea-Bissau both
Guyana both
Haiti both
Honduras both
Hungary both
Iceland both
India both
Indonesia both
Iran (Islamic Republic of) both
Iraq both
Ireland both
Israel both
Italy both
Jamaica both
Rate
Year

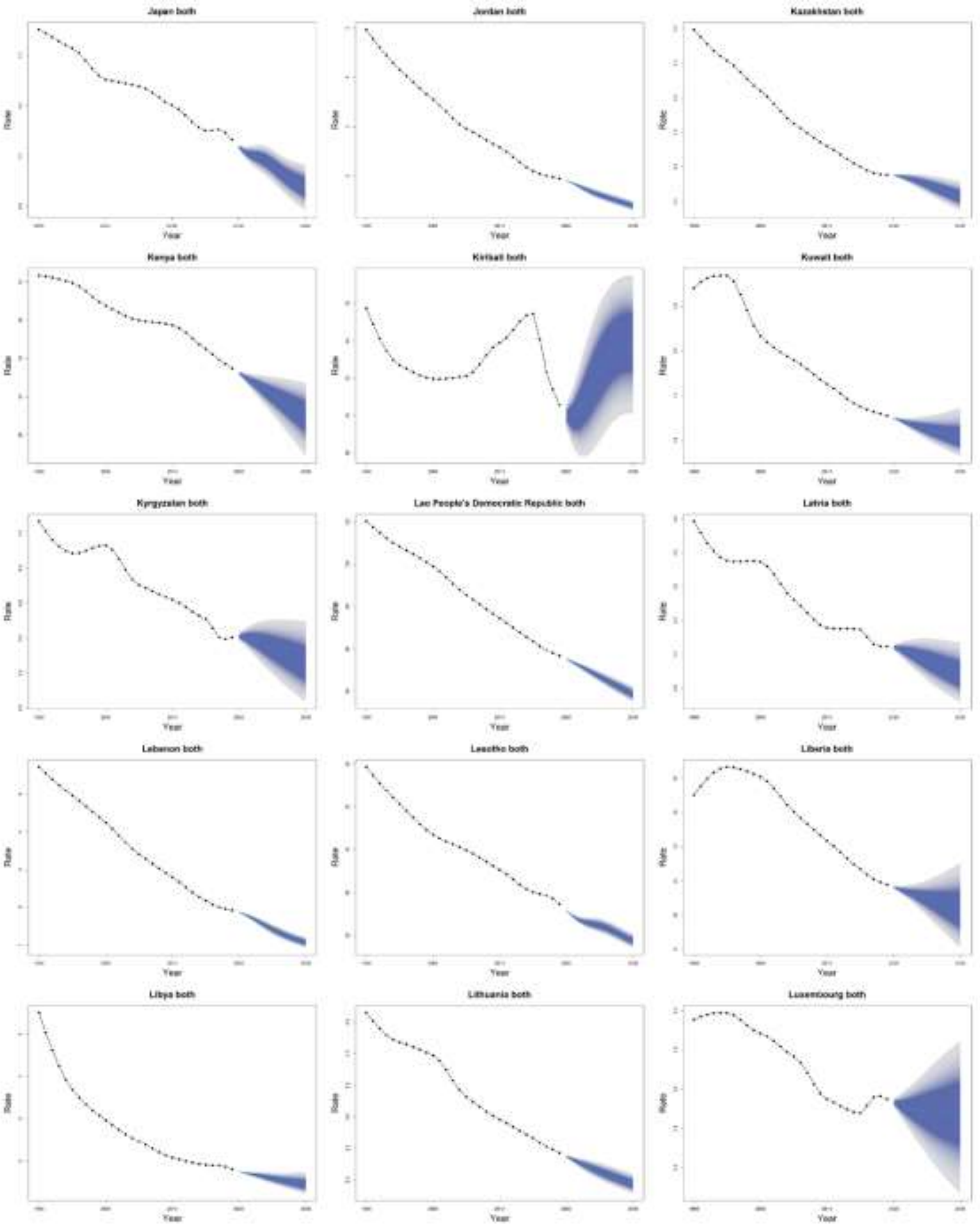
Japan both
Jordan both
Kazakhstan both
Kenya both
Kiribati both
Kuwait both
Kyrgyzstan both
Lao People's Democratic Republic both
Latvia both
Lebanon both
Lesotho both
Liberia both
Libya both
Lithuania both
Luxembourg both
Rate
Year

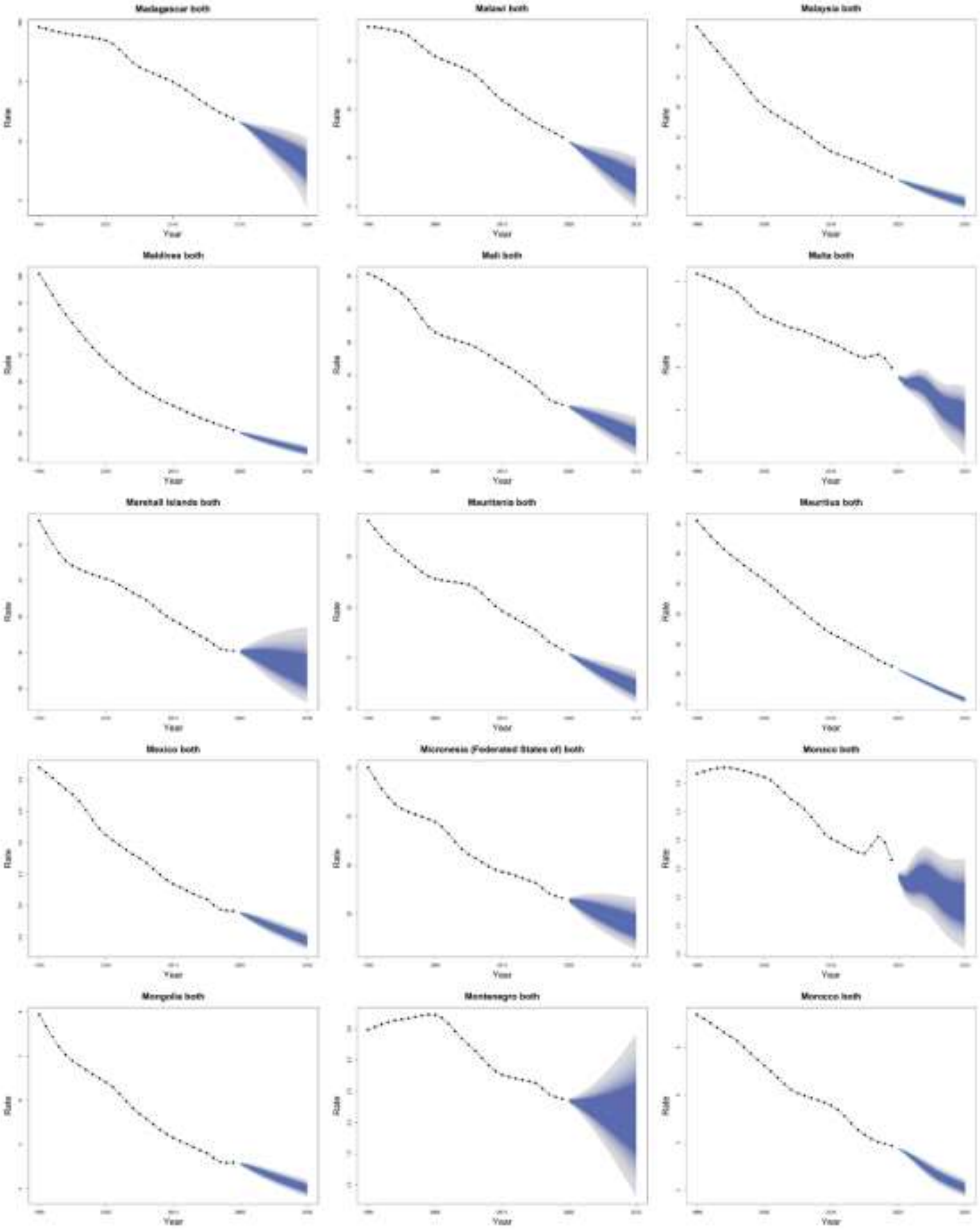
Madagascar both
Malawi both
Malaysia both
Maldives both
Mali both
Malta both
Marshall Islands both
Mauritania both
Mauritius both
Mexico both
Micronesia (Federated States of) both
Monaco both
Mongolia both
Montenegro both
Morocco both
Rate
Year

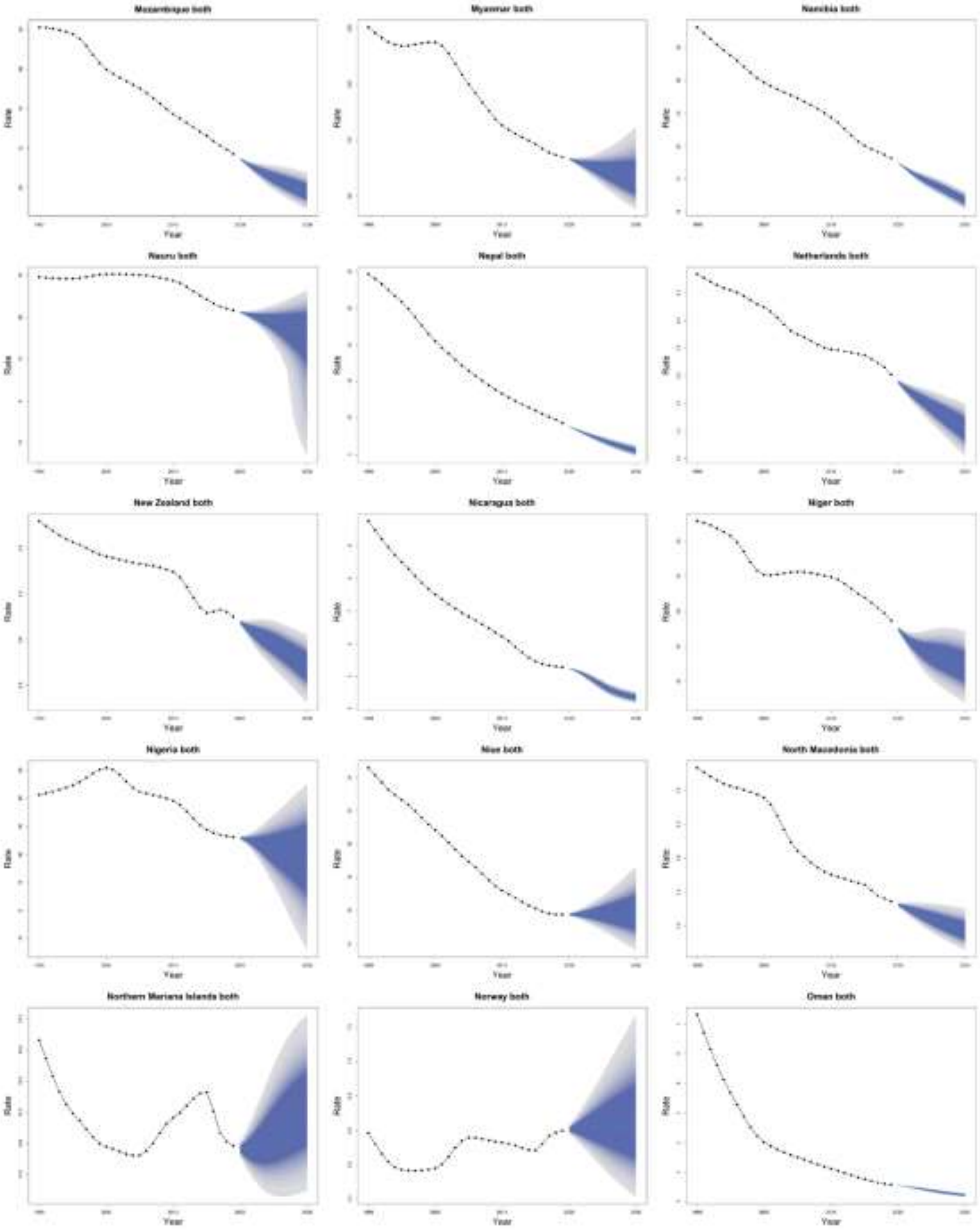
Mozambique both
Myanmar both
Namibia both
Nauru both
Nepal both
Netherlands both
New Zealand both
Nicaragua both
Niger both
Nigeria both
Niue both
North Macedonia both
Northern Mariana Islands both
Norway both
Oman both
Rate
Year

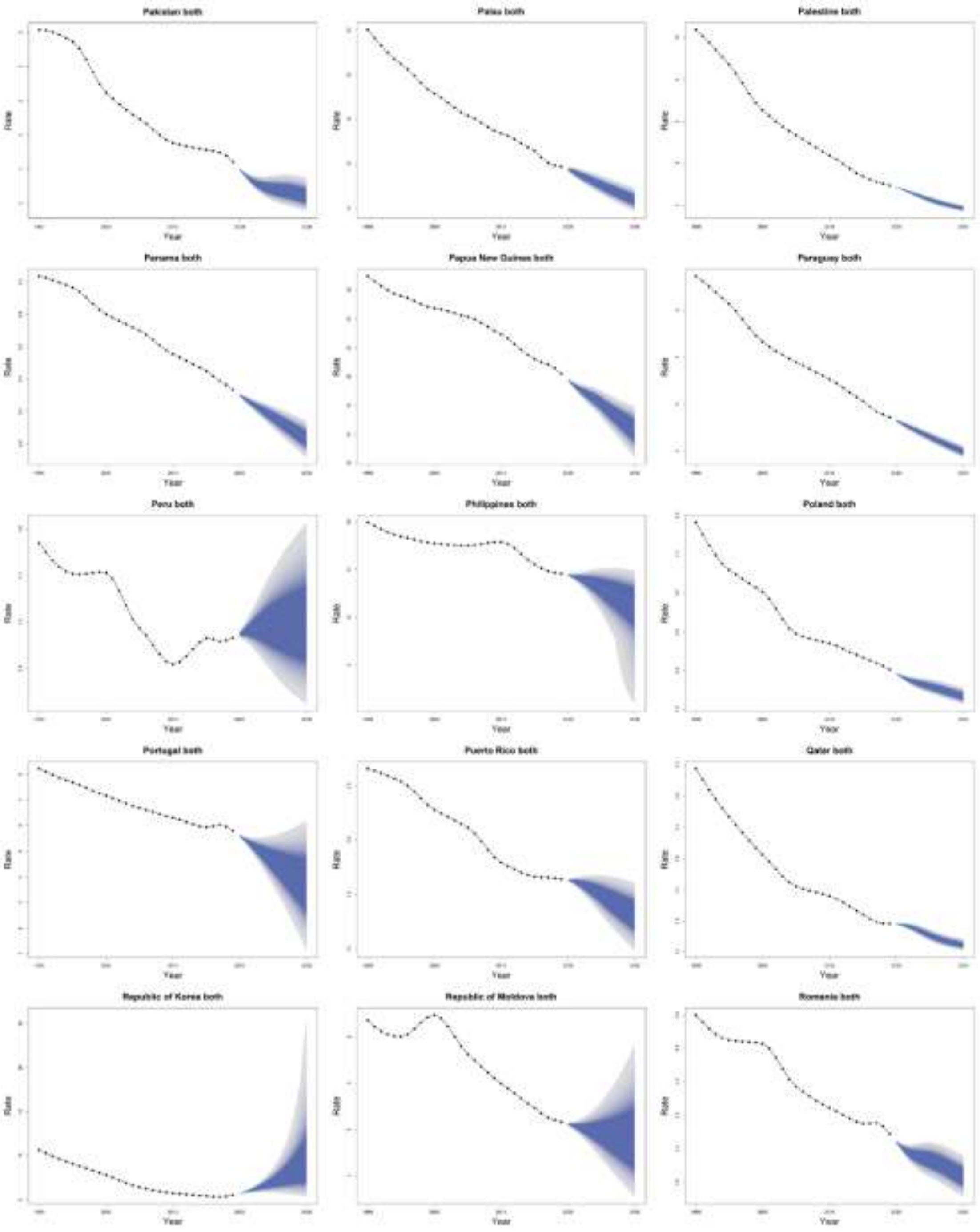
Pakistan both
Palau both
Palestine both
Panama both
Papua New Guinea both
Paraguay both
Peru both
Philippines both
Poland both
Portugal both
Puerto Rico both
Qatar both
Republic of Korea both
Republic of Moldova both
Romania both
Rate
Year

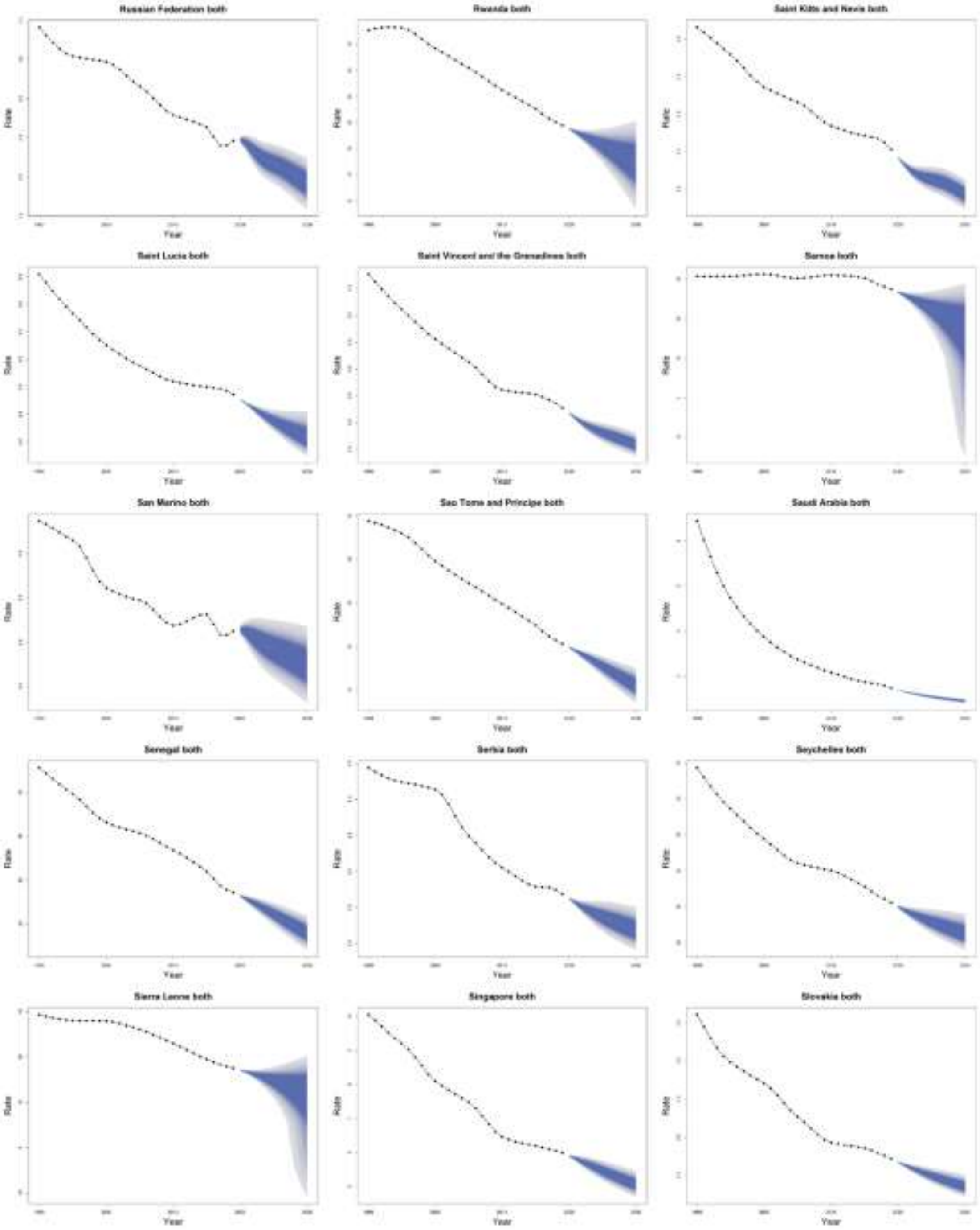
Russian Federation both
Rwanda both
Saint Kitts and Nevis both
Saint Lucia both
Saint Vincent and the Grenadines both
Samoa both
San Marino both
Sao Tome and Principe both
Saudi Arabia both
Senegal both
Serbia both
Seychelles both
Sierra Leone both
Singapore both
Slovakia both
Rate
Year

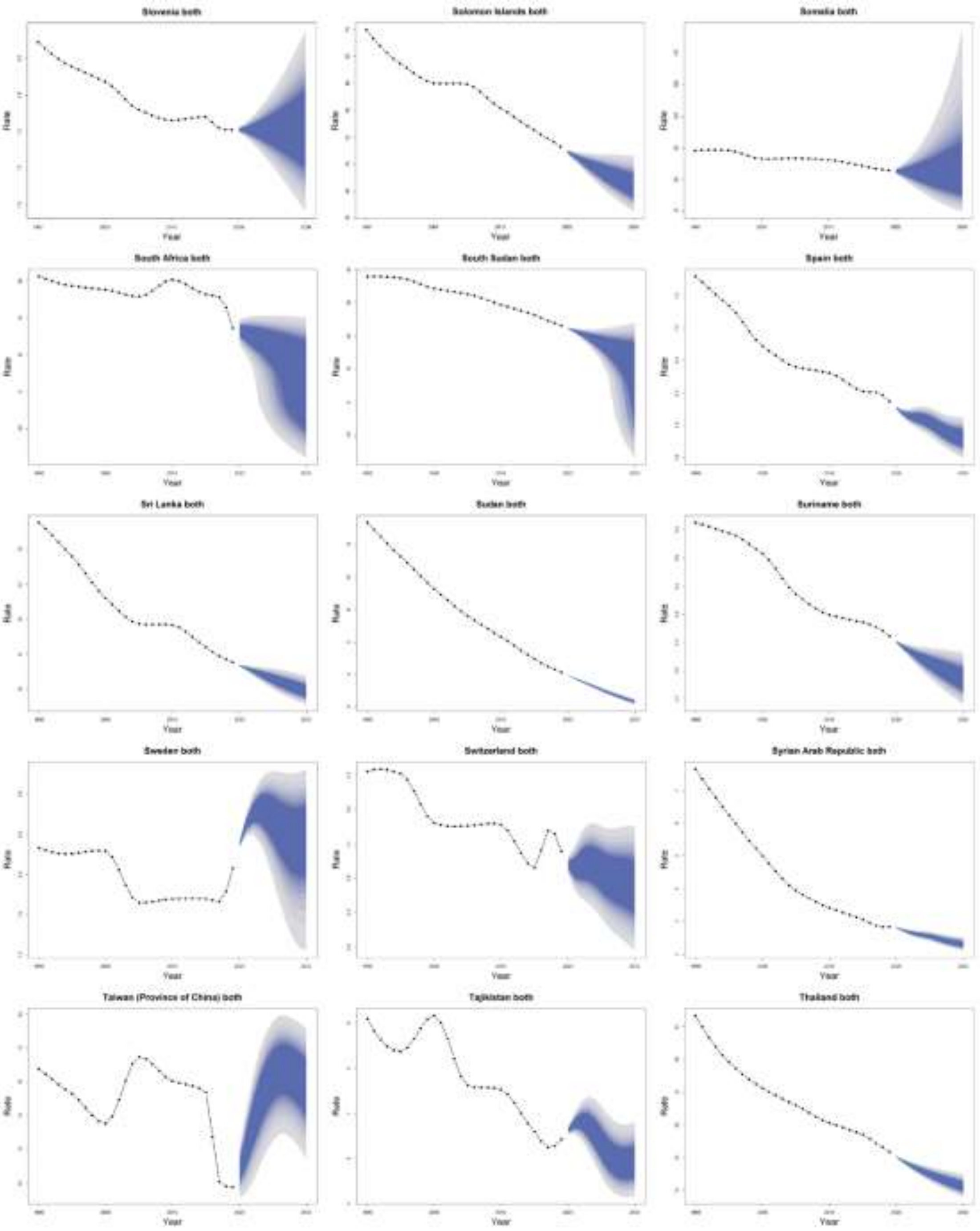
Slovenia both
Solomon Islands both
Somalia both
South Africa both
South Sudan both
Spain both
Sri Lanka both
Sudan both
Suriname both
Sweden both
Switzerland both
Syrian Arab Republic both
Taiwan (Province of China) both
Tajikistan both
Thailand both
Rate
Year

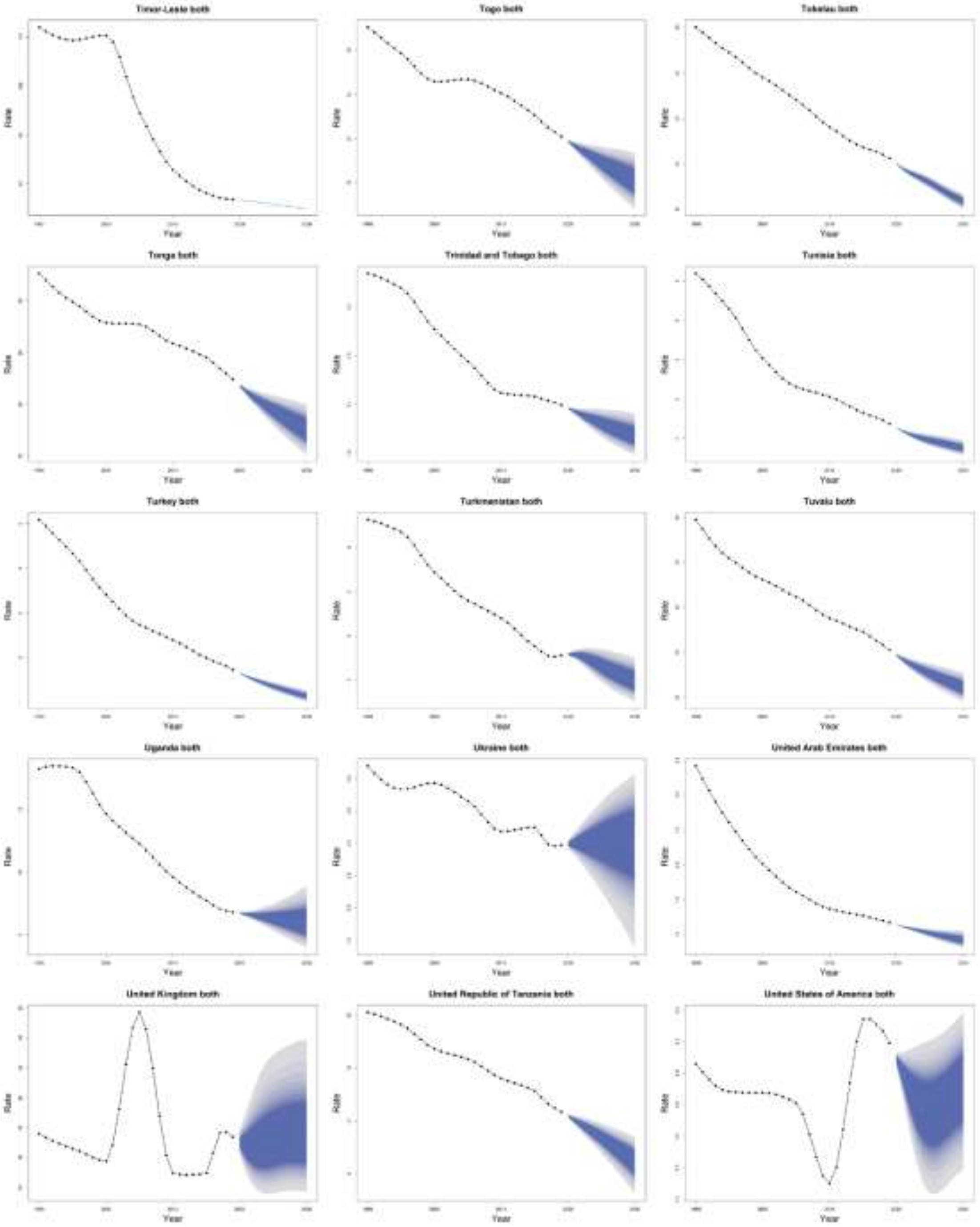
Timor-Leste both
Togo both
Tokelau both
Tonga both
Trinidad and Tobago both
Tunisia both
Turkey both
Turkmenistan both
Tuvalu both
Uganda both
Ukraine both
United Arab Emirates both
United Kingdom both
United Republic of Tanzania both
United States of America both
Year
Rate

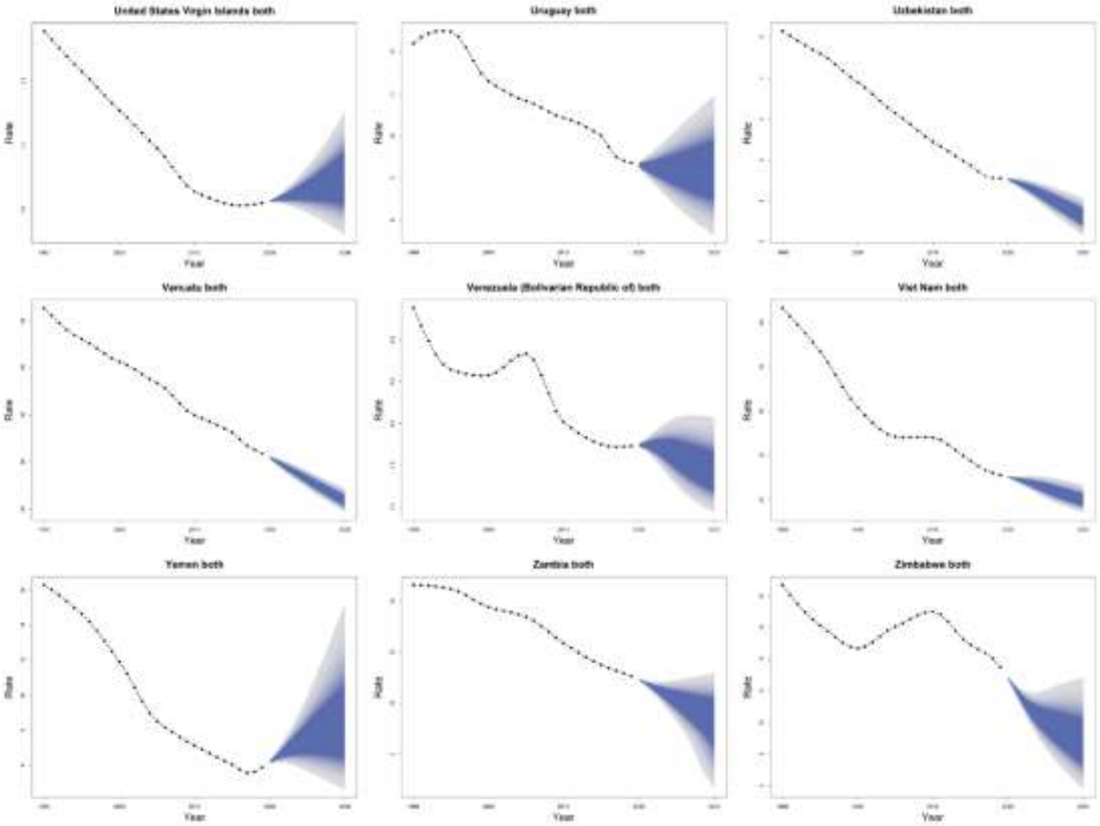

Figure S41. Trends in prevalence rates of 204 countries and territories from 1990 to 2030 for both sexes by ARIMA model.

Supplementary tables:

Supplementary Table 1. The prevalence counts and YLDs for complete hearing loss caused by congenital birth defects in children younger than 5 years in 1990 for both sexes, and percentage change of counts by Global Burden of Disease regions from 1990 to 2019.

	Prevalence (95%Uncertainty Interval)			YLD (95%Uncertainty Interval)		
	Counts	Rates per 100,000 population (95%UI)	Percentage change in counts per 100,000 p opulation (95%UI) (%)	Counts	Rates per 100,00 0 population (9 5%UI)	Percentage change in c ounts per 100,000 pop ulation(95%UI) (%)
Global	178734 (71322 to 367673)	28.3 (11.3 to 58.2)	-43 (-49 to -38.3)	37657 (13816 to 80294)	6 (2.2 to 12.7)	-42.6 (-49.8 to -36.9)
Male	99676 (39802 to 202569)	30.6 (12.2 to 62.3)	-44.1 (-49.9 to -39.5)	20971 (7732 to 44010)	6.4 (2.4 to 13.5)	-43.6 (-51.3 to -36.9)
Female	79058 (31553 to 165734)	25.8 (10.3 to 54.0)	-41.2 (-47.9 to -36.7)	16686 (6066 to 36010)	5.4 (2.0 to 11.7)	-41.3 (-49.5 to -34.6)
High SDI	4090 (1225 to 10550)	7.1 (2.1 to 18.3)	-26.9 (-31.9 to -23.4)	877 (233 to 2354)	1.5 (0.4 to 4.1)	-26.9 (-33 to -22.8)
High-middle SDI	20953 (8216 to 46190)	20 (7.9 to 44.2)	-56.7 (-62.4 to -52.4)	4446 (1572 to 9743)	4.3 (1.5 to 9.3)	-56.4 (-66.3 to -47.7)
Middle SDI	62487 (25647 to 126652)	30.5 (12.5 to 61.8)	-55 (-60.7 to -50.8)	13230 (4746 to 28116)	6.5 (2.3 to 13.7)	-54.7 (-63.2 to -47.3)
Low-middle SDI	55101 (21974 to 113011)	32.8 (13.1 to 67.2)	-56 (-62.3 to -50.9)	11551 (4334 to 24268)	6.9 (2.6 to 14.4)	-55.3 (-63.5 to -48)
Low SDI	35990 (14243 to 72065)	37.3 (14.7 to 74.6)	3.9 (-4.7 to 10.8)	7530 (2751 to 15654)	7.8 (2.8 to 16.2)	4.4 (-9.3 to 16.6)
Andean Latin Ame	233 (59 to 650)	4.3 (1.1 to 11.9)	-18.3 (-40.7 to -8)	50 (12 to 138)	0.9 (0.2 to 2.5)	-18.3 (-40.7 to -8)

rica						
Australasia	78 (19 to 213)	5.1 (1.3 to 13.8)	-9.1 (-30.9 to 6.4)	17 (4 to 46)	1.1 (0.3 to 3)	-9.2 (-30.9 to 6.4)
Caribbean	260 (71 to 646)	6.3 (1.7 to 15.6)	-33.3 (-50.6 to -23.9)	56 (15 to 150)	1.3 (0.4 to 3.6)	-33.3 (-50.6 to -23.9)
Central Asia	671 (185 to 1794)	7.1 (2 to 18.9)	-36 (-49.5 to -26.9)	144 (36 to 395)	1.5 (0.4 to 4.2)	-36 (-49.5 to -26.9)
Central Europe	384 (91 to 1139)	4.3 (1 to 12.7)	-65.1 (-79.6 to -58.6)	82 (20 to 246)	0.9 (0.2 to 2.8)	-65.1 (-79.6 to -58.6)
Central Latin America	1405 (387 to 3549)	6.1 (1.7 to 15.5)	-45 (-58.7 to -37.1)	301 (80 to 812)	1.3 (0.3 to 3.5)	-45 (-58.7 to -37.1)
Central Sub-Saharan Africa	2228 (843 to 4739)	20.7 (7.8 to 44.1)	7.9 (-13.6 to 27.7)	473 (157 to 1056)	4.4 (1.5 to 9.8)	9 (-27.8 to 58.9)
East Asia	51907 (21080 to 104708)	43.3 (17.6 to 87.4)	-61.5 (-67.3 to -58.1)	10993 (3996 to 23281)	9.2 (3.3 to 19.4)	-61.2 (-70.1 to -53.5)
Eastern Europe	718 (165 to 2227)	4.2 (1 to 12.9)	-54.9 (-70.7 to -48.5)	154 (34 to 461)	0.9 (0.2 to 2.7)	-54.9 (-70.7 to -48.5)
Eastern Sub-Saharan Africa	18124 (7595 to 35433)	50.3 (21.1 to 98.4)	-3.7 (-14.2 to 3.7)	3801 (1442 to 7837)	10.6 (4 to 21.8)	-3 (-19.2 to 13.9)
High-income Asia Pacific	765 (204 to 1998)	7.5 (2 to 19.5)	-52.4 (-63.2 to -46.5)	164 (42 to 443)	1.6 (0.4 to 4.3)	-52.4 (-63.2 to -46.5)
High-income North America	1098 (221 to 3328)	5.1 (1 to 15.5)	-2.2 (-8.4 to 7.3)	236 (45 to 720)	1.1 (0.2 to 3.4)	-2.2 (-8.4 to 7.3)
North Africa and Middle East	4281 (1105 to 12051)	8 (2.1 to 22.6)	-51.8 (-69 to -39.1)	918 (231 to 2654)	1.7 (0.4 to 5)	-51.8 (-69 to -39.1)
Oceania	582 (237 to 1162)	59.2 (24.2 to 118.2)	50 (32.8 to 66.5)	122 (46 to 260)	12.4 (4.6 to 26.5)	51 (-9 to 134.6)
South Asia	34149 (12001 to 77189)	21.1 (7.4 to 47.7)	-64.5 (-76.5 to -55.4)	7149 (2379 to 16395)	4.4 (1.5 to 10.1)	-63.7 (-76.6 to -52.6)
Southeast Asia	40160 (17134 to 78343)	67.5 (28.8 to 131.7)	-52.8 (-59 to -48.5)	8449 (3322 to 17292)	14.2 (5.6 to 29.1)	-52.4 (-61.3 to -44.9)
Southern Latin America	618 (186 to 1530)	12 (3.6 to 29.7)	-31.5 (-42.1 to -23.3)	133 (38 to 327)	2.6 (0.7 to 6.3)	-31.5 (-42.1 to -23.3)

Southern Sub-Saharan Africa	3572 (1486 to 7064)	49.8 (20.7 to 98.5)	-37.8 (-44.5 to -31.5)	757 (284 to 1592)	10.6 (4 to 22.2)	-37.6 (-51.2 to -24.1)
Tropical Latin America	1056 (319 to 2521)	6 (1.8 to 14.4)	-46.6 (-55.6 to -41.1)	226 (67 to 551)	1.3 (0.4 to 3.1)	-46.6 (-55.6 to -41.1)
Western Europe	1706 (474 to 4491)	7.4 (2.1 to 19.6)	-14.3 (-20.5 to -1.1)	365 (97 to 1014)	1.6 (0.4 to 4.4)	-14.3 (-20.9 to -1.5)
Western Sub-Saharan Africa	14737 (6189 to 28853)	41.3 (17.3 to 80.9)	34.6 (24 to 43.9)	3069 (1155 to 6532)	8.6 (3.2 to 18.3)	35 (13.8 to 53.4)

YLD= years lived with disability. UI= uncertainty interval.

Supplementary Table 2. The prevalence and YLD for complete hearing loss caused by congenital birth defects in children younger than 5 years for both sexes in 2019, and percentage change of rates by 204 countries, 1990-2019.

	Prevalence (95% Uncertainty Interval)			YLD (95% Uncertainty Interval)		
	Counts	Rates per 100,000 population (95%UI)	Percentage change in Rates per 100,000 population (95%UI)	Counts	Rates per 100,000 population (95%UI)	Percentage change in rates per 100,000 population(95%UI)
Afghanistan	532 (127 to 1524)	8 (1.9 to 22.9)	-50.3 (-66.4 to -38.9)	114 (25 to 330)	1.7 (0.4 to 5)	-50.2 (-66.4 to -38.9)
Albania	6 (1 to 18)	3.6 (0.8 to 10.9)	-43 (-61.8 to -30.4)	1 (0 to 4)	0.8 (0.2 to 2.3)	-43 (-61.8 to -30.4)
Algeria	110 (21 to 368)	2.6 (0.5 to 8.6)	-60.9 (-79.6 to -47.4)	24 (4 to 80)	0.6 (0.1 to 1.9)	-60.9 (-79.6 to -47.4)
American Samoa	1 (0 to 2)	16.7 (6 to 36.9)	-27.8 (-40 to -18.7)	0 (0 to 0)	3.6 (1.2 to 8.2)	-25.7 (-55 to 32.6)
Andorra	0 (0 to 0)	3.4 (0.6 to 11.1)	-9.3 (-33.8 to 5.4)	0 (0 to 0)	0.7 (0.1 to 2.4)	-9.2 (-33.8 to 5.4)
Angola	484 (164 to 1139)	9.4 (3.2 to 22.1)	-58.1 (-68.6 to -48)	104 (33 to 248)	2 (0.6 to 4.8)	-58 (-76.4 to -18.9)
Antigua and Barbuda	0 (0 to 0)	2.1 (0.4 to 6.3)	-38.7 (-58.7 to -28.2)	0 (0 to 0)	0.4 (0.1 to 1.5)	-38.7 (-58.7 to -28.2)
Argentina	314 (87 to 827)	9.1 (2.5 to 23.9)	-24.8 (-37.4 to -13.6)	67 (19 to 182)	1.9 (0.5 to 5.3)	-24.8 (-37.4 to -13.6)
Armenia	8 (2 to 23)	3.7 (0.8 to 11.3)	-44.2 (-63.5 to -30.7)	2 (0 to 5)	0.8 (0.2 to 2.3)	-44.1 (-63.5 to -30.7)
Australia	62 (13 to 180)	4 (0.9 to 11.8)	-23 (-42.2 to -8.3)	13 (3 to 38)	0.9 (0.2 to 2.5)	-23.1 (-42.2 to -8.3)
Austria	15 (3 to 48)	3.5 (0.7 to 10.8)	-20.5 (-40.1 to -4.2)	3 (1 to 10)	0.7 (0.1 to 2.4)	-20.5 (-40.1 to -4.2)
Azerbaijan	30 (7 to 91)	3.9 (0.9 to 12)	-31.4 (-47.6 to -20.5)	6 (1 to 19)	0.8 (0.2 to 2.5)	-31.4 (-47.6 to -20.5)
Bahamas	0 (0 to 1)	1.7 (0.3 to 5.6)	-31 (-46.5 to -17.6)	0 (0 to 0)	0.4 (0.1 to 1.2)	-31 (-46.5 to -17.6)
Bahrain	1 (0 to 5)	1.9 (0.3 to 6.7)	-54.7 (-77.9 to -41.3)	0 (0 to 1)	0.4 (0.1 to 1.4)	-54.8 (-77.9 to -41.3)
Bangladesh	1343 (362 to 3608)	9.8 (2.6 to 26.2)	-59.9 (-75.1 to -49.8)	288 (79 to 765)	2.1 (0.6 to 5.6)	-58.8 (-79.3 to -15.3)

Barbados	0 (0 to 1)	2.1 (0.4 to 6.8)	-29.5 (-57.3 to -16.7)	0 (0 to 0)	0.5 (0.1 to 1.5)	-29.5 (-57.3 to -16.7)
Belarus	20 (4 to 64)	3.5 (0.7 to 11.3)	-43.9 (-67.3 to -31)	4 (1 to 13)	0.7 (0.1 to 2.4)	-44 (-67.3 to -31)
Belgium	26 (5 to 80)	4.2 (0.8 to 13)	-23.8 (-38.2 to -11.3)	6 (1 to 17)	0.9 (0.2 to 2.7)	-23.8 (-38.2 to -11.3)
Belize	1 (0 to 4)	3.5 (0.8 to 10.1)	-51.9 (-66.7 to -38.8)	0 (0 to 1)	0.8 (0.2 to 2.2)	-51.9 (-66.7 to -38.8)
Benin	540 (201 to 1127)	24.3 (9 to 50.6)	-39.8 (-48.3 to -30.2)	116 (29 to 259)	5.2 (1.3 to 11.6)	-38.3 (-71.2 to 24.2)
Bermuda	0 (0 to 0)	1.6 (0.3 to 5.2)	-37.1 (-59.2 to -25)	0 (0 to 0)	0.3 (0.1 to 1.1)	-37.2 (-59.2 to -25)
Bhutan	7 (2 to 18)	11.4 (3.3 to 28.2)	-55.3 (-69.6 to -44.9)	2 (0 to 4)	2.4 (0.7 to 6.2)	-53.8 (-75.3 to -7.2)
Bolivia (Plurinational State of)	54 (11 to 166)	3.6 (0.7 to 11)	-33.5 (-48.9 to -22.8)	12 (2 to 36)	0.8 (0.2 to 2.4)	-33.5 (-48.9 to -22.8)
Bosnia and Herzegovina	5 (1 to 15)	3.1 (0.7 to 10)	-51.6 (-74.2 to -37.7)	1 (0 to 3)	0.7 (0.1 to 2)	-51.6 (-74.2 to -37.7)
Botswana	40 (15 to 88)	17 (6.4 to 37.1)	-57.3 (-67 to -49.5)	9 (3 to 19)	3.6 (1.3 to 8)	-56.6 (-73.8 to -27)
Brazil	540 (142 to 1339)	3.5 (0.9 to 8.6)	-41.8 (-51.5 to -35.7)	115 (30 to 300)	0.7 (0.2 to 1.9)	-41.8 (-51.5 to -35.7)
Brunei Darussalam	2 (1 to 6)	7 (1.8 to 19)	-35.2 (-49.2 to -25.2)	0 (0 to 1)	1.5 (0.4 to 4.1)	-35.2 (-49.2 to -25.2)
Bulgaria	8 (2 to 28)	2.7 (0.5 to 9)	-38.9 (-63.2 to -27.9)	2 (0 to 6)	0.6 (0.1 to 1.9)	-38.9 (-63.2 to -27.9)
Burkina Faso	1421 (559 to 2972)	35.1 (13.8 to 73.5)	-34.4 (-44.4 to -25.7)	294 (88 to 666)	7.3 (2.2 to 16.5)	-34.8 (-67 to 20.8)
Burundi	667 (262 to 1383)	32.1 (12.6 to 66.5)	-29.3 (-39.4 to -20)	140 (41 to 311)	6.7 (2 to 15)	-29.8 (-64.6 to 30.2)
Cabo Verde	8 (3 to 16)	14.2 (5.1 to 30.7)	-52.4 (-63.5 to -44.1)	2 (1 to 4)	3 (1 to 6.7)	-51.9 (-70.6 to -10.1)
Cambodia	1044 (434 to 2069)	59.1 (24.6 to 117.1)	-52.2 (-60.4 to -45.6)	220 (73 to 479)	12.5 (4.2 to 27.1)	-51.8 (-71.3 to -24.5)
Cameroon	723 (262 to 1561)	17.2 (6.2 to 37.1)	-43.7 (-53.7 to -35.4)	155 (52 to 340)	3.7 (1.2 to 8.1)	-42.6 (-63.5 to 6.6)
Canada	85 (17 to 267)	4.4 (0.9 to 13.9)	-21.3 (-44.6 to -8.7)	18 (3 to 56)	0.9 (0.2 to 2.9)	-21.3 (-44.6 to -8.7)
Central African Republic	145 (52 to	17.3 (6.2 to 36.9)	-31.1 (-41.9 to -20.9)	31 (10 to 72)	3.7 (1.2 to 8.5)	-29.3 (-58.8 to 42.6)

	311)					
Chad	1236 (468 to 2527)	37.1 (14 to 75.8)	-36.2 (-47.7 to -27)	258 (73 to 592)	7.7 (2.2 to 17.8)	-36.5 (-66.9 to 5.7)
Chile	88 (22 to 237)	7.6 (1.9 to 20.4)	-35.6 (-52.3 to -24.8)	19 (5 to 51)	1.6 (0.4 to 4.4)	-35.6 (-52.3 to -24.8)
China	19254 (7406 to 41555)	23.6 (9.1 to 51)	-45.8 (-54.1 to -40.8)	4115 (1394 to 9038)	5 (1.7 to 11.1)	-45.3 (-58.1 to -34)
Colombia	133 (32 to 358)	3.5 (0.8 to 9.4)	-42.2 (-55.7 to -31.5)	28 (7 to 81)	0.7 (0.2 to 2.1)	-42.2 (-55.7 to -31.5)
Comoros	14 (5 to 29)	17.8 (6.9 to 37.3)	-47.1 (-58.4 to -38.9)	3 (1 to 6)	3.8 (1.3 to 8.3)	-46.3 (-65.1 to -9.7)
Congo	42 (14 to 101)	6.1 (2 to 14.5)	-55.3 (-66.7 to -46.1)	9 (3 to 22)	1.3 (0.4 to 3.2)	-55.3 (-66.7 to -46.1)
Cook Islands	0 (0 to 0)	13.5 (4.9 to 30.2)	-33.6 (-44.7 to -24.6)	0 (0 to 0)	2.9 (1 to 6.5)	-33.2 (-59.5 to 20.2)
Costa Rica	10 (2 to 27)	2.9 (0.6 to 8.1)	-39.6 (-56.1 to -27.4)	2 (0 to 6)	0.6 (0.1 to 1.9)	-39.6 (-56.1 to -27.4)
Côte d'Ivoire	818 (303 to 1717)	20.4 (7.6 to 42.8)	-40.4 (-50.5 to -31.2)	171 (50 to 380)	4.3 (1.2 to 9.5)	-40 (-69.7 to 18.7)
Croatia	4 (1 to 15)	2.4 (0.5 to 8)	-35.9 (-65 to -20.7)	1 (0 to 3)	0.5 (0.1 to 1.7)	-35.9 (-65 to -20.7)
Cuba	16 (3 to 45)	2.8 (0.6 to 8.1)	-27.6 (-52.8 to -14.2)	3 (1 to 10)	0.6 (0.1 to 1.8)	-27.5 (-52.8 to -14.2)
Cyprus	3 (1 to 10)	4.3 (0.9 to 13.3)	-38.9 (-64.4 to -26.9)	1 (0 to 2)	0.9 (0.2 to 2.9)	-38.9 (-64.4 to -26.9)
Czechia	12 (2 to 40)	2.1 (0.4 to 7.1)	-42.9 (-67.4 to -30.2)	3 (0 to 9)	0.4 (0.1 to 1.5)	-42.9 (-67.4 to -30.2)
Democratic People's Republic of Korea	624 (244 to 1278)	37.5 (14.7 to 76.7)	-22.5 (-35.3 to -9.6)	131 (40 to 292)	7.9 (2.4 to 17.6)	-23.3 (-60.8 to 39.4)
Democratic Republic of the Congo	1714 (620 to 3868)	12.6 (4.5 to 28.4)	-38.7 (-51.2 to -26.4)	368 (120 to 844)	2.7 (0.9 to 6.2)	-37.9 (-61.4 to 5.5)
Denmark	11 (2 to 36)	3.6 (0.7 to 11.5)	-15.7 (-29.3 to -4.5)	2 (0 to 8)	0.8 (0.1 to 2.6)	-15.8 (-29.3 to -4.5)
Djibouti	27 (10 to 57)	16.7 (6.4 to 36)	-52.4 (-61.6 to -44.3)	6 (2 to 13)	3.6 (1.3 to 8)	-51.6 (-69.7 to -16.9)
Dominica	0 (0 to 0)	2.3 (0.5 to 6.9)	-43.5 (-64.7 to -31.2)	0 (0 to 0)	0.5 (0.1 to 1.6)	-43.4 (-64.7 to -31.2)
Dominican Republic	39 (8 to 110)	3.6 (0.8 to 10)	-46.5 (-67.2 to -32.8)	8 (2 to 24)	0.8 (0.2 to 2.2)	-46.5 (-67.2 to -32.8)

Ecuador	106 (25 to 299)	6.2 (1.5 to 17.5)	-38.9 (-57.5 to -27.5)	23 (5 to 64)	1.3 (0.3 to 3.7)	-39 (-57.5 to -27.5)
Egypt	313 (59 to 1072)	2.9 (0.5 to 9.9)	-60 (-80.3 to -45.6)	67 (11 to 237)	0.6 (0.1 to 2.2)	-60 (-80.3 to -45.6)
El Salvador	24 (6 to 67)	4.3 (1 to 11.7)	-46.2 (-62.8 to -35.3)	5 (1 to 15)	0.9 (0.2 to 2.6)	-46.2 (-62.8 to -35.3)
Equatorial Guinea	9 (3 to 22)	4.6 (1.5 to 11.6)	-83.7 (-89.7 to -78.8)	2 (1 to 5)	1 (0.3 to 2.6)	-83.3 (-91.4 to -68.2)
Eritrea	203 (78 to 422)	21.8 (8.4 to 45.3)	-52.2 (-60.7 to -44)	43 (10 to 107)	4.6 (1.1 to 11.5)	-51.4 (-79.3 to -3.7)
Estonia	2 (0 to 6)	2.5 (0.5 to 8)	-47.4 (-70 to -35.2)	0 (0 to 1)	0.5 (0.1 to 1.8)	-47.4 (-70 to -35.2)
Eswatini	29 (11 to 64)	20.7 (7.7 to 45.5)	-53 (-60.8 to -46.1)	6 (2 to 15)	4.4 (1.2 to 10.5)	-52.4 (-78.4 to -7.1)
Ethiopia	4510 (1728 to 9183)	27 (10.4 to 55.1)	-53.9 (-60.6 to -48.7)	949 (325 to 2086)	5.7 (1.9 to 12.5)	-53.6 (-68.1 to -37.9)
Fiji	18 (7 to 38)	20.2 (7.5 to 43.1)	-34.3 (-44.5 to -25.6)	4 (1 to 9)	4.3 (1.1 to 10.4)	-33.7 (-67.1 to 38.5)
Finland	6 (1 to 19)	2.1 (0.3 to 7.4)	-21.1 (-46.8 to -6.6)	1 (0 to 4)	0.5 (0.1 to 1.5)	-21.2 (-46.8 to -6.6)
France	169 (33 to 515)	4.6 (0.9 to 14.1)	-16.9 (-32.3 to -3.1)	36 (7 to 112)	1 (0.2 to 3.1)	-16.9 (-32.3 to -3.1)
Gabon	9 (3 to 24)	4.7 (1.5 to 11.7)	-61.5 (-70.5 to -53)	2 (1 to 5)	1 (0.3 to 2.5)	-61.5 (-70.5 to -53)
Gambia	74 (28 to 158)	22.3 (8.4 to 48)	-46.4 (-54.2 to -39.1)	15 (4 to 36)	4.7 (1.2 to 10.8)	-45.5 (-75 to 4.4)
Georgia	9 (2 to 27)	3.6 (0.9 to 11.1)	-19.6 (-37.8 to -4)	2 (0 to 6)	0.8 (0.2 to 2.4)	-19.5 (-37.8 to -4)
Germany	131 (24 to 422)	3.3 (0.6 to 10.8)	-14.9 (-27.5 to -3.2)	28 (5 to 91)	0.7 (0.1 to 2.3)	-14.9 (-27.5 to -3.2)
Ghana	498 (179 to 1094)	12.5 (4.5 to 27.5)	-49 (-58.2 to -40)	107 (36 to 243)	2.7 (0.9 to 6.1)	-47.9 (-68.7 to 5.1)
Greece	24 (5 to 72)	5.3 (1.1 to 15.8)	-23.2 (-44.7 to -10.5)	5 (1 to 16)	1.1 (0.2 to 3.5)	-23.2 (-44.7 to -10.5)
Greenland	0 (0 to 1)	8.3 (2.1 to 22.2)	-15.2 (-33.9 to -0.1)	0 (0 to 0)	1.8 (0.4 to 4.9)	-15.3 (-33.9 to -0.1)
Grenada	0 (0 to 1)	3 (0.6 to 8.5)	-50.1 (-65.8 to -38.4)	0 (0 to 0)	0.6 (0.1 to 1.9)	-50.1 (-65.8 to -38.4)

Guam	2 (1 to 4)	11.9 (4.1 to 27.4)	-30.2 (-43.6 to -19.5)	0 (0 to 1)	2.5 (0.8 to 5.9)	-30.2 (-43.6 to -19.5)
Guatemala	104 (27 to 265)	5.2 (1.3 to 13.3)	-52.8 (-68 to -42.1)	22 (6 to 62)	1.1 (0.3 to 3.1)	-52.8 (-68 to -42.1)
Guinea	633 (242 to 1336)	29.5 (11.3 to 62.3)	-36.4 (-46.6 to -27.5)	132 (38 to 299)	6.2 (1.8 to 13.9)	-36.5 (-71 to 20.4)
Guinea-Bissau	76 (28 to 161)	26.6 (9.8 to 55.9)	-41.6 (-51.3 to -32.9)	16 (4 to 36)	5.5 (1.3 to 12.5)	-41.7 (-76.2 to 14.8)
Guyana	2 (1 to 7)	3.4 (0.8 to 9.3)	-41.3 (-58.5 to -30.7)	1 (0 to 2)	0.7 (0.2 to 2.1)	-41.4 (-58.5 to -30.7)
Haiti	97 (25 to 246)	6.4 (1.6 to 16.1)	-37.8 (-55 to -26.3)	21 (5 to 56)	1.4 (0.3 to 3.7)	-37.9 (-55 to -26.3)
Honduras	66 (17 to 170)	5.9 (1.5 to 15.1)	-44.4 (-63.5 to -34.1)	14 (4 to 38)	1.3 (0.3 to 3.4)	-44.5 (-63.5 to -34.1)
Hungary	10 (2 to 35)	2.4 (0.4 to 8)	-41.7 (-72.4 to -27.8)	2 (0 to 7)	0.5 (0.1 to 1.7)	-41.8 (-72.4 to -27.8)
Iceland	1 (0 to 3)	3.8 (0.7 to 12)	-24.4 (-46.2 to -13)	0 (0 to 1)	0.8 (0.1 to 2.6)	-24.4 (-46.2 to -13)
India	9216 (2728 to 23737)	7.9 (2.3 to 20.3)	-65.1 (-76.8 to -56.1)	1974 (576 to 5132)	1.7 (0.5 to 4.4)	-64.3 (-76.7 to -53)
Indonesia	4603 (1700 to 10029)	23.4 (8.6 to 50.9)	-40.2 (-47.2 to -35)	979 (340 to 2198)	5 (1.7 to 11.2)	-39.7 (-55.2 to -22.9)
Iran (Islamic Republic of)	137 (19 to 516)	1.9 (0.3 to 7.3)	-66.9 (-87.1 to -54.5)	29 (4 to 108)	0.4 (0.1 to 1.5)	-66.9 (-87.1 to -54.5)
Iraq	114 (19 to 394)	2.5 (0.4 to 8.5)	-66.1 (-82.9 to -52.2)	25 (4 to 86)	0.5 (0.1 to 1.9)	-66.2 (-82.9 to -52.2)
Ireland	12 (2 to 38)	3.9 (0.8 to 11.9)	-31.4 (-48.6 to -19.9)	3 (0 to 8)	0.8 (0.2 to 2.6)	-31.4 (-48.6 to -19.9)
Israel	46 (9 to 135)	4.9 (1 to 14.2)	-18.9 (-43.3 to -5.9)	10 (2 to 31)	1 (0.2 to 3.2)	-18.9 (-43.3 to -5.9)
Italy	117 (23 to 370)	5 (1 to 15.7)	-22 (-39.3 to -14.6)	25 (5 to 81)	1.1 (0.2 to 3.4)	-22 (-39.3 to -14.6)
Jamaica	5 (1 to 14)	2.6 (0.6 to 7.5)	-42.5 (-58.3 to -29.6)	1 (0 to 3)	0.6 (0.1 to 1.8)	-42.5 (-58.3 to -29.6)
Japan	223 (51 to 640)	4.7 (1.1 to 13.4)	-19 (-27.4 to -14.5)	48 (11 to 140)	1 (0.2 to 2.9)	-19 (-27.4 to -14.5)

Jordan	23 (3 to 84)	1.9 (0.3 to 7)	-60.9 (-82.5 to -46.3)	5 (1 to 17)	0.4 (0.1 to 1.4)	-60.9 (-82.5 to -46.3)
Kazakhstan	62 (13 to 194)	3.4 (0.7 to 10.6)	-38.3 (-56.3 to -26.8)	13 (3 to 41)	0.7 (0.1 to 2.2)	-38.3 (-56.3 to -26.8)
Kenya	2161 (860 to 4225)	33.7 (13.4 to 65.9)	-26.6 (-30.9 to -23.6)	458 (167 to 944)	7.1 (2.6 to 14.7)	-26.1 (-36.4 to -16.8)
Kiribati	6 (2 to 12)	40.6 (15.6 to 83.2)	-11.3 (-22.5 to 1)	1 (0 to 3)	8.5 (2.6 to 19.5)	-11.6 (-53.5 to 65.7)
Kuwait	4 (1 to 14)	1.3 (0.2 to 4.7)	-52.8 (-70 to -40.9)	1 (0 to 3)	0.3 (0 to 1)	-52.8 (-70 to -40.9)
Kyrgyzstan	41 (11 to 117)	5.5 (1.4 to 15.5)	-23.1 (-37.9 to -13.3)	9 (2 to 26)	1.2 (0.3 to 3.4)	-23.1 (-37.9 to -13.3)
Lao People's Democratic Republic	454 (184 to 891)	56.7 (23 to 111.1)	-52.9 (-60.8 to -45)	97 (31 to 205)	12.1 (3.9 to 25.6)	-52.2 (-72 to -27)
Latvia	3 (1 to 9)	2.6 (0.5 to 8.8)	-41.4 (-70 to -28.4)	1 (0 to 2)	0.6 (0.1 to 1.9)	-41.4 (-70 to -28.4)
Lebanon	10 (1 to 35)	1.9 (0.3 to 7)	-66.5 (-88.3 to -52.2)	2 (0 to 8)	0.4 (0.1 to 1.5)	-66.6 (-88.3 to -52.2)
Lesotho	60 (22 to 126)	27.3 (10.2 to 57.9)	-54 (-63.4 to -46.3)	13 (3 to 30)	5.9 (1.4 to 13.7)	-52.6 (-78.8 to -11)
Liberia	155 (57 to 324)	24.5 (9 to 51.2)	-34.8 (-44.2 to -25.3)	32 (7 to 75)	5.1 (1.2 to 11.8)	-35 (-74.9 to 30.7)
Libya	8 (1 to 28)	1.8 (0.3 to 6.7)	-67 (-87 to -54.1)	2 (0 to 6)	0.4 (0.1 to 1.5)	-67 (-87 to -54.1)
Lithuania	3 (1 to 12)	2.4 (0.5 to 8.1)	-47.8 (-69.2 to -35.1)	1 (0 to 2)	0.5 (0.1 to 1.7)	-47.8 (-69.2 to -35.1)
Luxembourg	1 (0 to 4)	3.5 (0.7 to 11.6)	-10.3 (-26.1 to 4.1)	0 (0 to 1)	0.8 (0.1 to 2.5)	-10.3 (-26.1 to 4.1)
Madagascar	2726 (1166 to 5312)	69.2 (29.6 to 134.8)	-52.8 (-60.4 to -45.9)	578 (191 to 1255)	14.7 (4.8 to 31.9)	-52.2 (-71.4 to -27.4)
Malawi	628 (239 to 1299)	24.3 (9.2 to 50.2)	-48.3 (-58.9 to -39.8)	131 (30 to 308)	5.1 (1.2 to 11.9)	-48.5 (-79.9 to -2.6)
Malaysia	602 (231 to 1260)	23.4 (9 to 49.1)	-51.5 (-61.3 to -43.8)	127 (34 to 296)	5 (1.3 to 11.5)	-50.9 (-81.1 to -0.7)
Maldives	17 (7 to 34)	41.3 (16.8 to 83.5)	-59.2 (-66.4 to -52.9)	4 (1 to 8)	8.6 (2.8 to 19.2)	-58.9 (-78.6 to -31.2)
Mali	1446 (558 to 2982)	35.6 (13.7 to 73.4)	-35.7 (-45.8 to -26.1)	297 (87 to 692)	7.3 (2.1 to 17)	-36.5 (-70.1 to 8)

Malta	1 (0 to 3)	5 (1.1 to 14.4)	-30.5 (-49.8 to -18.4)	0 (0 to 1)	1.1 (0.2 to 3.2)	-30.4 (-49.8 to -18.4)
Marshall Islands	2 (1 to 4)	30.3 (11.7 to 64.2)	-37.5 (-48.2 to -27.8)	0 (0 to 1)	6.4 (1.8 to 14.7)	-37.1 (-69.2 to 22.4)
Mauritania	84 (30 to 184)	15.9 (5.7 to 34.7)	-44.4 (-52.3 to -37.5)	18 (6 to 39)	3.4 (1.1 to 7.4)	-42.5 (-65.3 to 2.4)
Mauritius	17 (7 to 35)	26.3 (10.1 to 54.4)	-47.9 (-57.2 to -39.5)	4 (1 to 8)	5.6 (1.5 to 12.8)	-47.7 (-76.9 to -4)
Mexico	305 (67 to 855)	2.9 (0.6 to 8.2)	-43.9 (-60.3 to -34.1)	65 (14 to 195)	0.6 (0.1 to 1.9)	-43.9 (-60.3 to -34.1)
Micronesia (Federated States of)	3 (1 to 6)	26.6 (9.9 to 57.2)	-33.4 (-42.6 to -24.9)	1 (0 to 1)	5.6 (1.4 to 13)	-32.3 (-72.2 to 37.2)
Monaco	0 (0 to 0)	3.3 (0.5 to 10.9)	-8.3 (-39.8 to 14.1)	0 (0 to 0)	0.7 (0.1 to 2.4)	-8.3 (-39.8 to 14.1)
Mongolia	18 (4 to 54)	4.6 (1.1 to 13.7)	-42.2 (-59.1 to -30.9)	4 (1 to 12)	1 (0.2 to 2.9)	-42.1 (-59.1 to -30.9)
Montenegro	1 (0 to 3)	2.4 (0.5 to 7.8)	-31.7 (-65.2 to -18.1)	0 (0 to 1)	0.5 (0.1 to 1.6)	-31.7 (-65.2 to -18.1)
Morocco	118 (23 to 393)	3.9 (0.7 to 12.9)	-58.9 (-78.9 to -47)	25 (5 to 85)	0.8 (0.1 to 2.8)	-58.9 (-78.9 to -47)
Mozambique	1455 (552 to 2916)	28.5 (10.8 to 57.1)	-52.9 (-61.2 to -46)	308 (90 to 694)	6 (1.8 to 13.6)	-52.2 (-76.8 to -16.1)
Myanmar	4267 (1849 to 8370)	85 (36.8 to 166.8)	-57.7 (-64.6 to -50.3)	898 (332 to 1853)	17.9 (6.6 to 36.9)	-57.4 (-70.7 to -38.7)
Namibia	54 (20 to 118)	18.2 (6.6 to 39.7)	-52.2 (-61 to -43.5)	12 (4 to 25)	3.9 (1.3 to 8.5)	-51.3 (-69.9 to -15.7)
Nauru	0 (0 to 1)	21.7 (8 to 47.3)	-26.7 (-36 to -18)	0 (0 to 0)	4.6 (1.1 to 11.1)	-26.8 (-66.4 to 57.2)
Nepal	278 (65 to 772)	9.3 (2.2 to 25.8)	-68.8 (-83.7 to -58.5)	59 (14 to 165)	2 (0.5 to 5.5)	-67.9 (-85.2 to -40.1)
Netherlands	32 (6 to 101)	3.6 (0.6 to 11.5)	-16.8 (-37.8 to 2.8)	7 (1 to 22)	0.8 (0.1 to 2.5)	-16.8 (-37.8 to 2.8)
New Zealand	10 (2 to 30)	3.3 (0.7 to 10.3)	-24.4 (-53.9 to -13.2)	2 (0 to 6)	0.7 (0.1 to 2.1)	-24.4 (-53.9 to -13.2)
Nicaragua	35 (9 to 93)	5.3 (1.4 to 14)	-46 (-61.5 to -34.4)	7 (2 to 21)	1.1 (0.3 to 3.2)	-46.1 (-61.5 to -34.4)
Niger	2362 (941 to 4738)	48.7 (19.4 to 97.6)	-22.6 (-34.3 to -9.9)	492 (162 to 1064)	10.1 (3.3 to 21.9)	-22.7 (-58.3 to 33.3)

Nigeria	8810 (3486 to 17969)	26.3 (10.4 to 53.6)	-36.2 (-41.3 to -31.9)	1838 (658 to 4025)	5.5 (2 to 12)	-35.8 (-45.8 to -24.7)
Niue	0 (0 to 0)	15.8 (5.5 to 35.7)	-35.9 (-45.7 to -25.9)	0 (0 to 0)	3.4 (1.2 to 7.8)	-34.4 (-61.3 to 30.7)
North Macedonia	3 (1 to 11)	2.9 (0.5 to 9.7)	-40.8 (-70.6 to -26.7)	1 (0 to 2)	0.6 (0.1 to 2)	-40.8 (-70.6 to -26.7)
Northern Mariana Islands	0 (0 to 1)	14.5 (5.2 to 32.1)	-10.5 (-22.1 to 0.1)	0 (0 to 0)	3.1 (1.1 to 6.9)	-10.4 (-22.1 to 0.1)
Norway	18 (4 to 47)	6 (1.4 to 15.8)	0.6 (-6.7 to 16.2)	4 (1 to 11)	1.3 (0.3 to 3.6)	0.6 (-6.7 to 16.2)
Oman	6 (1 to 23)	1.6 (0.2 to 5.8)	-78.3 (-92.3 to -67.7)	1 (0 to 5)	0.3 (0 to 1.2)	-78.3 (-92.3 to -67.7)
Pakistan	1286 (306 to 3856)	4.2 (1 to 12.6)	-47.8 (-62.7 to -36.5)	276 (64 to 800)	0.9 (0.2 to 2.6)	-47.8 (-62.7 to -36.5)
Palau	0 (0 to 0)	15.9 (5.6 to 36.2)	-27.9 (-40 to -18.5)	0 (0 to 0)	3.4 (1.1 to 7.7)	-26.8 (-56.4 to 41.7)
Palestine	18 (3 to 63)	3 (0.5 to 10.1)	-71.5 (-87.3 to -60)	4 (1 to 14)	0.6 (0.1 to 2.2)	-71.5 (-87.3 to -60)
Panama	11 (2 to 32)	2.8 (0.6 to 8.4)	-38.2 (-55.5 to -26.7)	2 (0 to 7)	0.6 (0.1 to 1.9)	-38.2 (-55.5 to -26.7)
Papua New Guinea	732 (291 to 1500)	50.5 (20.1 to 103.6)	-25 (-35 to -14.8)	155 (48 to 354)	10.7 (3.3 to 24.4)	-24.3 (-58.3 to 29)
Paraguay	24 (6 to 59)	3.7 (1 to 9.3)	-44.7 (-59 to -34.6)	5 (1 to 14)	0.8 (0.2 to 2.1)	-44.7 (-59 to -34.6)
Peru	30 (4 to 109)	1 (0.1 to 3.5)	-17.3 (-31.5 to 3.1)	6 (1 to 24)	0.2 (0 to 0.8)	-17.4 (-31.5 to 3.1)
Philippines	4839 (1919 to 9797)	38.2 (15.2 to 77.4)	-36 (-42.5 to -31.8)	1029 (382 to 2187)	8.1 (3 to 17.3)	-35.7 (-45.4 to -27.1)
Poland	38 (7 to 130)	2 (0.4 to 6.8)	-48.5 (-70.8 to -37.3)	8 (1 to 28)	0.4 (0.1 to 1.5)	-48.5 (-70.8 to -37.3)
Portugal	24 (5 to 67)	5.8 (1.2 to 16.3)	-29.5 (-48.1 to -18)	5 (1 to 15)	1.2 (0.3 to 3.6)	-29.5 (-48.1 to -18)
Puerto Rico	2 (0 to 7)	1.6 (0.3 to 5.2)	-38.1 (-58.1 to -24.3)	0 (0 to 2)	0.4 (0.1 to 1.2)	-38.1 (-58.1 to -24.3)
Qatar	2 (0 to 8)	1.5 (0.2 to 5.5)	-63 (-77.5 to -48)	0 (0 to 2)	0.3 (0 to 1.1)	-63 (-77.5 to -48)
Republic of Korea	121 (28 to 334)	5.6 (1.3 to 15.4)	-47.6 (-66.2 to -35.1)	26 (6 to 72)	1.2 (0.3 to 3.3)	-47.6 (-66.2 to -35.1)
Republic of Moldova	7 (2 to 22)	4.2 (0.9 to 12.8)	-34.5 (-53.2 to -22.7)	2 (0 to 5)	0.9 (0.2 to 2.8)	-34.5 (-53.2 to -22.7)
Romania	26 (5 to 82)	2.7 (0.5 to 8.7)	-39.3 (-59.8 to -28.8)	5 (1 to 17)	0.6 (0.1 to 1.8)	-39.4 (-59.8 to -28.8)

Russian Federation	225 (43 to 757)	2.5 (0.5 to 8.3)	-37 (-58.6 to -28.1)	48 (8 to 163)	0.5 (0.1 to 1.8)	-37 (-58.6 to -28.1)
Rwanda	321 (122 to 682)	19.5 (7.4 to 41.4)	-48.3 (-56.7 to -40.4)	69 (23 to 157)	4.2 (1.4 to 9.5)	-47.4 (-70.1 to 4)
Saint Kitts and Nevis	0 (0 to 0)	2 (0.4 to 6.3)	-44.4 (-62.6 to -31.7)	0 (0 to 0)	0.4 (0.1 to 1.4)	-44.4 (-62.6 to -31.7)
Saint Lucia	0 (0 to 1)	2.9 (0.7 to 8.2)	-43.3 (-58.1 to -31.5)	0 (0 to 0)	0.6 (0.1 to 1.9)	-43.3 (-58.1 to -31.5)
Saint Vincent and the Grenadines	0 (0 to 1)	3.3 (0.7 to 9.2)	-43.2 (-65.1 to -32.4)	0 (0 to 0)	0.7 (0.1 to 2.1)	-43.2 (-65.1 to -32.4)
Samoa	6 (2 to 12)	27.4 (10.5 to 59)	-10.5 (-21.2 to 0.2)	1 (0 to 3)	5.9 (1.6 to 14.4)	-9.5 (-60.8 to 97.7)
San Marino	0 (0 to 0)	3.7 (0.7 to 11.6)	-12 (-30.1 to 5.6)	0 (0 to 0)	0.8 (0.1 to 2.5)	-12 (-30.1 to 5.6)
Sao Tome and Principe	4 (1 to 8)	15.4 (5.6 to 32.9)	-47.7 (-56.8 to -39.6)	1 (0 to 2)	3.3 (1.1 to 7.4)	-46.8 (-67.6 to 5.5)
Saudi Arabia	34 (4 to 129)	1.5 (0.2 to 5.6)	-83.4 (-95.5 to -73.5)	7 (1 to 28)	0.3 (0 to 1.2)	-83.4 (-95.5 to -73.5)
Senegal	507 (189 to 1063)	23.6 (8.8 to 49.4)	-37.6 (-46.3 to -28.1)	106 (26 to 257)	4.9 (1.2 to 11.9)	-38.1 (-75.2 to 45.1)
Serbia	12 (2 to 41)	2.7 (0.5 to 9)	-39.4 (-67.7 to -25.4)	3 (0 to 9)	0.6 (0.1 to 1.9)	-39.4 (-67.7 to -25.4)
Seychelles	2 (1 to 4)	25.6 (9.6 to 54.1)	-42.4 (-53.1 to -32.4)	0 (0 to 1)	5.4 (1.5 to 12.8)	-42 (-74.7 to 12.8)
Sierra Leone	215 (79 to 473)	17.5 (6.4 to 38.5)	-40.2 (-54 to -28.1)	46 (12 to 112)	3.7 (1 to 9.1)	-39.6 (-73.7 to 31.4)
Singapore	17 (4 to 49)	6 (1.5 to 16.8)	-40.6 (-54.6 to -29.4)	4 (1 to 11)	1.3 (0.3 to 3.7)	-40.6 (-54.6 to -29.4)
Slovakia	6 (1 to 21)	2.2 (0.4 to 7.3)	-46.1 (-71.8 to -32.8)	1 (0 to 4)	0.5 (0.1 to 1.5)	-46.1 (-71.8 to -32.8)
Slovenia	2 (0 to 7)	2 (0.3 to 6.8)	-37 (-56.5 to -22.5)	0 (0 to 1)	0.4 (0.1 to 1.5)	-37 (-56.5 to -22.5)
Solomon Islands	46 (19 to 96)	48.3 (19.9 to 99.5)	-30.9 (-40.4 to -21.6)	10 (3 to 22)	10.2 (3.2 to 22.4)	-31.7 (-60.9 to 10.6)
Somalia	2447 (1020 to 4789)	65.6 (27.3 to 128.3)	-16.2 (-27.6 to -5.2)	512 (160 to 1164)	13.7 (4.3 to 31.2)	-16.1 (-57 to 37.4)
South Africa	1756 (685 to 3545)	34.5 (13.5 to 69.6)	-44.8 (-50.3 to -39.5)	372 (134 to 797)	7.3 (2.6 to 15.6)	-44.8 (-58.8 to -30.5)

South Sudan	351 (137 to 717)	23.1 (9.1 to 47.3)	-39.1 (-48.4 to -28.7)	74 (20 to 171)	4.9 (1.3 to 11.3)	-38.4 (-74.9 to 26.2)
Spain	118 (26 to 324)	5.9 (1.3 to 16.1)	-24.7 (-39.3 to -11.8)	25 (5 to 73)	1.3 (0.3 to 3.7)	-24.7 (-39.3 to -11.8)
Sri Lanka	582 (223 to 1170)	37.7 (14.4 to 75.7)	-51.5 (-59.9 to -44.2)	123 (39 to 280)	8 (2.5 to 18.1)	-51.7 (-74.6 to -21)
Sudan	228 (45 to 736)	4.1 (0.8 to 13.4)	-69.1 (-85.5 to -57.4)	49 (9 to 160)	0.9 (0.2 to 2.9)	-69.1 (-85.5 to -57.4)
Suriname	1 (0 to 4)	3.1 (0.7 to 8.8)	-39.2 (-56.2 to -26.6)	0 (0 to 1)	0.7 (0.1 to 2.1)	-39.2 (-56.2 to -26.6)
Sweden	12 (2 to 42)	2.1 (0.4 to 7.1)	-10.8 (-24.1 to 4.6)	3 (0 to 9)	0.4 (0.1 to 1.5)	-10.8 (-24.1 to 4.6)
Switzerland	14 (3 to 46)	3.1 (0.6 to 10.2)	-7 (-18.1 to 6.1)	3 (1 to 10)	0.7 (0.1 to 2.2)	-7 (-18.1 to 6.1)
Syrian Arab Republic	32 (6 to 110)	2.8 (0.5 to 9.7)	-63 (-83.2 to -48.5)	7 (1 to 23)	0.6 (0.1 to 2.1)	-63 (-83.2 to -48.5)
Taiwan (Province of China)	96 (33 to 214)	9.8 (3.4 to 21.7)	-41.6 (-52.9 to -32.4)	21 (7 to 47)	2.1 (0.7 to 4.8)	-41.6 (-52.9 to -32.4)
Tajikistan	78 (21 to 213)	6.4 (1.7 to 17.7)	-29.3 (-45.8 to -16.5)	17 (4 to 45)	1.4 (0.4 to 3.8)	-29.3 (-45.8 to -16.5)
Thailand	652 (243 to 1389)	20.9 (7.8 to 44.4)	-49.9 (-58.5 to -42.3)	139 (37 to 311)	4.5 (1.2 to 10)	-49.1 (-77.8 to 7.5)
Timor-Leste	94 (39 to 188)	53.6 (22 to 107.1)	-56.8 (-64.6 to -50)	20 (7 to 43)	11.3 (3.9 to 24.6)	-56.7 (-73.5 to -38.9)
Togo	226 (87 to 464)	20.2 (7.8 to 41.6)	-37.9 (-48.9 to -27.9)	48 (12 to 112)	4.3 (1.1 to 10)	-37.2 (-69.5 to 30.4)
Tokelau	0 (0 to 0)	20.6 (7.6 to 44.2)	-41.2 (-49.8 to -33.7)	0 (0 to 0)	4.4 (1.2 to 10.1)	-40.4 (-69.6 to 18.6)
Tonga	3 (1 to 6)	22.4 (8.4 to 50.1)	-31.3 (-40.8 to -21.5)	1 (0 to 1)	4.8 (1.1 to 12.3)	-30.7 (-74.6 to 44.5)
Trinidad and Tobago	2 (0 to 5)	2 (0.4 to 6.2)	-40.5 (-66.7 to -27.5)	0 (0 to 1)	0.4 (0.1 to 1.4)	-40.5 (-66.7 to -27.5)
Tunisia	21 (3 to 72)	2.4 (0.4 to 8.3)	-61.7 (-80.6 to -48.5)	4 (1 to 15)	0.5 (0.1 to 1.8)	-61.7 (-80.6 to -48.5)
Turkey	85 (12 to 307)	1.7 (0.2 to 6.3)	-66 (-86.4 to -53.4)	18 (3 to 67)	0.4 (0.1 to 1.4)	-66 (-86.4 to -53.4)
Turkmenistan	20 (4 to 62)	3.6 (0.8 to 11.2)	-46.4 (-64.9 to -33.8)	4 (1 to 13)	0.8 (0.2 to 2.3)	-46.3 (-64.9 to -33.8)
Tuvalu	0 (0 to 1)	25.3 (9.5 to 53.1)	-36.4 (-46 to -28.5)	0 (0 to 0)	5.3 (1.5 to 12.3)	-36.5 (-71.8 to 30.9)

Uganda	483 (154 to 1170)	6.8 (2.2 to 16.5)	-62.8 (-75.3 to -52.6)	104 (32 to 262)	1.5 (0.5 to 3.7)	-62.6 (-77.9 to -45.7)
Ukraine	64 (13 to 210)	3 (0.6 to 9.8)	-29.2 (-51.9 to -17.2)	14 (3 to 46)	0.6 (0.1 to 2.1)	-29.1 (-51.9 to -17.2)
United Arab Emirates	4 (1 to 15)	1.2 (0.2 to 4.4)	-65.6 (-81.3 to -51.4)	1 (0 to 3)	0.3 (0 to 0.9)	-65.6 (-81.3 to -51.4)
United Kingdom	680 (238 to 1508)	17.4 (6.1 to 38.7)	-1.1 (-16.6 to 20.7)	145 (48 to 339)	3.7 (1.2 to 8.7)	-1.1 (-17.5 to 21.9)
United Republic of Tanzania	1010 (362 to 2294)	10.9 (3.9 to 24.6)	-46.5 (-59.5 to -35.7)	217 (70 to 505)	2.3 (0.8 to 5.4)	-45.9 (-66.9 to -12)
United States of America	989 (198 to 3007)	5.2 (1 to 15.8)	2.6 (-4.6 to 14.4)	212 (42 to 649)	1.1 (0.2 to 3.4)	2.6 (-4.6 to 14.4)
United States Virgin Islands	0 (0 to 0)	1.6 (0.3 to 4.9)	-46.2 (-63.4 to -33.7)	0 (0 to 0)	0.3 (0.1 to 1.1)	-46.2 (-63.4 to -33.7)
Uruguay	22 (6 to 57)	9.4 (2.5 to 24.7)	-23.2 (-37.7 to -10.2)	5 (1 to 13)	2 (0.5 to 5.5)	-23.2 (-37.7 to -10.2)
Uzbekistan	164 (36 to 483)	4.5 (1 to 13.3)	-44.2 (-63.7 to -31.4)	35 (8 to 104)	1 (0.2 to 2.9)	-44.2 (-63.7 to -31.4)
Vanuatu	14 (5 to 28)	35.9 (14.1 to 73.9)	-30.1 (-39.3 to -21.6)	3 (1 to 7)	7.5 (2.3 to 17.3)	-31 (-66.7 to 40.1)
Venezuela (Bolivarian Republic of)	86 (20 to 236)	3.7 (0.9 to 10.3)	-30.7 (-48.2 to -18.5)	18 (4 to 54)	0.8 (0.2 to 2.4)	-30.7 (-48.2 to -18.5)
Viet Nam	1773 (689 to 3809)	25.6 (9.9 to 54.9)	-59.6 (-68 to -51.5)	380 (103 to 870)	5.5 (1.5 to 12.5)	-59.1 (-81.7 to -24.4)
Yemen	262 (57 to 821)	5.9 (1.3 to 18.4)	-63.9 (-79.3 to -53.1)	56 (11 to 174)	1.3 (0.2 to 3.9)	-63.9 (-79.3 to -53.1)
Zambia	431 (165 to 938)	15.3 (5.9 to 33.3)	-53.9 (-62.4 to -46.5)	92 (32 to 209)	3.3 (1.1 to 7.4)	-52.8 (-70.7 to -16.1)
Zimbabwe	285 (100 to 651)	13.5 (4.8 to 30.8)	-27.8 (-41 to -16.1)	61 (20 to 141)	2.9 (0.9 to 6.7)	-27.8 (-41 to -16.1)

UI=uncertainty interval.

Supplementary Table 3. The prevalence and YLD for complete hearing loss younger caused by congenital birth defects in children younger than 5 years for both sexes in 1990, and percentage change of counts by 204 countries, 1990-2019.

	Prevalence (95% Uncertainty Interval)			YLD (95% Uncertainty Interval)		
	Counts	Rates per 100,000 population (95%UI)	Percentage change in counts per 100,000 population (95%UI)	Counts	Rates per 100,000 population (95%UI)	Percentage change in counts per 100,000 population(95%UI)
Afghanistan	325 (98 to 820)	16 (5 to 41)	63.8 (10.5 to 101.3)	70 (20 to 184)	3 (1 to 9)	63.8 (10.5 to 101.3)
Albania	25 (7 to 68)	6 (2 to 17)	-76.7 (-84.4 to -71.5)	5 (1 to 15)	1 (0 to 4)	-76.7 (-84.4 to -71.5)
Algeria	246 (62 to 712)	7 (2 to 19)	-55.1 (-76.6 to -39.7)	53 (12 to 159)	1 (0 to 4)	-55.1 (-76.6 to -39.7)
American Samoa	2 (1 to 4)	23 (9 to 49)	-50.1 (-58.5 to -43.8)	0 (0 to 1)	5 (1 to 11)	-48.7 (-68.9 to -8.4)
Andorra	0 (0 to 0)	4 (1 to 12)	-9.4 (-33.9 to 5.2)	0 (0 to 0)	1 (0 to 3)	-9.3 (-33.9 to 5.2)
Angola	443 (168 to 919)	22 (8 to 46)	9.3 (-18.3 to 35.5)	95 (25 to 218)	5 (1 to 11)	9.5 (-38.4 to 111.4)
Antigua and Barbuda	0 (0 to 1)	3 (1 to 9)	-52.2 (-67.8 to -44)	0 (0 to 0)	1 (0 to 2)	-52.2 (-67.8 to -44)
Argentina	414 (128 to 1000)	12 (4 to 29)	-24.1 (-36.8 to -12.9)	89 (26 to 220)	3 (1 to 6)	-24.2 (-36.8 to -12.9)
Armenia	25 (7 to 67)	7 (2 to 18)	-70 (-80.4 to -62.8)	5 (1 to 15)	1 (0 to 4)	-70 (-80.4 to -62.8)
Australia	66 (17 to 182)	5 (1 to 14)	-7.1 (-30.2 to 10.7)	14 (3 to 39)	1 (0 to 3)	-7.1 (-30.2 to 10.7)
Austria	19 (4 to 59)	4 (1 to 13)	-21.7 (-41 to -5.7)	4 (1 to 13)	1 (0 to 3)	-21.7 (-41 to -5.7)
Azerbaijan	51 (14 to 143)	6 (2 to 16)	-42.5 (-56.1 to -33.3)	11 (3 to 31)	1 (0 to 3)	-42.5 (-56.1 to -33.3)
Bahamas	1 (0 to 2)	2 (1 to 8)	-40.4 (-53.8 to -28.8)	0 (0 to 0)	1 (0 to 2)	-40.4 (-53.8 to -28.8)
Bahrain	3 (1 to 8)	4 (1 to 13)	-49 (-75.1 to -33.9)	1 (0 to 2)	1 (0 to 3)	-49 (-75.1 to -33.9)
Bangladesh	4463 (1561 to 10320)	24 (9 to 56)	-69.9 (-81.3 to -62.3)	931 (207 to 2265)	5 (1 to 12)	-69.1 (-84.5 to -36.5)
Barbados	1 (0 to 2)	3 (1 to 8)	-48.5 (-68.8 to -39.1)	0 (0 to 0)	1 (0 to 2)	-48.4 (-68.8 to -39.1)
Belarus	51 (12 to 149)	6 (2 to 18)	-61 (-77.3 to -52.1)	11 (2 to 31)	1 (0 to 4)	-61.1 (-77.3 to -52.1)
Belgium	33 (7 to 92)	5 (1 to 15)	-21 (-36 to -8.1)	7 (1 to 21)	1 (0 to 3)	-21 (-36 to -8.1)
Belize	2 (1 to 5)	7 (2 to 18)	-36.6 (-56.1 to -19.3)	0 (0 to 1)	2 (0 to 4)	-36.6 (-56.1 to -19.3)

Benin	398 (158 to 798)	40 (16 to 81)	35.7 (16.6 to 57.3)	83 (27 to 189)	8 (3 to 19)	39.1 (-35.2 to 179.9)
Bermuda	0 (0 to 0)	3 (1 to 7)	-61.7 (-75.2 to -54.3)	0 (0 to 0)	1 (0 to 2)	-61.7 (-75.2 to -54.3)
Bhutan	24 (8 to 55)	25 (9 to 58)	-69.3 (-79.1 to -62.2)	5 (1 to 13)	5 (1 to 13)	-68.3 (-83 to -36.3)
Bolivia (Plurinational State of)	56 (12 to 163)	5 (1 to 16)	-4.4 (-26.6 to 11)	12 (3 to 35)	1 (0 to 3)	-4.4 (-26.6 to 11)
Bosnia and Herzegovina	23 (6 to 60)	6 (2 to 17)	-80.2 (-89.4 to -74.4)	5 (1 to 13)	1 (0 to 4)	-80.1 (-89.4 to -74.4)
Botswana	78 (32 to 156)	40 (16 to 80)	-48.2 (-60 to -38.8)	16 (5 to 36)	8 (3 to 19)	-47.3 (-68.2 to -11.4)
Brazil	1014 (306 to 2415)	6 (2 to 14)	-46.8 (-55.7 to -41.1)	217 (64 to 530)	1 (0 to 3)	-46.8 (-55.7 to -41.1)
Brunei Darussalam	4 (1 to 10)	11 (3 to 28)	-41.2 (-53.9 to -32.2)	1 (0 to 2)	2 (1 to 6)	-41.2 (-53.9 to -32.2)
Bulgaria	24 (5 to 69)	4 (1 to 13)	-64.4 (-78.5 to -57.9)	5 (1 to 15)	1 (0 to 3)	-64.3 (-78.5 to -57.9)
Burkina Faso	1000 (416 to 1975)	54 (22 to 106)	42.2 (20.5 to 61)	208 (63 to 445)	11 (3 to 24)	41.4 (-28.3 to 162)
Burundi	497 (200 to 997)	45 (18 to 91)	34.2 (15.1 to 51.9)	105 (35 to 226)	10 (3 to 21)	33.3 (-32.7 to 147.3)
Cabo Verde	17 (7 to 34)	30 (11 to 60)	-56 (-66.2 to -48.3)	4 (1 to 8)	6 (2 to 15)	-55.5 (-72.8 to -16.8)
Cambodia	2339 (1039 to 4376)	124 (55 to 231)	-55.4 (-63 to -49.2)	489 (178 to 970)	26 (9 to 51)	-55 (-73.2 to -29.5)
Cameroon	599 (227 to 1212)	30 (12 to 62)	20.6 (-0.8 to 38.1)	126 (34 to 290)	6 (2 to 15)	22.8 (-21.8 to 128)
Canada	108 (22 to 321)	6 (1 to 17)	-21.3 (-44.6 to -8.8)	23 (5 to 69)	1 (0 to 4)	-21.3 (-44.6 to -8.8)
Central African Republic	127 (48 to 263)	25 (9 to 52)	14.8 (-3.2 to 31.8)	26 (7 to 65)	5 (1 to 13)	17.9 (-31.4 to 137.7)
Chad	721 (314 to 1404)	58 (25 to 113)	71.6 (40.7 to 96.4)	151 (54 to 331)	12 (4 to 27)	70.8 (-10.9 to 184.4)
Chile	171 (49 to 428)	12 (3 to 29)	-48.6 (-62 to -39.9)	37 (10 to 93)	3 (1 to 6)	-48.6 (-62 to -39.9)
China	50267 (20397 to 101163)	44 (18 to 88)	-61.7 (-67.6 to -58.2)	10644 (3863 to 22597)	9 (3 to 20)	-61.3 (-70.4 to -53.4)
Colombia	255 (66 to 640)	6 (2 to 15)	-47.9 (-60.1 to -38.3)	55 (14 to 150)	1 (0 to 4)	-47.9 (-60.1 to -38.3)
Comoros	28 (11 to 56)	34 (14 to 68)	-49.7 (-60.5 to -42)	6 (2 to 13)	7 (2 to 16)	-49 (-66.8 to -14.2)
Congo	58 (22 to 129)	14 (5 to 30)	-27.5 (-46.1 to -12.7)	13 (4 to 28)	3 (1 to 7)	-27.5 (-46.1 to -12.7)
Cook Islands	0 (0 to 1)	20 (8 to 44)	-62.4 (-68.7 to -57.3)	0 (0 to 0)	4 (1 to 10)	-62.1 (-77.1 to -31.9)
Costa Rica	19 (5 to 51)	5 (1 to 13)	-49.3 (-63.2 to -39.1)	4 (1 to 11)	1 (0 to 3)	-49.3 (-63.2 to -39.1)
Côte d'Ivoire	798 (313 to 1608)	34 (13 to 69)	2.5 (-14.8 to 18.4)	166 (49 to 375)	7 (2 to 16)	3.1 (-47.9 to 104.1)

Croatia	11 (3 to 34)	4 (1 to 11)	-60.5 (-78.4 to -51.1)	2 (1 to 7)	1 (0 to 2)	-60.5 (-78.4 to -51.1)
Cuba	35 (8 to 95)	4 (1 to 11)	-54.5 (-70.3 to -46.1)	7 (2 to 22)	1 (0 to 2)	-54.5 (-70.3 to -46.1)
Cyprus	5 (1 to 12)	7 (2 to 19)	-29.2 (-58.8 to -15.4)	1 (0 to 3)	2 (0 to 4)	-29.2 (-58.8 to -15.4)
Czechia	23 (5 to 72)	4 (1 to 11)	-49.8 (-71.4 to -38.6)	5 (1 to 15)	1 (0 to 2)	-49.8 (-71.4 to -38.6)
Democratic People's Republic of Korea	1370 (583 to 2656)	48 (21 to 94)	-54.4 (-61.9 to -46.8)	291 (99 to 601)	10 (3 to 21)	-54.9 (-76.9 to -18)
Democratic Republic of the Congo	1555 (585 to 3418)	21 (8 to 45)	10.3 (-12.4 to 32.3)	329 (92 to 757)	4 (1 to 10)	11.6 (-30.5 to 89.6)
Denmark	12 (3 to 37)	4 (1 to 13)	-10.3 (-24.8 to 1.6)	3 (1 to 8)	1 (0 to 3)	-10.4 (-24.8 to 1.6)
Djibouti	32 (13 to 66)	35 (14 to 72)	-16.7 (-32.8 to -2.5)	7 (2 to 16)	7 (2 to 18)	-15.2 (-46.9 to 45.5)
Dominica	0 (0 to 1)	4 (1 to 11)	-71.1 (-82 to -64.9)	0 (0 to 0)	1 (0 to 3)	-71.1 (-82 to -64.9)
Dominican Republic	70 (19 to 170)	7 (2 to 16)	-43.7 (-65.5 to -29.3)	15 (4 to 40)	1 (0 to 4)	-43.7 (-65.5 to -29.3)
Ecuador	141 (39 to 371)	10 (3 to 27)	-24.7 (-47.6 to -10.7)	30 (8 to 81)	2 (1 to 6)	-24.8 (-47.6 to -10.7)
Egypt	617 (148 to 1799)	7 (2 to 21)	-49.3 (-75.1 to -31)	132 (30 to 381)	2 (0 to 4)	-49.3 (-75.1 to -31)
El Salvador	59 (17 to 143)	8 (2 to 19)	-58.7 (-71.5 to -50.4)	13 (4 to 33)	2 (0 to 4)	-58.7 (-71.5 to -50.4)
Equatorial Guinea	25 (10 to 52)	28 (11 to 58)	-66.4 (-78.7 to -56.3)	5 (2 to 13)	6 (2 to 14)	-65.4 (-82.3 to -34.4)
Eritrea	254 (107 to 499)	46 (19 to 90)	-20.2 (-34.3 to -6.4)	53 (16 to 116)	10 (3 to 21)	-18.8 (-65.4 to 60.9)
Estonia	6 (1 to 16)	5 (1 to 14)	-69.7 (-82.7 to -62.6)	1 (0 to 4)	1 (0 to 3)	-69.6 (-82.7 to -62.6)
Eswatini	64 (25 to 125)	44 (17 to 86)	-54.4 (-61.9 to -47.7)	14 (4 to 31)	9 (3 to 22)	-53.8 (-79.1 to -9.8)
Ethiopia	5766 (2335 to 11342)	59 (24 to 115)	-21.8 (-33.2 to -13)	1207 (449 to 2497)	12 (5 to 25)	-21.4 (-45.9 to 5.2)
Fiji	29 (11 to 62)	31 (12 to 65)	-38.7 (-48.2 to -30.6)	6 (2 to 15)	6 (2 to 15)	-38.1 (-69.3 to 29.2)
Finland	8 (1 to 28)	3 (0 to 9)	-34.2 (-55.6 to -22.1)	2 (0 to 6)	1 (0 to 2)	-34.2 (-55.6 to -22.1)
France	217 (46 to 636)	6 (1 to 16)	-22 (-36.5 to -9)	46 (9 to 144)	1 (0 to 4)	-22 (-36.5 to -9)
Gabon	21 (7 to 44)	12 (4 to 26)	-53.7 (-64.4 to -43.4)	4 (1 to 10)	3 (1 to 6)	-53.7 (-64.4 to -43.4)
Gambia	81 (31 to 161)	42 (16 to 83)	-9.3 (-22.5 to 3)	17 (5 to 38)	9 (3 to 19)	-7.8 (-57.8 to 76.6)

Georgia	21 (5 to 60)	5 (1 to 13)	-57.3 (-67 to -49.1)	5 (1 to 13)	1 (0 to 3)	-57.3 (-67 to -49.1)
Germany	177 (34 to 554)	4 (1 to 12)	-25.7 (-36.7 to -15.4)	38 (7 to 125)	1 (0 to 3)	-25.6 (-36.7 to -15.4)
Ghana	652 (252 to 1328)	25 (10 to 50)	-23.6 (-37.4 to -10.1)	137 (36 to 312)	5 (1 to 12)	-21.9 (-53 to 57.4)
Greece	39 (9 to 105)	7 (2 to 19)	-37.3 (-54.9 to -26.9)	8 (2 to 24)	1 (0 to 4)	-37.3 (-54.9 to -26.9)
Greenland	1 (0 to 1)	10 (3 to 26)	-38.3 (-51.9 to -27.3)	0 (0 to 0)	2 (1 to 6)	-38.4 (-51.9 to -27.3)
Grenada	1 (0 to 2)	6 (2 to 15)	-67 (-77.4 to -59.3)	0 (0 to 0)	1 (0 to 3)	-67 (-77.4 to -59.3)
Guam	3 (1 to 6)	17 (7 to 37)	-31.6 (-44.7 to -21.1)	1 (0 to 1)	4 (1 to 8)	-31.6 (-44.7 to -21.1)
Guatemala	160 (50 to 386)	11 (3 to 27)	-35.1 (-55.9 to -20.4)	34 (10 to 85)	2 (1 to 6)	-35.1 (-55.9 to -20.4)
Guinea	566 (239 to 1129)	46 (20 to 93)	11.9 (-6 to 27.6)	118 (38 to 256)	10 (3 to 21)	11.8 (-49 to 112)
Guinea-Bissau	86 (34 to 175)	45 (18 to 93)	-10.9 (-25.7 to 2.4)	18 (6 to 40)	9 (3 to 21)	-11 (-63.7 to 75.1)
Guyana	6 (2 to 15)	6 (2 to 15)	-59.5 (-71.4 to -52.2)	1 (0 to 4)	1 (0 to 3)	-59.5 (-71.4 to -52.2)
Haiti	106 (31 to 252)	10 (3 to 24)	-8.9 (-34 to 8)	23 (7 to 58)	2 (1 to 6)	-9 (-34 to 8)
Honduras	87 (27 to 206)	11 (3 to 25)	-23.6 (-49.9 to -9.4)	19 (6 to 47)	2 (1 to 6)	-23.6 (-49.9 to -9.4)
Hungary	25 (6 to 75)	4 (1 to 12)	-58.4 (-80.3 to -48.4)	5 (1 to 16)	1 (0 to 3)	-58.4 (-80.3 to -48.4)
Iceland	1 (0 to 3)	5 (1 to 15)	-24.5 (-46.2 to -13.1)	0 (0 to 1)	1 (0 to 3)	-24.5 (-46.2 to -13.1)
India	27043 (9555 to 59994)	23 (8 to 50)	-65.9 (-77.4 to -57.2)	5657 (1878 to 12726)	5 (2 to 11)	-65.1 (-77.3 to -54.1)
Indonesia	8847 (3503 to 18153)	39 (15 to 80)	-48 (-54.1 to -43.5)	1868 (663 to 4042)	8 (3 to 18)	-47.6 (-61 to -33)
Iran (Islamic Republic of)	534 (118 to 1623)	6 (1 to 18)	-74.4 (-90.1 to -64.8)	115 (25 to 349)	1 (0 to 4)	-74.4 (-90.1 to -64.8)
Iraq	228 (52 to 644)	7 (2 to 20)	-49.9 (-74.7 to -29.2)	49 (11 to 141)	2 (0 to 4)	-50 (-74.7 to -29.2)
Ireland	16 (4 to 47)	6 (1 to 16)	-24.4 (-43.3 to -11.7)	3 (1 to 10)	1 (0 to 4)	-24.4 (-43.3 to -11.7)
Israel	31 (7 to 86)	6 (1 to 17)	48.9 (4.1 to 72.8)	7 (1 to 20)	1 (0 to 4)	48.8 (4.1 to 72.8)
Italy	175 (36 to 509)	6 (1 to 19)	-32.9 (-47.8 to -26.5)	37 (8 to 113)	1 (0 to 4)	-32.9 (-47.8 to -26.5)
Jamaica	13 (3 to 33)	5 (1 to 12)	-61.6 (-72.1 to -53.1)	3 (1 to 8)	1 (0 to 3)	-61.6 (-72.1 to -53.1)
Japan	382 (91 to 1037)	6 (1 to 16)	-41.5 (-47.6 to -38.2)	82 (19 to 234)	1 (0 to 4)	-41.5 (-47.6 to -38.2)

Jordan	31 (7 to 96)	5 (1 to 15)	-24.5 (-66.1 to 3.7)	7 (1 to 20)	1 (0 to 3)	-24.5 (-66.1 to 3.7)
Kazakhstan	102 (25 to 291)	5 (1 to 16)	-38.7 (-56.5 to -27.2)	22 (5 to 61)	1 (0 to 3)	-38.7 (-56.5 to -27.2)
Kenya	1994 (835 to 3877)	46 (19 to 89)	8.4 (2 to 12.7)	420 (158 to 864)	10 (4 to 20)	9.1 (-6.2 to 22.8)
Kiribati	5 (2 to 10)	46 (18 to 91)	13.3 (-1 to 29)	1 (0 to 3)	10 (3 to 22)	12.9 (-40.5 to 111.7)
Kuwait	6 (1 to 19)	3 (1 to 9)	-32.8 (-57.3 to -15.9)	1 (0 to 4)	1 (0 to 2)	-32.8 (-57.3 to -15.9)
Kyrgyzstan	45 (12 to 123)	7 (2 to 20)	-8.3 (-26 to 3.5)	10 (2 to 27)	2 (0 to 4)	-8.3 (-26 to 3.5)
Lao People's Democratic Republic	835 (375 to 1561)	120 (54 to 225)	-45.6 (-54.7 to -36.4)	175 (67 to 349)	25 (10 to 50)	-44.7 (-67.7 to -15.7)
Latvia	9 (2 to 27)	4 (1 to 13)	-70 (-84.6 to -63.3)	2 (0 to 6)	1 (0 to 3)	-69.9 (-84.6 to -63.3)
Lebanon	28 (7 to 84)	6 (1 to 17)	-65.3 (-87.9 to -50.3)	6 (1 to 18)	1 (0 to 4)	-65.3 (-87.9 to -50.3)
Lesotho	160 (67 to 314)	59 (25 to 116)	-62.8 (-70.4 to -56.7)	33 (11 to 73)	12 (4 to 27)	-61.8 (-82.9 to -28.2)
Liberia	124 (50 to 251)	38 (15 to 76)	25 (7 to 43.2)	26 (7 to 58)	8 (2 to 17)	24.6 (-52 to 150.5)
Libya	38 (9 to 115)	5 (1 to 17)	-80 (-92.1 to -72.2)	8 (2 to 25)	1 (0 to 4)	-80 (-92.1 to -72.2)
Lithuania	13 (3 to 40)	5 (1 to 14)	-74 (-84.6 to -67.6)	3 (1 to 8)	1 (0 to 3)	-74 (-84.6 to -67.6)
Luxembourg	1 (0 to 3)	4 (1 to 12)	27.3 (4.8 to 47.7)	0 (0 to 1)	1 (0 to 3)	27.2 (4.8 to 47.7)
Madagascar	3226 (1433 to 6016)	147 (65 to 274)	-15.5 (-29.1 to -3)	675 (256 to 1380)	31 (12 to 63)	-14.4 (-48.8 to 30)
Malawi	848 (345 to 1652)	47 (19 to 92)	-25.9 (-41.2 to -13.7)	178 (59 to 389)	10 (3 to 22)	-26.1 (-71.2 to 39.6)
Malaysia	1155 (475 to 2264)	48 (20 to 95)	-47.9 (-58.4 to -39.7)	242 (80 to 537)	10 (3 to 22)	-47.3 (-79.7 to 6.6)
Maldives	42 (19 to 81)	101 (45 to 195)	-59.5 (-66.6 to -53.2)	9 (3 to 17)	21 (8 to 42)	-59.1 (-78.7 to -31.6)
Mali	954 (392 to 1921)	55 (23 to 111)	51.5 (27.7 to 74.2)	199 (66 to 425)	12 (4 to 25)	49.6 (-29.5 to 154.5)
Malta	2 (0 to 6)	7 (2 to 20)	-46.5 (-61.4 to -37.2)	0 (0 to 1)	2 (0 to 4)	-46.5 (-61.4 to -37.2)
Marshall Islands	4 (1 to 7)	48 (19 to 97)	-50 (-58.6 to -42.3)	1 (0 to 2)	10 (3 to 22)	-49.7 (-75.4 to -2.1)
Mauritania	109 (42 to 219)	29 (11 to 57)	-22.4 (-33.4 to -12.7)	22 (7 to 54)	6 (2 to 14)	-19.7 (-51.5 to 42.9)
Mauritius	53 (22 to 104)	51 (21 to 99)	-68 (-73.8 to -62.9)	11 (4 to 25)	11 (4 to 24)	-67.9 (-85.9 to -41.1)
Mexico	609 (162 to 1551)	5 (1 to 13)	-49.9 (-64.6 to -41.3)	131 (34 to 362)	1 (0 to 3)	-49.9 (-64.6 to -41.3)
Micronesia (Federated States	7 (3 to 14)	40 (16 to 81)	-61.2 (-66.6 to -56.3)	1 (0 to 3)	8 (3 to 19)	-60.6 (-83.8 to -20)

of)						
Monaco	0 (0 to 0)	4 (1 to 12)	26.9 (-16.6 to 58)	0 (0 to 0)	1 (0 to 3)	26.9 (-16.6 to 58)
Mongolia	27 (8 to 71)	8 (2 to 21)	-32.8 (-52.5 to -19.6)	6 (2 to 15)	2 (0 to 5)	-32.7 (-52.5 to -19.6)
Montenegro	2 (0 to 6)	3 (1 to 10)	-55.1 (-77.1 to -46.2)	0 (0 to 1)	1 (0 to 2)	-55.1 (-77.1 to -46.2)
Morocco	334 (86 to 927)	9 (2 to 26)	-64.8 (-81.9 to -54.6)	72 (18 to 197)	2 (1 to 6)	-64.8 (-81.9 to -54.6)
Mozambique	1410 (609 to 2721)	61 (26 to 117)	3.2 (-15.1 to 18.3)	294 (105 to 623)	13 (5 to 27)	4.6 (-49.2 to 83.8)
Myanmar	11641 (5252 to 21684)	201 (91 to 375)	-63.3 (-69.3 to -56.9)	2430 (938 to 4830)	42 (16 to 83)	-63 (-74.6 to -46.8)
Namibia	84 (32 to 174)	38 (14 to 79)	-35.9 (-47.6 to -24.1)	18 (6 to 41)	8 (3 to 19)	-34.6 (-59.5 to 13.2)
Nauru	1 (0 to 1)	30 (11 to 61)	-44.2 (-51.3 to -37.6)	0 (0 to 0)	6 (2 to 15)	-44.3 (-74.4 to 19.6)
Nepal	1000 (348 to 2315)	30 (10 to 69)	-72.2 (-85.5 to -63.2)	209 (54 to 530)	6 (2 to 16)	-71.5 (-86.8 to -46.8)
Netherlands	41 (8 to 123)	4 (1 to 13)	-21.8 (-41.5 to -3.4)	9 (2 to 28)	1 (0 to 3)	-21.8 (-41.5 to -3.4)
New Zealand	12 (3 to 34)	4 (1 to 12)	-20.5 (-51.5 to -8.7)	3 (1 to 7)	1 (0 to 3)	-20.5 (-51.5 to -8.7)
Nicaragua	66 (20 to 161)	10 (3 to 24)	-47.1 (-62.2 to -35.7)	14 (4 to 35)	2 (1 to 5)	-47.2 (-62.2 to -35.7)
Niger	1057 (453 to 2103)	63 (27 to 125)	123.4 (89.7 to 160)	221 (76 to 473)	13 (5 to 28)	123.2 (20.5 to 284.8)
Nigeria	6603 (2752 to 13087)	41 (17 to 82)	33.4 (22.7 to 42.3)	1371 (495 to 3013)	9 (3 to 19)	34.1 (13.4 to 57.3)
Niue	0 (0 to 0)	25 (10 to 51)	-70.7 (-75.2 to -66.1)	0 (0 to 0)	5 (1 to 12)	-70 (-82.3 to -40.3)
North Macedonia	9 (2 to 24)	5 (1 to 14)	-61.7 (-80.9 to -52.5)	2 (0 to 5)	1 (0 to 3)	-61.6 (-80.9 to -52.5)
Northern Mariana Islands	1 (0 to 2)	16 (6 to 36)	-59 (-64.3 to -54.2)	0 (0 to 0)	3 (1 to 8)	-59 (-64.3 to -54.2)
Norway	16 (4 to 44)	6 (1 to 16)	7 (-0.8 to 23.6)	4 (1 to 10)	1 (0 to 4)	6.9 (-0.8 to 23.6)
Oman	23 (5 to 66)	7 (2 to 21)	-72.9 (-90.3 to -59.8)	5 (1 to 14)	2 (0 to 5)	-72.9 (-90.3 to -59.8)
Pakistan	1618 (460 to 4280)	8 (2 to 21)	-20.5 (-43.2 to -3.4)	347 (95 to 908)	2 (0 to 5)	-20.6 (-43.2 to -3.4)
Palau	0 (0 to 1)	22 (8 to 47)	-57 (-64.2 to -51.4)	0 (0 to 0)	5 (1 to 11)	-56.3 (-74 to -15.5)
Palestine	43 (11 to 116)	10 (3 to 28)	-57 (-80.8 to -39.6)	9 (2 to 25)	2 (1 to 6)	-57 (-80.8 to -39.6)
Panama	13 (3 to 34)	5 (1 to 12)	-16.8 (-40.1 to -1.4)	3 (1 to 8)	1 (0 to 3)	-16.9 (-40.1 to -1.4)

Papua New Guinea	431 (174 to 857)	67 (27 to 134)	69.6 (47 to 92.7)	90 (33 to 190)	14 (5 to 30)	71.1 (-5.8 to 191.7)
Paraguay	41 (13 to 100)	7 (2 to 16)	-43 (-57.7 to -32.5)	9 (3 to 21)	1 (0 to 3)	-42.9 (-57.7 to -32.5)
Peru	35 (5 to 124)	1 (0 to 4)	-14.5 (-29.1 to 6.7)	8 (1 to 28)	0 (0 to 1)	-14.5 (-29.1 to 6.7)
Philippines	5633 (2407 to 10984)	60 (26 to 117)	-14.1 (-22.8 to -8.5)	1192 (450 to 2491)	13 (5 to 26)	-13.7 (-26.7 to -2.1)
Poland	113 (26 to 348)	4 (1 to 12)	-65.9 (-80.7 to -58.5)	24 (6 to 73)	1 (0 to 3)	-65.9 (-80.7 to -58.5)
Portugal	48 (12 to 125)	8 (2 to 22)	-49.4 (-62.7 to -41.1)	10 (3 to 28)	2 (0 to 5)	-49.4 (-62.7 to -41.1)
Puerto Rico	8 (2 to 24)	3 (1 to 8)	-74.5 (-82.8 to -68.8)	2 (0 to 6)	1 (0 to 2)	-74.5 (-82.8 to -68.8)
Qatar	2 (0 to 7)	4 (1 to 13)	5.6 (-35.7 to 48.3)	0 (0 to 1)	1 (0 to 3)	5.4 (-35.7 to 48.3)
Republic of Korea	359 (103 to 895)	11 (3 to 27)	-66.2 (-78.3 to -58.2)	77 (22 to 194)	2 (1 to 6)	-66.2 (-78.3 to -58.2)
Republic of Moldova	27 (6 to 79)	6 (2 to 19)	-73.3 (-81 to -68.5)	6 (1 to 17)	1 (0 to 4)	-73.3 (-81 to -68.5)
Romania	80 (18 to 243)	4 (1 to 14)	-68 (-78.8 to -62.5)	17 (4 to 50)	1 (0 to 3)	-68 (-78.8 to -62.5)
Russian Federation	456 (102 to 1424)	4 (1 to 12)	-50.6 (-67.5 to -43.6)	98 (20 to 300)	1 (0 to 3)	-50.6 (-67.5 to -43.6)
Rwanda	510 (205 to 1021)	38 (15 to 75)	-37 (-47.3 to -27.4)	107 (32 to 245)	8 (2 to 18)	-35.9 (-63.5 to 26.7)
Saint Kitts and Nevis	0 (0 to 0)	4 (1 to 10)	-57 (-71.1 to -47.1)	0 (0 to 0)	1 (0 to 2)	-57 (-71.1 to -47.1)
Saint Lucia	1 (0 to 2)	5 (1 to 13)	-71.5 (-79 to -65.6)	0 (0 to 1)	1 (0 to 3)	-71.6 (-79 to -65.6)
Saint Vincent and the Grenadines	1 (0 to 2)	6 (2 to 15)	-66.9 (-79.7 to -60.7)	0 (0 to 0)	1 (0 to 3)	-67 (-79.7 to -60.7)
Samoa	6 (2 to 13)	31 (12 to 63)	-7.3 (-18.5 to 3.7)	1 (0 to 3)	7 (2 to 15)	-6.3 (-59.5 to 104.7)
San Marino	0 (0 to 0)	4 (1 to 13)	1.9 (-19.1 to 22.3)	0 (0 to 0)	1 (0 to 3)	1.9 (-19.1 to 22.3)
Sao Tome and Principe	6 (2 to 12)	29 (11 to 59)	-41.1 (-51.4 to -32)	1 (0 to 3)	6 (2 to 14)	-40.1 (-63.5 to 18.9)
Saudi Arabia	223 (57 to 606)	9 (2 to 24)	-84.9 (-95.9 to -75.9)	48 (12 to 135)	2 (0 to 5)	-84.9 (-95.9 to -75.9)
Senegal	550 (224 to 1107)	38 (15 to 76)	-7.8 (-20.6 to 6.3)	116 (30 to 263)	8 (2 to 18)	-8.5 (-63.3 to 114.5)
Serbia	29 (7 to 85)	4 (1 to 13)	-57.9 (-77.5 to -48.2)	6 (1 to 18)	1 (0 to 3)	-57.9 (-77.5 to -48.2)
Seychelles	4 (2 to 7)	44 (19 to 87)	-47.4 (-57.2 to -38.3)	1 (0 to 2)	9 (3 to 20)	-47.1 (-76.9 to 2.9)
Sierra Leone	189 (76 to 390)	29 (12 to 60)	13.9 (-12.4 to 36.8)	40 (11 to 92)	6 (2 to 14)	15 (-50 to 150.3)

Singapore	21 (6 to 51)	10 (3 to 25)	-15.7 (-35.7 to 0.1)	4 (1 to 11)	2 (1 to 6)	-15.8 (-35.7 to 0.1)
Slovakia	17 (4 to 50)	4 (1 to 12)	-62.3 (-80.3 to -53)	4 (1 to 10)	1 (0 to 3)	-62.3 (-80.3 to -53)
Slovenia	4 (1 to 13)	3 (1 to 10)	-48.3 (-64.3 to -36.4)	1 (0 to 3)	1 (0 to 2)	-48.3 (-64.3 to -36.4)
Solomon Islands	43 (17 to 85)	70 (28 to 139)	8.5 (-6.3 to 23.1)	9 (3 to 20)	15 (5 to 32)	7.4 (-38.5 to 73.7)
Somalia	1014 (440 to 1952)	78 (34 to 151)	141.3 (108.6 to 173.1)	212 (76 to 439)	16 (6 to 34)	141.5 (24 to 295.8)
South Africa	2856 (1196 to 5595)	62 (26 to 122)	-38.5 (-44.7 to -32.6)	606 (228 to 1270)	13 (5 to 28)	-38.5 (-54.1 to -22.6)
South Sudan	382 (154 to 769)	38 (15 to 76)	-8.3 (-22.3 to 7.3)	80 (25 to 186)	8 (2 to 18)	-7.3 (-62.1 to 90)
Spain	162 (41 to 442)	8 (2 to 21)	-27.2 (-41.3 to -14.7)	35 (8 to 99)	2 (0 to 5)	-27.2 (-41.3 to -14.7)
Sri Lanka	1402 (613 to 2708)	78 (34 to 150)	-58.5 (-65.7 to -52.2)	298 (108 to 632)	17 (6 to 35)	-58.6 (-78.2 to -32.3)
Sudan	494 (141 to 1277)	13 (4 to 35)	-53.9 (-78.4 to -36.5)	106 (28 to 283)	3 (1 to 8)	-53.9 (-78.4 to -36.5)
Suriname	2 (1 to 6)	5 (1 to 13)	-36.3 (-54.1 to -23.1)	0 (0 to 1)	1 (0 to 3)	-36.3 (-54.1 to -23.1)
Sweden	13 (2 to 42)	2 (0 to 7)	-5.6 (-19.8 to 10.6)	3 (0 to 9)	0 (0 to 2)	-5.7 (-19.8 to 10.6)
Switzerland	13 (2 to 43)	3 (1 to 11)	4.6 (-7.8 to 19.4)	3 (0 to 9)	1 (0 to 2)	4.6 (-7.8 to 19.4)
Syrian Arab Republic	176 (44 to 495)	8 (2 to 22)	-81.7 (-91.7 to -74.5)	38 (9 to 108)	2 (0 to 5)	-81.7 (-91.7 to -74.5)
Taiwan (Province of China)	270 (99 to 558)	17 (6 to 35)	-64.4 (-71.2 to -58.7)	58 (20 to 125)	4 (1 to 8)	-64.4 (-71.2 to -58.7)
Tajikistan	85 (24 to 221)	9 (3 to 24)	-9.2 (-30.4 to 7.3)	18 (5 to 49)	2 (1 to 5)	-9.2 (-30.4 to 7.3)
Thailand	2236 (911 to 4591)	42 (17 to 86)	-70.8 (-75.8 to -66.4)	469 (138 to 1104)	9 (3 to 21)	-70.3 (-87.1 to -37.4)
Timor-Leste	176 (78 to 333)	124 (55 to 234)	-46.5 (-56.1 to -38)	37 (14 to 76)	26 (10 to 54)	-46.3 (-67.2 to -24.3)
Togo	227 (88 to 466)	33 (13 to 67)	-0.7 (-18.3 to 15.3)	48 (13 to 109)	7 (2 to 16)	0.4 (-51.2 to 108.4)
Tokelau	0 (0 to 0)	35 (14 to 73)	-65.3 (-70.4 to -61)	0 (0 to 0)	7 (2 to 17)	-64.9 (-82.1 to -30.2)
Tonga	4 (2 to 9)	33 (13 to 69)	-37.1 (-45.8 to -28.1)	1 (0 to 2)	7 (2 to 16)	-36.5 (-76.7 to 32.3)
Trinidad and Tobago	4 (1 to 12)	3 (1 to 9)	-62.4 (-79 to -54.2)	1 (0 to 3)	1 (0 to 2)	-62.4 (-79 to -54.2)
Tunisia	70 (16 to 212)	6 (1 to 19)	-70.7 (-85.2 to -60.6)	15 (3 to 47)	1 (0 to 4)	-70.7 (-85.2 to -60.6)
Turkey	386 (79 to 1171)	5 (1 to 15)	-78.1 (-91.2 to -69.9)	83 (16 to 258)	1 (0 to 3)	-78.1 (-91.2 to -69.9)
Turkmenistan	39 (11 to 105)	7 (2 to 18)	-49.7 (-67.1 to -37.9)	8 (2 to 23)	1 (0 to 4)	-49.7 (-67.1 to -37.9)
Tuvalu	1 (0 to 1)	40 (15 to 84)	-48.8 (-56.5 to -42.5)	0 (0 to 0)	8 (3 to 20)	-48.9 (-77.3 to 5.3)

Uganda	660 (258 to 1408)	18 (7 to 39)	-26.9 (-51.4 to -6.8)	141 (47 to 332)	4 (1 to 9)	-26.5 (-56.6 to 6.7)
Ukraine	157 (37 to 482)	4 (1 to 13)	-59.4 (-72.4 to -52.5)	34 (7 to 102)	1 (0 to 3)	-59.3 (-72.4 to -52.5)
United Arab Emirates	8 (2 to 25)	3 (1 to 11)	-49.8 (-72.7 to -29)	2 (0 to 5)	1 (0 to 2)	-49.8 (-72.7 to -29)
United Kingdom	676 (228 to 1532)	18 (6 to 40)	0.5 (-15.3 to 22.7)	145 (46 to 350)	4 (1 to 9)	0.5 (-16.2 to 23.9)
United Republic of Tanzania	988 (395 to 2020)	20 (8 to 42)	2.3 (-22.6 to 22.9)	209 (64 to 474)	4 (1 to 10)	3.5 (-36.7 to 68.3)
United States of America	990 (199 to 3011)	5 (1 to 15)	-0.1 (-7.1 to 11.4)	212 (41 to 651)	1 (0 to 3)	-0.1 (-7.1 to 11.4)
United States Virgin Islands	0 (0 to 1)	3 (1 to 8)	-68.3 (-78.5 to -61)	0 (0 to 0)	1 (0 to 2)	-68.4 (-78.5 to -61)
Uruguay	33 (10 to 82)	12 (4 to 30)	-35.1 (-47.3 to -24.1)	7 (2 to 18)	3 (1 to 7)	-35.1 (-47.3 to -24.1)
Uzbekistan	275 (75 to 715)	8 (2 to 21)	-40.2 (-61.2 to -26.5)	59 (15 to 156)	2 (0 to 5)	-40.3 (-61.2 to -26.5)
Vanuatu	13 (5 to 27)	51 (20 to 103)	1.4 (-11.9 to 13.7)	3 (1 to 6)	11 (3 to 25)	0.1 (-51.7 to 103.2)
Venezuela (Bolivarian Republic of)	137 (36 to 358)	5 (1 to 14)	-37.6 (-53.3 to -26.6)	29 (7 to 82)	1 (0 to 3)	-37.6 (-53.3 to -26.6)
Viet Nam	5743 (2591 to 11118)	63 (29 to 122)	-69.1 (-75.5 to -63)	1216 (408 to 2544)	13 (4 to 28)	-68.8 (-86 to -42.2)
Yemen	464 (138 to 1184)	16 (5 to 42)	-43.5 (-67.6 to -26.6)	99 (28 to 255)	3 (1 to 9)	-43.5 (-67.6 to -26.6)
Zambia	502 (201 to 1017)	33 (13 to 67)	-14.3 (-30.1 to -0.6)	105 (30 to 236)	7 (2 to 16)	-12.4 (-45.6 to 55.9)
Zimbabwe	330 (123 to 719)	19 (7 to 41)	-13.8 (-29.5 to 0.2)	71 (24 to 157)	4 (1 to 9)	-13.7 (-29.5 to 0.2)

Supplementary Table 4 SDI quintile in 2019

SDI quintile	Lower bound	Upper bound
Low SDI	0	0.454743
Low-middle SDI	0.454743	0.607679
Middle SDI	0.607679	0.689504
High-middle SDI	0.689504	0.805129
High SDI	0.805129	1

SDI=Socio-demographic Index.

Supplementary Table 5 The age-specific prevalence and YLD counts in children younger than 5 years by both sexes, females, males, from 1990 to 2019.

		Prevalence counts (95%Uncertainty Interval)			YLD counts (95%Uncertainty Interval)		
		Both	Male	Female	Both	Male	Female
0-6 days	1990	756 (302 to 1548)	422 (169 to 856)	334 (133 to 698)	161 (59 to 342)	90 (33 to 191)	71 (25 to 152)
	1995	687 (273 to 1412)	381 (152 to 769)	306 (121 to 647)	146 (53 to 311)	81 (30 to 171)	65 (24 to 138)
	2000	626 (245 to 1287)	349 (136 to 702)	277 (109 to 586)	133 (47 to 282)	74 (27 to 158)	59 (21 to 125)
	2005	607 (234 to 1260)	333 (129 to 677)	274 (106 to 578)	129 (46 to 274)	71 (26 to 149)	58 (20 to 126)
	2010	524 (201 to 1122)	285 (110 to 590)	239 (93 to 520)	112 (40 to 239)	61 (22 to 127)	51 (18 to 111)
	2015	495 (192 to 1075)	270 (104 to 573)	225 (86 to 498)	106 (37 to 231)	58 (21 to 123)	48 (17 to 106)
	2019	403 (151 to 883)	220 (83 to 473)	182 (68 to 404)	86 (30 to 190)	47 (16 to 102)	39 (14 to 88)
7-27 days	1990	2228 (890 to 4565)	1243 (496 to 251 9)	986 (393 to 2061)	466 (175 to 998)	260 (99 to 553)	206 (77 to 443)
	1995	2028 (805 to 4165)	1123 (449 to 226 7)	905 (358 to 1913)	424 (158 to 916)	235 (87 to 501)	189 (69 to 412)
	2000	1848 (722 to 3798)	1028 (400 to 206 9)	820 (322 to 1733)	387 (143 to 828)	216 (78 to 449)	172 (62 to 376)
	2005	1793 (691 to 3724)	982 (379 to 1999)	812 (313 to 1710)	375 (135 to 805)	205 (74 to 432)	170 (61 to 367)
	2010	1552 (594 to 3324)	843 (325 to 1745)	709 (275 to 1542)	326 (116 to 696)	176 (65 to 373)	149 (52 to 325)
	2015	1468 (568 to 3189)	801 (307 to 1698)	668 (254 to 1477)	310 (110 to 669)	169 (64 to 361)	141 (48 to 312)
	2019	1196 (450 to 2622)	654 (246 to 1403)	542 (201 to 1203)	252 (87 to 556)	137 (47 to 299)	114 (39 to 259)
28-364 days	1990	34810 (13900 to 7138 0)	19402 (7749 to 39 352)	15408 (6149 to 32218)	7332 (2706 to 152 89)	4084 (1546 to 847 4)	3248 (1177 to 6931)
	1995	31827 (12633 to 6548 2)	17614 (7045 to 35 588)	14213 (5624 to 30063)	6705 (2395 to 143 05)	3706 (1362 to 778 8)	2998 (1034 to 6460)
	2000	29109 (11373 to 5989	16192 (6305 to 32	12917 (5072 to 27329)	6127 (2131 to 128	3401 (1223 to 703	2726 (968 to 5916)

		4)	631)		72)	7)	
	2005	28227 (10879 to 58683)	15448 (5968 to 31499)	12779 (4931 to 26955)	5943 (2063 to 12780)	3247 (1149 to 6745)	2695 (942 to 5793)
	2010	24611 (9423 to 52798)	13369 (5162 to 27714)	11241 (4362 to 24469)	5200 (1857 to 11224)	2821 (1016 to 6017)	2379 (834 to 5228)
	2015	23099 (8919 to 50272)	12592 (4823 to 26723)	10508 (3996 to 23293)	4891 (1722 to 10668)	2667 (960 to 5687)	2224 (755 to 4990)
	2019	18963 (7132 to 41600)	10370 (3902 to 22241)	8593 (3192 to 19094)	4014 (1375 to 8801)	2195 (759 to 4768)	1819 (621 to 4145)
1-4 years	1990	140939 (56197 to 290180)	78610 (31387 to 159842)	62330 (24878 to 130807)	29698 (10678 to 63731)	16538 (5937 to 34825)	13160 (4805 to 28518)
	1995	130277 (51697 to 269743)	72182 (28830 to 146144)	58094 (23088 to 123207)	27461 (9866 to 58306)	15191 (5531 to 32100)	12270 (4420 to 26578)
	2000	119223 (46561 to 246207)	66423 (25836 to 134486)	52800 (20831 to 112181)	25145 (8985 to 54451)	14002 (5072 to 29889)	11143 (3970 to 24127)
	2005	116395 (44966 to 243065)	63656 (24574 to 130378)	52739 (20400 to 111779)	24583 (8788 to 52427)	13436 (4840 to 28073)	11148 (3912 to 24176)
	2010	102314 (39125 to 220873)	55562 (21412 to 115668)	46751 (18086 to 102041)	21637 (7602 to 46519)	11725 (4168 to 25123)	9912 (3422 to 21825)
	2015	96320 (37026 to 210703)	52456 (20176 to 111760)	43864 (16667 to 97635)	20449 (7048 to 44499)	11133 (3867 to 24082)	9316 (3153 to 20668)
	2019	81345 (30462 to 178805)	44508 (16747 to 95800)	36837 (13669 to 82288)	17264 (5966 to 37728)	9441 (3264 to 20452)	7822 (2704 to 17757)

Supplementary Table 6 The age-specific prevalence and YLD rates in children younger than 5 years by both sexes, females, males, from 1990 to 2019.

		Prevalence rate per 100,000 population (95%Uncertainty Interval)			YLD rate per 100,000 population (95%Uncertainty Interval)		
		Both	Male	Female	Both	Male	Female
0-6 days	1990	28.9 (11.6 to 59.3)	31.3 (12.5 to 63.4)	26.4 (10.5 to 55.3)	6.2 (2.2 to 13.1)	6.7 (2.5 to 14.1)	5.6 (2 to 12)
	1995	26.8 (10.6 to 55)	28.7 (11.5 to 57.9)	24.7 (9.8 to 52.3)	5.7 (2.1 to 12.1)	6.1 (2.3 to 12.8)	5.3 (1.9 to 11.1)
	2000	24.7 (9.6 to 50.7)	26.5 (10.3 to 53.4)	22.7 (8.9 to 47.9)	5.2 (1.9 to 11.1)	5.6 (2 to 12)	4.8 (1.7 to 10.2)
	2005	23.6 (9.1 to 49)	25 (9.6 to 50.8)	22.1 (8.5 to 46.6)	5 (1.8 to 10.7)	5.3 (1.9 to 11.2)	4.7 (1.6 to 10.2)
	2010	20.1 (7.7 to 43.1)	21.1 (8.2 to 43.8)	19 (7.4 to 41.4)	4.3 (1.5 to 9.2)	4.5 (1.6 to 9.4)	4.1 (1.4 to 8.9)
	2015	18.8 (7.3 to 40.7)	19.8 (7.6 to 42)	17.7 (6.7 to 39.1)	4 (1.4 to 8.7)	4.2 (1.5 to 9)	3.8 (1.3 to 8.4)
	2019	15.7 (5.9 to 34.3)	16.6 (6.2 to 35.6)	14.7 (5.5 to 32.6)	3.3 (1.2 to 7.4)	3.5 (1.2 to 7.7)	3.1 (1.1 to 7.1)
7-27 days	1990	28.9 (11.5 to 59.2)	31.2 (12.5 to 63.3)	26.4 (10.5 to 55.2)	6 (2.3 to 12.9)	6.5 (2.5 to 13.9)	5.5 (2.1 to 11.9)
	1995	26.7 (10.6 to 54.9)	28.6 (11.4 to 57.8)	24.7 (9.8 to 52.2)	5.6 (2.1 to 12.1)	6 (2.2 to 12.8)	5.2 (1.9 to 11.2)
	2000	24.6 (9.6 to 50.6)	26.5 (10.3 to 53.2)	22.6 (8.9 to 47.8)	5.2 (1.9 to 11)	5.6 (2 to 11.6)	4.7 (1.7 to 10.4)
	2005	23.5 (9.1 to 48.9)	24.9 (9.6 to 50.7)	22.1 (8.5 to 46.5)	4.9 (1.8 to 10.6)	5.2 (1.9 to 11)	4.6 (1.7 to 10)
	2010	20.1 (7.7 to 43)	21.1 (8.1 to 43.7)	19 (7.4 to 41.3)	4.2 (1.5 to 9)	4.4 (1.6 to 9.3)	4 (1.4 to 8.7)
	2015	18.7 (7.2 to 40.7)	19.8 (7.6 to 41.9)	17.6 (6.7 to 39)	4 (1.4 to 8.5)	4.2 (1.6 to 8.9)	3.7 (1.3 to 8.2)
	2019	15.6 (5.9 to 34.3)	16.5 (6.2 to 35.5)	14.7 (5.5 to 32.5)	3.3 (1.1 to 7.3)	3.5 (1.2 to 7.6)	3.1 (1.1 to 7)
28-364 days	1990	28.7 (11.5 to 58.9)	31.1 (12.4 to 63)	26.2 (10.5 to 54.8)	6 (2.2 to 12.6)	6.5 (2.5 to 13.6)	5.5 (2 to 11.8)
	1995	26.6 (10.5 to 54.7)	28.5 (11.4 to 57.5)	24.5 (9.7 to 51.9)	5.6 (2 to 11.9)	6 (2.2 to 12.6)	5.2 (1.8 to 11.2)
	2000	24.5 (9.6 to 50.4)	26.3 (10.3 to 53.1)	22.5 (8.8 to 47.6)	5.2 (1.8 to 10.8)	5.5 (2 to 11.4)	4.8 (1.7 to 10.3)
	2005	23.4 (9 to 48.7)	24.8 (9.6 to 50.6)	22 (8.5 to 46.4)	4.9 (1.7 to 10.6)	5.2 (1.8 to 10.8)	4.6 (1.6 to 10)
	2010	20 (7.7 to 42.9)	21 (8.1 to 43.6)	18.9 (7.3 to 41.2)	4.2 (1.5 to 9.1)	4.4 (1.6 to 9.5)	4 (1.4 to 8.8)
	2015	18.6 (7.2 to 40.5)	19.6 (7.5 to 41.6)	17.5 (6.7 to 38.8)	3.9 (1.4 to 8.6)	4.2 (1.5 to 8.9)	3.7 (1.3 to 8.3)
	2019	15.6 (5.9 to 34.2)	16.5 (6.2 to 35.3)	14.6 (5.4 to 32.4)	3.3 (1.1 to 7.2)	3.5 (1.2 to 7.6)	3.1 (1.1 to 7)
1-4 years	1990	28.2 (11.2 to 58)	30.5 (12.2 to 62.1)	25.7 (10.2 to 53.8)	5.9 (2.1 to 12.7)	6.4 (2.3 to 13.5)	5.4 (2 to 11.7)

1995	26 (10.3 to 53.9)	27.9 (11.2 to 56.6)	24 (9.5 to 50.9)	5.5 (2 to 11.7)	5.9 (2.1 to 12.4)	5.1 (1.8 to 11)
2000	24 (9.4 to 49.7)	25.9 (10.1 to 52.5)	22 (8.7 to 46.8)	5.1 (1.8 to 11)	5.5 (2 to 11.7)	4.7 (1.7 to 10.1)
2005	23.2 (9 to 48.5)	24.5 (9.5 to 50.3)	21.8 (8.4 to 46.1)	4.9 (1.8 to 10.5)	5.2 (1.9 to 10.8)	4.6 (1.6 to 10)
2010	19.8 (7.6 to 42.7)	20.8 (8 to 43.2)	18.7 (7.2 to 40.8)	4.2 (1.5 to 9)	4.4 (1.6 to 9.4)	4 (1.4 to 8.7)
2015	18.3 (7 to 39.9)	19.3 (7.4 to 41)	17.2 (6.5 to 38.3)	3.9 (1.3 to 8.4)	4.1 (1.4 to 8.8)	3.7 (1.2 to 8.1)
2019	15.3 (5.7 to 33.7)	16.2 (6.1 to 34.9)	14.4 (5.3 to 32.1)	3.3 (1.1 to 7.1)	3.4 (1.2 to 7.5)	3 (1.1 to 6.9)

Supplementary method

SDI analysis

SDI definition

The Socio-demographic Index (SDI) is a composite indicator of background social and economic conditions that influence health outcomes in each location. In short, it is the geometric mean of 0 to 1 indices of total fertility rate (TFR) for those younger than 25 years old (TFU25), mean education for those 15 years old and older (EDU15+), and lag-distributed income (LDI) per capita. For GBD 2019, after calculating SDI, values were multiplied by 100 for a scale of 0 to 100.

Development of revised SDI indicator

SDI was originally constructed for GBD 2015 by using the Human Development Index (HDI) methodology, wherein a 0 to 1 index value was determined for each of the original three covariate inputs (TFR in ages 15 to 49 years, EDU15+, and LDI per capita) by using the observed minima and maxima over the estimation period to set the scales[1].

In response to feedback from collaborators and the evolution of the GBD, we have refined the indicator with each GBD cycle. Beginning in GBD 2017, along with our expanded estimation of age-specific fertility, we replaced TFR with TFU25 as one of the three component indices. The TFU25 provides a better measure of women's status in society because it focuses on ages at which childbearing disrupts the pursuit of education and entrance into the workforce. In addition, we observed that in highly developed countries, the TFU25 has tended to decline consistently over time despite rebounds in TFR driven by increasing fertility at older ages. The concordance correlation coefficient between SDI based on the GBD 2016 method and the updated method for GBD 2017 was 0.981.

During GBD 2016, we moved from using relative index scales to using absolute scales to enhance the stability of SDI interpretation over time because we noticed that the measure was highly sensitive to the addition of subnational units that tended to stretch the empirical minima and maxima [2]. We selected the minima and maxima of the scales by examining the relationships each of the inputs had with life expectancy at birth and under-5 mortality and by identifying points of limiting returns at both high and low values if they occurred before theoretical limits (eg, a TFU25 of 0) were reached.

Thus, for each covariate input, an index score of 0 represents the minimum level of each covariate in put past which selected health outcomes can get no worse, and an index score of 1 represents the maxi mum level of each covariate input past which selected health outcomes cease to improve. As a compos ite, a location with an SDI of 0 would have a theoretical minimum level of sociodemographic dev elopment relevant to these health outcomes, and a location with an SDI of 1 (before multiplying by 10 0 for reporting) would have a theoretical maximum level of sociodemographic development relevan t to these health outcomes. We computed the index scores underlying SDI as follows:

$$I_{Cly}=\max\left(\frac{C_{ly}-C_{low}}{C_{high}-C_{low}}, 0.005\right)$$

Where:

I_{Cly} is the index for covariate C, location l, and year y and is equal to the difference between the value of that covariate in that location-year and the lower bound of the covariate divided by the difference between the upper and lower bounds for that covariate.

If the values of input covariates fell outside the upper or lower bounds, they were mapped to the respective upper or lower bounds. We also note that the index value for TFU25 was computed as 1 - $I_{TFU25ly}$ because lower TFU25s correspond to higher levels of development and thus higher index scores. For GBD 2019, we expanded the computation of SDI to 1062 national and subnational locations spanning the time period 1950–2019.

The composite SDI is the geometric mean of these three indices for a given location-year. The cut-off values used to determine quintiles for analysis were then computed by using country-level estimates of SDI for the year 2019, excluding countries with populations less than 1 million.

For GBD 2019, final SDI values were multiplied by 100 for reporting, in order to improve understanding of and broader engagement with the values. As such, GBD 2019 SDI is calculated as it was in 2017, but multiplied by 100 at the end (see example calculation below). Final reporting values are on a 0 to 100 scale.

Example calculation

We present the equation used to calculate SDI for a hypothetical country in the year 2010:

$$TFU25 = 1.09;\ Mean\ educ\ yrs\ pc = 8.23;\ lnLDI = 9.60$$

$$I_{TFU25} = 1- \frac{1.09-0}{3-0} = 0.637$$

$$I_{Educ} = \frac{8.23-0}{17-0} = 0.484$$

$$I_{\mathrm{lnLDI}} = \frac{9.60-5.52}{11.00-5.52} = 0.744$$

$$\mathrm{SDI} = \sqrt[3]{I_{TFU25} * I_{Educ} * I_{lnLDI}} = \sqrt[3]{0.637 * 0.484 * 0.744} = 0.611$$

$$I_{lnLDI} = \frac{9.58-5.52}{11.00-5.22} = 0.741$$

$$\mathrm{SDI} = \sqrt[3]{I_{TFR} * I_{Educ} * I_{lnLDI}} = \sqrt[3]{0.855 * 0.543 * 0.741} = 0.701$$

GBD 2019 reporting SDI = 0.701*100 = 70.1

[1] Global, regional, and national life expectancy, all-cause mortality, and cause-specific mortality for 249 causes of death, 1980-2015: a systematic analysis for the Global Burden of Disease Study 2015. Lancet (London, England) 388 (2016) 1459-1544.

[2] Global, regional, and national under-5 mortality, adult mortality, age-specific mortality, and life expectancy, 1970-2016: a systematic analysis for the Global Burden of Disease Study 2016. Lancet (London, England) 390 (2017) 1084-1150.
